# X chromosome genetic data in a Spanish children cohort, dataset description and analysis pipeline

**DOI:** 10.1038/s41597-019-0109-3

**Published:** 2019-07-22

**Authors:** Augusto Anguita-Ruiz, Julio Plaza-Diaz, Francisco Javier Ruiz-Ojeda, Azahara I. Rupérez, Rosaura Leis, Gloria Bueno, Mercedes Gil-Campos, Rocío Vázquez-Cobela, Ramón Cañete, Luis A. Moreno, Ángel Gil, Concepción María Aguilera

**Affiliations:** 1 Department of Biochemistry and Molecular Biology II, School of Pharmacy, University of Granada, Granada, 18011 Spain; 2 Institute of Nutrition and Food Technology “José Mataix”, Center of Biomedical Research, University of Granada, Avda. del Conocimiento s/n, Granada, 18016 Spain; 3 Biosanitary Research Institute of Granada (IBS.GRANADA), University Clinical Hospital San Cecilio, Av. de la Investigación, s/n, Granada, 18016 Spain; 4 CIBEROBN, (Physiopathology of Obesity and Nutrition CB12/03/30038), Institute of Health Carlos III (ISCIII), Madrid, 28029 Spain; 5 Unit of Investigation in Nutrition, Growth and Human Development of Galicia, Pediatric Department (USC). Instituto de Investigación Sanitaria de Santiago de Compostela (IDIS), University Clinical Hospital, Santiago de Compostela, Spain; 6 Growth, Exercise, Nutrition and Development (GENUD) Research Group, Universidad de Zaragoza, Zaragoza, Spain; 7 Instituto Agroalimentario de Aragón (IA2) and Instituto de Investigación Sanitaria de Aragón (IIS Aragón), Zaragoza, Spain; 8 Department of Pediatric Endocrinology, Reina Sofia University Clinical Hospital, Institute Maimónides of Biomedicine Investigation of Córdoba (IMIBIC), University of Córdoba, Avda. Menéndez Pidal s/n, 14004 Córdoba, Spain; 9 Present Address: RG Adipocytes and metabolism, Institute for Diabetes and Obesity, Helmholtz Diabetes Center at Helmholtz Center Munich, Munich, Germany

**Keywords:** Genetic association study, Obesity

## Abstract

X chromosome genetic variation has been proposed as a potential source of missing heritability for many complex diseases, including obesity. Currently, there is a lack of public available genetic datasets incorporating X chromosome genotype data. Although several X chromosome-specific statistics have been developed, there is also a lack of readily available implementations for routine analysis. Here, we aimed: (1) to make public and describe a dataset incorporating phenotype and X chromosome genotype data from a cohort of 915 normal-weight, overweight and obese children, and (2) to deeply describe a whole implementation of the special X chromosome analytic process in genetics. Datasets and pipelines like this are crucial to get familiar with the steps in which X chromosome requires special attention and may raise awareness of the importance of this genomic region.

## Abstract (summary)

**Table:** 

Design Type(s)	disease analysis objective • factorial design • genetic structural variation analysis objective • genotyping design
Measurement Type(s)	genotyping assay
Technology Type(s)	genotyping
Factor Type(s)	sex • experimental condition
Sample Characteristic(s)	Homo sapiens • blood

Machine-accessible metadata file describing the reported data (ISA-Tab format)

## Background & Summary

Overweight and obesity in children are a public health problem that has raised concern worldwide^1^. Childhood obesity is characterized by an expansion of the adipose tissue (AT)^1^ and plays an important role in the development of cardiometabolic alterations during early adulthood, thereby increasing morbidity and mortality^2^. According to twin and family studies, around 40–70% of the interindividual variability in body mass index (BMI) has been attributed to genetic factors^3–5^. Despite this, known single-nucleotide polymorphisms (SNPs) explain <2% of BMI variation^6^, a phenomenon termed ‘missing heritability’. Potential sources explaining this missing heritability include epigenetic components, the existence of low frequency and rare variants as well as the presence of X chromosome genetic variation.

Analysis in current genetic association studies is usually focused on autosomal variants while the sex chromosomes, and specially the X chromosome, are often neglected. Among the reasons, it highlights a lower gene density on the X chromosome, a lower coverage of the region in current genotyping platforms and a number of technical hurdles including complications in genotype calling, imputation and selection of test statistics^7^. According to a previous report, only 242 out of all 743 GWAS conducted from 2005 to 2011 considered the X chromosome in their analyses^7^. The proportion was similar when only family-based GWAS were considered. There is therefore a lack of available public datasets including X chromosome genotype data for analysis. On the other hand, although several X chromosome-specific statistical tests and guidelines have now become available, there is also a lack of readily available implementations and user-friendly apps incorporating them for routine analysis^8,9^.

The majority of the technical hurdles faced when analysing X chromosomal data rise from two of its main particularities. The first one is the fact of women having two allele copies while males having only one. As a consequence, if males are included in the analysis, special caution must be taken. Particularly, the study design process should be performed carefully, trying to maintain a balanced female/male ratio across experimental conditions. Otherwise, many available statistical tests will suffer from type I errors as soon as sex-specific allele frequencies occur, which is typically observed for a great number of variants. Other problems derived from an unbalanced sex ratio in the study sample include problems during the genotype calling process, as the signal intensities obtained from standard array genotyping platforms will be always lower in males than for females (who carry two alleles). The second uniqueness motivating X-chromosome specific analyses lies in the X chromosome inactivation (XCI) process, through which most of the cells of females express only one X chromosome allele in order to compensate the genetic dosage with regard to males. Before selecting a particular statistical approach, it should be mandatory to carefully investigate the concrete XCI model to assume for a gene in a particular tissue. Depending on the XCI model assumed, we should proceed one way or another during the selection of the test statistics. These and other particularities must be addressed as long as X chromosomal data are included into genetic studies.

In relation to obesity, only a few studies have reported association with markers on the X chromosome. One of the most remarkable findings involves the tenomodulin (*TNMD*) gene, a Xq22 located locus encoding a type II transmembrane glycoprotein. First time associated with adult obesity at the genetic level^10^, its presence in adult human AT has been demonstrated showing higher expression in obesity and lower expression after diet-induced weight loss. Regarding children population, our research group found that *TNMD* expression was five fold-times up-regulated in visceral adipose tissue (VAT) of children with obesity, compared with their normal-weight counterparts (Gene Expression Omnibus GSE9624)^11,12^. Recently, we have reported new associations between *TNMD* SNPs and childhood obesity and metabolic alterations in a Spanish children population^13^. Interestingly, our study has been the first to analyse and detect associations between X chromosome *TNMD* genetic variants and obesity in a children cohort. Similarly, SNPs in the *SLC6A14* gene, also located in the X chromosome, have shown evidence of association with obesity^14^. As a whole, these *TNMD* and *SLC6A14* reports support the fact that X chromosome genetic variants may be not only useful early life risk indicators of obesity but also an interesting source of missing heritability^13^.

Given the lack of public available genetic datasets incorporating X chromosome genetic variants and the still prevalent statistical hurdles that make the X chromosome a difficult region to be tested in functional genetics, we here aimed: (1) to make public and describe a dataset incorporating X chromosome genotype data from a children cohort^13,15^, and (2) to outline a whole implementation of the special X chromosome analytic process in genetics. The presented research dataset includes X-chromosomal SNP data (mapping the genes *TNMD* and *SLC6A14*) from a children cohort composed of 915 normal-weight, overweight and obese subjects. Some topics covered in this paper include dataset sharing and description, explanation of sample design, genotype calling, quality control, and test statistics selection procedures. Additionally, a short section explaining and interpreting findings obtained after analysing the dataset with a specific X chromosome analytic approach is presented.

## Methods

### Experimental design and study population

These methods are an expanded version of descriptions in our related work and general characteristics of the dataset have been previously described^13^. Briefly, in this case-control multicentre study, 915 Spanish children (438 males and 477 females) were recruited from three national health institutions: Lozano Blesa University Clinical Hospital, Santiago de Compostela University Clinical Hospital and Reina Sofía University Clinical Hospital. According to specific X-chromosomal analytic requirements, the female/male ratio of the study sample was perfectly balanced.

**Fig 1:**
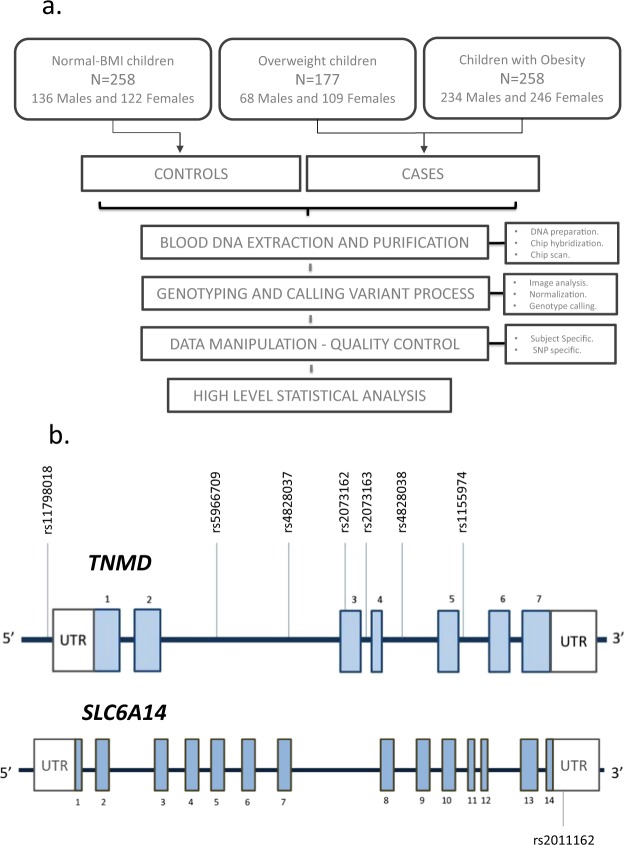
Study design and characteristics. (**a**) Experimental workflow used to generate and analyse the data. (**b**) Genomic context of selected markers; light blue boxes represent exons, while the connecting lines are introns. Abbreviations; rs, reference SNP; UTR, untranslated region.

Childhood obesity status was defined according to the International Obesity Task Force (IOTF) reference for children^16^ which is based on the application, on children population, of the widely used cut-off points of BMI for adults (25 and 30 kg/m^2^, for overweight and obesity respectively). Particularly, these criteria constitute a range of age and sex specific cut-off points for children that have been extracted from solid percentile tables constructed on 97876 boys and 94851 girls ranging from 2 to 18 years. After the application these specific cut-off points, the dataset was composed of 480 children in the obesity group, 177 in the overweight group and 258 in the normal-BMI group. Children were allocated into two experimental conditions according to their obesity status; the affected group (cases) composed of both children with obesity or overweight and the control group composed of normal-weight children. An unbalanced female/male ratio across cases and controls has been proven to heavily affect the power of some specific X chromosome association tests^17^. In our study, a balanced female/male ratio was maintained across each experimental condition (122/136 in controls and 355/302 in cases) (Fig. 1).

Inclusion criteria were European-Caucasian heritage and the absence of congenital metabolic diseases. Otherwise, the exclusion criteria were non-European Caucasian heritage, the presence of congenital metabolic diseases (e.g., diabetes or hyperlipidemia), undernutrition, and the use of medication that alters blood pressure, glucose or lipid metabolism.

### Ethical statement

All procedures in the study were conducted in accordance with the Declaration of Helsinki (Edinburgh 2000 revised), and followed the recommendations of both the Good Clinical Practice of the CEE (Document 111/3976/88 July 1990) and the legally enforced Spanish regulation for clinical investigation of human beings (RD 223/04 about clinical trials). The Ethics Committees on Human Research of all participant institutions approved all experiments and analyses with registration Code: “2011/198”. All parents or guardians provided written informed consent and children gave their assent.

### DNA extraction, processing and analysis

The presented dataset consists on genotype data for eight target SNPs mapping the X-chromosomal genes *TNMD* and *SLC6A14* in the study population. Details regarding SNP selection and molecular analyses are briefly covered here since they have already been fully detailed in our previous work^13^. On the contrary, we pay special attention in the explanation of X chromosomal particularities, data description as well as in the summarization of each data analysis and processing step.

Seven SNPs located at the *TNMD* locus and one located at the *SLC6A14* were selected for genotyping analysis. Genomic DNA was extracted from peripheral white blood cells using two automated kits, the Qiamp DNA Investigator Kit for coagulated samples and the Qiamp DNA Mini & Blood Mini Kit for non-coagulated samples (QIAgen Systems, Inc., Valencia, CA, USA). All extractions were purified using the DNA Clean and Concentrator kit from Zymo Research (Zymo Research, Irvine, CA, USA). Genotyping was performed by TaqMan allelic discrimination assay using the QuantStudio 12 K Flex Real-Time PCR System (Thermo Fisher Scientific, Waltham, MA, USA). Given the X-chromosomal location, it is recommendable to analyse females and males in separate plates during the genotyping process or, at least, maintain a balanced female/male ratio by plate.

Once genotyping was accomplished, we checked candidate SNPs for sex-specific allele frequencies, which can induce type I errors in some statistical X-chromosome analyses (especially in the case of unbalanced designs). Tested by means of the Fisher exact test, all SNPs in the *TNMD* showed no significant P-values and thus equal allele frequencies across sex groups (Table 1). On the contrary, the SNP in the *SLC6A14* did not (P = 0.01). This fact should be taken into consideration when selecting an appropriate test for high-level statistical analyses unless a balanced sex ratio across experimental conditions is presented in the population (which is our case). Information regarding minor allele frequencies (MAFs) stratified by experimental condition for all candidate markers is presented in (Table 2). Linkage disequilibrium (LD) status of the *TNMD* gene was studied using the Haploview Software separately in males and females^13,18^.

**Table 1:** Allele frequencies in the whole study population and each sex group.

CHR	SNP	BP	A1	MAF (All)	MAF (Females)	MAF (Males)	A2	P	OR
23	rs11798018	100584572	A	0.26	0.26	0.27	C	0.84	0.97
23	rs5966709	100589508	T	0.32	0.32	0.31	G	0.75	1.05
23	rs4828037	100590686	C	0.34	0.34	0.33	T	0.53	1.08
23	rs2073162	100594019	A	0.45	0.45	0.42	G	0.37	1.12
23	rs2073163	100594053	C	0.45	0.46	0.43	T	0.35	1.13
23	rs4828038	100596678	T	0.44	0.45	0.42	C	0.31	1.13
23	rs1155974	100598283	T	0.44	0.44	0.42	C	0.59	1.07
**23**	**rs2011162**	**116459132**	**C**	**0.34**	**0.37**	**0.30**	**G**	0.02*	**1.36**

P and OR columns correspond to P-values and odd ratios obtained by means of the Fisher exact test for sex-specific allele frequencies (sex differences in allele frequency per SNP). *P < 0.05. Abbreviations; CHR, Chromosome; SNP, Single Nucleotide Polymorphism; BP, Base Pair; A1, Minor Allele; MAF, Minor Allele Frequency; A2, Alternative Allele.

**Table 2:** Allele frequencies in the study population stratified by experimental condition.

CHR	SNP	GROUP	A1	MAF
23	rs11798018	OVERWEIGHT	A	0.26
23	rs11798018	OBESITY	A	0.27
23	rs11798018	NORMAL-BMI	A	0.28
23	rs5966709	OVERWEIGHT	T	0.29
23	rs5966709	OBESITY	T	0.34
23	rs5966709	NORMAL-BMI	T	0.30
23	rs4828037	OVERWEIGHT	C	0.32
23	rs4828037	OBESITY	C	0.36
23	rs4828037	NORMAL-BMI	C	0.31
23	rs2073162	OVERWEIGHT	A	0.44
23	rs2073162	OBESITY	A	0.46
23	rs2073162	NORMAL-BMI	A	0.43
23	rs2073163	OVERWEIGHT	C	0.45
23	rs2073163	OBESITY	C	0.47
23	rs2073163	NORMAL-BMI	C	0.43
23	rs4828038	OVERWEIGHT	T	0.43
23	rs4828038	OBESITY	T	0.46
23	rs4828038	NORMAL-BMI	T	0.43
23	rs1155974	OVERWEIGHT	T	0.42
23	rs1155974	OBESITY	T	0.45
23	rs1155974	NORMAL-BMI	T	0.43
23	rs2011162	OVERWEIGHT	C	0.34
23	rs2011162	OBESITY	C	0.36
23	rs2011162	NORMAL-BMI	C	0.34

Abbreviations; CHR, Chromosome; SNP, Single Nucleotide Polymorphism; BMI, Body Mass Index; A1, Minor Allele; MAF, Minor Allele Frequency.

## Data Records

The complete research dataset (genotype and phenotype data) has been uploaded into the European Genome-Phenome archive (EGA). The work can be found online with the title “X chromosomal genetic variants are associated with childhood obesity” or with the identifier EGAS00001002738 (2018)^15^. Online data are sorted and presented according to obesity status; the affected group (cases) composed of both children with obesity or overweight (EGA reference EGAD00010001482 (2018)) and the control group composed of normal-weight children (EGA reference EGAD00010001481 (2018)). Three files by-experimental condition (a total of six) are available online (.*bed*, *bim* and *fam* files). The *bed* files contain raw genotype data while the *bim* files describe information relative to target SNPs (chromosome number, SNP identifier, genetic distance in morgans (set as 0 for all markers), base-pair position and coding alleles). Instead, the *fam* files contain information relative to subjects (sample identifiers, family and paternal identifiers (here set as 0), sex (1 for males and 2 for females) and experimental group (1 for control and 2 for cases)). All presented formats can be easily readable in PLINK 1.9 software using the *–bfile* command option and further transformed into a more standard file format with the *–dosage* option^19^.

The complete data set in the current study complies with the requirements of the EGA archive. Detailed information about each sample and shared data files is presented in Online-only Tables 1 and 2, and Supplementary File 1. Specifically, DOI and descriptions for each shared file are provided in the Online-only Table 2.

## Technical Validation

### X chromosome particularities

Before introducing further steps, we here list two issues making the X chromosome a difficult region for genetic analyses. These particularities will determine important decisions related to genotype calling, data imputation and statistical analysis. It is important to note, however, that all here-described particularities are only applicable to those X chromosomal loci outside the pseudo-autosomal region of the X chromosome (which is the case of *TNMD* and *SLC6A14*).

The first noticeable uniqueness of the X chromosome is the fact of women having two allele copies while males having only one. As a result, while females can present the standard three possible allele combinations (AA, AB and BB), males are homozygous and have only two distinct possible genotypes (A- and B-). For this reason, standard autosomal association tests, such as the Cochran-Armitage trend test^20,21^, are not immediately applicable to X chromosome data. The second particularity affecting the X-chromosome analysis lies in the X chromosome inactivation (XCI) process, through which the transcription from one of the two X chromosome copies in female mammalian cells is silenced in order to balance the expression dosage between XX females and XY males. XCI is, however, incomplete in humans: with up to one-third of the X-chromosomal genes escaping from this silencing epigenetic mechanism. The degree of ‘escape’ from inactivation has been reported to strongly vary between genes, tissues and individuals^22,23^, with three possible scenarios at the gene level: complete XCI, partial XCI or total escape from XCI^24,25^. Depending on the XCI model assumed for a certain gene, we should proceed one way or another during the selection of the test statistics (see section ‘High-Level Analysis: Statistical Analysis’ for further details). The assumption of a particular XCI model is therefore a process that must be performed carefully.

Until date, the extent to which XCI is shared between cells and tissues remains poorly characterized and there is a lack of standardized criteria nor well-established databases to check if a gene escapes or not from XCI in a concrete situation. In order to do so, an exhaustive search in PUBMED and other scientific databases should be performed looking for particular studies supporting a certain XCI hypothesis. Currently, the most similar resource to a standardized database on this regard is the initiative carried out by the Genotype-Tissue Expression (GTEx) consortium^9^ in 2017, which describes a systematic survey of XCI, integrating over 5500 transcriptomes from 449 Individuals, spanning 29 tissues from the GTEx (v6p release) and 940 single-cell transcriptomes, combined with genomic sequence data. Particularly, they show that XCI at 683 X-chromosomal genes is generally uniform across human tissues and that incomplete XCI affects at least 23% of X-chromosomal genes. Overall, this work presents an updated catalogue of XCI across human tissues which may be of great utility during the selection of a particular XCI model for a gene. Other available resources also include the work of Slavney *et al*.^26^, which gathers the main XCI insights from previous studies on X-chromosome gene expression datasets.

By way of example, we here illustrate the whole process followed for the identification of the optimal XCI model in the case of *TNMD*. First, we interrogated the Slanvey *et al*. (2016) work^26^, where no evidence of escape from XCI was reported. In order to get more information about this fact, we further studied in detail the three works summarized in the Slanvey *et al*.^26^ paper. The first work on which the paper is based is a study from Carrel *et al*.^22^, in which we could not identify any probe covering the *TNMD* region. Instead, a few surrounding regions were mapped; among which the *SRPX2*, *ZD89B07* and the *SYTL4* reported escaping from the XCI process. In spite of it, this study was based on a fibroblast cell model and thus not applicable to our adipose tissue context. Regarding the second revised article^27^, again, there were not available probes covering *TNMD*. Thus, neither conclusions nor new information could be extracted. In relation to the third included article^28^, we were not able to find any table or supplemental material showing an output list of the analysed regions. Next, we investigated the well-established work from the GTEx consortium^9^ and found that the XCI status of the *TNMD* region remains catalogued as *unknown* (Supplementary Tables S2 and S13 *of this paper*). As a complementary approach, we performed a search in PUBMEP looking for individual studies focused on the gene expression status of *TNMD* from different sexes. As a result, we found a work reporting higher basal expression of *TNMD* in women than in men^29^, which could indicate that *TNMD* escapes from the XCI.

Taking all this into consideration and given the lack of agreement, both possibilities (‘escape from XCI’ and ‘XCI’) should be tested in the case of *TNMD*. A searching process like this is highly recommendable to be done for any X chromosome locus before the selection of a particular statistical approach.

### Raw data processing

The primary step of the data analysis consisted on the extraction of genotype calls from fluorescence array data and the construction of work data files for data manipulation and analysis. Details regarding the exact procedure for genotype calling, which is an important procedure in X-chromosomal analyses, are listed below (‘Genotype Calling’ section).

Once we obtained genotype calls for the 915 individuals, we generated standard format files (*.ped* and .*map*) transforming the ThermoFisher cloud-derived outputs from long to wide format using an own script in R environment^30^. Finally, data were imported into PLINK 1.9 software^19^ and converted into binary format files using the *–make-bed* flag. These binary formats *(.bed*, .*bim* and *.fam*) are a more compact representation of the data that saves space and speeds up subsequent analyses.

### Genotype calling

This is the first step of any primary genotype analysis and consists on the extraction of genotype calls from fluorescence array data at the SNP and individual level. Along with the test statistics selection procedure, the genotype calling process is an analytical step heavily affected by X chromosome particularities. Specifically, the main X chromosome uniqueness affecting this process is the dosage imbalance between males and females. Since males carry only one X allele, signal intensities obtained from the Real-Time PCR System are lower in males than for females and thus a correction should be implemented. On this matter, calling algorithms which apply different models to male and female samples (e.g. Illuminus and CRLMM) have been proven to generally perform better than methods which do not (e.g. GenCall and GenoSNP)^31^.

Here, we employed the Applied Biosystems qPCR app module (Thermo Fisher Cloud software) and the autocalling method for genotype calling. According to literature recommendations, the sex information for each sample was supplied to the software and genotype calling was performed separately in both sexes. In this regard, although genotyped plates did not consist on only boys or girls, the balanced sex ratio of our population (477 females and 438 males) favoured a better performance of the algorithm. Five signal clusters were identified (three in the case of females and two in the case of males). Then, sex information and scatter of the clusters were used to call the genotypes (AA, AB and BB for females, and A- and B- for males). Since the employed software also allows the option of applying user-definable boundaries for data analysis, those samples classified as undetermined by the autocalling method were recalled using the manual option. A set of controls were used to deduce these questionable genotype calls. Outliers were omitted from the analysis.

### Data QC

Prior to high-level statistical analyses, the quality control (QC) process is an important step in any genetic analysis and especially in the X-chromosome analysis. Specific QC guidelines for X chromosome genotype data have been previously reviewed in detail^8^. All these criteria can help us to detect genotype errors or not reliable SNPs which should be excluded from analysis.

Here, the whole QC process was implemented in PLINK 1.9 software^19^. According to literature, two criteria concerning missing frequency were employed (the sex-specific missing frequency and the differential missingness between sexes)^32,33^. As genotype calling was performed separately in males and females (that is, no heterozygote calls in males were allowed), the proportion of heterozygote calls in males, proposed as a filter criterion by Ling and Ziegler *et al*.^32,33^, was not considered in our QC process. All SNPs (with exception of the rs11798018 and the rs2073163 from the T*NMD* gene) passed the recommended missing frequency filter in females (<=2%) (Table 3). On the other hand, none SNP passed the filter in males. Regarding the differential missingness test, the SNPs (rs11798018, rs4828037 and rs2073163) from the *TNMD* and the rs2011162 from the *SLC6A14*, passed the recommended filter (P ≥ 10^−7^). The other SNPs, instead, evidenced a marked differential missingness between sex groups. This test was performed in PLINK software using the flag “*test-missing*” and replacing the phenotype column of the *ped* file by the sex information (Table 3).

**Table 3:** Missing frequency quality control (QC) in our selected markers stratified by sex.

CHR	SNP	MISS FREQ (Males)	MISS FREQ (Females)	MISS FREQ (Males-Females)	P
23	rs11798018	0.11	0.04	0.07	**2.01e-05**
23	rs5966709	0.06	**0.004**	0.06	3.11e-07
23	rs4828037	0.06	**0.01**	0.05	**3.46e-05**
23	rs2073162	0.08	**0.008**	0.07	2.86e-08
23	rs2073163	0.14	0.09	0.05	**0.02**
23	rs4828038	0.07	**0.002**	0.07	4.19e-10
23	rs1155974	0.08	**0.002**	0.07	9.73e-11
23	rs2011162	0.07	**0.02**	0.05	9.30e-05

P column correspond to differential missingness test between sex groups. Asymptotic p-values were obtained by means of Fisher’s exact test. SNPs in bold did pass the QC recommended filters. Abbreviations; CHR, Chromosome; SNP, Single Nucleotide Polymorphism; MISS FREQ, Missing Frequency.

Regarding additional MAF quality checks, all SNPs showed appropriated frequencies >1% by sex groups (Table 1). When analysing the Hardy Weinberg equilibrium (HWE) in girls belonging to the normal-BMI group, all SNPs reported proper values (P ≥ 10^−4^) (Table 4). According to this QC process, we ensured that there were not important genotyping errors and that our genetic data were reliable for further analyses.

**Table 4:** Genotype counts and Hardy-Weinberg test statistics for each SNP in the female group.

CHR	SNP	TEST	A1	GENO	O(HET)	E(HET)	P
23	rs11798018	ALL	A	34/177/249	0.38	0.39	0.72
23	rs11798018	AFF	A	25/133/187	0.38	0.39	0.89
23	rs11798018	UNAFF	A	9/44/61	0.39	0.40	0.81
23	rs5966709	ALL	T	67/175/234	0.37	0.44	0.0005
23	rs5966709	AFF	T	55/128/171	0.36	0.45	0.0005
23	rs5966709	UNAFF	T	12/46/63	0.38	0.41	0.38
23	rs4828037	ALL	C	75/178/219	0.38	0.45	0.0003
23	rs4828037	AFF	C	63/129/158	0.37	0.46	0.0001
23	rs4828037	UNAFF	C	12/48/61	0.40	0.42	0.66
23	rs2073162	ALL	A	132/172/170	0.36	0.50	4.59e-09
23	rs2073162	AFF	A	99/128/124	0.36	0.50	6.78e-07
23	rs2073162	UNAFF	A	33/43/46	0.35	0.49	0.002
23	rs2073163	ALL	C	129/148/156	0.34	0.50	6.92e-011
23	rs2073163	AFF	C	98/112/113	0.35	0.50	3.83e-08
23	rs2073163	UNAFF	C	31/35/43	0.32	0.49	0.0002
23	rs4828038	ALL	T	133/171/173	0.36	0.50	1.56e-09
23	rs4828038	AFF	T	100/128/127	0.36	0.50	2.49e-07
23	rs4828038	UNAFF	T	33/43/46	0.35	0.49	0.002
23	rs1155974	ALL	T	127/172/178	0.36	0.49	4.18e-09
23	rs1155974	AFF	T	94/128/132	0.36	0.49	4.02e-07
23	rs1155974	UNAFF	T	33/43/46	0.35	0.49	0.002
23	rs2011162	ALL	C	84/179/207	0.38	0.47	0.0001
23	rs2011162	AFF	C	62/136/150	0.39	0.47	0.003
23	rs2011162	UNAFF	C	22/42/57	0.35	0.46	0.01

Each SNP has three entries showing results for either ALL individuals, AFF (overweight and children with obesity) or UNAFF (normal-BMI children only). Hardy Weinberg analysis was performed with the exact test described and implemented by Wigginton *et al*.^42^. Abbreviations; CHR, Chromosome; SNP, Single Nucleotide Polymorphism; A1, minor allele; GENO, genotype counts; O(HET), observed heterozygosity; E(HET), expected heterozygosity; P, hardy weinberg P-value.

On this point, it is important to note that since genotyping array technologies are not specially designed for sexual chromosomes, quality is always hoped to be lower on X chromosome genetic variants compared to autosomal data.

### High-level analysis: statistical analysis

As we previously mentioned, most of available test statistics for performing genetic association analyses have been designed for autosomal variants and thus they are not applicable to X chromosome data (especially when dealing with mixed-sex samples). In these cases, testing for association on the X chromosome raises unique challenges that have motivated the development of X-specific statistical tests in the literature^34,35^. Association tests on the X chromosome should incorporate into their models not only the fact of dosage imbalance between males and females but also, depending on the analysed locus, a specific XCI model. Some of available approaches include:

- Clayton Tests (2008)^34^. Clayton tests are two X chromosome specific versions of the common autosomal tests that explicitly account for the XCI process and allow the inclusion of males and females together. In the case of different allele frequencies in males and females, Clayton statistics have inflated type I error frequencies. These tests are available in the R package snpMatrix^36^ with the names:
  - S1^34^: It is analogous to a Cochran-Armitage trend test of a combined male and female genotype contingency table; it follows a Chi² distribution on one degree freedom (df) under the null hypothesis.
  - S2^34^: It is analogous to a Pearson’s Chi² test on 2 df of a combined male and female genotype contingency table, it follows a Chi² distribution on 2 df under the null hypothesis.
- Zheng tests (2007)^35^. They are a set of six different statistics that apply to the same SNP and from which a minimum *P*-value is computed, needing to be adjusted according to the correlation between the test statistics. Zheng *et al*.^35^ showed that the optimal choice of statistic among the six tests depends on whether HWE holds at the locus and whether males and females have the same risk allele. For example, in the case there is departure from HWE in females, the Zheng (Z^2^_mfG_) test has been presented a good choice. For further information regarding test statistic selection, we recommend to read works^8,35^. Of note is that the Zheng’s tests do not explicitly account for the XCI process.

As previously mentioned, an unbalanced female/male ratio between cases and control would affect the relative power of both Zheng and Clayton statistics. If combined with sex-specific allele frequencies, these tests will suffer from increased type I errors.

- Traditional methods easily implementable in PLINK 1.9 or R environment:
  - Ignore males entirely and analyse female data using conventional autosomal tests (a genotypic-based Cochran-Armitage trend test or an allele-based Chi² by Pearson with 1 df). The problem related to this approach is that we are missing all data from male subjects and therefore losing statistical power. The Cochran-Armitage trend test is the default test employed when a naive analysis of X chromosome data is run in PLINK using the flag *–model*^19^. Regarding males, an allele-based test accounting for the number of A- and B- alleles between experimental conditions should be employed apart.
  - Linear or logistic regression analyses on all the samples adjusting by sex. This approach further has the advantage of adjusting the model by covariates of interest. Here, if we assume that the locus of interest escapes from XCI, females should be coded as 0, 1, or 2, according to 0, 1, or 2 number of SNP risk alleles, and males should be coded as 0 or 1 according to 0 or 1 allele copies. On the contrary, if XCI is assumed to occur, females should be coded as 0, 1, or 2, according to 0, 1, or 2 number of SNP risk alleles, and males should be coded as 0 or 2 according to 0 or 1 allele copies. By default, the application of the “*–dosage*” flag to X chromosome input data files (.*bed*, *bim* and *fam*) in PLINK will produce a codification which assumes escape from XCI. For XCI to be considered, new allele code numbers should be manually replaced in male samples with a standard text editor (e.g: gedit software).

In general, the selection of the most suitable test among the presented choices will depend on three different criteria; the XCI model assumed for the locus of interest, deviation from HWE of analysed markers and the existence of sex-specific allele frequencies in the study population, which would be a substantial problem in the case of an unbalanced female/male ratio. Regarding XCI, if inactivation is assumed to occur, then either the Clayton’s statistics or regression models (with males coded as 0 and 2 (for 0 and 1 risk allele, respectively)) would be the tests of choice. On the contrary, in the case of a locus ‘escaping’ from XCI, Zheng’s tests or regression models (with males coded as 0 and 1 (for 0 and 1 risk allele, respectively)) should be employed. In the case of sex-specific allele frequencies, independently of the XCI assumed model, the Zheng’s test (Z^2^_mfG_) has been presented a better choice over the Clayton approach. On the other hand, in the case of an adjustment for covariates is required, only regression models can be applied. Of note is that most of the test statistics and analysis considerations covered here are available to implement in the command-line toolset *XWAS* developed by Keinan A. and collaborators^37–39^.

Although for the analysis of our dataset both possibilities (‘escape from XCI’ and ‘XCI’) were tested in the original work^13^, we here only present results under the XCI assumption. As we have previously seen, selected markers in our sample did not exhibit HWE deviations nor sex-specific allele frequencies. Moreover, the female/male ratio was balanced across experimental groups. For these reasons, and following published recommendations^8,17,34,40^, Clayton test was here selected to perform the main statistical analysis. According to an *in silico* simulation work, the Clayton’s S1 statistic has shown the best performance among all X-specific introduced tests across a wide range of disease models, sex ratios and allele frequencies^40^. Moreover, it allows the inclusion of females and males together, increasing thereby the statistical power.

In Table 5, results derived from the application of Clayton’s S1 and S2 statistics to three different continuous phenotypes of the population are presented. All these phenotype data have also been shared and are available in the metadata file (Online-only Table 1). The implementation of this process was performed in R, using the snpStats R package and the code have been shared online^41^. All reported associations in our previous work^13^ were here replicated under XCI assumption. These findings support therefore a good performance of the Clayton statistics as well as ensure the reliability of the present dataset.

**Table 5:** Association between X chromosome SNPs and HOMA-IR, Glucose and BMI z-score in our dataset.

SNP	N	Chi.squared.1.df	Chi.squared.2.df	P.1df	P.2df
**HOMA-IR**					
rs11798018	811	0.10	1.68	0.74	0.43
rs5966709	849	0.14	2.71	0.70	0.25
rs4828037	844	0.35	2.91	0.55	0.23
**rs2073162**	841	5.48	6.34	**0.01**	**0.04**
**rs2073163**	773	4.78	5.93	**0.02**	0.05
**rs4828038**	849	6.00	7.24	**0.01**	**0.02**
**rs1155974**	844	4.22	6.68	**0.03**	**0.03**
rs2011162	839	0.48	0.91	0.48	0.63
**Glucose (mg/dl)**					
rs11798018	844	0.004	1.06	0.94	0.58
rs5966709	881	0.55	0.59	0.45	0.74
rs4828037	876	1.22	1.25	0.26	0.53
**rs2073162**	873	5.17	8.13	**0.02**	**0.01**
rs2073163	804	2.8	4.006	0.09	0.13
**rs4828038**	880	4.78	6.42	**0.02**	**0.04**
**rs1155974**	876	3.94	4.74	**0.04**	0.09
rs2011162	871	0.92	3.55	0.33	0.16
**BMI z-score**					
rs11798018	845	0.97	1.15	0.32	0.56
rs5966709	881	0.77	1.21	0.37	0.54
rs4828037	877	0.51	0.51	0.47	0.77
**rs2073162**	872	8.61	9.59	**0.003**	**0.008**
**rs2073163**	803	7.09	8.60	**0.007**	**0.01**
**rs4828038**	877	9.02	10.38	**0.002**	**0.005**
**rs1155974**	875	7.75	8.69	**0.005**	**0.01**
rs2011162	871	3.31	5.33	0.06	0.06

SNPs in bold showed statistically significant associations with presented phenotypes under Clayton Statistics. This test explicitly accounts for random X-inactivation and allows the inclusion of females and males together, increasing thereby the statistical power. P.1df and Chi.squared.1.df columns corresponds to Clayton S1 statistic results while P.2df and Chi.squared.2.df corresponds to Clayton S2 statistic. Abbreviations; SNP, Single Nucleotide Polymorphism; N, number of included subjects in the analysis; HOMA-IR, homeostasis model assessment for insulin resistance; BMI z-score, body mass index adjusted by sex and age.

In conclusion, we here share a genetic dataset and present a whole implementation of the special X chromosome analytic process in genetics. Altogether, the pipeline and the shared data will allow researchers to get familiar with the X chromosome particularities and should encourage them to include X chromosome into their genetic studies. Closing this gap is crucial to elucidate the genetic background of complex diseases, especially of those with sex-specific features.

## Supplementary Information

### ISA-Tab metadata file

Download metadata file

### Supplementary Information

Supplementary File 1

## Data Availability

All custom R codes employed in this work have been shared online in a GitHub repository (10.5281/zenodo.2578182)^41^. Two short scripts are available online; “*script_from_long_to_wide.r*” and “*Clayton_analysis_code.r*”. The first one (named “*script_from_long_to_wide.r*”) is a short script designed for loading a genetic dataset (genotype calls) derived from OpenArray technology and transforming it into a handy-format file, which can be further imported into PLINK software. Basically, this script carries out a dataset manipulation and transformation from long to wide format. In order to run the script, users will need an input file derived from OpenArray technology containing information in the long format arranged into three columns (NCBI_SNP_Reference, Sample_ID and Genotype_Call). The second script shared (named “*Clayton_analysis_code.r*”) gathers functions and R commands required for the application of the X-chromosome specific statistical tests developed by Clayton and collaborators^34,36^ (see section ‘High-Level Analysis: Statistical Analysis’ for further details).

## Online-only Tables

**Online-only Table 1:** Samples and Phenotypes. This table collects phenotype data of the samples included in the dataset. Each row represents a unique sample while each column refers to a specific characteristic/attribute of the sample.

Source name	Sample Name	Description	Characteristics [Organism]	Characteristics [Organism_Part]	Characteristics [Cell line]	Characteristics [Gender]	Characteristics [Region]	Characteristics [Case-Control]	Characteristics [Disease_state]	Characteristics [Glucose (mg/dl)]	Characteristics [HOMA-IR]	Characteristics [BMI z-score]
EGAN00001617164	EGAN00001617164	Genomic DNA extracted from peripheral white blood cells using two kits, the Qiamp DNA Investigator Kit for coagulated samples and Qiamp DNA Mini & Blood Mini Kit for non-coagulated samples (QIAgen Systems, Inc., Valencia, CA, USA).	Human	blood	NA	female	Iberian Population in Spain	overweight	Overweight and obesity	82	3.75	1.88
EGAN00001617165	EGAN00001617165	Genomic DNA extracted from peripheral white blood cells using two kits, the Qiamp DNA Investigator Kit for coagulated samples and Qiamp DNA Mini & Blood Mini Kit for non-coagulated samples (QIAgen Systems, Inc., Valencia, CA, USA).	Human	blood	NA	female	Iberian Population in Spain	obese	Overweight and obesity	75	2.5	4.82
EGAN00001617166	EGAN00001617166	Genomic DNA extracted from peripheral white blood cells using two kits, the Qiamp DNA Investigator Kit for coagulated samples and Qiamp DNA Mini & Blood Mini Kit for non-coagulated samples (QIAgen Systems, Inc., Valencia, CA, USA).	Human	blood	NA	female	Iberian Population in Spain	obese	Overweight and obesity	109	15.31	2.87
EGAN00001617167	EGAN00001617167	Genomic DNA extracted from peripheral white blood cells using two kits, the Qiamp DNA Investigator Kit for coagulated samples and Qiamp DNA Mini & Blood Mini Kit for non-coagulated samples (QIAgen Systems, Inc., Valencia, CA, USA).	Human	blood	NA	female	Iberian Population in Spain	overweight	Overweight and obesity	78	2.74	1.6
EGAN00001617168	EGAN00001617168	Genomic DNA extracted from peripheral white blood cells using two kits, the Qiamp DNA Investigator Kit for coagulated samples and Qiamp DNA Mini & Blood Mini Kit for non-coagulated samples (QIAgen Systems, Inc., Valencia, CA, USA).	Human	blood	NA	female	Iberian Population in Spain	obese	Overweight and obesity	81	3.32	3.26
EGAN00001617169	EGAN00001617169	Genomic DNA extracted from peripheral white blood cells using two kits, the Qiamp DNA Investigator Kit for coagulated samples and Qiamp DNA Mini & Blood Mini Kit for non-coagulated samples (QIAgen Systems, Inc., Valencia, CA, USA).	Human	blood	NA	female	Iberian Population in Spain	obese	Overweight and obesity	84	2.43	2.32
EGAN00001617170	EGAN00001617170	Genomic DNA extracted from peripheral white blood cells using two kits, the Qiamp DNA Investigator Kit for coagulated samples and Qiamp DNA Mini & Blood Mini Kit for non-coagulated samples (QIAgen Systems, Inc., Valencia, CA, USA).	Human	blood	NA	female	Iberian Population in Spain	overweight	Overweight and obesity	97	3.11	1.54
EGAN00001617171	EGAN00001617171	Genomic DNA extracted from peripheral white blood cells using two kits, the Qiamp DNA Investigator Kit for coagulated samples and Qiamp DNA Mini & Blood Mini Kit for non-coagulated samples (QIAgen Systems, Inc., Valencia, CA, USA).	Human	blood	NA	female	Iberian Population in Spain	overweight	Overweight and obesity	77	1.52	1.27
EGAN00001617172	EGAN00001617172	Genomic DNA extracted from peripheral white blood cells using two kits, the Qiamp DNA Investigator Kit for coagulated samples and Qiamp DNA Mini & Blood Mini Kit for non-coagulated samples (QIAgen Systems, Inc., Valencia, CA, USA).	Human	blood	NA	female	Iberian Population in Spain	overweight	Overweight and obesity	86	0.76	1.43
EGAN00001617173	EGAN00001617173	Genomic DNA extracted from peripheral white blood cells using two kits, the Qiamp DNA Investigator Kit for coagulated samples and Qiamp DNA Mini & Blood Mini Kit for non-coagulated samples (QIAgen Systems, Inc., Valencia, CA, USA).	Human	blood	NA	female	Iberian Population in Spain	overweight	Overweight and obesity	86	2.59	1.19
EGAN00001617174	EGAN00001617174	Genomic DNA extracted from peripheral white blood cells using two kits, the Qiamp DNA Investigator Kit for coagulated samples and Qiamp DNA Mini & Blood Mini Kit for non-coagulated samples (QIAgen Systems, Inc., Valencia, CA, USA).	Human	blood	NA	female	Iberian Population in Spain	obese	Overweight and obesity	86	3.74	2.61
EGAN00001617175	EGAN00001617175	Genomic DNA extracted from peripheral white blood cells using two kits, the Qiamp DNA Investigator Kit for coagulated samples and Qiamp DNA Mini & Blood Mini Kit for non-coagulated samples (QIAgen Systems, Inc., Valencia, CA, USA).	Human	blood	NA	female	Iberian Population in Spain	overweight	Overweight and obesity	93	3.12	0.92
EGAN00001617176	EGAN00001617176	Genomic DNA extracted from peripheral white blood cells using two kits, the Qiamp DNA Investigator Kit for coagulated samples and Qiamp DNA Mini & Blood Mini Kit for non-coagulated samples (QIAgen Systems, Inc., Valencia, CA, USA).	Human	blood	NA	female	Iberian Population in Spain	overweight	Overweight and obesity	84	1.6	0.99
EGAN00001617177	EGAN00001617177	Genomic DNA extracted from peripheral white blood cells using two kits, the Qiamp DNA Investigator Kit for coagulated samples and Qiamp DNA Mini & Blood Mini Kit for non-coagulated samples (QIAgen Systems, Inc., Valencia, CA, USA).	Human	blood	NA	female	Iberian Population in Spain	obese	Overweight and obesity	85	3.48	2.85
EGAN00001617178	EGAN00001617178	Genomic DNA extracted from peripheral white blood cells using two kits, the Qiamp DNA Investigator Kit for coagulated samples and Qiamp DNA Mini & Blood Mini Kit for non-coagulated samples (QIAgen Systems, Inc., Valencia, CA, USA).	Human	blood	NA	female	Iberian Population in Spain	obese	Overweight and obesity	78	2.29	3.35
EGAN00001617179	EGAN00001617179	Genomic DNA extracted from peripheral white blood cells using two kits, the Qiamp DNA Investigator Kit for coagulated samples and Qiamp DNA Mini & Blood Mini Kit for non-coagulated samples (QIAgen Systems, Inc., Valencia, CA, USA).	Human	blood	NA	female	Iberian Population in Spain	obese	Overweight and obesity	81	1.98	2.33
EGAN00001617180	EGAN00001617180	Genomic DNA extracted from peripheral white blood cells using two kits, the Qiamp DNA Investigator Kit for coagulated samples and Qiamp DNA Mini & Blood Mini Kit for non-coagulated samples (QIAgen Systems, Inc., Valencia, CA, USA).	Human	blood	NA	female	Iberian Population in Spain	obese	Overweight and obesity	77	2.3	4.25
EGAN00001617181	EGAN00001617181	Genomic DNA extracted from peripheral white blood cells using two kits, the Qiamp DNA Investigator Kit for coagulated samples and Qiamp DNA Mini & Blood Mini Kit for non-coagulated samples (QIAgen Systems, Inc., Valencia, CA, USA).	Human	blood	NA	female	Iberian Population in Spain	obese	Overweight and obesity	78	3.39	6.13
EGAN00001617182	EGAN00001617182	Genomic DNA extracted from peripheral white blood cells using two kits, the Qiamp DNA Investigator Kit for coagulated samples and Qiamp DNA Mini & Blood Mini Kit for non-coagulated samples (QIAgen Systems, Inc., Valencia, CA, USA).	Human	blood	NA	female	Iberian Population in Spain	obese	Overweight and obesity	82	6.13	6.95
EGAN00001617183	EGAN00001617183	Genomic DNA extracted from peripheral white blood cells using two kits, the Qiamp DNA Investigator Kit for coagulated samples and Qiamp DNA Mini & Blood Mini Kit for non-coagulated samples (QIAgen Systems, Inc., Valencia, CA, USA).	Human	blood	NA	female	Iberian Population in Spain	obese	Overweight and obesity	79	1.97	1.94
EGAN00001617184	EGAN00001617184	Genomic DNA extracted from peripheral white blood cells using two kits, the Qiamp DNA Investigator Kit for coagulated samples and Qiamp DNA Mini & Blood Mini Kit for non-coagulated samples (QIAgen Systems, Inc., Valencia, CA, USA).	Human	blood	NA	female	Iberian Population in Spain	obese	Overweight and obesity	89	2.31	3.43
EGAN00001617185	EGAN00001617185	Genomic DNA extracted from peripheral white blood cells using two kits, the Qiamp DNA Investigator Kit for coagulated samples and Qiamp DNA Mini & Blood Mini Kit for non-coagulated samples (QIAgen Systems, Inc., Valencia, CA, USA).	Human	blood	NA	female	Iberian Population in Spain	obese	Overweight and obesity	89	2.51	3.55
EGAN00001617186	EGAN00001617186	Genomic DNA extracted from peripheral white blood cells using two kits, the Qiamp DNA Investigator Kit for coagulated samples and Qiamp DNA Mini & Blood Mini Kit for non-coagulated samples (QIAgen Systems, Inc., Valencia, CA, USA).	Human	blood	NA	female	Iberian Population in Spain	obese	Overweight and obesity	79	NA	2.67
EGAN00001617187	EGAN00001617187	Genomic DNA extracted from peripheral white blood cells using two kits, the Qiamp DNA Investigator Kit for coagulated samples and Qiamp DNA Mini & Blood Mini Kit for non-coagulated samples (QIAgen Systems, Inc., Valencia, CA, USA).	Human	blood	NA	female	Iberian Population in Spain	overweight	Overweight and obesity	77	1.03	1.35
EGAN00001617188	EGAN00001617188	Genomic DNA extracted from peripheral white blood cells using two kits, the Qiamp DNA Investigator Kit for coagulated samples and Qiamp DNA Mini & Blood Mini Kit for non-coagulated samples (QIAgen Systems, Inc., Valencia, CA, USA).	Human	blood	NA	female	Iberian Population in Spain	overweight	Overweight and obesity	82	0.79	1.96
EGAN00001617189	EGAN00001617189	Genomic DNA extracted from peripheral white blood cells using two kits, the Qiamp DNA Investigator Kit for coagulated samples and Qiamp DNA Mini & Blood Mini Kit for non-coagulated samples (QIAgen Systems, Inc., Valencia, CA, USA).	Human	blood	NA	female	Iberian Population in Spain	overweight	Overweight and obesity	78	0.62	1.43
EGAN00001617190	EGAN00001617190	Genomic DNA extracted from peripheral white blood cells using two kits, the Qiamp DNA Investigator Kit for coagulated samples and Qiamp DNA Mini & Blood Mini Kit for non-coagulated samples (QIAgen Systems, Inc., Valencia, CA, USA).	Human	blood	NA	female	Iberian Population in Spain	overweight	Overweight and obesity	80	0.75	0.95
EGAN00001617191	EGAN00001617191	Genomic DNA extracted from peripheral white blood cells using two kits, the Qiamp DNA Investigator Kit for coagulated samples and Qiamp DNA Mini & Blood Mini Kit for non-coagulated samples (QIAgen Systems, Inc., Valencia, CA, USA).	Human	blood	NA	female	Iberian Population in Spain	overweight	Overweight and obesity	74	0.71	0.79
EGAN00001617192	EGAN00001617192	Genomic DNA extracted from peripheral white blood cells using two kits, the Qiamp DNA Investigator Kit for coagulated samples and Qiamp DNA Mini & Blood Mini Kit for non-coagulated samples (QIAgen Systems, Inc., Valencia, CA, USA).	Human	blood	NA	female	Iberian Population in Spain	overweight	Overweight and obesity	86	1.93	0.88
EGAN00001617193	EGAN00001617193	Genomic DNA extracted from peripheral white blood cells using two kits, the Qiamp DNA Investigator Kit for coagulated samples and Qiamp DNA Mini & Blood Mini Kit for non-coagulated samples (QIAgen Systems, Inc., Valencia, CA, USA).	Human	blood	NA	female	Iberian Population in Spain	overweight	Overweight and obesity	81	0.84	0.49
EGAN00001617194	EGAN00001617194	Genomic DNA extracted from peripheral white blood cells using two kits, the Qiamp DNA Investigator Kit for coagulated samples and Qiamp DNA Mini & Blood Mini Kit for non-coagulated samples (QIAgen Systems, Inc., Valencia, CA, USA).	Human	blood	NA	female	Iberian Population in Spain	overweight	Overweight and obesity	87	1.14	0.8
EGAN00001617195	EGAN00001617195	Genomic DNA extracted from peripheral white blood cells using two kits, the Qiamp DNA Investigator Kit for coagulated samples and Qiamp DNA Mini & Blood Mini Kit for non-coagulated samples (QIAgen Systems, Inc., Valencia, CA, USA).	Human	blood	NA	female	Iberian Population in Spain	overweight	Overweight and obesity	95	1.83	0.68
EGAN00001617196	EGAN00001617196	Genomic DNA extracted from peripheral white blood cells using two kits, the Qiamp DNA Investigator Kit for coagulated samples and Qiamp DNA Mini & Blood Mini Kit for non-coagulated samples (QIAgen Systems, Inc., Valencia, CA, USA).	Human	blood	NA	female	Iberian Population in Spain	overweight	Overweight and obesity	88	2.15	1.28
EGAN00001617197	EGAN00001617197	Genomic DNA extracted from peripheral white blood cells using two kits, the Qiamp DNA Investigator Kit for coagulated samples and Qiamp DNA Mini & Blood Mini Kit for non-coagulated samples (QIAgen Systems, Inc., Valencia, CA, USA).	Human	blood	NA	female	Iberian Population in Spain	overweight	Overweight and obesity	82	0.67	1.67
EGAN00001617198	EGAN00001617198	Genomic DNA extracted from peripheral white blood cells using two kits, the Qiamp DNA Investigator Kit for coagulated samples and Qiamp DNA Mini & Blood Mini Kit for non-coagulated samples (QIAgen Systems, Inc., Valencia, CA, USA).	Human	blood	NA	female	Iberian Population in Spain	overweight	Overweight and obesity	96	3.18	0.95
EGAN00001617199	EGAN00001617199	Genomic DNA extracted from peripheral white blood cells using two kits, the Qiamp DNA Investigator Kit for coagulated samples and Qiamp DNA Mini & Blood Mini Kit for non-coagulated samples (QIAgen Systems, Inc., Valencia, CA, USA).	Human	blood	NA	female	Iberian Population in Spain	overweight	Overweight and obesity	104	3.85	1.06
EGAN00001617200	EGAN00001617200	Genomic DNA extracted from peripheral white blood cells using two kits, the Qiamp DNA Investigator Kit for coagulated samples and Qiamp DNA Mini & Blood Mini Kit for non-coagulated samples (QIAgen Systems, Inc., Valencia, CA, USA).	Human	blood	NA	female	Iberian Population in Spain	obese	Overweight and obesity	83	2.71	1.93
EGAN00001617201	EGAN00001617201	Genomic DNA extracted from peripheral white blood cells using two kits, the Qiamp DNA Investigator Kit for coagulated samples and Qiamp DNA Mini & Blood Mini Kit for non-coagulated samples (QIAgen Systems, Inc., Valencia, CA, USA).	Human	blood	NA	female	Iberian Population in Spain	overweight	Overweight and obesity	95	0.47	1.28
EGAN00001617202	EGAN00001617202	Genomic DNA extracted from peripheral white blood cells using two kits, the Qiamp DNA Investigator Kit for coagulated samples and Qiamp DNA Mini & Blood Mini Kit for non-coagulated samples (QIAgen Systems, Inc., Valencia, CA, USA).	Human	blood	NA	female	Iberian Population in Spain	overweight	Overweight and obesity	90	2.78	1.33
EGAN00001617203	EGAN00001617203	Genomic DNA extracted from peripheral white blood cells using two kits, the Qiamp DNA Investigator Kit for coagulated samples and Qiamp DNA Mini & Blood Mini Kit for non-coagulated samples (QIAgen Systems, Inc., Valencia, CA, USA).	Human	blood	NA	female	Iberian Population in Spain	obese	Overweight and obesity	98	4.99	4.01
EGAN00001617204	EGAN00001617204	Genomic DNA extracted from peripheral white blood cells using two kits, the Qiamp DNA Investigator Kit for coagulated samples and Qiamp DNA Mini & Blood Mini Kit for non-coagulated samples (QIAgen Systems, Inc., Valencia, CA, USA).	Human	blood	NA	female	Iberian Population in Spain	overweight	Overweight and obesity	84	2.14	1.44
EGAN00001617205	EGAN00001617205	Genomic DNA extracted from peripheral white blood cells using two kits, the Qiamp DNA Investigator Kit for coagulated samples and Qiamp DNA Mini & Blood Mini Kit for non-coagulated samples (QIAgen Systems, Inc., Valencia, CA, USA).	Human	blood	NA	female	Iberian Population in Spain	obese	Overweight and obesity	83	2.07	2.78
EGAN00001617206	EGAN00001617206	Genomic DNA extracted from peripheral white blood cells using two kits, the Qiamp DNA Investigator Kit for coagulated samples and Qiamp DNA Mini & Blood Mini Kit for non-coagulated samples (QIAgen Systems, Inc., Valencia, CA, USA).	Human	blood	NA	female	Iberian Population in Spain	obese	Overweight and obesity	83	1.39	1.91
EGAN00001617207	EGAN00001617207	Genomic DNA extracted from peripheral white blood cells using two kits, the Qiamp DNA Investigator Kit for coagulated samples and Qiamp DNA Mini & Blood Mini Kit for non-coagulated samples (QIAgen Systems, Inc., Valencia, CA, USA).	Human	blood	NA	female	Iberian Population in Spain	overweight	Overweight and obesity	90	4.04	1.63
EGAN00001617208	EGAN00001617208	Genomic DNA extracted from peripheral white blood cells using two kits, the Qiamp DNA Investigator Kit for coagulated samples and Qiamp DNA Mini & Blood Mini Kit for non-coagulated samples (QIAgen Systems, Inc., Valencia, CA, USA).	Human	blood	NA	female	Iberian Population in Spain	obese	Overweight and obesity	86	3.57	1.83
EGAN00001617209	EGAN00001617209	Genomic DNA extracted from peripheral white blood cells using two kits, the Qiamp DNA Investigator Kit for coagulated samples and Qiamp DNA Mini & Blood Mini Kit for non-coagulated samples (QIAgen Systems, Inc., Valencia, CA, USA).	Human	blood	NA	female	Iberian Population in Spain	obese	Overweight and obesity	83	3.75	1.99
EGAN00001617210	EGAN00001617210	Genomic DNA extracted from peripheral white blood cells using two kits, the Qiamp DNA Investigator Kit for coagulated samples and Qiamp DNA Mini & Blood Mini Kit for non-coagulated samples (QIAgen Systems, Inc., Valencia, CA, USA).	Human	blood	NA	female	Iberian Population in Spain	overweight	Overweight and obesity	87	0.8	0.64
EGAN00001617211	EGAN00001617211	Genomic DNA extracted from peripheral white blood cells using two kits, the Qiamp DNA Investigator Kit for coagulated samples and Qiamp DNA Mini & Blood Mini Kit for non-coagulated samples (QIAgen Systems, Inc., Valencia, CA, USA).	Human	blood	NA	female	Iberian Population in Spain	obese	Overweight and obesity	79	2.98	2.86
EGAN00001617212	EGAN00001617212	Genomic DNA extracted from peripheral white blood cells using two kits, the Qiamp DNA Investigator Kit for coagulated samples and Qiamp DNA Mini & Blood Mini Kit for non-coagulated samples (QIAgen Systems, Inc., Valencia, CA, USA).	Human	blood	NA	female	Iberian Population in Spain	overweight	Overweight and obesity	86	2.34	1.82
EGAN00001617213	EGAN00001617213	Genomic DNA extracted from peripheral white blood cells using two kits, the Qiamp DNA Investigator Kit for coagulated samples and Qiamp DNA Mini & Blood Mini Kit for non-coagulated samples (QIAgen Systems, Inc., Valencia, CA, USA).	Human	blood	NA	female	Iberian Population in Spain	overweight	Overweight and obesity	91	1.98	0.64
EGAN00001617214	EGAN00001617214	Genomic DNA extracted from peripheral white blood cells using two kits, the Qiamp DNA Investigator Kit for coagulated samples and Qiamp DNA Mini & Blood Mini Kit for non-coagulated samples (QIAgen Systems, Inc., Valencia, CA, USA).	Human	blood	NA	female	Iberian Population in Spain	overweight	Overweight and obesity	83	1.93	1.67
EGAN00001617215	EGAN00001617215	Genomic DNA extracted from peripheral white blood cells using two kits, the Qiamp DNA Investigator Kit for coagulated samples and Qiamp DNA Mini & Blood Mini Kit for non-coagulated samples (QIAgen Systems, Inc., Valencia, CA, USA).	Human	blood	NA	female	Iberian Population in Spain	overweight	Overweight and obesity	83	1.05	1.27
EGAN00001617216	EGAN00001617216	Genomic DNA extracted from peripheral white blood cells using two kits, the Qiamp DNA Investigator Kit for coagulated samples and Qiamp DNA Mini & Blood Mini Kit for non-coagulated samples (QIAgen Systems, Inc., Valencia, CA, USA).	Human	blood	NA	female	Iberian Population in Spain	overweight	Overweight and obesity	94	1.44	1.39
EGAN00001617217	EGAN00001617217	Genomic DNA extracted from peripheral white blood cells using two kits, the Qiamp DNA Investigator Kit for coagulated samples and Qiamp DNA Mini & Blood Mini Kit for non-coagulated samples (QIAgen Systems, Inc., Valencia, CA, USA).	Human	blood	NA	female	Iberian Population in Spain	obese	Overweight and obesity	89	2.15	3.89
EGAN00001617218	EGAN00001617218	Genomic DNA extracted from peripheral white blood cells using two kits, the Qiamp DNA Investigator Kit for coagulated samples and Qiamp DNA Mini & Blood Mini Kit for non-coagulated samples (QIAgen Systems, Inc., Valencia, CA, USA).	Human	blood	NA	female	Iberian Population in Spain	obese	Overweight and obesity	85	2.32	2.15
EGAN00001617219	EGAN00001617219	Genomic DNA extracted from peripheral white blood cells using two kits, the Qiamp DNA Investigator Kit for coagulated samples and Qiamp DNA Mini & Blood Mini Kit for non-coagulated samples (QIAgen Systems, Inc., Valencia, CA, USA).	Human	blood	NA	female	Iberian Population in Spain	overweight	Overweight and obesity	87	1.07	1.09
EGAN00001617220	EGAN00001617220	Genomic DNA extracted from peripheral white blood cells using two kits, the Qiamp DNA Investigator Kit for coagulated samples and Qiamp DNA Mini & Blood Mini Kit for non-coagulated samples (QIAgen Systems, Inc., Valencia, CA, USA).	Human	blood	NA	female	Iberian Population in Spain	overweight	Overweight and obesity	85	0.84	0.63
EGAN00001617221	EGAN00001617221	Genomic DNA extracted from peripheral white blood cells using two kits, the Qiamp DNA Investigator Kit for coagulated samples and Qiamp DNA Mini & Blood Mini Kit for non-coagulated samples (QIAgen Systems, Inc., Valencia, CA, USA).	Human	blood	NA	female	Iberian Population in Spain	obese	Overweight and obesity	85	1.28	1.66
EGAN00001617222	EGAN00001617222	Genomic DNA extracted from peripheral white blood cells using two kits, the Qiamp DNA Investigator Kit for coagulated samples and Qiamp DNA Mini & Blood Mini Kit for non-coagulated samples (QIAgen Systems, Inc., Valencia, CA, USA).	Human	blood	NA	female	Iberian Population in Spain	overweight	Overweight and obesity	99	1.33	1.21
EGAN00001617223	EGAN00001617223	Genomic DNA extracted from peripheral white blood cells using two kits, the Qiamp DNA Investigator Kit for coagulated samples and Qiamp DNA Mini & Blood Mini Kit for non-coagulated samples (QIAgen Systems, Inc., Valencia, CA, USA).	Human	blood	NA	female	Iberian Population in Spain	obese	Overweight and obesity	87	1.42	2.81
EGAN00001617224	EGAN00001617224	Genomic DNA extracted from peripheral white blood cells using two kits, the Qiamp DNA Investigator Kit for coagulated samples and Qiamp DNA Mini & Blood Mini Kit for non-coagulated samples (QIAgen Systems, Inc., Valencia, CA, USA).	Human	blood	NA	female	Iberian Population in Spain	overweight	Overweight and obesity	91	2.94	1.51
EGAN00001617225	EGAN00001617225	Genomic DNA extracted from peripheral white blood cells using two kits, the Qiamp DNA Investigator Kit for coagulated samples and Qiamp DNA Mini & Blood Mini Kit for non-coagulated samples (QIAgen Systems, Inc., Valencia, CA, USA).	Human	blood	NA	female	Iberian Population in Spain	overweight	Overweight and obesity	97	5.53	1.24
EGAN00001617226	EGAN00001617226	Genomic DNA extracted from peripheral white blood cells using two kits, the Qiamp DNA Investigator Kit for coagulated samples and Qiamp DNA Mini & Blood Mini Kit for non-coagulated samples (QIAgen Systems, Inc., Valencia, CA, USA).	Human	blood	NA	female	Iberian Population in Spain	overweight	Overweight and obesity	97	2.13	1.13
EGAN00001617227	EGAN00001617227	Genomic DNA extracted from peripheral white blood cells using two kits, the Qiamp DNA Investigator Kit for coagulated samples and Qiamp DNA Mini & Blood Mini Kit for non-coagulated samples (QIAgen Systems, Inc., Valencia, CA, USA).	Human	blood	NA	female	Iberian Population in Spain	overweight	Overweight and obesity	80	1.36	1.42
EGAN00001617228	EGAN00001617228	Genomic DNA extracted from peripheral white blood cells using two kits, the Qiamp DNA Investigator Kit for coagulated samples and Qiamp DNA Mini & Blood Mini Kit for non-coagulated samples (QIAgen Systems, Inc., Valencia, CA, USA).	Human	blood	NA	female	Iberian Population in Spain	obese	Overweight and obesity	77	0.57	2.32
EGAN00001617229	EGAN00001617229	Genomic DNA extracted from peripheral white blood cells using two kits, the Qiamp DNA Investigator Kit for coagulated samples and Qiamp DNA Mini & Blood Mini Kit for non-coagulated samples (QIAgen Systems, Inc., Valencia, CA, USA).	Human	blood	NA	female	Iberian Population in Spain	overweight	Overweight and obesity	86	1.42	1.44
EGAN00001617230	EGAN00001617230	Genomic DNA extracted from peripheral white blood cells using two kits, the Qiamp DNA Investigator Kit for coagulated samples and Qiamp DNA Mini & Blood Mini Kit for non-coagulated samples (QIAgen Systems, Inc., Valencia, CA, USA).	Human	blood	NA	female	Iberian Population in Spain	overweight	Overweight and obesity	84	1.41	1.6
EGAN00001617231	EGAN00001617231	Genomic DNA extracted from peripheral white blood cells using two kits, the Qiamp DNA Investigator Kit for coagulated samples and Qiamp DNA Mini & Blood Mini Kit for non-coagulated samples (QIAgen Systems, Inc., Valencia, CA, USA).	Human	blood	NA	female	Iberian Population in Spain	overweight	Overweight and obesity	75	0.37	2.23
EGAN00001617232	EGAN00001617232	Genomic DNA extracted from peripheral white blood cells using two kits, the Qiamp DNA Investigator Kit for coagulated samples and Qiamp DNA Mini & Blood Mini Kit for non-coagulated samples (QIAgen Systems, Inc., Valencia, CA, USA).	Human	blood	NA	female	Iberian Population in Spain	overweight	Overweight and obesity	96	5.14	1.76
EGAN00001617233	EGAN00001617233	Genomic DNA extracted from peripheral white blood cells using two kits, the Qiamp DNA Investigator Kit for coagulated samples and Qiamp DNA Mini & Blood Mini Kit for non-coagulated samples (QIAgen Systems, Inc., Valencia, CA, USA).	Human	blood	NA	female	Iberian Population in Spain	overweight	Overweight and obesity	92	1.95	1
EGAN00001617234	EGAN00001617234	Genomic DNA extracted from peripheral white blood cells using two kits, the Qiamp DNA Investigator Kit for coagulated samples and Qiamp DNA Mini & Blood Mini Kit for non-coagulated samples (QIAgen Systems, Inc., Valencia, CA, USA).	Human	blood	NA	female	Iberian Population in Spain	obese	Overweight and obesity	84	1	1.52
EGAN00001617235	EGAN00001617235	Genomic DNA extracted from peripheral white blood cells using two kits, the Qiamp DNA Investigator Kit for coagulated samples and Qiamp DNA Mini & Blood Mini Kit for non-coagulated samples (QIAgen Systems, Inc., Valencia, CA, USA).	Human	blood	NA	female	Iberian Population in Spain	obese	Overweight and obesity	93	3.42	2.91
EGAN00001617236	EGAN00001617236	Genomic DNA extracted from peripheral white blood cells using two kits, the Qiamp DNA Investigator Kit for coagulated samples and Qiamp DNA Mini & Blood Mini Kit for non-coagulated samples (QIAgen Systems, Inc., Valencia, CA, USA).	Human	blood	NA	female	Iberian Population in Spain	obese	Overweight and obesity	100	4.32	2.14
EGAN00001617237	EGAN00001617237	Genomic DNA extracted from peripheral white blood cells using two kits, the Qiamp DNA Investigator Kit for coagulated samples and Qiamp DNA Mini & Blood Mini Kit for non-coagulated samples (QIAgen Systems, Inc., Valencia, CA, USA).	Human	blood	NA	female	Iberian Population in Spain	obese	Overweight and obesity	100	10.52	4.61
EGAN00001617238	EGAN00001617238	Genomic DNA extracted from peripheral white blood cells using two kits, the Qiamp DNA Investigator Kit for coagulated samples and Qiamp DNA Mini & Blood Mini Kit for non-coagulated samples (QIAgen Systems, Inc., Valencia, CA, USA).	Human	blood	NA	female	Iberian Population in Spain	obese	Overweight and obesity	85	3.53	3.63
EGAN00001617239	EGAN00001617239	Genomic DNA extracted from peripheral white blood cells using two kits, the Qiamp DNA Investigator Kit for coagulated samples and Qiamp DNA Mini & Blood Mini Kit for non-coagulated samples (QIAgen Systems, Inc., Valencia, CA, USA).	Human	blood	NA	female	Iberian Population in Spain	obese	Overweight and obesity	80	13.53	2.8
EGAN00001617240	EGAN00001617240	Genomic DNA extracted from peripheral white blood cells using two kits, the Qiamp DNA Investigator Kit for coagulated samples and Qiamp DNA Mini & Blood Mini Kit for non-coagulated samples (QIAgen Systems, Inc., Valencia, CA, USA).	Human	blood	NA	female	Iberian Population in Spain	obese	Overweight and obesity	81	4.28	2.68
EGAN00001617241	EGAN00001617241	Genomic DNA extracted from peripheral white blood cells using two kits, the Qiamp DNA Investigator Kit for coagulated samples and Qiamp DNA Mini & Blood Mini Kit for non-coagulated samples (QIAgen Systems, Inc., Valencia, CA, USA).	Human	blood	NA	female	Iberian Population in Spain	overweight	Overweight and obesity	81	2.64	2.21
EGAN00001617242	EGAN00001617242	Genomic DNA extracted from peripheral white blood cells using two kits, the Qiamp DNA Investigator Kit for coagulated samples and Qiamp DNA Mini & Blood Mini Kit for non-coagulated samples (QIAgen Systems, Inc., Valencia, CA, USA).	Human	blood	NA	female	Iberian Population in Spain	obese	Overweight and obesity	75	3.37	2.25
EGAN00001617243	EGAN00001617243	Genomic DNA extracted from peripheral white blood cells using two kits, the Qiamp DNA Investigator Kit for coagulated samples and Qiamp DNA Mini & Blood Mini Kit for non-coagulated samples (QIAgen Systems, Inc., Valencia, CA, USA).	Human	blood	NA	female	Iberian Population in Spain	obese	Overweight and obesity	96	2.99	5.47
EGAN00001617244	EGAN00001617244	Genomic DNA extracted from peripheral white blood cells using two kits, the Qiamp DNA Investigator Kit for coagulated samples and Qiamp DNA Mini & Blood Mini Kit for non-coagulated samples (QIAgen Systems, Inc., Valencia, CA, USA).	Human	blood	NA	female	Iberian Population in Spain	obese	Overweight and obesity	87	2.02	2.56
EGAN00001617245	EGAN00001617245	Genomic DNA extracted from peripheral white blood cells using two kits, the Qiamp DNA Investigator Kit for coagulated samples and Qiamp DNA Mini & Blood Mini Kit for non-coagulated samples (QIAgen Systems, Inc., Valencia, CA, USA).	Human	blood	NA	female	Iberian Population in Spain	overweight	Overweight and obesity	90	1.11	1.17
EGAN00001617246	EGAN00001617246	Genomic DNA extracted from peripheral white blood cells using two kits, the Qiamp DNA Investigator Kit for coagulated samples and Qiamp DNA Mini & Blood Mini Kit for non-coagulated samples (QIAgen Systems, Inc., Valencia, CA, USA).	Human	blood	NA	female	Iberian Population in Spain	overweight	Overweight and obesity	87	2.19	1.8
EGAN00001617247	EGAN00001617247	Genomic DNA extracted from peripheral white blood cells using two kits, the Qiamp DNA Investigator Kit for coagulated samples and Qiamp DNA Mini & Blood Mini Kit for non-coagulated samples (QIAgen Systems, Inc., Valencia, CA, USA).	Human	blood	NA	female	Iberian Population in Spain	obese	Overweight and obesity	84	3.9	3.89
EGAN00001617248	EGAN00001617248	Genomic DNA extracted from peripheral white blood cells using two kits, the Qiamp DNA Investigator Kit for coagulated samples and Qiamp DNA Mini & Blood Mini Kit for non-coagulated samples (QIAgen Systems, Inc., Valencia, CA, USA).	Human	blood	NA	female	Iberian Population in Spain	overweight	Overweight and obesity	91	1.8	1.29
EGAN00001617249	EGAN00001617249	Genomic DNA extracted from peripheral white blood cells using two kits, the Qiamp DNA Investigator Kit for coagulated samples and Qiamp DNA Mini & Blood Mini Kit for non-coagulated samples (QIAgen Systems, Inc., Valencia, CA, USA).	Human	blood	NA	female	Iberian Population in Spain	obese	Overweight and obesity	85	2.58	3.2
EGAN00001617250	EGAN00001617250	Genomic DNA extracted from peripheral white blood cells using two kits, the Qiamp DNA Investigator Kit for coagulated samples and Qiamp DNA Mini & Blood Mini Kit for non-coagulated samples (QIAgen Systems, Inc., Valencia, CA, USA).	Human	blood	NA	female	Iberian Population in Spain	obese	Overweight and obesity	74	1.63	3.94
EGAN00001617251	EGAN00001617251	Genomic DNA extracted from peripheral white blood cells using two kits, the Qiamp DNA Investigator Kit for coagulated samples and Qiamp DNA Mini & Blood Mini Kit for non-coagulated samples (QIAgen Systems, Inc., Valencia, CA, USA).	Human	blood	NA	female	Iberian Population in Spain	obese	Overweight and obesity	83	1.62	3.58
EGAN00001617252	EGAN00001617252	Genomic DNA extracted from peripheral white blood cells using two kits, the Qiamp DNA Investigator Kit for coagulated samples and Qiamp DNA Mini & Blood Mini Kit for non-coagulated samples (QIAgen Systems, Inc., Valencia, CA, USA).	Human	blood	NA	female	Iberian Population in Spain	obese	Overweight and obesity	82	4.35	4.68
EGAN00001617253	EGAN00001617253	Genomic DNA extracted from peripheral white blood cells using two kits, the Qiamp DNA Investigator Kit for coagulated samples and Qiamp DNA Mini & Blood Mini Kit for non-coagulated samples (QIAgen Systems, Inc., Valencia, CA, USA).	Human	blood	NA	female	Iberian Population in Spain	obese	Overweight and obesity	82	1.38	2.26
EGAN00001617254	EGAN00001617254	Genomic DNA extracted from peripheral white blood cells using two kits, the Qiamp DNA Investigator Kit for coagulated samples and Qiamp DNA Mini & Blood Mini Kit for non-coagulated samples (QIAgen Systems, Inc., Valencia, CA, USA).	Human	blood	NA	female	Iberian Population in Spain	obese	Overweight and obesity	81	2.84	3.95
EGAN00001617255	EGAN00001617255	Genomic DNA extracted from peripheral white blood cells using two kits, the Qiamp DNA Investigator Kit for coagulated samples and Qiamp DNA Mini & Blood Mini Kit for non-coagulated samples (QIAgen Systems, Inc., Valencia, CA, USA).	Human	blood	NA	female	Iberian Population in Spain	obese	Overweight and obesity	59	0.61	3.15
EGAN00001617256	EGAN00001617256	Genomic DNA extracted from peripheral white blood cells using two kits, the Qiamp DNA Investigator Kit for coagulated samples and Qiamp DNA Mini & Blood Mini Kit for non-coagulated samples (QIAgen Systems, Inc., Valencia, CA, USA).	Human	blood	NA	female	Iberian Population in Spain	obese	Overweight and obesity	85	1.91	2.96
EGAN00001617257	EGAN00001617257	Genomic DNA extracted from peripheral white blood cells using two kits, the Qiamp DNA Investigator Kit for coagulated samples and Qiamp DNA Mini & Blood Mini Kit for non-coagulated samples (QIAgen Systems, Inc., Valencia, CA, USA).	Human	blood	NA	female	Iberian Population in Spain	obese	Overweight and obesity	87	3.27	3.36
EGAN00001617258	EGAN00001617258	Genomic DNA extracted from peripheral white blood cells using two kits, the Qiamp DNA Investigator Kit for coagulated samples and Qiamp DNA Mini & Blood Mini Kit for non-coagulated samples (QIAgen Systems, Inc., Valencia, CA, USA).	Human	blood	NA	female	Iberian Population in Spain	obese	Overweight and obesity	70	3.2	6.6
EGAN00001617259	EGAN00001617259	Genomic DNA extracted from peripheral white blood cells using two kits, the Qiamp DNA Investigator Kit for coagulated samples and Qiamp DNA Mini & Blood Mini Kit for non-coagulated samples (QIAgen Systems, Inc., Valencia, CA, USA).	Human	blood	NA	female	Iberian Population in Spain	overweight	Overweight and obesity	80	0.79	0.95
EGAN00001617260	EGAN00001617260	Genomic DNA extracted from peripheral white blood cells using two kits, the Qiamp DNA Investigator Kit for coagulated samples and Qiamp DNA Mini & Blood Mini Kit for non-coagulated samples (QIAgen Systems, Inc., Valencia, CA, USA).	Human	blood	NA	female	Iberian Population in Spain	obese	Overweight and obesity	76	1.24	6.45
EGAN00001617261	EGAN00001617261	Genomic DNA extracted from peripheral white blood cells using two kits, the Qiamp DNA Investigator Kit for coagulated samples and Qiamp DNA Mini & Blood Mini Kit for non-coagulated samples (QIAgen Systems, Inc., Valencia, CA, USA).	Human	blood	NA	female	Iberian Population in Spain	obese	Overweight and obesity	79	2.11	4.21
EGAN00001617262	EGAN00001617262	Genomic DNA extracted from peripheral white blood cells using two kits, the Qiamp DNA Investigator Kit for coagulated samples and Qiamp DNA Mini & Blood Mini Kit for non-coagulated samples (QIAgen Systems, Inc., Valencia, CA, USA).	Human	blood	NA	female	Iberian Population in Spain	obese	Overweight and obesity	86	1.59	4.64
EGAN00001617263	EGAN00001617263	Genomic DNA extracted from peripheral white blood cells using two kits, the Qiamp DNA Investigator Kit for coagulated samples and Qiamp DNA Mini & Blood Mini Kit for non-coagulated samples (QIAgen Systems, Inc., Valencia, CA, USA).	Human	blood	NA	female	Iberian Population in Spain	obese	Overweight and obesity	82	3.08	3.25
EGAN00001617264	EGAN00001617264	Genomic DNA extracted from peripheral white blood cells using two kits, the Qiamp DNA Investigator Kit for coagulated samples and Qiamp DNA Mini & Blood Mini Kit for non-coagulated samples (QIAgen Systems, Inc., Valencia, CA, USA).	Human	blood	NA	female	Iberian Population in Spain	obese	Overweight and obesity	80	0.85	1.74
EGAN00001617265	EGAN00001617265	Genomic DNA extracted from peripheral white blood cells using two kits, the Qiamp DNA Investigator Kit for coagulated samples and Qiamp DNA Mini & Blood Mini Kit for non-coagulated samples (QIAgen Systems, Inc., Valencia, CA, USA).	Human	blood	NA	female	Iberian Population in Spain	overweight	Overweight and obesity	94	1.12	1.36
EGAN00001617266	EGAN00001617266	Genomic DNA extracted from peripheral white blood cells using two kits, the Qiamp DNA Investigator Kit for coagulated samples and Qiamp DNA Mini & Blood Mini Kit for non-coagulated samples (QIAgen Systems, Inc., Valencia, CA, USA).	Human	blood	NA	female	Iberian Population in Spain	overweight	Overweight and obesity	84	0.99	0.91
EGAN00001617267	EGAN00001617267	Genomic DNA extracted from peripheral white blood cells using two kits, the Qiamp DNA Investigator Kit for coagulated samples and Qiamp DNA Mini & Blood Mini Kit for non-coagulated samples (QIAgen Systems, Inc., Valencia, CA, USA).	Human	blood	NA	female	Iberian Population in Spain	overweight	Overweight and obesity	84	0.81	0.61
EGAN00001617268	EGAN00001617268	Genomic DNA extracted from peripheral white blood cells using two kits, the Qiamp DNA Investigator Kit for coagulated samples and Qiamp DNA Mini & Blood Mini Kit for non-coagulated samples (QIAgen Systems, Inc., Valencia, CA, USA).	Human	blood	NA	female	Iberian Population in Spain	overweight	Overweight and obesity	91	2.09	0.54
EGAN00001617269	EGAN00001617269	Genomic DNA extracted from peripheral white blood cells using two kits, the Qiamp DNA Investigator Kit for coagulated samples and Qiamp DNA Mini & Blood Mini Kit for non-coagulated samples (QIAgen Systems, Inc., Valencia, CA, USA).	Human	blood	NA	female	Iberian Population in Spain	obese	Overweight and obesity	87	1.22	2.48
EGAN00001617270	EGAN00001617270	Genomic DNA extracted from peripheral white blood cells using two kits, the Qiamp DNA Investigator Kit for coagulated samples and Qiamp DNA Mini & Blood Mini Kit for non-coagulated samples (QIAgen Systems, Inc., Valencia, CA, USA).	Human	blood	NA	female	Iberian Population in Spain	obese	Overweight and obesity	79	1.5	4.09
EGAN00001617271	EGAN00001617271	Genomic DNA extracted from peripheral white blood cells using two kits, the Qiamp DNA Investigator Kit for coagulated samples and Qiamp DNA Mini & Blood Mini Kit for non-coagulated samples (QIAgen Systems, Inc., Valencia, CA, USA).	Human	blood	NA	female	Iberian Population in Spain	obese	Overweight and obesity	82	2.07	3.92
EGAN00001617272	EGAN00001617272	Genomic DNA extracted from peripheral white blood cells using two kits, the Qiamp DNA Investigator Kit for coagulated samples and Qiamp DNA Mini & Blood Mini Kit for non-coagulated samples (QIAgen Systems, Inc., Valencia, CA, USA).	Human	blood	NA	female	Iberian Population in Spain	obese	Overweight and obesity	100	NA	2.55
EGAN00001617273	EGAN00001617273	Genomic DNA extracted from peripheral white blood cells using two kits, the Qiamp DNA Investigator Kit for coagulated samples and Qiamp DNA Mini & Blood Mini Kit for non-coagulated samples (QIAgen Systems, Inc., Valencia, CA, USA).	Human	blood	NA	female	Iberian Population in Spain	obese	Overweight and obesity	77	1.24	1.92
EGAN00001617274	EGAN00001617274	Genomic DNA extracted from peripheral white blood cells using two kits, the Qiamp DNA Investigator Kit for coagulated samples and Qiamp DNA Mini & Blood Mini Kit for non-coagulated samples (QIAgen Systems, Inc., Valencia, CA, USA).	Human	blood	NA	female	Iberian Population in Spain	obese	Overweight and obesity	79	2.19	2.91
EGAN00001617275	EGAN00001617275	Genomic DNA extracted from peripheral white blood cells using two kits, the Qiamp DNA Investigator Kit for coagulated samples and Qiamp DNA Mini & Blood Mini Kit for non-coagulated samples (QIAgen Systems, Inc., Valencia, CA, USA).	Human	blood	NA	female	Iberian Population in Spain	obese	Overweight and obesity	82	4.09	2.8
EGAN00001617276	EGAN00001617276	Genomic DNA extracted from peripheral white blood cells using two kits, the Qiamp DNA Investigator Kit for coagulated samples and Qiamp DNA Mini & Blood Mini Kit for non-coagulated samples (QIAgen Systems, Inc., Valencia, CA, USA).	Human	blood	NA	female	Iberian Population in Spain	obese	Overweight and obesity	95	5.49	5.49
EGAN00001617277	EGAN00001617277	Genomic DNA extracted from peripheral white blood cells using two kits, the Qiamp DNA Investigator Kit for coagulated samples and Qiamp DNA Mini & Blood Mini Kit for non-coagulated samples (QIAgen Systems, Inc., Valencia, CA, USA).	Human	blood	NA	female	Iberian Population in Spain	obese	Overweight and obesity	88	2.85	3.1
EGAN00001617278	EGAN00001617278	Genomic DNA extracted from peripheral white blood cells using two kits, the Qiamp DNA Investigator Kit for coagulated samples and Qiamp DNA Mini & Blood Mini Kit for non-coagulated samples (QIAgen Systems, Inc., Valencia, CA, USA).	Human	blood	NA	female	Iberian Population in Spain	obese	Overweight and obesity	89	2.09	5.3
EGAN00001617279	EGAN00001617279	Genomic DNA extracted from peripheral white blood cells using two kits, the Qiamp DNA Investigator Kit for coagulated samples and Qiamp DNA Mini & Blood Mini Kit for non-coagulated samples (QIAgen Systems, Inc., Valencia, CA, USA).	Human	blood	NA	female	Iberian Population in Spain	obese	Overweight and obesity	80	1.46	4.05
EGAN00001617280	EGAN00001617280	Genomic DNA extracted from peripheral white blood cells using two kits, the Qiamp DNA Investigator Kit for coagulated samples and Qiamp DNA Mini & Blood Mini Kit for non-coagulated samples (QIAgen Systems, Inc., Valencia, CA, USA).	Human	blood	NA	female	Iberian Population in Spain	obese	Overweight and obesity	81	0.82	3.03
EGAN00001617281	EGAN00001617281	Genomic DNA extracted from peripheral white blood cells using two kits, the Qiamp DNA Investigator Kit for coagulated samples and Qiamp DNA Mini & Blood Mini Kit for non-coagulated samples (QIAgen Systems, Inc., Valencia, CA, USA).	Human	blood	NA	female	Iberian Population in Spain	obese	Overweight and obesity	80	0.57	2.58
EGAN00001617282	EGAN00001617282	Genomic DNA extracted from peripheral white blood cells using two kits, the Qiamp DNA Investigator Kit for coagulated samples and Qiamp DNA Mini & Blood Mini Kit for non-coagulated samples (QIAgen Systems, Inc., Valencia, CA, USA).	Human	blood	NA	female	Iberian Population in Spain	obese	Overweight and obesity	91	4.83	4.67
EGAN00001617283	EGAN00001617283	Genomic DNA extracted from peripheral white blood cells using two kits, the Qiamp DNA Investigator Kit for coagulated samples and Qiamp DNA Mini & Blood Mini Kit for non-coagulated samples (QIAgen Systems, Inc., Valencia, CA, USA).	Human	blood	NA	female	Iberian Population in Spain	overweight	Overweight and obesity	85	1.64	1.77
EGAN00001617284	EGAN00001617284	Genomic DNA extracted from peripheral white blood cells using two kits, the Qiamp DNA Investigator Kit for coagulated samples and Qiamp DNA Mini & Blood Mini Kit for non-coagulated samples (QIAgen Systems, Inc., Valencia, CA, USA).	Human	blood	NA	female	Iberian Population in Spain	obese	Overweight and obesity	87	1.74	2.12
EGAN00001617285	EGAN00001617285	Genomic DNA extracted from peripheral white blood cells using two kits, the Qiamp DNA Investigator Kit for coagulated samples and Qiamp DNA Mini & Blood Mini Kit for non-coagulated samples (QIAgen Systems, Inc., Valencia, CA, USA).	Human	blood	NA	female	Iberian Population in Spain	obese	Overweight and obesity	81	2	3.25
EGAN00001617286	EGAN00001617286	Genomic DNA extracted from peripheral white blood cells using two kits, the Qiamp DNA Investigator Kit for coagulated samples and Qiamp DNA Mini & Blood Mini Kit for non-coagulated samples (QIAgen Systems, Inc., Valencia, CA, USA).	Human	blood	NA	female	Iberian Population in Spain	obese	Overweight and obesity	79	2.42	3.54
EGAN00001617287	EGAN00001617287	Genomic DNA extracted from peripheral white blood cells using two kits, the Qiamp DNA Investigator Kit for coagulated samples and Qiamp DNA Mini & Blood Mini Kit for non-coagulated samples (QIAgen Systems, Inc., Valencia, CA, USA).	Human	blood	NA	female	Iberian Population in Spain	obese	Overweight and obesity	78	1.29	2.92
EGAN00001617288	EGAN00001617288	Genomic DNA extracted from peripheral white blood cells using two kits, the Qiamp DNA Investigator Kit for coagulated samples and Qiamp DNA Mini & Blood Mini Kit for non-coagulated samples (QIAgen Systems, Inc., Valencia, CA, USA).	Human	blood	NA	female	Iberian Population in Spain	obese	Overweight and obesity	80	2.15	0.99
EGAN00001617289	EGAN00001617289	Genomic DNA extracted from peripheral white blood cells using two kits, the Qiamp DNA Investigator Kit for coagulated samples and Qiamp DNA Mini & Blood Mini Kit for non-coagulated samples (QIAgen Systems, Inc., Valencia, CA, USA).	Human	blood	NA	female	Iberian Population in Spain	overweight	Overweight and obesity	82	0.87	0.93
EGAN00001617290	EGAN00001617290	Genomic DNA extracted from peripheral white blood cells using two kits, the Qiamp DNA Investigator Kit for coagulated samples and Qiamp DNA Mini & Blood Mini Kit for non-coagulated samples (QIAgen Systems, Inc., Valencia, CA, USA).	Human	blood	NA	female	Iberian Population in Spain	overweight	Overweight and obesity	90	0.88	1.12
EGAN00001617291	EGAN00001617291	Genomic DNA extracted from peripheral white blood cells using two kits, the Qiamp DNA Investigator Kit for coagulated samples and Qiamp DNA Mini & Blood Mini Kit for non-coagulated samples (QIAgen Systems, Inc., Valencia, CA, USA).	Human	blood	NA	female	Iberian Population in Spain	overweight	Overweight and obesity	84	1.1	0.8
EGAN00001617292	EGAN00001617292	Genomic DNA extracted from peripheral white blood cells using two kits, the Qiamp DNA Investigator Kit for coagulated samples and Qiamp DNA Mini & Blood Mini Kit for non-coagulated samples (QIAgen Systems, Inc., Valencia, CA, USA).	Human	blood	NA	female	Iberian Population in Spain	obese	Overweight and obesity	80	1.67	2.16
EGAN00001617293	EGAN00001617293	Genomic DNA extracted from peripheral white blood cells using two kits, the Qiamp DNA Investigator Kit for coagulated samples and Qiamp DNA Mini & Blood Mini Kit for non-coagulated samples (QIAgen Systems, Inc., Valencia, CA, USA).	Human	blood	NA	female	Iberian Population in Spain	overweight	Overweight and obesity	84	1.17	0.64
EGAN00001617294	EGAN00001617294	Genomic DNA extracted from peripheral white blood cells using two kits, the Qiamp DNA Investigator Kit for coagulated samples and Qiamp DNA Mini & Blood Mini Kit for non-coagulated samples (QIAgen Systems, Inc., Valencia, CA, USA).	Human	blood	NA	female	Iberian Population in Spain	obese	Overweight and obesity	80	3.08	2.89
EGAN00001617295	EGAN00001617295	Genomic DNA extracted from peripheral white blood cells using two kits, the Qiamp DNA Investigator Kit for coagulated samples and Qiamp DNA Mini & Blood Mini Kit for non-coagulated samples (QIAgen Systems, Inc., Valencia, CA, USA).	Human	blood	NA	female	Iberian Population in Spain	obese	Overweight and obesity	80	1.09	4.05
EGAN00001617296	EGAN00001617296	Genomic DNA extracted from peripheral white blood cells using two kits, the Qiamp DNA Investigator Kit for coagulated samples and Qiamp DNA Mini & Blood Mini Kit for non-coagulated samples (QIAgen Systems, Inc., Valencia, CA, USA).	Human	blood	NA	female	Iberian Population in Spain	obese	Overweight and obesity	83	1.56	2.91
EGAN00001617297	EGAN00001617297	Genomic DNA extracted from peripheral white blood cells using two kits, the Qiamp DNA Investigator Kit for coagulated samples and Qiamp DNA Mini & Blood Mini Kit for non-coagulated samples (QIAgen Systems, Inc., Valencia, CA, USA).	Human	blood	NA	female	Iberian Population in Spain	obese	Overweight and obesity	81	1.52	2.35
EGAN00001617298	EGAN00001617298	Genomic DNA extracted from peripheral white blood cells using two kits, the Qiamp DNA Investigator Kit for coagulated samples and Qiamp DNA Mini & Blood Mini Kit for non-coagulated samples (QIAgen Systems, Inc., Valencia, CA, USA).	Human	blood	NA	female	Iberian Population in Spain	obese	Overweight and obesity	88	2.11	3.38
EGAN00001617299	EGAN00001617299	Genomic DNA extracted from peripheral white blood cells using two kits, the Qiamp DNA Investigator Kit for coagulated samples and Qiamp DNA Mini & Blood Mini Kit for non-coagulated samples (QIAgen Systems, Inc., Valencia, CA, USA).	Human	blood	NA	female	Iberian Population in Spain	obese	Overweight and obesity	85	1.68	2.95
EGAN00001617300	EGAN00001617300	Genomic DNA extracted from peripheral white blood cells using two kits, the Qiamp DNA Investigator Kit for coagulated samples and Qiamp DNA Mini & Blood Mini Kit for non-coagulated samples (QIAgen Systems, Inc., Valencia, CA, USA).	Human	blood	NA	female	Iberian Population in Spain	obese	Overweight and obesity	84	1.31	3.69
EGAN00001617301	EGAN00001617301	Genomic DNA extracted from peripheral white blood cells using two kits, the Qiamp DNA Investigator Kit for coagulated samples and Qiamp DNA Mini & Blood Mini Kit for non-coagulated samples (QIAgen Systems, Inc., Valencia, CA, USA).	Human	blood	NA	female	Iberian Population in Spain	obese	Overweight and obesity	76	2.33	2.46
EGAN00001617302	EGAN00001617302	Genomic DNA extracted from peripheral white blood cells using two kits, the Qiamp DNA Investigator Kit for coagulated samples and Qiamp DNA Mini & Blood Mini Kit for non-coagulated samples (QIAgen Systems, Inc., Valencia, CA, USA).	Human	blood	NA	female	Iberian Population in Spain	obese	Overweight and obesity	76	2.27	2.05
EGAN00001617303	EGAN00001617303	Genomic DNA extracted from peripheral white blood cells using two kits, the Qiamp DNA Investigator Kit for coagulated samples and Qiamp DNA Mini & Blood Mini Kit for non-coagulated samples (QIAgen Systems, Inc., Valencia, CA, USA).	Human	blood	NA	female	Iberian Population in Spain	overweight	Overweight and obesity	90	8.51	2.01
EGAN00001617304	EGAN00001617304	Genomic DNA extracted from peripheral white blood cells using two kits, the Qiamp DNA Investigator Kit for coagulated samples and Qiamp DNA Mini & Blood Mini Kit for non-coagulated samples (QIAgen Systems, Inc., Valencia, CA, USA).	Human	blood	NA	female	Iberian Population in Spain	obese	Overweight and obesity	80	NA	1.98
EGAN00001617305	EGAN00001617305	Genomic DNA extracted from peripheral white blood cells using two kits, the Qiamp DNA Investigator Kit for coagulated samples and Qiamp DNA Mini & Blood Mini Kit for non-coagulated samples (QIAgen Systems, Inc., Valencia, CA, USA).	Human	blood	NA	female	Iberian Population in Spain	obese	Overweight and obesity	71	2.95	2.35
EGAN00001617306	EGAN00001617306	Genomic DNA extracted from peripheral white blood cells using two kits, the Qiamp DNA Investigator Kit for coagulated samples and Qiamp DNA Mini & Blood Mini Kit for non-coagulated samples (QIAgen Systems, Inc., Valencia, CA, USA).	Human	blood	NA	female	Iberian Population in Spain	obese	Overweight and obesity	90	4.47	2.65
EGAN00001617307	EGAN00001617307	Genomic DNA extracted from peripheral white blood cells using two kits, the Qiamp DNA Investigator Kit for coagulated samples and Qiamp DNA Mini & Blood Mini Kit for non-coagulated samples (QIAgen Systems, Inc., Valencia, CA, USA).	Human	blood	NA	female	Iberian Population in Spain	obese	Overweight and obesity	72	NA	2.08
EGAN00001617308	EGAN00001617308	Genomic DNA extracted from peripheral white blood cells using two kits, the Qiamp DNA Investigator Kit for coagulated samples and Qiamp DNA Mini & Blood Mini Kit for non-coagulated samples (QIAgen Systems, Inc., Valencia, CA, USA).	Human	blood	NA	female	Iberian Population in Spain	obese	Overweight and obesity	76	3.79	2.77
EGAN00001617309	EGAN00001617309	Genomic DNA extracted from peripheral white blood cells using two kits, the Qiamp DNA Investigator Kit for coagulated samples and Qiamp DNA Mini & Blood Mini Kit for non-coagulated samples (QIAgen Systems, Inc., Valencia, CA, USA).	Human	blood	NA	female	Iberian Population in Spain	obese	Overweight and obesity	83	2.52	2.57
EGAN00001617310	EGAN00001617310	Genomic DNA extracted from peripheral white blood cells using two kits, the Qiamp DNA Investigator Kit for coagulated samples and Qiamp DNA Mini & Blood Mini Kit for non-coagulated samples (QIAgen Systems, Inc., Valencia, CA, USA).	Human	blood	NA	female	Iberian Population in Spain	obese	Overweight and obesity	77	0.38	5.55
EGAN00001617311	EGAN00001617311	Genomic DNA extracted from peripheral white blood cells using two kits, the Qiamp DNA Investigator Kit for coagulated samples and Qiamp DNA Mini & Blood Mini Kit for non-coagulated samples (QIAgen Systems, Inc., Valencia, CA, USA).	Human	blood	NA	female	Iberian Population in Spain	obese	Overweight and obesity	81	1.78	2.2
EGAN00001617312	EGAN00001617312	Genomic DNA extracted from peripheral white blood cells using two kits, the Qiamp DNA Investigator Kit for coagulated samples and Qiamp DNA Mini & Blood Mini Kit for non-coagulated samples (QIAgen Systems, Inc., Valencia, CA, USA).	Human	blood	NA	female	Iberian Population in Spain	obese	Overweight and obesity	81	2.36	2.23
EGAN00001617313	EGAN00001617313	Genomic DNA extracted from peripheral white blood cells using two kits, the Qiamp DNA Investigator Kit for coagulated samples and Qiamp DNA Mini & Blood Mini Kit for non-coagulated samples (QIAgen Systems, Inc., Valencia, CA, USA).	Human	blood	NA	female	Iberian Population in Spain	obese	Overweight and obesity	73	1.98	3.33
EGAN00001617314	EGAN00001617314	Genomic DNA extracted from peripheral white blood cells using two kits, the Qiamp DNA Investigator Kit for coagulated samples and Qiamp DNA Mini & Blood Mini Kit for non-coagulated samples (QIAgen Systems, Inc., Valencia, CA, USA).	Human	blood	NA	female	Iberian Population in Spain	overweight	Overweight and obesity	77	0.76	1.09
EGAN00001617315	EGAN00001617315	Genomic DNA extracted from peripheral white blood cells using two kits, the Qiamp DNA Investigator Kit for coagulated samples and Qiamp DNA Mini & Blood Mini Kit for non-coagulated samples (QIAgen Systems, Inc., Valencia, CA, USA).	Human	blood	NA	female	Iberian Population in Spain	obese	Overweight and obesity	79	1.42	2.35
EGAN00001617316	EGAN00001617316	Genomic DNA extracted from peripheral white blood cells using two kits, the Qiamp DNA Investigator Kit for coagulated samples and Qiamp DNA Mini & Blood Mini Kit for non-coagulated samples (QIAgen Systems, Inc., Valencia, CA, USA).	Human	blood	NA	female	Iberian Population in Spain	obese	Overweight and obesity	71	2.1	5.42
EGAN00001617317	EGAN00001617317	Genomic DNA extracted from peripheral white blood cells using two kits, the Qiamp DNA Investigator Kit for coagulated samples and Qiamp DNA Mini & Blood Mini Kit for non-coagulated samples (QIAgen Systems, Inc., Valencia, CA, USA).	Human	blood	NA	female	Iberian Population in Spain	obese	Overweight and obesity	78	3.52	3.58
EGAN00001617318	EGAN00001617318	Genomic DNA extracted from peripheral white blood cells using two kits, the Qiamp DNA Investigator Kit for coagulated samples and Qiamp DNA Mini & Blood Mini Kit for non-coagulated samples (QIAgen Systems, Inc., Valencia, CA, USA).	Human	blood	NA	female	Iberian Population in Spain	obese	Overweight and obesity	77	3.27	2.43
EGAN00001617319	EGAN00001617319	Genomic DNA extracted from peripheral white blood cells using two kits, the Qiamp DNA Investigator Kit for coagulated samples and Qiamp DNA Mini & Blood Mini Kit for non-coagulated samples (QIAgen Systems, Inc., Valencia, CA, USA).	Human	blood	NA	female	Iberian Population in Spain	obese	Overweight and obesity	78	2.18	2.78
EGAN00001617320	EGAN00001617320	Genomic DNA extracted from peripheral white blood cells using two kits, the Qiamp DNA Investigator Kit for coagulated samples and Qiamp DNA Mini & Blood Mini Kit for non-coagulated samples (QIAgen Systems, Inc., Valencia, CA, USA).	Human	blood	NA	female	Iberian Population in Spain	obese	Overweight and obesity	90	1.13	3.92
EGAN00001617321	EGAN00001617321	Genomic DNA extracted from peripheral white blood cells using two kits, the Qiamp DNA Investigator Kit for coagulated samples and Qiamp DNA Mini & Blood Mini Kit for non-coagulated samples (QIAgen Systems, Inc., Valencia, CA, USA).	Human	blood	NA	female	Iberian Population in Spain	obese	Overweight and obesity	77	0.67	2.58
EGAN00001617322	EGAN00001617322	Genomic DNA extracted from peripheral white blood cells using two kits, the Qiamp DNA Investigator Kit for coagulated samples and Qiamp DNA Mini & Blood Mini Kit for non-coagulated samples (QIAgen Systems, Inc., Valencia, CA, USA).	Human	blood	NA	female	Iberian Population in Spain	obese	Overweight and obesity	87	4.92	3.86
EGAN00001617323	EGAN00001617323	Genomic DNA extracted from peripheral white blood cells using two kits, the Qiamp DNA Investigator Kit for coagulated samples and Qiamp DNA Mini & Blood Mini Kit for non-coagulated samples (QIAgen Systems, Inc., Valencia, CA, USA).	Human	blood	NA	female	Iberian Population in Spain	obese	Overweight and obesity	79	5.36	3.31
EGAN00001617324	EGAN00001617324	Genomic DNA extracted from peripheral white blood cells using two kits, the Qiamp DNA Investigator Kit for coagulated samples and Qiamp DNA Mini & Blood Mini Kit for non-coagulated samples (QIAgen Systems, Inc., Valencia, CA, USA).	Human	blood	NA	female	Iberian Population in Spain	overweight	Overweight and obesity	73	0.38	1.35
EGAN00001617325	EGAN00001617325	Genomic DNA extracted from peripheral white blood cells using two kits, the Qiamp DNA Investigator Kit for coagulated samples and Qiamp DNA Mini & Blood Mini Kit for non-coagulated samples (QIAgen Systems, Inc., Valencia, CA, USA).	Human	blood	NA	female	Iberian Population in Spain	obese	Overweight and obesity	78	5.45	4.15
EGAN00001617326	EGAN00001617326	Genomic DNA extracted from peripheral white blood cells using two kits, the Qiamp DNA Investigator Kit for coagulated samples and Qiamp DNA Mini & Blood Mini Kit for non-coagulated samples (QIAgen Systems, Inc., Valencia, CA, USA).	Human	blood	NA	female	Iberian Population in Spain	obese	Overweight and obesity	78	3.64	2.65
EGAN00001617327	EGAN00001617327	Genomic DNA extracted from peripheral white blood cells using two kits, the Qiamp DNA Investigator Kit for coagulated samples and Qiamp DNA Mini & Blood Mini Kit for non-coagulated samples (QIAgen Systems, Inc., Valencia, CA, USA).	Human	blood	NA	female	Iberian Population in Spain	obese	Overweight and obesity	81	3.46	1.86
EGAN00001617328	EGAN00001617328	Genomic DNA extracted from peripheral white blood cells using two kits, the Qiamp DNA Investigator Kit for coagulated samples and Qiamp DNA Mini & Blood Mini Kit for non-coagulated samples (QIAgen Systems, Inc., Valencia, CA, USA).	Human	blood	NA	female	Iberian Population in Spain	obese	Overweight and obesity	77	1.44	2.08
EGAN00001617329	EGAN00001617329	Genomic DNA extracted from peripheral white blood cells using two kits, the Qiamp DNA Investigator Kit for coagulated samples and Qiamp DNA Mini & Blood Mini Kit for non-coagulated samples (QIAgen Systems, Inc., Valencia, CA, USA).	Human	blood	NA	female	Iberian Population in Spain	obese	Overweight and obesity	74	5.41	3.38
EGAN00001617330	EGAN00001617330	Genomic DNA extracted from peripheral white blood cells using two kits, the Qiamp DNA Investigator Kit for coagulated samples and Qiamp DNA Mini & Blood Mini Kit for non-coagulated samples (QIAgen Systems, Inc., Valencia, CA, USA).	Human	blood	NA	female	Iberian Population in Spain	obese	Overweight and obesity	78	3.91	2.76
EGAN00001617331	EGAN00001617331	Genomic DNA extracted from peripheral white blood cells using two kits, the Qiamp DNA Investigator Kit for coagulated samples and Qiamp DNA Mini & Blood Mini Kit for non-coagulated samples (QIAgen Systems, Inc., Valencia, CA, USA).	Human	blood	NA	female	Iberian Population in Spain	obese	Overweight and obesity	81	3.26	3.34
EGAN00001617332	EGAN00001617332	Genomic DNA extracted from peripheral white blood cells using two kits, the Qiamp DNA Investigator Kit for coagulated samples and Qiamp DNA Mini & Blood Mini Kit for non-coagulated samples (QIAgen Systems, Inc., Valencia, CA, USA).	Human	blood	NA	female	Iberian Population in Spain	obese	Overweight and obesity	79	2.87	2.84
EGAN00001617333	EGAN00001617333	Genomic DNA extracted from peripheral white blood cells using two kits, the Qiamp DNA Investigator Kit for coagulated samples and Qiamp DNA Mini & Blood Mini Kit for non-coagulated samples (QIAgen Systems, Inc., Valencia, CA, USA).	Human	blood	NA	female	Iberian Population in Spain	obese	Overweight and obesity	78	2.56	2.55
EGAN00001617334	EGAN00001617334	Genomic DNA extracted from peripheral white blood cells using two kits, the Qiamp DNA Investigator Kit for coagulated samples and Qiamp DNA Mini & Blood Mini Kit for non-coagulated samples (QIAgen Systems, Inc., Valencia, CA, USA).	Human	blood	NA	female	Iberian Population in Spain	overweight	Overweight and obesity	80	2.8	1.01
EGAN00001617335	EGAN00001617335	Genomic DNA extracted from peripheral white blood cells using two kits, the Qiamp DNA Investigator Kit for coagulated samples and Qiamp DNA Mini & Blood Mini Kit for non-coagulated samples (QIAgen Systems, Inc., Valencia, CA, USA).	Human	blood	NA	female	Iberian Population in Spain	overweight	Overweight and obesity	93	8.22	1.83
EGAN00001617336	EGAN00001617336	Genomic DNA extracted from peripheral white blood cells using two kits, the Qiamp DNA Investigator Kit for coagulated samples and Qiamp DNA Mini & Blood Mini Kit for non-coagulated samples (QIAgen Systems, Inc., Valencia, CA, USA).	Human	blood	NA	female	Iberian Population in Spain	overweight	Overweight and obesity	83	0.94	1.69
EGAN00001617337	EGAN00001617337	Genomic DNA extracted from peripheral white blood cells using two kits, the Qiamp DNA Investigator Kit for coagulated samples and Qiamp DNA Mini & Blood Mini Kit for non-coagulated samples (QIAgen Systems, Inc., Valencia, CA, USA).	Human	blood	NA	female	Iberian Population in Spain	overweight	Overweight and obesity	94	1.39	1.64
EGAN00001617338	EGAN00001617338	Genomic DNA extracted from peripheral white blood cells using two kits, the Qiamp DNA Investigator Kit for coagulated samples and Qiamp DNA Mini & Blood Mini Kit for non-coagulated samples (QIAgen Systems, Inc., Valencia, CA, USA).	Human	blood	NA	female	Iberian Population in Spain	obese	Overweight and obesity	77	2.3	3.59
EGAN00001617339	EGAN00001617339	Genomic DNA extracted from peripheral white blood cells using two kits, the Qiamp DNA Investigator Kit for coagulated samples and Qiamp DNA Mini & Blood Mini Kit for non-coagulated samples (QIAgen Systems, Inc., Valencia, CA, USA).	Human	blood	NA	female	Iberian Population in Spain	overweight	Overweight and obesity	83	1.27	0.45
EGAN00001617340	EGAN00001617340	Genomic DNA extracted from peripheral white blood cells using two kits, the Qiamp DNA Investigator Kit for coagulated samples and Qiamp DNA Mini & Blood Mini Kit for non-coagulated samples (QIAgen Systems, Inc., Valencia, CA, USA).	Human	blood	NA	female	Iberian Population in Spain	obese	Overweight and obesity	77	1.77	4.19
EGAN00001617341	EGAN00001617341	Genomic DNA extracted from peripheral white blood cells using two kits, the Qiamp DNA Investigator Kit for coagulated samples and Qiamp DNA Mini & Blood Mini Kit for non-coagulated samples (QIAgen Systems, Inc., Valencia, CA, USA).	Human	blood	NA	female	Iberian Population in Spain	overweight	Overweight and obesity	67	2.58	1.18
EGAN00001617342	EGAN00001617342	Genomic DNA extracted from peripheral white blood cells using two kits, the Qiamp DNA Investigator Kit for coagulated samples and Qiamp DNA Mini & Blood Mini Kit for non-coagulated samples (QIAgen Systems, Inc., Valencia, CA, USA).	Human	blood	NA	female	Iberian Population in Spain	obese	Overweight and obesity	91	3.6	3.73
EGAN00001617343	EGAN00001617343	Genomic DNA extracted from peripheral white blood cells using two kits, the Qiamp DNA Investigator Kit for coagulated samples and Qiamp DNA Mini & Blood Mini Kit for non-coagulated samples (QIAgen Systems, Inc., Valencia, CA, USA).	Human	blood	NA	female	Iberian Population in Spain	obese	Overweight and obesity	90	1.78	3.34
EGAN00001617344	EGAN00001617344	Genomic DNA extracted from peripheral white blood cells using two kits, the Qiamp DNA Investigator Kit for coagulated samples and Qiamp DNA Mini & Blood Mini Kit for non-coagulated samples (QIAgen Systems, Inc., Valencia, CA, USA).	Human	blood	NA	female	Iberian Population in Spain	overweight	Overweight and obesity	91	2.74	1.86
EGAN00001617345	EGAN00001617345	Genomic DNA extracted from peripheral white blood cells using two kits, the Qiamp DNA Investigator Kit for coagulated samples and Qiamp DNA Mini & Blood Mini Kit for non-coagulated samples (QIAgen Systems, Inc., Valencia, CA, USA).	Human	blood	NA	female	Iberian Population in Spain	obese	Overweight and obesity	91	3.17	3.25
EGAN00001617346	EGAN00001617346	Genomic DNA extracted from peripheral white blood cells using two kits, the Qiamp DNA Investigator Kit for coagulated samples and Qiamp DNA Mini & Blood Mini Kit for non-coagulated samples (QIAgen Systems, Inc., Valencia, CA, USA).	Human	blood	NA	female	Iberian Population in Spain	overweight	Overweight and obesity	81	4.12	1.91
EGAN00001617347	EGAN00001617347	Genomic DNA extracted from peripheral white blood cells using two kits, the Qiamp DNA Investigator Kit for coagulated samples and Qiamp DNA Mini & Blood Mini Kit for non-coagulated samples (QIAgen Systems, Inc., Valencia, CA, USA).	Human	blood	NA	female	Iberian Population in Spain	overweight	Overweight and obesity	80	2.53	1.29
EGAN00001617348	EGAN00001617348	Genomic DNA extracted from peripheral white blood cells using two kits, the Qiamp DNA Investigator Kit for coagulated samples and Qiamp DNA Mini & Blood Mini Kit for non-coagulated samples (QIAgen Systems, Inc., Valencia, CA, USA).	Human	blood	NA	female	Iberian Population in Spain	overweight	Overweight and obesity	81	1.06	1.4
EGAN00001617349	EGAN00001617349	Genomic DNA extracted from peripheral white blood cells using two kits, the Qiamp DNA Investigator Kit for coagulated samples and Qiamp DNA Mini & Blood Mini Kit for non-coagulated samples (QIAgen Systems, Inc., Valencia, CA, USA).	Human	blood	NA	female	Iberian Population in Spain	obese	Overweight and obesity	79	2.05	2.01
EGAN00001617350	EGAN00001617350	Genomic DNA extracted from peripheral white blood cells using two kits, the Qiamp DNA Investigator Kit for coagulated samples and Qiamp DNA Mini & Blood Mini Kit for non-coagulated samples (QIAgen Systems, Inc., Valencia, CA, USA).	Human	blood	NA	female	Iberian Population in Spain	obese	Overweight and obesity	83	0.84	3.69
EGAN00001617351	EGAN00001617351	Genomic DNA extracted from peripheral white blood cells using two kits, the Qiamp DNA Investigator Kit for coagulated samples and Qiamp DNA Mini & Blood Mini Kit for non-coagulated samples (QIAgen Systems, Inc., Valencia, CA, USA).	Human	blood	NA	female	Iberian Population in Spain	overweight	Overweight and obesity	83	2.3	1.96
EGAN00001617352	EGAN00001617352	Genomic DNA extracted from peripheral white blood cells using two kits, the Qiamp DNA Investigator Kit for coagulated samples and Qiamp DNA Mini & Blood Mini Kit for non-coagulated samples (QIAgen Systems, Inc., Valencia, CA, USA).	Human	blood	NA	female	Iberian Population in Spain	obese	Overweight and obesity	85	0.99	3.69
EGAN00001617353	EGAN00001617353	Genomic DNA extracted from peripheral white blood cells using two kits, the Qiamp DNA Investigator Kit for coagulated samples and Qiamp DNA Mini & Blood Mini Kit for non-coagulated samples (QIAgen Systems, Inc., Valencia, CA, USA).	Human	blood	NA	female	Iberian Population in Spain	obese	Overweight and obesity	65	1.67	3.5
EGAN00001617354	EGAN00001617354	Genomic DNA extracted from peripheral white blood cells using two kits, the Qiamp DNA Investigator Kit for coagulated samples and Qiamp DNA Mini & Blood Mini Kit for non-coagulated samples (QIAgen Systems, Inc., Valencia, CA, USA).	Human	blood	NA	female	Iberian Population in Spain	obese	Overweight and obesity	72	5.3	5.13
EGAN00001617355	EGAN00001617355	Genomic DNA extracted from peripheral white blood cells using two kits, the Qiamp DNA Investigator Kit for coagulated samples and Qiamp DNA Mini & Blood Mini Kit for non-coagulated samples (QIAgen Systems, Inc., Valencia, CA, USA).	Human	blood	NA	female	Iberian Population in Spain	obese	Overweight and obesity	78	5.03	4.04
EGAN00001617356	EGAN00001617356	Genomic DNA extracted from peripheral white blood cells using two kits, the Qiamp DNA Investigator Kit for coagulated samples and Qiamp DNA Mini & Blood Mini Kit for non-coagulated samples (QIAgen Systems, Inc., Valencia, CA, USA).	Human	blood	NA	female	Iberian Population in Spain	obese	Overweight and obesity	70	1.37	2.5
EGAN00001617357	EGAN00001617357	Genomic DNA extracted from peripheral white blood cells using two kits, the Qiamp DNA Investigator Kit for coagulated samples and Qiamp DNA Mini & Blood Mini Kit for non-coagulated samples (QIAgen Systems, Inc., Valencia, CA, USA).	Human	blood	NA	female	Iberian Population in Spain	obese	Overweight and obesity	78	4.12	3.51
EGAN00001617358	EGAN00001617358	Genomic DNA extracted from peripheral white blood cells using two kits, the Qiamp DNA Investigator Kit for coagulated samples and Qiamp DNA Mini & Blood Mini Kit for non-coagulated samples (QIAgen Systems, Inc., Valencia, CA, USA).	Human	blood	NA	female	Iberian Population in Spain	obese	Overweight and obesity	72	3.27	2.21
EGAN00001617359	EGAN00001617359	Genomic DNA extracted from peripheral white blood cells using two kits, the Qiamp DNA Investigator Kit for coagulated samples and Qiamp DNA Mini & Blood Mini Kit for non-coagulated samples (QIAgen Systems, Inc., Valencia, CA, USA).	Human	blood	NA	female	Iberian Population in Spain	obese	Overweight and obesity	83	0.72	2.23
EGAN00001617360	EGAN00001617360	Genomic DNA extracted from peripheral white blood cells using two kits, the Qiamp DNA Investigator Kit for coagulated samples and Qiamp DNA Mini & Blood Mini Kit for non-coagulated samples (QIAgen Systems, Inc., Valencia, CA, USA).	Human	blood	NA	female	Iberian Population in Spain	obese	Overweight and obesity	83	3.42	2.28
EGAN00001617361	EGAN00001617361	Genomic DNA extracted from peripheral white blood cells using two kits, the Qiamp DNA Investigator Kit for coagulated samples and Qiamp DNA Mini & Blood Mini Kit for non-coagulated samples (QIAgen Systems, Inc., Valencia, CA, USA).	Human	blood	NA	female	Iberian Population in Spain	overweight	Overweight and obesity	82	1.78	1.08
EGAN00001617362	EGAN00001617362	Genomic DNA extracted from peripheral white blood cells using two kits, the Qiamp DNA Investigator Kit for coagulated samples and Qiamp DNA Mini & Blood Mini Kit for non-coagulated samples (QIAgen Systems, Inc., Valencia, CA, USA).	Human	blood	NA	female	Iberian Population in Spain	overweight	Overweight and obesity	87	1.78	1.49
EGAN00001617363	EGAN00001617363	Genomic DNA extracted from peripheral white blood cells using two kits, the Qiamp DNA Investigator Kit for coagulated samples and Qiamp DNA Mini & Blood Mini Kit for non-coagulated samples (QIAgen Systems, Inc., Valencia, CA, USA).	Human	blood	NA	female	Iberian Population in Spain	obese	Overweight and obesity	82	4.19	4.44
EGAN00001617364	EGAN00001617364	Genomic DNA extracted from peripheral white blood cells using two kits, the Qiamp DNA Investigator Kit for coagulated samples and Qiamp DNA Mini & Blood Mini Kit for non-coagulated samples (QIAgen Systems, Inc., Valencia, CA, USA).	Human	blood	NA	female	Iberian Population in Spain	overweight	Overweight and obesity	78	NA	0.82
EGAN00001617365	EGAN00001617365	Genomic DNA extracted from peripheral white blood cells using two kits, the Qiamp DNA Investigator Kit for coagulated samples and Qiamp DNA Mini & Blood Mini Kit for non-coagulated samples (QIAgen Systems, Inc., Valencia, CA, USA).	Human	blood	NA	female	Iberian Population in Spain	obese	Overweight and obesity	92	9.81	5.48
EGAN00001617366	EGAN00001617366	Genomic DNA extracted from peripheral white blood cells using two kits, the Qiamp DNA Investigator Kit for coagulated samples and Qiamp DNA Mini & Blood Mini Kit for non-coagulated samples (QIAgen Systems, Inc., Valencia, CA, USA).	Human	blood	NA	female	Iberian Population in Spain	obese	Overweight and obesity	86	2.25	2.35
EGAN00001617367	EGAN00001617367	Genomic DNA extracted from peripheral white blood cells using two kits, the Qiamp DNA Investigator Kit for coagulated samples and Qiamp DNA Mini & Blood Mini Kit for non-coagulated samples (QIAgen Systems, Inc., Valencia, CA, USA).	Human	blood	NA	female	Iberian Population in Spain	overweight	Overweight and obesity	76	1.97	1.3
EGAN00001617368	EGAN00001617368	Genomic DNA extracted from peripheral white blood cells using two kits, the Qiamp DNA Investigator Kit for coagulated samples and Qiamp DNA Mini & Blood Mini Kit for non-coagulated samples (QIAgen Systems, Inc., Valencia, CA, USA).	Human	blood	NA	female	Iberian Population in Spain	obese	Overweight and obesity	83	7.09	5.91
EGAN00001617369	EGAN00001617369	Genomic DNA extracted from peripheral white blood cells using two kits, the Qiamp DNA Investigator Kit for coagulated samples and Qiamp DNA Mini & Blood Mini Kit for non-coagulated samples (QIAgen Systems, Inc., Valencia, CA, USA).	Human	blood	NA	female	Iberian Population in Spain	obese	Overweight and obesity	NA	NA	3.59
EGAN00001617370	EGAN00001617370	Genomic DNA extracted from peripheral white blood cells using two kits, the Qiamp DNA Investigator Kit for coagulated samples and Qiamp DNA Mini & Blood Mini Kit for non-coagulated samples (QIAgen Systems, Inc., Valencia, CA, USA).	Human	blood	NA	female	Iberian Population in Spain	obese	Overweight and obesity	84	2.09	2.18
EGAN00001617371	EGAN00001617371	Genomic DNA extracted from peripheral white blood cells using two kits, the Qiamp DNA Investigator Kit for coagulated samples and Qiamp DNA Mini & Blood Mini Kit for non-coagulated samples (QIAgen Systems, Inc., Valencia, CA, USA).	Human	blood	NA	female	Iberian Population in Spain	obese	Overweight and obesity	87	3.48	1.78
EGAN00001617372	EGAN00001617372	Genomic DNA extracted from peripheral white blood cells using two kits, the Qiamp DNA Investigator Kit for coagulated samples and Qiamp DNA Mini & Blood Mini Kit for non-coagulated samples (QIAgen Systems, Inc., Valencia, CA, USA).	Human	blood	NA	female	Iberian Population in Spain	overweight	Overweight and obesity	77	0.61	0.4
EGAN00001617373	EGAN00001617373	Genomic DNA extracted from peripheral white blood cells using two kits, the Qiamp DNA Investigator Kit for coagulated samples and Qiamp DNA Mini & Blood Mini Kit for non-coagulated samples (QIAgen Systems, Inc., Valencia, CA, USA).	Human	blood	NA	female	Iberian Population in Spain	obese	Overweight and obesity	74	4.33	4.05
EGAN00001617374	EGAN00001617374	Genomic DNA extracted from peripheral white blood cells using two kits, the Qiamp DNA Investigator Kit for coagulated samples and Qiamp DNA Mini & Blood Mini Kit for non-coagulated samples (QIAgen Systems, Inc., Valencia, CA, USA).	Human	blood	NA	female	Iberian Population in Spain	obese	Overweight and obesity	84	1.91	2.98
EGAN00001617375	EGAN00001617375	Genomic DNA extracted from peripheral white blood cells using two kits, the Qiamp DNA Investigator Kit for coagulated samples and Qiamp DNA Mini & Blood Mini Kit for non-coagulated samples (QIAgen Systems, Inc., Valencia, CA, USA).	Human	blood	NA	female	Iberian Population in Spain	overweight	Overweight and obesity	93	0.67	0.65
EGAN00001617376	EGAN00001617376	Genomic DNA extracted from peripheral white blood cells using two kits, the Qiamp DNA Investigator Kit for coagulated samples and Qiamp DNA Mini & Blood Mini Kit for non-coagulated samples (QIAgen Systems, Inc., Valencia, CA, USA).	Human	blood	NA	female	Iberian Population in Spain	obese	Overweight and obesity	73	2.52	6.32
EGAN00001617377	EGAN00001617377	Genomic DNA extracted from peripheral white blood cells using two kits, the Qiamp DNA Investigator Kit for coagulated samples and Qiamp DNA Mini & Blood Mini Kit for non-coagulated samples (QIAgen Systems, Inc., Valencia, CA, USA).	Human	blood	NA	female	Iberian Population in Spain	overweight	Overweight and obesity	74	NA	1.21
EGAN00001617378	EGAN00001617378	Genomic DNA extracted from peripheral white blood cells using two kits, the Qiamp DNA Investigator Kit for coagulated samples and Qiamp DNA Mini & Blood Mini Kit for non-coagulated samples (QIAgen Systems, Inc., Valencia, CA, USA).	Human	blood	NA	female	Iberian Population in Spain	overweight	Overweight and obesity	74	5.32	1.48
EGAN00001617379	EGAN00001617379	Genomic DNA extracted from peripheral white blood cells using two kits, the Qiamp DNA Investigator Kit for coagulated samples and Qiamp DNA Mini & Blood Mini Kit for non-coagulated samples (QIAgen Systems, Inc., Valencia, CA, USA).	Human	blood	NA	female	Iberian Population in Spain	obese	Overweight and obesity	75	0.37	2.39
EGAN00001617380	EGAN00001617380	Genomic DNA extracted from peripheral white blood cells using two kits, the Qiamp DNA Investigator Kit for coagulated samples and Qiamp DNA Mini & Blood Mini Kit for non-coagulated samples (QIAgen Systems, Inc., Valencia, CA, USA).	Human	blood	NA	female	Iberian Population in Spain	obese	Overweight and obesity	73	2.09	1.91
EGAN00001617381	EGAN00001617381	Genomic DNA extracted from peripheral white blood cells using two kits, the Qiamp DNA Investigator Kit for coagulated samples and Qiamp DNA Mini & Blood Mini Kit for non-coagulated samples (QIAgen Systems, Inc., Valencia, CA, USA).	Human	blood	NA	female	Iberian Population in Spain	obese	Overweight and obesity	82	4.17	3.85
EGAN00001617382	EGAN00001617382	Genomic DNA extracted from peripheral white blood cells using two kits, the Qiamp DNA Investigator Kit for coagulated samples and Qiamp DNA Mini & Blood Mini Kit for non-coagulated samples (QIAgen Systems, Inc., Valencia, CA, USA).	Human	blood	NA	female	Iberian Population in Spain	obese	Overweight and obesity	79	3.04	3.75
EGAN00001617383	EGAN00001617383	Genomic DNA extracted from peripheral white blood cells using two kits, the Qiamp DNA Investigator Kit for coagulated samples and Qiamp DNA Mini & Blood Mini Kit for non-coagulated samples (QIAgen Systems, Inc., Valencia, CA, USA).	Human	blood	NA	female	Iberian Population in Spain	obese	Overweight and obesity	86	3.25	2.31
EGAN00001617384	EGAN00001617384	Genomic DNA extracted from peripheral white blood cells using two kits, the Qiamp DNA Investigator Kit for coagulated samples and Qiamp DNA Mini & Blood Mini Kit for non-coagulated samples (QIAgen Systems, Inc., Valencia, CA, USA).	Human	blood	NA	female	Iberian Population in Spain	obese	Overweight and obesity	74	1.64	2.9
EGAN00001617385	EGAN00001617385	Genomic DNA extracted from peripheral white blood cells using two kits, the Qiamp DNA Investigator Kit for coagulated samples and Qiamp DNA Mini & Blood Mini Kit for non-coagulated samples (QIAgen Systems, Inc., Valencia, CA, USA).	Human	blood	NA	female	Iberian Population in Spain	overweight	Overweight and obesity	79	0.72	1.86
EGAN00001617386	EGAN00001617386	Genomic DNA extracted from peripheral white blood cells using two kits, the Qiamp DNA Investigator Kit for coagulated samples and Qiamp DNA Mini & Blood Mini Kit for non-coagulated samples (QIAgen Systems, Inc., Valencia, CA, USA).	Human	blood	NA	female	Iberian Population in Spain	overweight	Overweight and obesity	73	0.4	1.61
EGAN00001617387	EGAN00001617387	Genomic DNA extracted from peripheral white blood cells using two kits, the Qiamp DNA Investigator Kit for coagulated samples and Qiamp DNA Mini & Blood Mini Kit for non-coagulated samples (QIAgen Systems, Inc., Valencia, CA, USA).	Human	blood	NA	female	Iberian Population in Spain	obese	Overweight and obesity	70	2.73	1.92
EGAN00001617388	EGAN00001617388	Genomic DNA extracted from peripheral white blood cells using two kits, the Qiamp DNA Investigator Kit for coagulated samples and Qiamp DNA Mini & Blood Mini Kit for non-coagulated samples (QIAgen Systems, Inc., Valencia, CA, USA).	Human	blood	NA	female	Iberian Population in Spain	obese	Overweight and obesity	82	3.28	3.24
EGAN00001617389	EGAN00001617389	Genomic DNA extracted from peripheral white blood cells using two kits, the Qiamp DNA Investigator Kit for coagulated samples and Qiamp DNA Mini & Blood Mini Kit for non-coagulated samples (QIAgen Systems, Inc., Valencia, CA, USA).	Human	blood	NA	female	Iberian Population in Spain	obese	Overweight and obesity	81	4.66	3.26
EGAN00001617390	EGAN00001617390	Genomic DNA extracted from peripheral white blood cells using two kits, the Qiamp DNA Investigator Kit for coagulated samples and Qiamp DNA Mini & Blood Mini Kit for non-coagulated samples (QIAgen Systems, Inc., Valencia, CA, USA).	Human	blood	NA	female	Iberian Population in Spain	overweight	Overweight and obesity	77	2.3	3.51
EGAN00001617391	EGAN00001617391	Genomic DNA extracted from peripheral white blood cells using two kits, the Qiamp DNA Investigator Kit for coagulated samples and Qiamp DNA Mini & Blood Mini Kit for non-coagulated samples (QIAgen Systems, Inc., Valencia, CA, USA).	Human	blood	NA	female	Iberian Population in Spain	obese	Overweight and obesity	80	2.57	2.46
EGAN00001617392	EGAN00001617392	Genomic DNA extracted from peripheral white blood cells using two kits, the Qiamp DNA Investigator Kit for coagulated samples and Qiamp DNA Mini & Blood Mini Kit for non-coagulated samples (QIAgen Systems, Inc., Valencia, CA, USA).	Human	blood	NA	female	Iberian Population in Spain	obese	Overweight and obesity	79	4.45	5.5
EGAN00001617393	EGAN00001617393	Genomic DNA extracted from peripheral white blood cells using two kits, the Qiamp DNA Investigator Kit for coagulated samples and Qiamp DNA Mini & Blood Mini Kit for non-coagulated samples (QIAgen Systems, Inc., Valencia, CA, USA).	Human	blood	NA	female	Iberian Population in Spain	obese	Overweight and obesity	92	2.95	2.15
EGAN00001617394	EGAN00001617394	Genomic DNA extracted from peripheral white blood cells using two kits, the Qiamp DNA Investigator Kit for coagulated samples and Qiamp DNA Mini & Blood Mini Kit for non-coagulated samples (QIAgen Systems, Inc., Valencia, CA, USA).	Human	blood	NA	female	Iberian Population in Spain	obese	Overweight and obesity	88	5	2.02
EGAN00001617395	EGAN00001617395	Genomic DNA extracted from peripheral white blood cells using two kits, the Qiamp DNA Investigator Kit for coagulated samples and Qiamp DNA Mini & Blood Mini Kit for non-coagulated samples (QIAgen Systems, Inc., Valencia, CA, USA).	Human	blood	NA	female	Iberian Population in Spain	obese	Overweight and obesity	83	2.4	1.71
EGAN00001617396	EGAN00001617396	Genomic DNA extracted from peripheral white blood cells using two kits, the Qiamp DNA Investigator Kit for coagulated samples and Qiamp DNA Mini & Blood Mini Kit for non-coagulated samples (QIAgen Systems, Inc., Valencia, CA, USA).	Human	blood	NA	female	Iberian Population in Spain	obese	Overweight and obesity	85	3.04	2.82
EGAN00001617397	EGAN00001617397	Genomic DNA extracted from peripheral white blood cells using two kits, the Qiamp DNA Investigator Kit for coagulated samples and Qiamp DNA Mini & Blood Mini Kit for non-coagulated samples (QIAgen Systems, Inc., Valencia, CA, USA).	Human	blood	NA	female	Iberian Population in Spain	obese	Overweight and obesity	80	2.55	3.59
EGAN00001617398	EGAN00001617398	Genomic DNA extracted from peripheral white blood cells using two kits, the Qiamp DNA Investigator Kit for coagulated samples and Qiamp DNA Mini & Blood Mini Kit for non-coagulated samples (QIAgen Systems, Inc., Valencia, CA, USA).	Human	blood	NA	female	Iberian Population in Spain	overweight	Overweight and obesity	84	1.14	1.26
EGAN00001617399	EGAN00001617399	Genomic DNA extracted from peripheral white blood cells using two kits, the Qiamp DNA Investigator Kit for coagulated samples and Qiamp DNA Mini & Blood Mini Kit for non-coagulated samples (QIAgen Systems, Inc., Valencia, CA, USA).	Human	blood	NA	female	Iberian Population in Spain	obese	Overweight and obesity	78	1.05	2.11
EGAN00001617400	EGAN00001617400	Genomic DNA extracted from peripheral white blood cells using two kits, the Qiamp DNA Investigator Kit for coagulated samples and Qiamp DNA Mini & Blood Mini Kit for non-coagulated samples (QIAgen Systems, Inc., Valencia, CA, USA).	Human	blood	NA	female	Iberian Population in Spain	obese	Overweight and obesity	77	0.66	2.9
EGAN00001617401	EGAN00001617401	Genomic DNA extracted from peripheral white blood cells using two kits, the Qiamp DNA Investigator Kit for coagulated samples and Qiamp DNA Mini & Blood Mini Kit for non-coagulated samples (QIAgen Systems, Inc., Valencia, CA, USA).	Human	blood	NA	female	Iberian Population in Spain	obese	Overweight and obesity	78	0.64	2.85
EGAN00001617402	EGAN00001617402	Genomic DNA extracted from peripheral white blood cells using two kits, the Qiamp DNA Investigator Kit for coagulated samples and Qiamp DNA Mini & Blood Mini Kit for non-coagulated samples (QIAgen Systems, Inc., Valencia, CA, USA).	Human	blood	NA	female	Iberian Population in Spain	overweight	Overweight and obesity	83	0.75	0.6
EGAN00001617403	EGAN00001617403	Genomic DNA extracted from peripheral white blood cells using two kits, the Qiamp DNA Investigator Kit for coagulated samples and Qiamp DNA Mini & Blood Mini Kit for non-coagulated samples (QIAgen Systems, Inc., Valencia, CA, USA).	Human	blood	NA	female	Iberian Population in Spain	overweight	Overweight and obesity	88	1.43	0.46
EGAN00001617404	EGAN00001617404	Genomic DNA extracted from peripheral white blood cells using two kits, the Qiamp DNA Investigator Kit for coagulated samples and Qiamp DNA Mini & Blood Mini Kit for non-coagulated samples (QIAgen Systems, Inc., Valencia, CA, USA).	Human	blood	NA	female	Iberian Population in Spain	overweight	Overweight and obesity	84	4.52	0.51
EGAN00001617405	EGAN00001617405	Genomic DNA extracted from peripheral white blood cells using two kits, the Qiamp DNA Investigator Kit for coagulated samples and Qiamp DNA Mini & Blood Mini Kit for non-coagulated samples (QIAgen Systems, Inc., Valencia, CA, USA).	Human	blood	NA	female	Iberian Population in Spain	obese	Overweight and obesity	88	3.35	3.18
EGAN00001617406	EGAN00001617406	Genomic DNA extracted from peripheral white blood cells using two kits, the Qiamp DNA Investigator Kit for coagulated samples and Qiamp DNA Mini & Blood Mini Kit for non-coagulated samples (QIAgen Systems, Inc., Valencia, CA, USA).	Human	blood	NA	female	Iberian Population in Spain	obese	Overweight and obesity	87	8.31	2.66
EGAN00001617407	EGAN00001617407	Genomic DNA extracted from peripheral white blood cells using two kits, the Qiamp DNA Investigator Kit for coagulated samples and Qiamp DNA Mini & Blood Mini Kit for non-coagulated samples (QIAgen Systems, Inc., Valencia, CA, USA).	Human	blood	NA	female	Iberian Population in Spain	obese	Overweight and obesity	90	0.87	1.74
EGAN00001617408	EGAN00001617408	Genomic DNA extracted from peripheral white blood cells using two kits, the Qiamp DNA Investigator Kit for coagulated samples and Qiamp DNA Mini & Blood Mini Kit for non-coagulated samples (QIAgen Systems, Inc., Valencia, CA, USA).	Human	blood	NA	female	Iberian Population in Spain	overweight	Overweight and obesity	83	1.1	0.95
EGAN00001617409	EGAN00001617409	Genomic DNA extracted from peripheral white blood cells using two kits, the Qiamp DNA Investigator Kit for coagulated samples and Qiamp DNA Mini & Blood Mini Kit for non-coagulated samples (QIAgen Systems, Inc., Valencia, CA, USA).	Human	blood	NA	female	Iberian Population in Spain	obese	Overweight and obesity	87	3.74	1.93
EGAN00001617410	EGAN00001617410	Genomic DNA extracted from peripheral white blood cells using two kits, the Qiamp DNA Investigator Kit for coagulated samples and Qiamp DNA Mini & Blood Mini Kit for non-coagulated samples (QIAgen Systems, Inc., Valencia, CA, USA).	Human	blood	NA	female	Iberian Population in Spain	obese	Overweight and obesity	87	1.92	2.12
EGAN00001617411	EGAN00001617411	Genomic DNA extracted from peripheral white blood cells using two kits, the Qiamp DNA Investigator Kit for coagulated samples and Qiamp DNA Mini & Blood Mini Kit for non-coagulated samples (QIAgen Systems, Inc., Valencia, CA, USA).	Human	blood	NA	female	Iberian Population in Spain	overweight	Overweight and obesity	91	3.19	1.23
EGAN00001617412	EGAN00001617412	Genomic DNA extracted from peripheral white blood cells using two kits, the Qiamp DNA Investigator Kit for coagulated samples and Qiamp DNA Mini & Blood Mini Kit for non-coagulated samples (QIAgen Systems, Inc., Valencia, CA, USA).	Human	blood	NA	female	Iberian Population in Spain	obese	Overweight and obesity	91	0.8	2.2
EGAN00001617413	EGAN00001617413	Genomic DNA extracted from peripheral white blood cells using two kits, the Qiamp DNA Investigator Kit for coagulated samples and Qiamp DNA Mini & Blood Mini Kit for non-coagulated samples (QIAgen Systems, Inc., Valencia, CA, USA).	Human	blood	NA	female	Iberian Population in Spain	obese	Overweight and obesity	85	1.81	1.9
EGAN00001617414	EGAN00001617414	Genomic DNA extracted from peripheral white blood cells using two kits, the Qiamp DNA Investigator Kit for coagulated samples and Qiamp DNA Mini & Blood Mini Kit for non-coagulated samples (QIAgen Systems, Inc., Valencia, CA, USA).	Human	blood	NA	female	Iberian Population in Spain	overweight	Overweight and obesity	89	0.66	0.73
EGAN00001617415	EGAN00001617415	Genomic DNA extracted from peripheral white blood cells using two kits, the Qiamp DNA Investigator Kit for coagulated samples and Qiamp DNA Mini & Blood Mini Kit for non-coagulated samples (QIAgen Systems, Inc., Valencia, CA, USA).	Human	blood	NA	female	Iberian Population in Spain	obese	Overweight and obesity	82	1.87	3
EGAN00001617416	EGAN00001617416	Genomic DNA extracted from peripheral white blood cells using two kits, the Qiamp DNA Investigator Kit for coagulated samples and Qiamp DNA Mini & Blood Mini Kit for non-coagulated samples (QIAgen Systems, Inc., Valencia, CA, USA).	Human	blood	NA	female	Iberian Population in Spain	obese	Overweight and obesity	85	6.32	2.9
EGAN00001617417	EGAN00001617417	Genomic DNA extracted from peripheral white blood cells using two kits, the Qiamp DNA Investigator Kit for coagulated samples and Qiamp DNA Mini & Blood Mini Kit for non-coagulated samples (QIAgen Systems, Inc., Valencia, CA, USA).	Human	blood	NA	female	Iberian Population in Spain	overweight	Overweight and obesity	82	1.5	1.15
EGAN00001617418	EGAN00001617418	Genomic DNA extracted from peripheral white blood cells using two kits, the Qiamp DNA Investigator Kit for coagulated samples and Qiamp DNA Mini & Blood Mini Kit for non-coagulated samples (QIAgen Systems, Inc., Valencia, CA, USA).	Human	blood	NA	female	Iberian Population in Spain	obese	Overweight and obesity	80	NA	3.07
EGAN00001617419	EGAN00001617419	Genomic DNA extracted from peripheral white blood cells using two kits, the Qiamp DNA Investigator Kit for coagulated samples and Qiamp DNA Mini & Blood Mini Kit for non-coagulated samples (QIAgen Systems, Inc., Valencia, CA, USA).	Human	blood	NA	female	Iberian Population in Spain	obese	Overweight and obesity	88	4.26	2.46
EGAN00001617420	EGAN00001617420	Genomic DNA extracted from peripheral white blood cells using two kits, the Qiamp DNA Investigator Kit for coagulated samples and Qiamp DNA Mini & Blood Mini Kit for non-coagulated samples (QIAgen Systems, Inc., Valencia, CA, USA).	Human	blood	NA	female	Iberian Population in Spain	overweight	Overweight and obesity	90	1.96	0.96
EGAN00001617421	EGAN00001617421	Genomic DNA extracted from peripheral white blood cells using two kits, the Qiamp DNA Investigator Kit for coagulated samples and Qiamp DNA Mini & Blood Mini Kit for non-coagulated samples (QIAgen Systems, Inc., Valencia, CA, USA).	Human	blood	NA	female	Iberian Population in Spain	overweight	Overweight and obesity	111	3.3	1.2
EGAN00001617422	EGAN00001617422	Genomic DNA extracted from peripheral white blood cells using two kits, the Qiamp DNA Investigator Kit for coagulated samples and Qiamp DNA Mini & Blood Mini Kit for non-coagulated samples (QIAgen Systems, Inc., Valencia, CA, USA).	Human	blood	NA	female	Iberian Population in Spain	overweight	Overweight and obesity	89	7.77	2.7
EGAN00001617423	EGAN00001617423	Genomic DNA extracted from peripheral white blood cells using two kits, the Qiamp DNA Investigator Kit for coagulated samples and Qiamp DNA Mini & Blood Mini Kit for non-coagulated samples (QIAgen Systems, Inc., Valencia, CA, USA).	Human	blood	NA	female	Iberian Population in Spain	obese	Overweight and obesity	86	4.34	2.84
EGAN00001617424	EGAN00001617424	Genomic DNA extracted from peripheral white blood cells using two kits, the Qiamp DNA Investigator Kit for coagulated samples and Qiamp DNA Mini & Blood Mini Kit for non-coagulated samples (QIAgen Systems, Inc., Valencia, CA, USA).	Human	blood	NA	female	Iberian Population in Spain	obese	Overweight and obesity	97	NA	2.54
EGAN00001617425	EGAN00001617425	Genomic DNA extracted from peripheral white blood cells using two kits, the Qiamp DNA Investigator Kit for coagulated samples and Qiamp DNA Mini & Blood Mini Kit for non-coagulated samples (QIAgen Systems, Inc., Valencia, CA, USA).	Human	blood	NA	female	Iberian Population in Spain	obese	Overweight and obesity	91	5.96	2
EGAN00001617426	EGAN00001617426	Genomic DNA extracted from peripheral white blood cells using two kits, the Qiamp DNA Investigator Kit for coagulated samples and Qiamp DNA Mini & Blood Mini Kit for non-coagulated samples (QIAgen Systems, Inc., Valencia, CA, USA).	Human	blood	NA	female	Iberian Population in Spain	overweight	Overweight and obesity	83	3.4	0.74
EGAN00001617427	EGAN00001617427	Genomic DNA extracted from peripheral white blood cells using two kits, the Qiamp DNA Investigator Kit for coagulated samples and Qiamp DNA Mini & Blood Mini Kit for non-coagulated samples (QIAgen Systems, Inc., Valencia, CA, USA).	Human	blood	NA	female	Iberian Population in Spain	overweight	Overweight and obesity	97	4.02	1.31
EGAN00001617428	EGAN00001617428	Genomic DNA extracted from peripheral white blood cells using two kits, the Qiamp DNA Investigator Kit for coagulated samples and Qiamp DNA Mini & Blood Mini Kit for non-coagulated samples (QIAgen Systems, Inc., Valencia, CA, USA).	Human	blood	NA	female	Iberian Population in Spain	obese	Overweight and obesity	88	3.34	2.5
EGAN00001617429	EGAN00001617429	Genomic DNA extracted from peripheral white blood cells using two kits, the Qiamp DNA Investigator Kit for coagulated samples and Qiamp DNA Mini & Blood Mini Kit for non-coagulated samples (QIAgen Systems, Inc., Valencia, CA, USA).	Human	blood	NA	female	Iberian Population in Spain	overweight	Overweight and obesity	95	5.26	1.73
EGAN00001617430	EGAN00001617430	Genomic DNA extracted from peripheral white blood cells using two kits, the Qiamp DNA Investigator Kit for coagulated samples and Qiamp DNA Mini & Blood Mini Kit for non-coagulated samples (QIAgen Systems, Inc., Valencia, CA, USA).	Human	blood	NA	female	Iberian Population in Spain	obese	Overweight and obesity	91	3.05	2.6
EGAN00001617431	EGAN00001617431	Genomic DNA extracted from peripheral white blood cells using two kits, the Qiamp DNA Investigator Kit for coagulated samples and Qiamp DNA Mini & Blood Mini Kit for non-coagulated samples (QIAgen Systems, Inc., Valencia, CA, USA).	Human	blood	NA	female	Iberian Population in Spain	overweight	Overweight and obesity	88	7.35	2.35
EGAN00001617432	EGAN00001617432	Genomic DNA extracted from peripheral white blood cells using two kits, the Qiamp DNA Investigator Kit for coagulated samples and Qiamp DNA Mini & Blood Mini Kit for non-coagulated samples (QIAgen Systems, Inc., Valencia, CA, USA).	Human	blood	NA	female	Iberian Population in Spain	overweight	Overweight and obesity	83	2.27	0.87
EGAN00001617433	EGAN00001617433	Genomic DNA extracted from peripheral white blood cells using two kits, the Qiamp DNA Investigator Kit for coagulated samples and Qiamp DNA Mini & Blood Mini Kit for non-coagulated samples (QIAgen Systems, Inc., Valencia, CA, USA).	Human	blood	NA	female	Iberian Population in Spain	obese	Overweight and obesity	95	5.93	2.39
EGAN00001617434	EGAN00001617434	Genomic DNA extracted from peripheral white blood cells using two kits, the Qiamp DNA Investigator Kit for coagulated samples and Qiamp DNA Mini & Blood Mini Kit for non-coagulated samples (QIAgen Systems, Inc., Valencia, CA, USA).	Human	blood	NA	female	Iberian Population in Spain	obese	Overweight and obesity	85	1.9	1.87
EGAN00001617435	EGAN00001617435	Genomic DNA extracted from peripheral white blood cells using two kits, the Qiamp DNA Investigator Kit for coagulated samples and Qiamp DNA Mini & Blood Mini Kit for non-coagulated samples (QIAgen Systems, Inc., Valencia, CA, USA).	Human	blood	NA	female	Iberian Population in Spain	overweight	Overweight and obesity	100	7.06	0.98
EGAN00001617436	EGAN00001617436	Genomic DNA extracted from peripheral white blood cells using two kits, the Qiamp DNA Investigator Kit for coagulated samples and Qiamp DNA Mini & Blood Mini Kit for non-coagulated samples (QIAgen Systems, Inc., Valencia, CA, USA).	Human	blood	NA	female	Iberian Population in Spain	overweight	Overweight and obesity	89	4.12	1.39
EGAN00001617437	EGAN00001617437	Genomic DNA extracted from peripheral white blood cells using two kits, the Qiamp DNA Investigator Kit for coagulated samples and Qiamp DNA Mini & Blood Mini Kit for non-coagulated samples (QIAgen Systems, Inc., Valencia, CA, USA).	Human	blood	NA	female	Iberian Population in Spain	obese	Overweight and obesity	99	4.57	2.54
EGAN00001617438	EGAN00001617438	Genomic DNA extracted from peripheral white blood cells using two kits, the Qiamp DNA Investigator Kit for coagulated samples and Qiamp DNA Mini & Blood Mini Kit for non-coagulated samples (QIAgen Systems, Inc., Valencia, CA, USA).	Human	blood	NA	female	Iberian Population in Spain	obese	Overweight and obesity	85	4.7	2.45
EGAN00001617439	EGAN00001617439	Genomic DNA extracted from peripheral white blood cells using two kits, the Qiamp DNA Investigator Kit for coagulated samples and Qiamp DNA Mini & Blood Mini Kit for non-coagulated samples (QIAgen Systems, Inc., Valencia, CA, USA).	Human	blood	NA	female	Iberian Population in Spain	obese	Overweight and obesity	84	4	2.8
EGAN00001617440	EGAN00001617440	Genomic DNA extracted from peripheral white blood cells using two kits, the Qiamp DNA Investigator Kit for coagulated samples and Qiamp DNA Mini & Blood Mini Kit for non-coagulated samples (QIAgen Systems, Inc., Valencia, CA, USA).	Human	blood	NA	female	Iberian Population in Spain	obese	Overweight and obesity	89	5.75	2.58
EGAN00001617441	EGAN00001617441	Genomic DNA extracted from peripheral white blood cells using two kits, the Qiamp DNA Investigator Kit for coagulated samples and Qiamp DNA Mini & Blood Mini Kit for non-coagulated samples (QIAgen Systems, Inc., Valencia, CA, USA).	Human	blood	NA	female	Iberian Population in Spain	obese	Overweight and obesity	90	9.95	3.36
EGAN00001617442	EGAN00001617442	Genomic DNA extracted from peripheral white blood cells using two kits, the Qiamp DNA Investigator Kit for coagulated samples and Qiamp DNA Mini & Blood Mini Kit for non-coagulated samples (QIAgen Systems, Inc., Valencia, CA, USA).	Human	blood	NA	female	Iberian Population in Spain	overweight	Overweight and obesity	89	3.18	1.1
EGAN00001617443	EGAN00001617443	Genomic DNA extracted from peripheral white blood cells using two kits, the Qiamp DNA Investigator Kit for coagulated samples and Qiamp DNA Mini & Blood Mini Kit for non-coagulated samples (QIAgen Systems, Inc., Valencia, CA, USA).	Human	blood	NA	female	Iberian Population in Spain	obese	Overweight and obesity	88	3.75	2.55
EGAN00001617444	EGAN00001617444	Genomic DNA extracted from peripheral white blood cells using two kits, the Qiamp DNA Investigator Kit for coagulated samples and Qiamp DNA Mini & Blood Mini Kit for non-coagulated samples (QIAgen Systems, Inc., Valencia, CA, USA).	Human	blood	NA	female	Iberian Population in Spain	overweight	Overweight and obesity	91	4.16	1.65
EGAN00001617445	EGAN00001617445	Genomic DNA extracted from peripheral white blood cells using two kits, the Qiamp DNA Investigator Kit for coagulated samples and Qiamp DNA Mini & Blood Mini Kit for non-coagulated samples (QIAgen Systems, Inc., Valencia, CA, USA).	Human	blood	NA	female	Iberian Population in Spain	overweight	Overweight and obesity	93	5.26	0.98
EGAN00001617446	EGAN00001617446	Genomic DNA extracted from peripheral white blood cells using two kits, the Qiamp DNA Investigator Kit for coagulated samples and Qiamp DNA Mini & Blood Mini Kit for non-coagulated samples (QIAgen Systems, Inc., Valencia, CA, USA).	Human	blood	NA	female	Iberian Population in Spain	overweight	Overweight and obesity	101	5.04	2.12
EGAN00001617447	EGAN00001617447	Genomic DNA extracted from peripheral white blood cells using two kits, the Qiamp DNA Investigator Kit for coagulated samples and Qiamp DNA Mini & Blood Mini Kit for non-coagulated samples (QIAgen Systems, Inc., Valencia, CA, USA).	Human	blood	NA	female	Iberian Population in Spain	overweight	Overweight and obesity	86	3.64	1.45
EGAN00001617448	EGAN00001617448	Genomic DNA extracted from peripheral white blood cells using two kits, the Qiamp DNA Investigator Kit for coagulated samples and Qiamp DNA Mini & Blood Mini Kit for non-coagulated samples (QIAgen Systems, Inc., Valencia, CA, USA).	Human	blood	NA	female	Iberian Population in Spain	obese	Overweight and obesity	93	8.26	2.45
EGAN00001617449	EGAN00001617449	Genomic DNA extracted from peripheral white blood cells using two kits, the Qiamp DNA Investigator Kit for coagulated samples and Qiamp DNA Mini & Blood Mini Kit for non-coagulated samples (QIAgen Systems, Inc., Valencia, CA, USA).	Human	blood	NA	female	Iberian Population in Spain	overweight	Overweight and obesity	83	2.1	0.63
EGAN00001617450	EGAN00001617450	Genomic DNA extracted from peripheral white blood cells using two kits, the Qiamp DNA Investigator Kit for coagulated samples and Qiamp DNA Mini & Blood Mini Kit for non-coagulated samples (QIAgen Systems, Inc., Valencia, CA, USA).	Human	blood	NA	female	Iberian Population in Spain	overweight	Overweight and obesity	91	3.2	0.83
EGAN00001617451	EGAN00001617451	Genomic DNA extracted from peripheral white blood cells using two kits, the Qiamp DNA Investigator Kit for coagulated samples and Qiamp DNA Mini & Blood Mini Kit for non-coagulated samples (QIAgen Systems, Inc., Valencia, CA, USA).	Human	blood	NA	female	Iberian Population in Spain	overweight	Overweight and obesity	82	2,571	1.82
EGAN00001617452	EGAN00001617452	Genomic DNA extracted from peripheral white blood cells using two kits, the Qiamp DNA Investigator Kit for coagulated samples and Qiamp DNA Mini & Blood Mini Kit for non-coagulated samples (QIAgen Systems, Inc., Valencia, CA, USA).	Human	blood	NA	female	Iberian Population in Spain	obese	Overweight and obesity	104	NA	2.63
EGAN00001617453	EGAN00001617453	Genomic DNA extracted from peripheral white blood cells using two kits, the Qiamp DNA Investigator Kit for coagulated samples and Qiamp DNA Mini & Blood Mini Kit for non-coagulated samples (QIAgen Systems, Inc., Valencia, CA, USA).	Human	blood	NA	female	Iberian Population in Spain	overweight	Overweight and obesity	72	1,244	0.97
EGAN00001617454	EGAN00001617454	Genomic DNA extracted from peripheral white blood cells using two kits, the Qiamp DNA Investigator Kit for coagulated samples and Qiamp DNA Mini & Blood Mini Kit for non-coagulated samples (QIAgen Systems, Inc., Valencia, CA, USA).	Human	blood	NA	female	Iberian Population in Spain	obese	Overweight and obesity	83	2,336	2.55
EGAN00001617455	EGAN00001617455	Genomic DNA extracted from peripheral white blood cells using two kits, the Qiamp DNA Investigator Kit for coagulated samples and Qiamp DNA Mini & Blood Mini Kit for non-coagulated samples (QIAgen Systems, Inc., Valencia, CA, USA).	Human	blood	NA	female	Iberian Population in Spain	obese	Overweight and obesity	93	3.85	3.05
EGAN00001617456	EGAN00001617456	Genomic DNA extracted from peripheral white blood cells using two kits, the Qiamp DNA Investigator Kit for coagulated samples and Qiamp DNA Mini & Blood Mini Kit for non-coagulated samples (QIAgen Systems, Inc., Valencia, CA, USA).	Human	blood	NA	female	Iberian Population in Spain	obese	Overweight and obesity	89	3.03	2.99
EGAN00001617457	EGAN00001617457	Genomic DNA extracted from peripheral white blood cells using two kits, the Qiamp DNA Investigator Kit for coagulated samples and Qiamp DNA Mini & Blood Mini Kit for non-coagulated samples (QIAgen Systems, Inc., Valencia, CA, USA).	Human	blood	NA	female	Iberian Population in Spain	obese	Overweight and obesity	77	2.81	3.81
EGAN00001617458	EGAN00001617458	Genomic DNA extracted from peripheral white blood cells using two kits, the Qiamp DNA Investigator Kit for coagulated samples and Qiamp DNA Mini & Blood Mini Kit for non-coagulated samples (QIAgen Systems, Inc., Valencia, CA, USA).	Human	blood	NA	female	Iberian Population in Spain	obese	Overweight and obesity	81	2.28	3.59
EGAN00001617459	EGAN00001617459	Genomic DNA extracted from peripheral white blood cells using two kits, the Qiamp DNA Investigator Kit for coagulated samples and Qiamp DNA Mini & Blood Mini Kit for non-coagulated samples (QIAgen Systems, Inc., Valencia, CA, USA).	Human	blood	NA	female	Iberian Population in Spain	obese	Overweight and obesity	93	2.2	3.7
EGAN00001617460	EGAN00001617460	Genomic DNA extracted from peripheral white blood cells using two kits, the Qiamp DNA Investigator Kit for coagulated samples and Qiamp DNA Mini & Blood Mini Kit for non-coagulated samples (QIAgen Systems, Inc., Valencia, CA, USA).	Human	blood	NA	female	Iberian Population in Spain	obese	Overweight and obesity	89	3.07	2.21
EGAN00001617461	EGAN00001617461	Genomic DNA extracted from peripheral white blood cells using two kits, the Qiamp DNA Investigator Kit for coagulated samples and Qiamp DNA Mini & Blood Mini Kit for non-coagulated samples (QIAgen Systems, Inc., Valencia, CA, USA).	Human	blood	NA	female	Iberian Population in Spain	obese	Overweight and obesity	76	3.66	3.64
EGAN00001617462	EGAN00001617462	Genomic DNA extracted from peripheral white blood cells using two kits, the Qiamp DNA Investigator Kit for coagulated samples and Qiamp DNA Mini & Blood Mini Kit for non-coagulated samples (QIAgen Systems, Inc., Valencia, CA, USA).	Human	blood	NA	female	Iberian Population in Spain	obese	Overweight and obesity	88	3.93	3.32
EGAN00001617463	EGAN00001617463	Genomic DNA extracted from peripheral white blood cells using two kits, the Qiamp DNA Investigator Kit for coagulated samples and Qiamp DNA Mini & Blood Mini Kit for non-coagulated samples (QIAgen Systems, Inc., Valencia, CA, USA).	Human	blood	NA	female	Iberian Population in Spain	obese	Overweight and obesity	72	1.6	5.49
EGAN00001617464	EGAN00001617464	Genomic DNA extracted from peripheral white blood cells using two kits, the Qiamp DNA Investigator Kit for coagulated samples and Qiamp DNA Mini & Blood Mini Kit for non-coagulated samples (QIAgen Systems, Inc., Valencia, CA, USA).	Human	blood	NA	female	Iberian Population in Spain	obese	Overweight and obesity	91	3.97	3.96
EGAN00001617465	EGAN00001617465	Genomic DNA extracted from peripheral white blood cells using two kits, the Qiamp DNA Investigator Kit for coagulated samples and Qiamp DNA Mini & Blood Mini Kit for non-coagulated samples (QIAgen Systems, Inc., Valencia, CA, USA).	Human	blood	NA	female	Iberian Population in Spain	obese	Overweight and obesity	82	2.65	2.06
EGAN00001617466	EGAN00001617466	Genomic DNA extracted from peripheral white blood cells using two kits, the Qiamp DNA Investigator Kit for coagulated samples and Qiamp DNA Mini & Blood Mini Kit for non-coagulated samples (QIAgen Systems, Inc., Valencia, CA, USA).	Human	blood	NA	female	Iberian Population in Spain	obese	Overweight and obesity	77	1.54	2.87
EGAN00001617467	EGAN00001617467	Genomic DNA extracted from peripheral white blood cells using two kits, the Qiamp DNA Investigator Kit for coagulated samples and Qiamp DNA Mini & Blood Mini Kit for non-coagulated samples (QIAgen Systems, Inc., Valencia, CA, USA).	Human	blood	NA	female	Iberian Population in Spain	obese	Overweight and obesity	86	4.45	3.66
EGAN00001617468	EGAN00001617468	Genomic DNA extracted from peripheral white blood cells using two kits, the Qiamp DNA Investigator Kit for coagulated samples and Qiamp DNA Mini & Blood Mini Kit for non-coagulated samples (QIAgen Systems, Inc., Valencia, CA, USA).	Human	blood	NA	female	Iberian Population in Spain	obese	Overweight and obesity	79	3.29	4.81
EGAN00001617469	EGAN00001617469	Genomic DNA extracted from peripheral white blood cells using two kits, the Qiamp DNA Investigator Kit for coagulated samples and Qiamp DNA Mini & Blood Mini Kit for non-coagulated samples (QIAgen Systems, Inc., Valencia, CA, USA).	Human	blood	NA	female	Iberian Population in Spain	obese	Overweight and obesity	81	1	1.87
EGAN00001617470	EGAN00001617470	Genomic DNA extracted from peripheral white blood cells using two kits, the Qiamp DNA Investigator Kit for coagulated samples and Qiamp DNA Mini & Blood Mini Kit for non-coagulated samples (QIAgen Systems, Inc., Valencia, CA, USA).	Human	blood	NA	female	Iberian Population in Spain	obese	Overweight and obesity	72	1.72	2.93
EGAN00001617471	EGAN00001617471	Genomic DNA extracted from peripheral white blood cells using two kits, the Qiamp DNA Investigator Kit for coagulated samples and Qiamp DNA Mini & Blood Mini Kit for non-coagulated samples (QIAgen Systems, Inc., Valencia, CA, USA).	Human	blood	NA	female	Iberian Population in Spain	obese	Overweight and obesity	86	5.3	4.01
EGAN00001617472	EGAN00001617472	Genomic DNA extracted from peripheral white blood cells using two kits, the Qiamp DNA Investigator Kit for coagulated samples and Qiamp DNA Mini & Blood Mini Kit for non-coagulated samples (QIAgen Systems, Inc., Valencia, CA, USA).	Human	blood	NA	female	Iberian Population in Spain	obese	Overweight and obesity	94	2.5	3.97
EGAN00001617473	EGAN00001617473	Genomic DNA extracted from peripheral white blood cells using two kits, the Qiamp DNA Investigator Kit for coagulated samples and Qiamp DNA Mini & Blood Mini Kit for non-coagulated samples (QIAgen Systems, Inc., Valencia, CA, USA).	Human	blood	NA	female	Iberian Population in Spain	obese	Overweight and obesity	95	7.71	3.25
EGAN00001617474	EGAN00001617474	Genomic DNA extracted from peripheral white blood cells using two kits, the Qiamp DNA Investigator Kit for coagulated samples and Qiamp DNA Mini & Blood Mini Kit for non-coagulated samples (QIAgen Systems, Inc., Valencia, CA, USA).	Human	blood	NA	female	Iberian Population in Spain	obese	Overweight and obesity	92	5.42	2.91
EGAN00001617475	EGAN00001617475	Genomic DNA extracted from peripheral white blood cells using two kits, the Qiamp DNA Investigator Kit for coagulated samples and Qiamp DNA Mini & Blood Mini Kit for non-coagulated samples (QIAgen Systems, Inc., Valencia, CA, USA).	Human	blood	NA	female	Iberian Population in Spain	obese	Overweight and obesity	NA	NA	NA
EGAN00001617476	EGAN00001617476	Genomic DNA extracted from peripheral white blood cells using two kits, the Qiamp DNA Investigator Kit for coagulated samples and Qiamp DNA Mini & Blood Mini Kit for non-coagulated samples (QIAgen Systems, Inc., Valencia, CA, USA).	Human	blood	NA	female	Iberian Population in Spain	obese	Overweight and obesity	89	3.51	4.25
EGAN00001617477	EGAN00001617477	Genomic DNA extracted from peripheral white blood cells using two kits, the Qiamp DNA Investigator Kit for coagulated samples and Qiamp DNA Mini & Blood Mini Kit for non-coagulated samples (QIAgen Systems, Inc., Valencia, CA, USA).	Human	blood	NA	female	Iberian Population in Spain	obese	Overweight and obesity	102	6.57	NA
EGAN00001617478	EGAN00001617478	Genomic DNA extracted from peripheral white blood cells using two kits, the Qiamp DNA Investigator Kit for coagulated samples and Qiamp DNA Mini & Blood Mini Kit for non-coagulated samples (QIAgen Systems, Inc., Valencia, CA, USA).	Human	blood	NA	female	Iberian Population in Spain	obese	Overweight and obesity	86	4.16	NA
EGAN00001617479	EGAN00001617479	Genomic DNA extracted from peripheral white blood cells using two kits, the Qiamp DNA Investigator Kit for coagulated samples and Qiamp DNA Mini & Blood Mini Kit for non-coagulated samples (QIAgen Systems, Inc., Valencia, CA, USA).	Human	blood	NA	female	Iberian Population in Spain	obese	Overweight and obesity	86	6.17	2.57
EGAN00001617480	EGAN00001617480	Genomic DNA extracted from peripheral white blood cells using two kits, the Qiamp DNA Investigator Kit for coagulated samples and Qiamp DNA Mini & Blood Mini Kit for non-coagulated samples (QIAgen Systems, Inc., Valencia, CA, USA).	Human	blood	NA	female	Iberian Population in Spain	obese	Overweight and obesity	86	2.55	1.59
EGAN00001617481	EGAN00001617481	Genomic DNA extracted from peripheral white blood cells using two kits, the Qiamp DNA Investigator Kit for coagulated samples and Qiamp DNA Mini & Blood Mini Kit for non-coagulated samples (QIAgen Systems, Inc., Valencia, CA, USA).	Human	blood	NA	female	Iberian Population in Spain	obese	Overweight and obesity	88	11.03	NA
EGAN00001617482	EGAN00001617482	Genomic DNA extracted from peripheral white blood cells using two kits, the Qiamp DNA Investigator Kit for coagulated samples and Qiamp DNA Mini & Blood Mini Kit for non-coagulated samples (QIAgen Systems, Inc., Valencia, CA, USA).	Human	blood	NA	female	Iberian Population in Spain	obese	Overweight and obesity	89	6.28	2.88
EGAN00001617483	EGAN00001617483	Genomic DNA extracted from peripheral white blood cells using two kits, the Qiamp DNA Investigator Kit for coagulated samples and Qiamp DNA Mini & Blood Mini Kit for non-coagulated samples (QIAgen Systems, Inc., Valencia, CA, USA).	Human	blood	NA	female	Iberian Population in Spain	obese	Overweight and obesity	82	6.25	4.61
EGAN00001617484	EGAN00001617484	Genomic DNA extracted from peripheral white blood cells using two kits, the Qiamp DNA Investigator Kit for coagulated samples and Qiamp DNA Mini & Blood Mini Kit for non-coagulated samples (QIAgen Systems, Inc., Valencia, CA, USA).	Human	blood	NA	female	Iberian Population in Spain	obese	Overweight and obesity	91	2.99	1.97
EGAN00001617485	EGAN00001617485	Genomic DNA extracted from peripheral white blood cells using two kits, the Qiamp DNA Investigator Kit for coagulated samples and Qiamp DNA Mini & Blood Mini Kit for non-coagulated samples (QIAgen Systems, Inc., Valencia, CA, USA).	Human	blood	NA	female	Iberian Population in Spain	obese	Overweight and obesity	97	7.25	2.88
EGAN00001617486	EGAN00001617486	Genomic DNA extracted from peripheral white blood cells using two kits, the Qiamp DNA Investigator Kit for coagulated samples and Qiamp DNA Mini & Blood Mini Kit for non-coagulated samples (QIAgen Systems, Inc., Valencia, CA, USA).	Human	blood	NA	female	Iberian Population in Spain	obese	Overweight and obesity	81	1.34	1.4
EGAN00001617487	EGAN00001617487	Genomic DNA extracted from peripheral white blood cells using two kits, the Qiamp DNA Investigator Kit for coagulated samples and Qiamp DNA Mini & Blood Mini Kit for non-coagulated samples (QIAgen Systems, Inc., Valencia, CA, USA).	Human	blood	NA	female	Iberian Population in Spain	obese	Overweight and obesity	75	1.05	2.86
EGAN00001617488	EGAN00001617488	Genomic DNA extracted from peripheral white blood cells using two kits, the Qiamp DNA Investigator Kit for coagulated samples and Qiamp DNA Mini & Blood Mini Kit for non-coagulated samples (QIAgen Systems, Inc., Valencia, CA, USA).	Human	blood	NA	female	Iberian Population in Spain	obese	Overweight and obesity	85	1.89	1.76
EGAN00001617489	EGAN00001617489	Genomic DNA extracted from peripheral white blood cells using two kits, the Qiamp DNA Investigator Kit for coagulated samples and Qiamp DNA Mini & Blood Mini Kit for non-coagulated samples (QIAgen Systems, Inc., Valencia, CA, USA).	Human	blood	NA	female	Iberian Population in Spain	obese	Overweight and obesity	80	NA	1.81
EGAN00001617490	EGAN00001617490	Genomic DNA extracted from peripheral white blood cells using two kits, the Qiamp DNA Investigator Kit for coagulated samples and Qiamp DNA Mini & Blood Mini Kit for non-coagulated samples (QIAgen Systems, Inc., Valencia, CA, USA).	Human	blood	NA	female	Iberian Population in Spain	obese	Overweight and obesity	78	1.67	2.63
EGAN00001617491	EGAN00001617491	Genomic DNA extracted from peripheral white blood cells using two kits, the Qiamp DNA Investigator Kit for coagulated samples and Qiamp DNA Mini & Blood Mini Kit for non-coagulated samples (QIAgen Systems, Inc., Valencia, CA, USA).	Human	blood	NA	female	Iberian Population in Spain	obese	Overweight and obesity	77	4.33	4.65
EGAN00001617492	EGAN00001617492	Genomic DNA extracted from peripheral white blood cells using two kits, the Qiamp DNA Investigator Kit for coagulated samples and Qiamp DNA Mini & Blood Mini Kit for non-coagulated samples (QIAgen Systems, Inc., Valencia, CA, USA).	Human	blood	NA	female	Iberian Population in Spain	obese	Overweight and obesity	85	2.47	2.13
EGAN00001617493	EGAN00001617493	Genomic DNA extracted from peripheral white blood cells using two kits, the Qiamp DNA Investigator Kit for coagulated samples and Qiamp DNA Mini & Blood Mini Kit for non-coagulated samples (QIAgen Systems, Inc., Valencia, CA, USA).	Human	blood	NA	female	Iberian Population in Spain	obese	Overweight and obesity	76	6.51	2.82
EGAN00001617494	EGAN00001617494	Genomic DNA extracted from peripheral white blood cells using two kits, the Qiamp DNA Investigator Kit for coagulated samples and Qiamp DNA Mini & Blood Mini Kit for non-coagulated samples (QIAgen Systems, Inc., Valencia, CA, USA).	Human	blood	NA	female	Iberian Population in Spain	obese	Overweight and obesity	79	2.2	2.29
EGAN00001617495	EGAN00001617495	Genomic DNA extracted from peripheral white blood cells using two kits, the Qiamp DNA Investigator Kit for coagulated samples and Qiamp DNA Mini & Blood Mini Kit for non-coagulated samples (QIAgen Systems, Inc., Valencia, CA, USA).	Human	blood	NA	female	Iberian Population in Spain	obese	Overweight and obesity	78	0.6	1.54
EGAN00001617496	EGAN00001617496	Genomic DNA extracted from peripheral white blood cells using two kits, the Qiamp DNA Investigator Kit for coagulated samples and Qiamp DNA Mini & Blood Mini Kit for non-coagulated samples (QIAgen Systems, Inc., Valencia, CA, USA).	Human	blood	NA	female	Iberian Population in Spain	obese	Overweight and obesity	96	1.85	3.16
EGAN00001617497	EGAN00001617497	Genomic DNA extracted from peripheral white blood cells using two kits, the Qiamp DNA Investigator Kit for coagulated samples and Qiamp DNA Mini & Blood Mini Kit for non-coagulated samples (QIAgen Systems, Inc., Valencia, CA, USA).	Human	blood	NA	female	Iberian Population in Spain	obese	Overweight and obesity	79	1.54	2.59
EGAN00001617498	EGAN00001617498	Genomic DNA extracted from peripheral white blood cells using two kits, the Qiamp DNA Investigator Kit for coagulated samples and Qiamp DNA Mini & Blood Mini Kit for non-coagulated samples (QIAgen Systems, Inc., Valencia, CA, USA).	Human	blood	NA	female	Iberian Population in Spain	obese	Overweight and obesity	82	NA	3.67
EGAN00001617499	EGAN00001617499	Genomic DNA extracted from peripheral white blood cells using two kits, the Qiamp DNA Investigator Kit for coagulated samples and Qiamp DNA Mini & Blood Mini Kit for non-coagulated samples (QIAgen Systems, Inc., Valencia, CA, USA).	Human	blood	NA	female	Iberian Population in Spain	obese	Overweight and obesity	82	3.46	2.36
EGAN00001617500	EGAN00001617500	Genomic DNA extracted from peripheral white blood cells using two kits, the Qiamp DNA Investigator Kit for coagulated samples and Qiamp DNA Mini & Blood Mini Kit for non-coagulated samples (QIAgen Systems, Inc., Valencia, CA, USA).	Human	blood	NA	female	Iberian Population in Spain	obese	Overweight and obesity	80	3.99	2.44
EGAN00001617501	EGAN00001617501	Genomic DNA extracted from peripheral white blood cells using two kits, the Qiamp DNA Investigator Kit for coagulated samples and Qiamp DNA Mini & Blood Mini Kit for non-coagulated samples (QIAgen Systems, Inc., Valencia, CA, USA).	Human	blood	NA	female	Iberian Population in Spain	obese	Overweight and obesity	89	2.83	2.5
EGAN00001617502	EGAN00001617502	Genomic DNA extracted from peripheral white blood cells using two kits, the Qiamp DNA Investigator Kit for coagulated samples and Qiamp DNA Mini & Blood Mini Kit for non-coagulated samples (QIAgen Systems, Inc., Valencia, CA, USA).	Human	blood	NA	female	Iberian Population in Spain	obese	Overweight and obesity	74	3.38	2.52
EGAN00001617503	EGAN00001617503	Genomic DNA extracted from peripheral white blood cells using two kits, the Qiamp DNA Investigator Kit for coagulated samples and Qiamp DNA Mini & Blood Mini Kit for non-coagulated samples (QIAgen Systems, Inc., Valencia, CA, USA).	Human	blood	NA	female	Iberian Population in Spain	obese	Overweight and obesity	89	6.91	4.55
EGAN00001617504	EGAN00001617504	Genomic DNA extracted from peripheral white blood cells using two kits, the Qiamp DNA Investigator Kit for coagulated samples and Qiamp DNA Mini & Blood Mini Kit for non-coagulated samples (QIAgen Systems, Inc., Valencia, CA, USA).	Human	blood	NA	female	Iberian Population in Spain	obese	Overweight and obesity	93	4.43	4.87
EGAN00001617505	EGAN00001617505	Genomic DNA extracted from peripheral white blood cells using two kits, the Qiamp DNA Investigator Kit for coagulated samples and Qiamp DNA Mini & Blood Mini Kit for non-coagulated samples (QIAgen Systems, Inc., Valencia, CA, USA).	Human	blood	NA	female	Iberian Population in Spain	obese	Overweight and obesity	84	3.96	3.06
EGAN00001617506	EGAN00001617506	Genomic DNA extracted from peripheral white blood cells using two kits, the Qiamp DNA Investigator Kit for coagulated samples and Qiamp DNA Mini & Blood Mini Kit for non-coagulated samples (QIAgen Systems, Inc., Valencia, CA, USA).	Human	blood	NA	female	Iberian Population in Spain	obese	Overweight and obesity	83	2.98	2.04
EGAN00001617507	EGAN00001617507	Genomic DNA extracted from peripheral white blood cells using two kits, the Qiamp DNA Investigator Kit for coagulated samples and Qiamp DNA Mini & Blood Mini Kit for non-coagulated samples (QIAgen Systems, Inc., Valencia, CA, USA).	Human	blood	NA	female	Iberian Population in Spain	obese	Overweight and obesity	77	4.14	2.72
EGAN00001617508	EGAN00001617508	Genomic DNA extracted from peripheral white blood cells using two kits, the Qiamp DNA Investigator Kit for coagulated samples and Qiamp DNA Mini & Blood Mini Kit for non-coagulated samples (QIAgen Systems, Inc., Valencia, CA, USA).	Human	blood	NA	female	Iberian Population in Spain	obese	Overweight and obesity	80	6.91	2.4
EGAN00001617509	EGAN00001617509	Genomic DNA extracted from peripheral white blood cells using two kits, the Qiamp DNA Investigator Kit for coagulated samples and Qiamp DNA Mini & Blood Mini Kit for non-coagulated samples (QIAgen Systems, Inc., Valencia, CA, USA).	Human	blood	NA	female	Iberian Population in Spain	obese	Overweight and obesity	82	2.5	2.53
EGAN00001617510	EGAN00001617510	Genomic DNA extracted from peripheral white blood cells using two kits, the Qiamp DNA Investigator Kit for coagulated samples and Qiamp DNA Mini & Blood Mini Kit for non-coagulated samples (QIAgen Systems, Inc., Valencia, CA, USA).	Human	blood	NA	female	Iberian Population in Spain	obese	Overweight and obesity	84	3.11	2.78
EGAN00001617511	EGAN00001617511	Genomic DNA extracted from peripheral white blood cells using two kits, the Qiamp DNA Investigator Kit for coagulated samples and Qiamp DNA Mini & Blood Mini Kit for non-coagulated samples (QIAgen Systems, Inc., Valencia, CA, USA).	Human	blood	NA	female	Iberian Population in Spain	obese	Overweight and obesity	86	9.66	2.85
EGAN00001617512	EGAN00001617512	Genomic DNA extracted from peripheral white blood cells using two kits, the Qiamp DNA Investigator Kit for coagulated samples and Qiamp DNA Mini & Blood Mini Kit for non-coagulated samples (QIAgen Systems, Inc., Valencia, CA, USA).	Human	blood	NA	female	Iberian Population in Spain	obese	Overweight and obesity	86	7.46	2.66
EGAN00001617513	EGAN00001617513	Genomic DNA extracted from peripheral white blood cells using two kits, the Qiamp DNA Investigator Kit for coagulated samples and Qiamp DNA Mini & Blood Mini Kit for non-coagulated samples (QIAgen Systems, Inc., Valencia, CA, USA).	Human	blood	NA	female	Iberian Population in Spain	obese	Overweight and obesity	94	4.72	2.95
EGAN00001617514	EGAN00001617514	Genomic DNA extracted from peripheral white blood cells using two kits, the Qiamp DNA Investigator Kit for coagulated samples and Qiamp DNA Mini & Blood Mini Kit for non-coagulated samples (QIAgen Systems, Inc., Valencia, CA, USA).	Human	blood	NA	female	Iberian Population in Spain	obese	Overweight and obesity	99	5.68	3.39
EGAN00001617515	EGAN00001617515	Genomic DNA extracted from peripheral white blood cells using two kits, the Qiamp DNA Investigator Kit for coagulated samples and Qiamp DNA Mini & Blood Mini Kit for non-coagulated samples (QIAgen Systems, Inc., Valencia, CA, USA).	Human	blood	NA	female	Iberian Population in Spain	obese	Overweight and obesity	85	3.51	3.34
EGAN00001617516	EGAN00001617516	Genomic DNA extracted from peripheral white blood cells using two kits, the Qiamp DNA Investigator Kit for coagulated samples and Qiamp DNA Mini & Blood Mini Kit for non-coagulated samples (QIAgen Systems, Inc., Valencia, CA, USA).	Human	blood	NA	female	Iberian Population in Spain	obese	Overweight and obesity	83	5.18	3.17
EGAN00001617517	EGAN00001617517	Genomic DNA extracted from peripheral white blood cells using two kits, the Qiamp DNA Investigator Kit for coagulated samples and Qiamp DNA Mini & Blood Mini Kit for non-coagulated samples (QIAgen Systems, Inc., Valencia, CA, USA).	Human	blood	NA	female	Iberian Population in Spain	obese	Overweight and obesity	93	3.95	3.25
EGAN00001617518	EGAN00001617518	Genomic DNA extracted from peripheral white blood cells using two kits, the Qiamp DNA Investigator Kit for coagulated samples and Qiamp DNA Mini & Blood Mini Kit for non-coagulated samples (QIAgen Systems, Inc., Valencia, CA, USA).	Human	blood	NA	male	Iberian Population in Spain	overweight	Overweight and obesity	97	1.15	2.12
EGAN00001617519	EGAN00001617519	Genomic DNA extracted from peripheral white blood cells using two kits, the Qiamp DNA Investigator Kit for coagulated samples and Qiamp DNA Mini & Blood Mini Kit for non-coagulated samples (QIAgen Systems, Inc., Valencia, CA, USA).	Human	blood	NA	male	Iberian Population in Spain	obese	Overweight and obesity	84	2.18	3.5
EGAN00001617520	EGAN00001617520	Genomic DNA extracted from peripheral white blood cells using two kits, the Qiamp DNA Investigator Kit for coagulated samples and Qiamp DNA Mini & Blood Mini Kit for non-coagulated samples (QIAgen Systems, Inc., Valencia, CA, USA).	Human	blood	NA	male	Iberian Population in Spain	overweight	Overweight and obesity	91	2.25	0.77
EGAN00001617521	EGAN00001617521	Genomic DNA extracted from peripheral white blood cells using two kits, the Qiamp DNA Investigator Kit for coagulated samples and Qiamp DNA Mini & Blood Mini Kit for non-coagulated samples (QIAgen Systems, Inc., Valencia, CA, USA).	Human	blood	NA	male	Iberian Population in Spain	obese	Overweight and obesity	99	12.39	2.33
EGAN00001617522	EGAN00001617522	Genomic DNA extracted from peripheral white blood cells using two kits, the Qiamp DNA Investigator Kit for coagulated samples and Qiamp DNA Mini & Blood Mini Kit for non-coagulated samples (QIAgen Systems, Inc., Valencia, CA, USA).	Human	blood	NA	male	Iberian Population in Spain	overweight	Overweight and obesity	84	1.51	0.96
EGAN00001617523	EGAN00001617523	Genomic DNA extracted from peripheral white blood cells using two kits, the Qiamp DNA Investigator Kit for coagulated samples and Qiamp DNA Mini & Blood Mini Kit for non-coagulated samples (QIAgen Systems, Inc., Valencia, CA, USA).	Human	blood	NA	male	Iberian Population in Spain	obese	Overweight and obesity	84	1.64	2.61
EGAN00001617524	EGAN00001617524	Genomic DNA extracted from peripheral white blood cells using two kits, the Qiamp DNA Investigator Kit for coagulated samples and Qiamp DNA Mini & Blood Mini Kit for non-coagulated samples (QIAgen Systems, Inc., Valencia, CA, USA).	Human	blood	NA	male	Iberian Population in Spain	overweight	Overweight and obesity	91	1.69	1.17
EGAN00001617525	EGAN00001617525	Genomic DNA extracted from peripheral white blood cells using two kits, the Qiamp DNA Investigator Kit for coagulated samples and Qiamp DNA Mini & Blood Mini Kit for non-coagulated samples (QIAgen Systems, Inc., Valencia, CA, USA).	Human	blood	NA	male	Iberian Population in Spain	overweight	Overweight and obesity	91	3.48	0.99
EGAN00001617526	EGAN00001617526	Genomic DNA extracted from peripheral white blood cells using two kits, the Qiamp DNA Investigator Kit for coagulated samples and Qiamp DNA Mini & Blood Mini Kit for non-coagulated samples (QIAgen Systems, Inc., Valencia, CA, USA).	Human	blood	NA	male	Iberian Population in Spain	obese	Overweight and obesity	96	7.73	3.2
EGAN00001617527	EGAN00001617527	Genomic DNA extracted from peripheral white blood cells using two kits, the Qiamp DNA Investigator Kit for coagulated samples and Qiamp DNA Mini & Blood Mini Kit for non-coagulated samples (QIAgen Systems, Inc., Valencia, CA, USA).	Human	blood	NA	male	Iberian Population in Spain	obese	Overweight and obesity	85	1.81	3.41
EGAN00001617528	EGAN00001617528	Genomic DNA extracted from peripheral white blood cells using two kits, the Qiamp DNA Investigator Kit for coagulated samples and Qiamp DNA Mini & Blood Mini Kit for non-coagulated samples (QIAgen Systems, Inc., Valencia, CA, USA).	Human	blood	NA	male	Iberian Population in Spain	obese	Overweight and obesity	84	1.35	3.53
EGAN00001617529	EGAN00001617529	Genomic DNA extracted from peripheral white blood cells using two kits, the Qiamp DNA Investigator Kit for coagulated samples and Qiamp DNA Mini & Blood Mini Kit for non-coagulated samples (QIAgen Systems, Inc., Valencia, CA, USA).	Human	blood	NA	male	Iberian Population in Spain	overweight	Overweight and obesity	95	1.81	1.78
EGAN00001617530	EGAN00001617530	Genomic DNA extracted from peripheral white blood cells using two kits, the Qiamp DNA Investigator Kit for coagulated samples and Qiamp DNA Mini & Blood Mini Kit for non-coagulated samples (QIAgen Systems, Inc., Valencia, CA, USA).	Human	blood	NA	male	Iberian Population in Spain	obese	Overweight and obesity	79	0.98	3.63
EGAN00001617531	EGAN00001617531	Genomic DNA extracted from peripheral white blood cells using two kits, the Qiamp DNA Investigator Kit for coagulated samples and Qiamp DNA Mini & Blood Mini Kit for non-coagulated samples (QIAgen Systems, Inc., Valencia, CA, USA).	Human	blood	NA	male	Iberian Population in Spain	obese	Overweight and obesity	80	1.38	3.95
EGAN00001617532	EGAN00001617532	Genomic DNA extracted from peripheral white blood cells using two kits, the Qiamp DNA Investigator Kit for coagulated samples and Qiamp DNA Mini & Blood Mini Kit for non-coagulated samples (QIAgen Systems, Inc., Valencia, CA, USA).	Human	blood	NA	male	Iberian Population in Spain	overweight	Overweight and obesity	88	1.85	1.85
EGAN00001617533	EGAN00001617533	Genomic DNA extracted from peripheral white blood cells using two kits, the Qiamp DNA Investigator Kit for coagulated samples and Qiamp DNA Mini & Blood Mini Kit for non-coagulated samples (QIAgen Systems, Inc., Valencia, CA, USA).	Human	blood	NA	male	Iberian Population in Spain	obese	Overweight and obesity	84	1.12	2.76
EGAN00001617534	EGAN00001617534	Genomic DNA extracted from peripheral white blood cells using two kits, the Qiamp DNA Investigator Kit for coagulated samples and Qiamp DNA Mini & Blood Mini Kit for non-coagulated samples (QIAgen Systems, Inc., Valencia, CA, USA).	Human	blood	NA	male	Iberian Population in Spain	obese	Overweight and obesity	84	1.6	2.97
EGAN00001617535	EGAN00001617535	Genomic DNA extracted from peripheral white blood cells using two kits, the Qiamp DNA Investigator Kit for coagulated samples and Qiamp DNA Mini & Blood Mini Kit for non-coagulated samples (QIAgen Systems, Inc., Valencia, CA, USA).	Human	blood	NA	male	Iberian Population in Spain	obese	Overweight and obesity	74	2.39	2.42
EGAN00001617536	EGAN00001617536	Genomic DNA extracted from peripheral white blood cells using two kits, the Qiamp DNA Investigator Kit for coagulated samples and Qiamp DNA Mini & Blood Mini Kit for non-coagulated samples (QIAgen Systems, Inc., Valencia, CA, USA).	Human	blood	NA	male	Iberian Population in Spain	obese	Overweight and obesity	81	1.32	5.23
EGAN00001617537	EGAN00001617537	Genomic DNA extracted from peripheral white blood cells using two kits, the Qiamp DNA Investigator Kit for coagulated samples and Qiamp DNA Mini & Blood Mini Kit for non-coagulated samples (QIAgen Systems, Inc., Valencia, CA, USA).	Human	blood	NA	male	Iberian Population in Spain	obese	Overweight and obesity	90	2.18	5.39
EGAN00001617538	EGAN00001617538	Genomic DNA extracted from peripheral white blood cells using two kits, the Qiamp DNA Investigator Kit for coagulated samples and Qiamp DNA Mini & Blood Mini Kit for non-coagulated samples (QIAgen Systems, Inc., Valencia, CA, USA).	Human	blood	NA	male	Iberian Population in Spain	obese	Overweight and obesity	89	2	2.69
EGAN00001617539	EGAN00001617539	Genomic DNA extracted from peripheral white blood cells using two kits, the Qiamp DNA Investigator Kit for coagulated samples and Qiamp DNA Mini & Blood Mini Kit for non-coagulated samples (QIAgen Systems, Inc., Valencia, CA, USA).	Human	blood	NA	male	Iberian Population in Spain	obese	Overweight and obesity	83	2.89	3.01
EGAN00001617540	EGAN00001617540	Genomic DNA extracted from peripheral white blood cells using two kits, the Qiamp DNA Investigator Kit for coagulated samples and Qiamp DNA Mini & Blood Mini Kit for non-coagulated samples (QIAgen Systems, Inc., Valencia, CA, USA).	Human	blood	NA	male	Iberian Population in Spain	obese	Overweight and obesity	75	1.63	5.48
EGAN00001617541	EGAN00001617541	Genomic DNA extracted from peripheral white blood cells using two kits, the Qiamp DNA Investigator Kit for coagulated samples and Qiamp DNA Mini & Blood Mini Kit for non-coagulated samples (QIAgen Systems, Inc., Valencia, CA, USA).	Human	blood	NA	male	Iberian Population in Spain	obese	Overweight and obesity	87	1.74	3.76
EGAN00001617542	EGAN00001617542	Genomic DNA extracted from peripheral white blood cells using two kits, the Qiamp DNA Investigator Kit for coagulated samples and Qiamp DNA Mini & Blood Mini Kit for non-coagulated samples (QIAgen Systems, Inc., Valencia, CA, USA).	Human	blood	NA	male	Iberian Population in Spain	obese	Overweight and obesity	86	NA	2.53
EGAN00001617543	EGAN00001617543	Genomic DNA extracted from peripheral white blood cells using two kits, the Qiamp DNA Investigator Kit for coagulated samples and Qiamp DNA Mini & Blood Mini Kit for non-coagulated samples (QIAgen Systems, Inc., Valencia, CA, USA).	Human	blood	NA	male	Iberian Population in Spain	obese	Overweight and obesity	70	NA	3.47
EGAN00001617544	EGAN00001617544	Genomic DNA extracted from peripheral white blood cells using two kits, the Qiamp DNA Investigator Kit for coagulated samples and Qiamp DNA Mini & Blood Mini Kit for non-coagulated samples (QIAgen Systems, Inc., Valencia, CA, USA).	Human	blood	NA	male	Iberian Population in Spain	overweight	Overweight and obesity	101	3.47	2.04
EGAN00001617545	EGAN00001617545	Genomic DNA extracted from peripheral white blood cells using two kits, the Qiamp DNA Investigator Kit for coagulated samples and Qiamp DNA Mini & Blood Mini Kit for non-coagulated samples (QIAgen Systems, Inc., Valencia, CA, USA).	Human	blood	NA	male	Iberian Population in Spain	overweight	Overweight and obesity	94	3.2	1.68
EGAN00001617546	EGAN00001617546	Genomic DNA extracted from peripheral white blood cells using two kits, the Qiamp DNA Investigator Kit for coagulated samples and Qiamp DNA Mini & Blood Mini Kit for non-coagulated samples (QIAgen Systems, Inc., Valencia, CA, USA).	Human	blood	NA	male	Iberian Population in Spain	overweight	Overweight and obesity	85	2.92	1.34
EGAN00001617547	EGAN00001617547	Genomic DNA extracted from peripheral white blood cells using two kits, the Qiamp DNA Investigator Kit for coagulated samples and Qiamp DNA Mini & Blood Mini Kit for non-coagulated samples (QIAgen Systems, Inc., Valencia, CA, USA).	Human	blood	NA	male	Iberian Population in Spain	overweight	Overweight and obesity	91	NA	1.25
EGAN00001617548	EGAN00001617548	Genomic DNA extracted from peripheral white blood cells using two kits, the Qiamp DNA Investigator Kit for coagulated samples and Qiamp DNA Mini & Blood Mini Kit for non-coagulated samples (QIAgen Systems, Inc., Valencia, CA, USA).	Human	blood	NA	male	Iberian Population in Spain	overweight	Overweight and obesity	85	1.13	1.1
EGAN00001617549	EGAN00001617549	Genomic DNA extracted from peripheral white blood cells using two kits, the Qiamp DNA Investigator Kit for coagulated samples and Qiamp DNA Mini & Blood Mini Kit for non-coagulated samples (QIAgen Systems, Inc., Valencia, CA, USA).	Human	blood	NA	male	Iberian Population in Spain	overweight	Overweight and obesity	79	1.4	2.09
EGAN00001617550	EGAN00001617550	Genomic DNA extracted from peripheral white blood cells using two kits, the Qiamp DNA Investigator Kit for coagulated samples and Qiamp DNA Mini & Blood Mini Kit for non-coagulated samples (QIAgen Systems, Inc., Valencia, CA, USA).	Human	blood	NA	male	Iberian Population in Spain	overweight	Overweight and obesity	91	1.84	1.17
EGAN00001617551	EGAN00001617551	Genomic DNA extracted from peripheral white blood cells using two kits, the Qiamp DNA Investigator Kit for coagulated samples and Qiamp DNA Mini & Blood Mini Kit for non-coagulated samples (QIAgen Systems, Inc., Valencia, CA, USA).	Human	blood	NA	male	Iberian Population in Spain	obese	Overweight and obesity	85	3.42	2.73
EGAN00001617552	EGAN00001617552	Genomic DNA extracted from peripheral white blood cells using two kits, the Qiamp DNA Investigator Kit for coagulated samples and Qiamp DNA Mini & Blood Mini Kit for non-coagulated samples (QIAgen Systems, Inc., Valencia, CA, USA).	Human	blood	NA	male	Iberian Population in Spain	overweight	Overweight and obesity	80	2.51	2.04
EGAN00001617553	EGAN00001617553	Genomic DNA extracted from peripheral white blood cells using two kits, the Qiamp DNA Investigator Kit for coagulated samples and Qiamp DNA Mini & Blood Mini Kit for non-coagulated samples (QIAgen Systems, Inc., Valencia, CA, USA).	Human	blood	NA	male	Iberian Population in Spain	obese	Overweight and obesity	82	0.99	2.26
EGAN00001617554	EGAN00001617554	Genomic DNA extracted from peripheral white blood cells using two kits, the Qiamp DNA Investigator Kit for coagulated samples and Qiamp DNA Mini & Blood Mini Kit for non-coagulated samples (QIAgen Systems, Inc., Valencia, CA, USA).	Human	blood	NA	male	Iberian Population in Spain	overweight	Overweight and obesity	84	1.02	1.52
EGAN00001617555	EGAN00001617555	Genomic DNA extracted from peripheral white blood cells using two kits, the Qiamp DNA Investigator Kit for coagulated samples and Qiamp DNA Mini & Blood Mini Kit for non-coagulated samples (QIAgen Systems, Inc., Valencia, CA, USA).	Human	blood	NA	male	Iberian Population in Spain	overweight	Overweight and obesity	87	1.83	0.67
EGAN00001617556	EGAN00001617556	Genomic DNA extracted from peripheral white blood cells using two kits, the Qiamp DNA Investigator Kit for coagulated samples and Qiamp DNA Mini & Blood Mini Kit for non-coagulated samples (QIAgen Systems, Inc., Valencia, CA, USA).	Human	blood	NA	male	Iberian Population in Spain	overweight	Overweight and obesity	84	1.29	0.95
EGAN00001617557	EGAN00001617557	Genomic DNA extracted from peripheral white blood cells using two kits, the Qiamp DNA Investigator Kit for coagulated samples and Qiamp DNA Mini & Blood Mini Kit for non-coagulated samples (QIAgen Systems, Inc., Valencia, CA, USA).	Human	blood	NA	male	Iberian Population in Spain	obese	Overweight and obesity	98	3.07	2.44
EGAN00001617558	EGAN00001617558	Genomic DNA extracted from peripheral white blood cells using two kits, the Qiamp DNA Investigator Kit for coagulated samples and Qiamp DNA Mini & Blood Mini Kit for non-coagulated samples (QIAgen Systems, Inc., Valencia, CA, USA).	Human	blood	NA	male	Iberian Population in Spain	obese	Overweight and obesity	66	0.32	2.56
EGAN00001617559	EGAN00001617559	Genomic DNA extracted from peripheral white blood cells using two kits, the Qiamp DNA Investigator Kit for coagulated samples and Qiamp DNA Mini & Blood Mini Kit for non-coagulated samples (QIAgen Systems, Inc., Valencia, CA, USA).	Human	blood	NA	male	Iberian Population in Spain	overweight	Overweight and obesity	101	NA	1.14
EGAN00001617560	EGAN00001617560	Genomic DNA extracted from peripheral white blood cells using two kits, the Qiamp DNA Investigator Kit for coagulated samples and Qiamp DNA Mini & Blood Mini Kit for non-coagulated samples (QIAgen Systems, Inc., Valencia, CA, USA).	Human	blood	NA	male	Iberian Population in Spain	overweight	Overweight and obesity	95	4.67	1.45
EGAN00001617561	EGAN00001617561	Genomic DNA extracted from peripheral white blood cells using two kits, the Qiamp DNA Investigator Kit for coagulated samples and Qiamp DNA Mini & Blood Mini Kit for non-coagulated samples (QIAgen Systems, Inc., Valencia, CA, USA).	Human	blood	NA	male	Iberian Population in Spain	obese	Overweight and obesity	87	3.01	6.8
EGAN00001617562	EGAN00001617562	Genomic DNA extracted from peripheral white blood cells using two kits, the Qiamp DNA Investigator Kit for coagulated samples and Qiamp DNA Mini & Blood Mini Kit for non-coagulated samples (QIAgen Systems, Inc., Valencia, CA, USA).	Human	blood	NA	male	Iberian Population in Spain	overweight	Overweight and obesity	76	1.13	1
EGAN00001617563	EGAN00001617563	Genomic DNA extracted from peripheral white blood cells using two kits, the Qiamp DNA Investigator Kit for coagulated samples and Qiamp DNA Mini & Blood Mini Kit for non-coagulated samples (QIAgen Systems, Inc., Valencia, CA, USA).	Human	blood	NA	male	Iberian Population in Spain	overweight	Overweight and obesity	74	0.57	0.8
EGAN00001617564	EGAN00001617564	Genomic DNA extracted from peripheral white blood cells using two kits, the Qiamp DNA Investigator Kit for coagulated samples and Qiamp DNA Mini & Blood Mini Kit for non-coagulated samples (QIAgen Systems, Inc., Valencia, CA, USA).	Human	blood	NA	male	Iberian Population in Spain	overweight	Overweight and obesity	81	1.28	1.12
EGAN00001617565	EGAN00001617565	Genomic DNA extracted from peripheral white blood cells using two kits, the Qiamp DNA Investigator Kit for coagulated samples and Qiamp DNA Mini & Blood Mini Kit for non-coagulated samples (QIAgen Systems, Inc., Valencia, CA, USA).	Human	blood	NA	male	Iberian Population in Spain	overweight	Overweight and obesity	84	1.21	0.9
EGAN00001617566	EGAN00001617566	Genomic DNA extracted from peripheral white blood cells using two kits, the Qiamp DNA Investigator Kit for coagulated samples and Qiamp DNA Mini & Blood Mini Kit for non-coagulated samples (QIAgen Systems, Inc., Valencia, CA, USA).	Human	blood	NA	male	Iberian Population in Spain	overweight	Overweight and obesity	83	1.66	0.75
EGAN00001617567	EGAN00001617567	Genomic DNA extracted from peripheral white blood cells using two kits, the Qiamp DNA Investigator Kit for coagulated samples and Qiamp DNA Mini & Blood Mini Kit for non-coagulated samples (QIAgen Systems, Inc., Valencia, CA, USA).	Human	blood	NA	male	Iberian Population in Spain	obese	Overweight and obesity	87	2.58	2.62
EGAN00001617568	EGAN00001617568	Genomic DNA extracted from peripheral white blood cells using two kits, the Qiamp DNA Investigator Kit for coagulated samples and Qiamp DNA Mini & Blood Mini Kit for non-coagulated samples (QIAgen Systems, Inc., Valencia, CA, USA).	Human	blood	NA	male	Iberian Population in Spain	obese	Overweight and obesity	85	1.72	2.47
EGAN00001617569	EGAN00001617569	Genomic DNA extracted from peripheral white blood cells using two kits, the Qiamp DNA Investigator Kit for coagulated samples and Qiamp DNA Mini & Blood Mini Kit for non-coagulated samples (QIAgen Systems, Inc., Valencia, CA, USA).	Human	blood	NA	male	Iberian Population in Spain	overweight	Overweight and obesity	75	0.8	1.17
EGAN00001617570	EGAN00001617570	Genomic DNA extracted from peripheral white blood cells using two kits, the Qiamp DNA Investigator Kit for coagulated samples and Qiamp DNA Mini & Blood Mini Kit for non-coagulated samples (QIAgen Systems, Inc., Valencia, CA, USA).	Human	blood	NA	male	Iberian Population in Spain	overweight	Overweight and obesity	85	1.6	1.24
EGAN00001617571	EGAN00001617571	Genomic DNA extracted from peripheral white blood cells using two kits, the Qiamp DNA Investigator Kit for coagulated samples and Qiamp DNA Mini & Blood Mini Kit for non-coagulated samples (QIAgen Systems, Inc., Valencia, CA, USA).	Human	blood	NA	male	Iberian Population in Spain	obese	Overweight and obesity	79	0.94	3.89
EGAN00001617572	EGAN00001617572	Genomic DNA extracted from peripheral white blood cells using two kits, the Qiamp DNA Investigator Kit for coagulated samples and Qiamp DNA Mini & Blood Mini Kit for non-coagulated samples (QIAgen Systems, Inc., Valencia, CA, USA).	Human	blood	NA	male	Iberian Population in Spain	obese	Overweight and obesity	84	3.17	3.13
EGAN00001617573	EGAN00001617573	Genomic DNA extracted from peripheral white blood cells using two kits, the Qiamp DNA Investigator Kit for coagulated samples and Qiamp DNA Mini & Blood Mini Kit for non-coagulated samples (QIAgen Systems, Inc., Valencia, CA, USA).	Human	blood	NA	male	Iberian Population in Spain	obese	Overweight and obesity	85	1.43	4.09
EGAN00001617574	EGAN00001617574	Genomic DNA extracted from peripheral white blood cells using two kits, the Qiamp DNA Investigator Kit for coagulated samples and Qiamp DNA Mini & Blood Mini Kit for non-coagulated samples (QIAgen Systems, Inc., Valencia, CA, USA).	Human	blood	NA	male	Iberian Population in Spain	obese	Overweight and obesity	70	1.38	2.89
EGAN00001617575	EGAN00001617575	Genomic DNA extracted from peripheral white blood cells using two kits, the Qiamp DNA Investigator Kit for coagulated samples and Qiamp DNA Mini & Blood Mini Kit for non-coagulated samples (QIAgen Systems, Inc., Valencia, CA, USA).	Human	blood	NA	male	Iberian Population in Spain	obese	Overweight and obesity	86	1	8.41
EGAN00001617576	EGAN00001617576	Genomic DNA extracted from peripheral white blood cells using two kits, the Qiamp DNA Investigator Kit for coagulated samples and Qiamp DNA Mini & Blood Mini Kit for non-coagulated samples (QIAgen Systems, Inc., Valencia, CA, USA).	Human	blood	NA	male	Iberian Population in Spain	obese	Overweight and obesity	87	1.44	2.99
EGAN00001617577	EGAN00001617577	Genomic DNA extracted from peripheral white blood cells using two kits, the Qiamp DNA Investigator Kit for coagulated samples and Qiamp DNA Mini & Blood Mini Kit for non-coagulated samples (QIAgen Systems, Inc., Valencia, CA, USA).	Human	blood	NA	male	Iberian Population in Spain	obese	Overweight and obesity	101	1.85	3.39
EGAN00001617578	EGAN00001617578	Genomic DNA extracted from peripheral white blood cells using two kits, the Qiamp DNA Investigator Kit for coagulated samples and Qiamp DNA Mini & Blood Mini Kit for non-coagulated samples (QIAgen Systems, Inc., Valencia, CA, USA).	Human	blood	NA	male	Iberian Population in Spain	obese	Overweight and obesity	88	1.07	2.35
EGAN00001617579	EGAN00001617579	Genomic DNA extracted from peripheral white blood cells using two kits, the Qiamp DNA Investigator Kit for coagulated samples and Qiamp DNA Mini & Blood Mini Kit for non-coagulated samples (QIAgen Systems, Inc., Valencia, CA, USA).	Human	blood	NA	male	Iberian Population in Spain	obese	Overweight and obesity	77	4.83	3.22
EGAN00001617580	EGAN00001617580	Genomic DNA extracted from peripheral white blood cells using two kits, the Qiamp DNA Investigator Kit for coagulated samples and Qiamp DNA Mini & Blood Mini Kit for non-coagulated samples (QIAgen Systems, Inc., Valencia, CA, USA).	Human	blood	NA	male	Iberian Population in Spain	obese	Overweight and obesity	101	1.1	2.06
EGAN00001617581	EGAN00001617581	Genomic DNA extracted from peripheral white blood cells using two kits, the Qiamp DNA Investigator Kit for coagulated samples and Qiamp DNA Mini & Blood Mini Kit for non-coagulated samples (QIAgen Systems, Inc., Valencia, CA, USA).	Human	blood	NA	male	Iberian Population in Spain	obese	Overweight and obesity	90	1.8	3.31
EGAN00001617582	EGAN00001617582	Genomic DNA extracted from peripheral white blood cells using two kits, the Qiamp DNA Investigator Kit for coagulated samples and Qiamp DNA Mini & Blood Mini Kit for non-coagulated samples (QIAgen Systems, Inc., Valencia, CA, USA).	Human	blood	NA	male	Iberian Population in Spain	overweight	Overweight and obesity	39	0.67	1.69
EGAN00001617583	EGAN00001617583	Genomic DNA extracted from peripheral white blood cells using two kits, the Qiamp DNA Investigator Kit for coagulated samples and Qiamp DNA Mini & Blood Mini Kit for non-coagulated samples (QIAgen Systems, Inc., Valencia, CA, USA).	Human	blood	NA	male	Iberian Population in Spain	obese	Overweight and obesity	87	0.43	3.01
EGAN00001617584	EGAN00001617584	Genomic DNA extracted from peripheral white blood cells using two kits, the Qiamp DNA Investigator Kit for coagulated samples and Qiamp DNA Mini & Blood Mini Kit for non-coagulated samples (QIAgen Systems, Inc., Valencia, CA, USA).	Human	blood	NA	male	Iberian Population in Spain	obese	Overweight and obesity	91	2.38	3.08
EGAN00001617585	EGAN00001617585	Genomic DNA extracted from peripheral white blood cells using two kits, the Qiamp DNA Investigator Kit for coagulated samples and Qiamp DNA Mini & Blood Mini Kit for non-coagulated samples (QIAgen Systems, Inc., Valencia, CA, USA).	Human	blood	NA	male	Iberian Population in Spain	obese	Overweight and obesity	88	1.78	2.1
EGAN00001617586	EGAN00001617586	Genomic DNA extracted from peripheral white blood cells using two kits, the Qiamp DNA Investigator Kit for coagulated samples and Qiamp DNA Mini & Blood Mini Kit for non-coagulated samples (QIAgen Systems, Inc., Valencia, CA, USA).	Human	blood	NA	male	Iberian Population in Spain	obese	Overweight and obesity	73	1.41	4.72
EGAN00001617587	EGAN00001617587	Genomic DNA extracted from peripheral white blood cells using two kits, the Qiamp DNA Investigator Kit for coagulated samples and Qiamp DNA Mini & Blood Mini Kit for non-coagulated samples (QIAgen Systems, Inc., Valencia, CA, USA).	Human	blood	NA	male	Iberian Population in Spain	obese	Overweight and obesity	85	1.55	2.77
EGAN00001617588	EGAN00001617588	Genomic DNA extracted from peripheral white blood cells using two kits, the Qiamp DNA Investigator Kit for coagulated samples and Qiamp DNA Mini & Blood Mini Kit for non-coagulated samples (QIAgen Systems, Inc., Valencia, CA, USA).	Human	blood	NA	male	Iberian Population in Spain	obese	Overweight and obesity	81	2.18	3.66
EGAN00001617589	EGAN00001617589	Genomic DNA extracted from peripheral white blood cells using two kits, the Qiamp DNA Investigator Kit for coagulated samples and Qiamp DNA Mini & Blood Mini Kit for non-coagulated samples (QIAgen Systems, Inc., Valencia, CA, USA).	Human	blood	NA	male	Iberian Population in Spain	obese	Overweight and obesity	81	1.6	4.16
EGAN00001617590	EGAN00001617590	Genomic DNA extracted from peripheral white blood cells using two kits, the Qiamp DNA Investigator Kit for coagulated samples and Qiamp DNA Mini & Blood Mini Kit for non-coagulated samples (QIAgen Systems, Inc., Valencia, CA, USA).	Human	blood	NA	male	Iberian Population in Spain	obese	Overweight and obesity	72	2.1	9.8
EGAN00001617591	EGAN00001617591	Genomic DNA extracted from peripheral white blood cells using two kits, the Qiamp DNA Investigator Kit for coagulated samples and Qiamp DNA Mini & Blood Mini Kit for non-coagulated samples (QIAgen Systems, Inc., Valencia, CA, USA).	Human	blood	NA	male	Iberian Population in Spain	obese	Overweight and obesity	74	0.57	4.38
EGAN00001617592	EGAN00001617592	Genomic DNA extracted from peripheral white blood cells using two kits, the Qiamp DNA Investigator Kit for coagulated samples and Qiamp DNA Mini & Blood Mini Kit for non-coagulated samples (QIAgen Systems, Inc., Valencia, CA, USA).	Human	blood	NA	male	Iberian Population in Spain	overweight	Overweight and obesity	76	0.54	1.01
EGAN00001617593	EGAN00001617593	Genomic DNA extracted from peripheral white blood cells using two kits, the Qiamp DNA Investigator Kit for coagulated samples and Qiamp DNA Mini & Blood Mini Kit for non-coagulated samples (QIAgen Systems, Inc., Valencia, CA, USA).	Human	blood	NA	male	Iberian Population in Spain	obese	Overweight and obesity	78	1.25	4.91
EGAN00001617594	EGAN00001617594	Genomic DNA extracted from peripheral white blood cells using two kits, the Qiamp DNA Investigator Kit for coagulated samples and Qiamp DNA Mini & Blood Mini Kit for non-coagulated samples (QIAgen Systems, Inc., Valencia, CA, USA).	Human	blood	NA	male	Iberian Population in Spain	obese	Overweight and obesity	72	0.87	5.09
EGAN00001617595	EGAN00001617595	Genomic DNA extracted from peripheral white blood cells using two kits, the Qiamp DNA Investigator Kit for coagulated samples and Qiamp DNA Mini & Blood Mini Kit for non-coagulated samples (QIAgen Systems, Inc., Valencia, CA, USA).	Human	blood	NA	male	Iberian Population in Spain	obese	Overweight and obesity	79	1.37	4.91
EGAN00001617596	EGAN00001617596	Genomic DNA extracted from peripheral white blood cells using two kits, the Qiamp DNA Investigator Kit for coagulated samples and Qiamp DNA Mini & Blood Mini Kit for non-coagulated samples (QIAgen Systems, Inc., Valencia, CA, USA).	Human	blood	NA	male	Iberian Population in Spain	obese	Overweight and obesity	73	2.78	2.72
EGAN00001617597	EGAN00001617597	Genomic DNA extracted from peripheral white blood cells using two kits, the Qiamp DNA Investigator Kit for coagulated samples and Qiamp DNA Mini & Blood Mini Kit for non-coagulated samples (QIAgen Systems, Inc., Valencia, CA, USA).	Human	blood	NA	male	Iberian Population in Spain	obese	Overweight and obesity	82	1.9	4.95
EGAN00001617598	EGAN00001617598	Genomic DNA extracted from peripheral white blood cells using two kits, the Qiamp DNA Investigator Kit for coagulated samples and Qiamp DNA Mini & Blood Mini Kit for non-coagulated samples (QIAgen Systems, Inc., Valencia, CA, USA).	Human	blood	NA	male	Iberian Population in Spain	overweight	Overweight and obesity	82	1.8	1.36
EGAN00001617599	EGAN00001617599	Genomic DNA extracted from peripheral white blood cells using two kits, the Qiamp DNA Investigator Kit for coagulated samples and Qiamp DNA Mini & Blood Mini Kit for non-coagulated samples (QIAgen Systems, Inc., Valencia, CA, USA).	Human	blood	NA	male	Iberian Population in Spain	obese	Overweight and obesity	86	1.51	3.29
EGAN00001617600	EGAN00001617600	Genomic DNA extracted from peripheral white blood cells using two kits, the Qiamp DNA Investigator Kit for coagulated samples and Qiamp DNA Mini & Blood Mini Kit for non-coagulated samples (QIAgen Systems, Inc., Valencia, CA, USA).	Human	blood	NA	male	Iberian Population in Spain	obese	Overweight and obesity	90	2.33	5.89
EGAN00001617601	EGAN00001617601	Genomic DNA extracted from peripheral white blood cells using two kits, the Qiamp DNA Investigator Kit for coagulated samples and Qiamp DNA Mini & Blood Mini Kit for non-coagulated samples (QIAgen Systems, Inc., Valencia, CA, USA).	Human	blood	NA	male	Iberian Population in Spain	obese	Overweight and obesity	81	0.98	4.73
EGAN00001617602	EGAN00001617602	Genomic DNA extracted from peripheral white blood cells using two kits, the Qiamp DNA Investigator Kit for coagulated samples and Qiamp DNA Mini & Blood Mini Kit for non-coagulated samples (QIAgen Systems, Inc., Valencia, CA, USA).	Human	blood	NA	male	Iberian Population in Spain	obese	Overweight and obesity	99	1.66	2.69
EGAN00001617603	EGAN00001617603	Genomic DNA extracted from peripheral white blood cells using two kits, the Qiamp DNA Investigator Kit for coagulated samples and Qiamp DNA Mini & Blood Mini Kit for non-coagulated samples (QIAgen Systems, Inc., Valencia, CA, USA).	Human	blood	NA	male	Iberian Population in Spain	obese	Overweight and obesity	89	4.22	6.2
EGAN00001617604	EGAN00001617604	Genomic DNA extracted from peripheral white blood cells using two kits, the Qiamp DNA Investigator Kit for coagulated samples and Qiamp DNA Mini & Blood Mini Kit for non-coagulated samples (QIAgen Systems, Inc., Valencia, CA, USA).	Human	blood	NA	male	Iberian Population in Spain	obese	Overweight and obesity	90	3.98	5.14
EGAN00001617605	EGAN00001617605	Genomic DNA extracted from peripheral white blood cells using two kits, the Qiamp DNA Investigator Kit for coagulated samples and Qiamp DNA Mini & Blood Mini Kit for non-coagulated samples (QIAgen Systems, Inc., Valencia, CA, USA).	Human	blood	NA	male	Iberian Population in Spain	overweight	Overweight and obesity	88	1.61	1.05
EGAN00001617606	EGAN00001617606	Genomic DNA extracted from peripheral white blood cells using two kits, the Qiamp DNA Investigator Kit for coagulated samples and Qiamp DNA Mini & Blood Mini Kit for non-coagulated samples (QIAgen Systems, Inc., Valencia, CA, USA).	Human	blood	NA	male	Iberian Population in Spain	obese	Overweight and obesity	84	1.52	3.23
EGAN00001617607	EGAN00001617607	Genomic DNA extracted from peripheral white blood cells using two kits, the Qiamp DNA Investigator Kit for coagulated samples and Qiamp DNA Mini & Blood Mini Kit for non-coagulated samples (QIAgen Systems, Inc., Valencia, CA, USA).	Human	blood	NA	male	Iberian Population in Spain	overweight	Overweight and obesity	86	1.27	1.56
EGAN00001617608	EGAN00001617608	Genomic DNA extracted from peripheral white blood cells using two kits, the Qiamp DNA Investigator Kit for coagulated samples and Qiamp DNA Mini & Blood Mini Kit for non-coagulated samples (QIAgen Systems, Inc., Valencia, CA, USA).	Human	blood	NA	male	Iberian Population in Spain	overweight	Overweight and obesity	89	2.01	1.66
EGAN00001617609	EGAN00001617609	Genomic DNA extracted from peripheral white blood cells using two kits, the Qiamp DNA Investigator Kit for coagulated samples and Qiamp DNA Mini & Blood Mini Kit for non-coagulated samples (QIAgen Systems, Inc., Valencia, CA, USA).	Human	blood	NA	male	Iberian Population in Spain	overweight	Overweight and obesity	85	0.99	1.35
EGAN00001617610	EGAN00001617610	Genomic DNA extracted from peripheral white blood cells using two kits, the Qiamp DNA Investigator Kit for coagulated samples and Qiamp DNA Mini & Blood Mini Kit for non-coagulated samples (QIAgen Systems, Inc., Valencia, CA, USA).	Human	blood	NA	male	Iberian Population in Spain	obese	Overweight and obesity	96	9.48	6.87
EGAN00001617611	EGAN00001617611	Genomic DNA extracted from peripheral white blood cells using two kits, the Qiamp DNA Investigator Kit for coagulated samples and Qiamp DNA Mini & Blood Mini Kit for non-coagulated samples (QIAgen Systems, Inc., Valencia, CA, USA).	Human	blood	NA	male	Iberian Population in Spain	overweight	Overweight and obesity	86	1.1	1.22
EGAN00001617612	EGAN00001617612	Genomic DNA extracted from peripheral white blood cells using two kits, the Qiamp DNA Investigator Kit for coagulated samples and Qiamp DNA Mini & Blood Mini Kit for non-coagulated samples (QIAgen Systems, Inc., Valencia, CA, USA).	Human	blood	NA	male	Iberian Population in Spain	obese	Overweight and obesity	85	2.52	3.88
EGAN00001617613	EGAN00001617613	Genomic DNA extracted from peripheral white blood cells using two kits, the Qiamp DNA Investigator Kit for coagulated samples and Qiamp DNA Mini & Blood Mini Kit for non-coagulated samples (QIAgen Systems, Inc., Valencia, CA, USA).	Human	blood	NA	male	Iberian Population in Spain	obese	Overweight and obesity	86	2.63	3
EGAN00001617614	EGAN00001617614	Genomic DNA extracted from peripheral white blood cells using two kits, the Qiamp DNA Investigator Kit for coagulated samples and Qiamp DNA Mini & Blood Mini Kit for non-coagulated samples (QIAgen Systems, Inc., Valencia, CA, USA).	Human	blood	NA	male	Iberian Population in Spain	obese	Overweight and obesity	83	1.62	2.44
EGAN00001617615	EGAN00001617615	Genomic DNA extracted from peripheral white blood cells using two kits, the Qiamp DNA Investigator Kit for coagulated samples and Qiamp DNA Mini & Blood Mini Kit for non-coagulated samples (QIAgen Systems, Inc., Valencia, CA, USA).	Human	blood	NA	male	Iberian Population in Spain	overweight	Overweight and obesity	95	0.8	0.79
EGAN00001617616	EGAN00001617616	Genomic DNA extracted from peripheral white blood cells using two kits, the Qiamp DNA Investigator Kit for coagulated samples and Qiamp DNA Mini & Blood Mini Kit for non-coagulated samples (QIAgen Systems, Inc., Valencia, CA, USA).	Human	blood	NA	male	Iberian Population in Spain	obese	Overweight and obesity	82	2.47	3.16
EGAN00001617617	EGAN00001617617	Genomic DNA extracted from peripheral white blood cells using two kits, the Qiamp DNA Investigator Kit for coagulated samples and Qiamp DNA Mini & Blood Mini Kit for non-coagulated samples (QIAgen Systems, Inc., Valencia, CA, USA).	Human	blood	NA	male	Iberian Population in Spain	obese	Overweight and obesity	80	3.48	2.86
EGAN00001617618	EGAN00001617618	Genomic DNA extracted from peripheral white blood cells using two kits, the Qiamp DNA Investigator Kit for coagulated samples and Qiamp DNA Mini & Blood Mini Kit for non-coagulated samples (QIAgen Systems, Inc., Valencia, CA, USA).	Human	blood	NA	male	Iberian Population in Spain	obese	Overweight and obesity	80	0.44	3.43
EGAN00001617619	EGAN00001617619	Genomic DNA extracted from peripheral white blood cells using two kits, the Qiamp DNA Investigator Kit for coagulated samples and Qiamp DNA Mini & Blood Mini Kit for non-coagulated samples (QIAgen Systems, Inc., Valencia, CA, USA).	Human	blood	NA	male	Iberian Population in Spain	obese	Overweight and obesity	92	1.68	4.09
EGAN00001617620	EGAN00001617620	Genomic DNA extracted from peripheral white blood cells using two kits, the Qiamp DNA Investigator Kit for coagulated samples and Qiamp DNA Mini & Blood Mini Kit for non-coagulated samples (QIAgen Systems, Inc., Valencia, CA, USA).	Human	blood	NA	male	Iberian Population in Spain	obese	Overweight and obesity	86	1.47	4.96
EGAN00001617621	EGAN00001617621	Genomic DNA extracted from peripheral white blood cells using two kits, the Qiamp DNA Investigator Kit for coagulated samples and Qiamp DNA Mini & Blood Mini Kit for non-coagulated samples (QIAgen Systems, Inc., Valencia, CA, USA).	Human	blood	NA	male	Iberian Population in Spain	obese	Overweight and obesity	88	1.96	4.51
EGAN00001617622	EGAN00001617622	Genomic DNA extracted from peripheral white blood cells using two kits, the Qiamp DNA Investigator Kit for coagulated samples and Qiamp DNA Mini & Blood Mini Kit for non-coagulated samples (QIAgen Systems, Inc., Valencia, CA, USA).	Human	blood	NA	male	Iberian Population in Spain	obese	Overweight and obesity	85	1.74	3.09
EGAN00001617623	EGAN00001617623	Genomic DNA extracted from peripheral white blood cells using two kits, the Qiamp DNA Investigator Kit for coagulated samples and Qiamp DNA Mini & Blood Mini Kit for non-coagulated samples (QIAgen Systems, Inc., Valencia, CA, USA).	Human	blood	NA	male	Iberian Population in Spain	obese	Overweight and obesity	87	1.49	2.7
EGAN00001617624	EGAN00001617624	Genomic DNA extracted from peripheral white blood cells using two kits, the Qiamp DNA Investigator Kit for coagulated samples and Qiamp DNA Mini & Blood Mini Kit for non-coagulated samples (QIAgen Systems, Inc., Valencia, CA, USA).	Human	blood	NA	male	Iberian Population in Spain	obese	Overweight and obesity	78	1.01	2.24
EGAN00001617625	EGAN00001617625	Genomic DNA extracted from peripheral white blood cells using two kits, the Qiamp DNA Investigator Kit for coagulated samples and Qiamp DNA Mini & Blood Mini Kit for non-coagulated samples (QIAgen Systems, Inc., Valencia, CA, USA).	Human	blood	NA	male	Iberian Population in Spain	obese	Overweight and obesity	86	2.51	2.7
EGAN00001617626	EGAN00001617626	Genomic DNA extracted from peripheral white blood cells using two kits, the Qiamp DNA Investigator Kit for coagulated samples and Qiamp DNA Mini & Blood Mini Kit for non-coagulated samples (QIAgen Systems, Inc., Valencia, CA, USA).	Human	blood	NA	male	Iberian Population in Spain	obese	Overweight and obesity	80	0.92	3.75
EGAN00001617627	EGAN00001617627	Genomic DNA extracted from peripheral white blood cells using two kits, the Qiamp DNA Investigator Kit for coagulated samples and Qiamp DNA Mini & Blood Mini Kit for non-coagulated samples (QIAgen Systems, Inc., Valencia, CA, USA).	Human	blood	NA	male	Iberian Population in Spain	overweight	Overweight and obesity	87	0.99	1.39
EGAN00001617628	EGAN00001617628	Genomic DNA extracted from peripheral white blood cells using two kits, the Qiamp DNA Investigator Kit for coagulated samples and Qiamp DNA Mini & Blood Mini Kit for non-coagulated samples (QIAgen Systems, Inc., Valencia, CA, USA).	Human	blood	NA	male	Iberian Population in Spain	obese	Overweight and obesity	75	2.17	4.58
EGAN00001617629	EGAN00001617629	Genomic DNA extracted from peripheral white blood cells using two kits, the Qiamp DNA Investigator Kit for coagulated samples and Qiamp DNA Mini & Blood Mini Kit for non-coagulated samples (QIAgen Systems, Inc., Valencia, CA, USA).	Human	blood	NA	male	Iberian Population in Spain	obese	Overweight and obesity	77	1.54	5.71
EGAN00001617630	EGAN00001617630	Genomic DNA extracted from peripheral white blood cells using two kits, the Qiamp DNA Investigator Kit for coagulated samples and Qiamp DNA Mini & Blood Mini Kit for non-coagulated samples (QIAgen Systems, Inc., Valencia, CA, USA).	Human	blood	NA	male	Iberian Population in Spain	obese	Overweight and obesity	86	NA	2.92
EGAN00001617631	EGAN00001617631	Genomic DNA extracted from peripheral white blood cells using two kits, the Qiamp DNA Investigator Kit for coagulated samples and Qiamp DNA Mini & Blood Mini Kit for non-coagulated samples (QIAgen Systems, Inc., Valencia, CA, USA).	Human	blood	NA	male	Iberian Population in Spain	obese	Overweight and obesity	79	0.76	4.58
EGAN00001617632	EGAN00001617632	Genomic DNA extracted from peripheral white blood cells using two kits, the Qiamp DNA Investigator Kit for coagulated samples and Qiamp DNA Mini & Blood Mini Kit for non-coagulated samples (QIAgen Systems, Inc., Valencia, CA, USA).	Human	blood	NA	male	Iberian Population in Spain	obese	Overweight and obesity	88	3.41	6.26
EGAN00001617633	EGAN00001617633	Genomic DNA extracted from peripheral white blood cells using two kits, the Qiamp DNA Investigator Kit for coagulated samples and Qiamp DNA Mini & Blood Mini Kit for non-coagulated samples (QIAgen Systems, Inc., Valencia, CA, USA).	Human	blood	NA	male	Iberian Population in Spain	obese	Overweight and obesity	77	1.71	2.85
EGAN00001617634	EGAN00001617634	Genomic DNA extracted from peripheral white blood cells using two kits, the Qiamp DNA Investigator Kit for coagulated samples and Qiamp DNA Mini & Blood Mini Kit for non-coagulated samples (QIAgen Systems, Inc., Valencia, CA, USA).	Human	blood	NA	male	Iberian Population in Spain	obese	Overweight and obesity	70	1.16	7.37
EGAN00001617635	EGAN00001617635	Genomic DNA extracted from peripheral white blood cells using two kits, the Qiamp DNA Investigator Kit for coagulated samples and Qiamp DNA Mini & Blood Mini Kit for non-coagulated samples (QIAgen Systems, Inc., Valencia, CA, USA).	Human	blood	NA	male	Iberian Population in Spain	obese	Overweight and obesity	81	1.08	3.6
EGAN00001617636	EGAN00001617636	Genomic DNA extracted from peripheral white blood cells using two kits, the Qiamp DNA Investigator Kit for coagulated samples and Qiamp DNA Mini & Blood Mini Kit for non-coagulated samples (QIAgen Systems, Inc., Valencia, CA, USA).	Human	blood	NA	male	Iberian Population in Spain	obese	Overweight and obesity	67	0.31	3.66
EGAN00001617637	EGAN00001617637	Genomic DNA extracted from peripheral white blood cells using two kits, the Qiamp DNA Investigator Kit for coagulated samples and Qiamp DNA Mini & Blood Mini Kit for non-coagulated samples (QIAgen Systems, Inc., Valencia, CA, USA).	Human	blood	NA	male	Iberian Population in Spain	obese	Overweight and obesity	68	0.32	3.2
EGAN00001617638	EGAN00001617638	Genomic DNA extracted from peripheral white blood cells using two kits, the Qiamp DNA Investigator Kit for coagulated samples and Qiamp DNA Mini & Blood Mini Kit for non-coagulated samples (QIAgen Systems, Inc., Valencia, CA, USA).	Human	blood	NA	male	Iberian Population in Spain	obese	Overweight and obesity	66	0.75	2.44
EGAN00001617639	EGAN00001617639	Genomic DNA extracted from peripheral white blood cells using two kits, the Qiamp DNA Investigator Kit for coagulated samples and Qiamp DNA Mini & Blood Mini Kit for non-coagulated samples (QIAgen Systems, Inc., Valencia, CA, USA).	Human	blood	NA	male	Iberian Population in Spain	obese	Overweight and obesity	80	1.15	2.32
EGAN00001617640	EGAN00001617640	Genomic DNA extracted from peripheral white blood cells using two kits, the Qiamp DNA Investigator Kit for coagulated samples and Qiamp DNA Mini & Blood Mini Kit for non-coagulated samples (QIAgen Systems, Inc., Valencia, CA, USA).	Human	blood	NA	male	Iberian Population in Spain	overweight	Overweight and obesity	83	1.58	1.07
EGAN00001617641	EGAN00001617641	Genomic DNA extracted from peripheral white blood cells using two kits, the Qiamp DNA Investigator Kit for coagulated samples and Qiamp DNA Mini & Blood Mini Kit for non-coagulated samples (QIAgen Systems, Inc., Valencia, CA, USA).	Human	blood	NA	male	Iberian Population in Spain	obese	Overweight and obesity	80	1.98	2.3
EGAN00001617642	EGAN00001617642	Genomic DNA extracted from peripheral white blood cells using two kits, the Qiamp DNA Investigator Kit for coagulated samples and Qiamp DNA Mini & Blood Mini Kit for non-coagulated samples (QIAgen Systems, Inc., Valencia, CA, USA).	Human	blood	NA	male	Iberian Population in Spain	obese	Overweight and obesity	NA	NA	2.88
EGAN00001617643	EGAN00001617643	Genomic DNA extracted from peripheral white blood cells using two kits, the Qiamp DNA Investigator Kit for coagulated samples and Qiamp DNA Mini & Blood Mini Kit for non-coagulated samples (QIAgen Systems, Inc., Valencia, CA, USA).	Human	blood	NA	male	Iberian Population in Spain	obese	Overweight and obesity	76	3.55	2.98
EGAN00001617644	EGAN00001617644	Genomic DNA extracted from peripheral white blood cells using two kits, the Qiamp DNA Investigator Kit for coagulated samples and Qiamp DNA Mini & Blood Mini Kit for non-coagulated samples (QIAgen Systems, Inc., Valencia, CA, USA).	Human	blood	NA	male	Iberian Population in Spain	obese	Overweight and obesity	82	4.15	2.34
EGAN00001617645	EGAN00001617645	Genomic DNA extracted from peripheral white blood cells using two kits, the Qiamp DNA Investigator Kit for coagulated samples and Qiamp DNA Mini & Blood Mini Kit for non-coagulated samples (QIAgen Systems, Inc., Valencia, CA, USA).	Human	blood	NA	male	Iberian Population in Spain	obese	Overweight and obesity	79	2.05	2.98
EGAN00001617646	EGAN00001617646	Genomic DNA extracted from peripheral white blood cells using two kits, the Qiamp DNA Investigator Kit for coagulated samples and Qiamp DNA Mini & Blood Mini Kit for non-coagulated samples (QIAgen Systems, Inc., Valencia, CA, USA).	Human	blood	NA	male	Iberian Population in Spain	obese	Overweight and obesity	69	1.69	3.02
EGAN00001617647	EGAN00001617647	Genomic DNA extracted from peripheral white blood cells using two kits, the Qiamp DNA Investigator Kit for coagulated samples and Qiamp DNA Mini & Blood Mini Kit for non-coagulated samples (QIAgen Systems, Inc., Valencia, CA, USA).	Human	blood	NA	male	Iberian Population in Spain	obese	Overweight and obesity	88	3.78	2.73
EGAN00001617648	EGAN00001617648	Genomic DNA extracted from peripheral white blood cells using two kits, the Qiamp DNA Investigator Kit for coagulated samples and Qiamp DNA Mini & Blood Mini Kit for non-coagulated samples (QIAgen Systems, Inc., Valencia, CA, USA).	Human	blood	NA	male	Iberian Population in Spain	obese	Overweight and obesity	81	3.58	2.3
EGAN00001617649	EGAN00001617649	Genomic DNA extracted from peripheral white blood cells using two kits, the Qiamp DNA Investigator Kit for coagulated samples and Qiamp DNA Mini & Blood Mini Kit for non-coagulated samples (QIAgen Systems, Inc., Valencia, CA, USA).	Human	blood	NA	male	Iberian Population in Spain	obese	Overweight and obesity	65	2.34	3.48
EGAN00001617650	EGAN00001617650	Genomic DNA extracted from peripheral white blood cells using two kits, the Qiamp DNA Investigator Kit for coagulated samples and Qiamp DNA Mini & Blood Mini Kit for non-coagulated samples (QIAgen Systems, Inc., Valencia, CA, USA).	Human	blood	NA	male	Iberian Population in Spain	obese	Overweight and obesity	95	7.46	2.71
EGAN00001617651	EGAN00001617651	Genomic DNA extracted from peripheral white blood cells using two kits, the Qiamp DNA Investigator Kit for coagulated samples and Qiamp DNA Mini & Blood Mini Kit for non-coagulated samples (QIAgen Systems, Inc., Valencia, CA, USA).	Human	blood	NA	male	Iberian Population in Spain	obese	Overweight and obesity	75	0.37	4.26
EGAN00001617652	EGAN00001617652	Genomic DNA extracted from peripheral white blood cells using two kits, the Qiamp DNA Investigator Kit for coagulated samples and Qiamp DNA Mini & Blood Mini Kit for non-coagulated samples (QIAgen Systems, Inc., Valencia, CA, USA).	Human	blood	NA	male	Iberian Population in Spain	obese	Overweight and obesity	91	0.45	2.38
EGAN00001617653	EGAN00001617653	Genomic DNA extracted from peripheral white blood cells using two kits, the Qiamp DNA Investigator Kit for coagulated samples and Qiamp DNA Mini & Blood Mini Kit for non-coagulated samples (QIAgen Systems, Inc., Valencia, CA, USA).	Human	blood	NA	male	Iberian Population in Spain	obese	Overweight and obesity	84	2.92	2.32
EGAN00001617654	EGAN00001617654	Genomic DNA extracted from peripheral white blood cells using two kits, the Qiamp DNA Investigator Kit for coagulated samples and Qiamp DNA Mini & Blood Mini Kit for non-coagulated samples (QIAgen Systems, Inc., Valencia, CA, USA).	Human	blood	NA	male	Iberian Population in Spain	obese	Overweight and obesity	105	4.72	6.04
EGAN00001617655	EGAN00001617655	Genomic DNA extracted from peripheral white blood cells using two kits, the Qiamp DNA Investigator Kit for coagulated samples and Qiamp DNA Mini & Blood Mini Kit for non-coagulated samples (QIAgen Systems, Inc., Valencia, CA, USA).	Human	blood	NA	male	Iberian Population in Spain	obese	Overweight and obesity	89	8.7	4.7
EGAN00001617656	EGAN00001617656	Genomic DNA extracted from peripheral white blood cells using two kits, the Qiamp DNA Investigator Kit for coagulated samples and Qiamp DNA Mini & Blood Mini Kit for non-coagulated samples (QIAgen Systems, Inc., Valencia, CA, USA).	Human	blood	NA	male	Iberian Population in Spain	obese	Overweight and obesity	93	7.16	3.52
EGAN00001617657	EGAN00001617657	Genomic DNA extracted from peripheral white blood cells using two kits, the Qiamp DNA Investigator Kit for coagulated samples and Qiamp DNA Mini & Blood Mini Kit for non-coagulated samples (QIAgen Systems, Inc., Valencia, CA, USA).	Human	blood	NA	male	Iberian Population in Spain	obese	Overweight and obesity	83	1.99	2.16
EGAN00001617658	EGAN00001617658	Genomic DNA extracted from peripheral white blood cells using two kits, the Qiamp DNA Investigator Kit for coagulated samples and Qiamp DNA Mini & Blood Mini Kit for non-coagulated samples (QIAgen Systems, Inc., Valencia, CA, USA).	Human	blood	NA	male	Iberian Population in Spain	obese	Overweight and obesity	79	3.51	3.73
EGAN00001617659	EGAN00001617659	Genomic DNA extracted from peripheral white blood cells using two kits, the Qiamp DNA Investigator Kit for coagulated samples and Qiamp DNA Mini & Blood Mini Kit for non-coagulated samples (QIAgen Systems, Inc., Valencia, CA, USA).	Human	blood	NA	male	Iberian Population in Spain	obese	Overweight and obesity	82	3.36	3.06
EGAN00001617660	EGAN00001617660	Genomic DNA extracted from peripheral white blood cells using two kits, the Qiamp DNA Investigator Kit for coagulated samples and Qiamp DNA Mini & Blood Mini Kit for non-coagulated samples (QIAgen Systems, Inc., Valencia, CA, USA).	Human	blood	NA	male	Iberian Population in Spain	obese	Overweight and obesity	88	1.28	5.06
EGAN00001617661	EGAN00001617661	Genomic DNA extracted from peripheral white blood cells using two kits, the Qiamp DNA Investigator Kit for coagulated samples and Qiamp DNA Mini & Blood Mini Kit for non-coagulated samples (QIAgen Systems, Inc., Valencia, CA, USA).	Human	blood	NA	male	Iberian Population in Spain	obese	Overweight and obesity	85	0.8	4.01
EGAN00001617662	EGAN00001617662	Genomic DNA extracted from peripheral white blood cells using two kits, the Qiamp DNA Investigator Kit for coagulated samples and Qiamp DNA Mini & Blood Mini Kit for non-coagulated samples (QIAgen Systems, Inc., Valencia, CA, USA).	Human	blood	NA	male	Iberian Population in Spain	obese	Overweight and obesity	82	NA	2.55
EGAN00001617663	EGAN00001617663	Genomic DNA extracted from peripheral white blood cells using two kits, the Qiamp DNA Investigator Kit for coagulated samples and Qiamp DNA Mini & Blood Mini Kit for non-coagulated samples (QIAgen Systems, Inc., Valencia, CA, USA).	Human	blood	NA	male	Iberian Population in Spain	overweight	Overweight and obesity	71	1.96	1.46
EGAN00001617664	EGAN00001617664	Genomic DNA extracted from peripheral white blood cells using two kits, the Qiamp DNA Investigator Kit for coagulated samples and Qiamp DNA Mini & Blood Mini Kit for non-coagulated samples (QIAgen Systems, Inc., Valencia, CA, USA).	Human	blood	NA	male	Iberian Population in Spain	obese	Overweight and obesity	96	2.3	6.91
EGAN00001617665	EGAN00001617665	Genomic DNA extracted from peripheral white blood cells using two kits, the Qiamp DNA Investigator Kit for coagulated samples and Qiamp DNA Mini & Blood Mini Kit for non-coagulated samples (QIAgen Systems, Inc., Valencia, CA, USA).	Human	blood	NA	male	Iberian Population in Spain	overweight	Overweight and obesity	85	0.63	1.29
EGAN00001617666	EGAN00001617666	Genomic DNA extracted from peripheral white blood cells using two kits, the Qiamp DNA Investigator Kit for coagulated samples and Qiamp DNA Mini & Blood Mini Kit for non-coagulated samples (QIAgen Systems, Inc., Valencia, CA, USA).	Human	blood	NA	male	Iberian Population in Spain	obese	Overweight and obesity	77	NA	2.43
EGAN00001617667	EGAN00001617667	Genomic DNA extracted from peripheral white blood cells using two kits, the Qiamp DNA Investigator Kit for coagulated samples and Qiamp DNA Mini & Blood Mini Kit for non-coagulated samples (QIAgen Systems, Inc., Valencia, CA, USA).	Human	blood	NA	male	Iberian Population in Spain	overweight	Overweight and obesity	78	0.48	1.99
EGAN00001617668	EGAN00001617668	Genomic DNA extracted from peripheral white blood cells using two kits, the Qiamp DNA Investigator Kit for coagulated samples and Qiamp DNA Mini & Blood Mini Kit for non-coagulated samples (QIAgen Systems, Inc., Valencia, CA, USA).	Human	blood	NA	male	Iberian Population in Spain	obese	Overweight and obesity	91	3.3	3.18
EGAN00001617669	EGAN00001617669	Genomic DNA extracted from peripheral white blood cells using two kits, the Qiamp DNA Investigator Kit for coagulated samples and Qiamp DNA Mini & Blood Mini Kit for non-coagulated samples (QIAgen Systems, Inc., Valencia, CA, USA).	Human	blood	NA	male	Iberian Population in Spain	overweight	Overweight and obesity	76	5.74	1.93
EGAN00001617670	EGAN00001617670	Genomic DNA extracted from peripheral white blood cells using two kits, the Qiamp DNA Investigator Kit for coagulated samples and Qiamp DNA Mini & Blood Mini Kit for non-coagulated samples (QIAgen Systems, Inc., Valencia, CA, USA).	Human	blood	NA	male	Iberian Population in Spain	obese	Overweight and obesity	86	2.02	4.36
EGAN00001617671	EGAN00001617671	Genomic DNA extracted from peripheral white blood cells using two kits, the Qiamp DNA Investigator Kit for coagulated samples and Qiamp DNA Mini & Blood Mini Kit for non-coagulated samples (QIAgen Systems, Inc., Valencia, CA, USA).	Human	blood	NA	male	Iberian Population in Spain	obese	Overweight and obesity	82	3.89	5.35
EGAN00001617672	EGAN00001617672	Genomic DNA extracted from peripheral white blood cells using two kits, the Qiamp DNA Investigator Kit for coagulated samples and Qiamp DNA Mini & Blood Mini Kit for non-coagulated samples (QIAgen Systems, Inc., Valencia, CA, USA).	Human	blood	NA	male	Iberian Population in Spain	overweight	Overweight and obesity	80	0.67	1.5
EGAN00001617673	EGAN00001617673	Genomic DNA extracted from peripheral white blood cells using two kits, the Qiamp DNA Investigator Kit for coagulated samples and Qiamp DNA Mini & Blood Mini Kit for non-coagulated samples (QIAgen Systems, Inc., Valencia, CA, USA).	Human	blood	NA	male	Iberian Population in Spain	obese	Overweight and obesity	92	NA	2.35
EGAN00001617674	EGAN00001617674	Genomic DNA extracted from peripheral white blood cells using two kits, the Qiamp DNA Investigator Kit for coagulated samples and Qiamp DNA Mini & Blood Mini Kit for non-coagulated samples (QIAgen Systems, Inc., Valencia, CA, USA).	Human	blood	NA	male	Iberian Population in Spain	overweight	Overweight and obesity	74	0.66	1.7
EGAN00001617675	EGAN00001617675	Genomic DNA extracted from peripheral white blood cells using two kits, the Qiamp DNA Investigator Kit for coagulated samples and Qiamp DNA Mini & Blood Mini Kit for non-coagulated samples (QIAgen Systems, Inc., Valencia, CA, USA).	Human	blood	NA	male	Iberian Population in Spain	obese	Overweight and obesity	87	4.12	2.42
EGAN00001617676	EGAN00001617676	Genomic DNA extracted from peripheral white blood cells using two kits, the Qiamp DNA Investigator Kit for coagulated samples and Qiamp DNA Mini & Blood Mini Kit for non-coagulated samples (QIAgen Systems, Inc., Valencia, CA, USA).	Human	blood	NA	male	Iberian Population in Spain	overweight	Overweight and obesity	80	1.34	1.95
EGAN00001617677	EGAN00001617677	Genomic DNA extracted from peripheral white blood cells using two kits, the Qiamp DNA Investigator Kit for coagulated samples and Qiamp DNA Mini & Blood Mini Kit for non-coagulated samples (QIAgen Systems, Inc., Valencia, CA, USA).	Human	blood	NA	male	Iberian Population in Spain	obese	Overweight and obesity	78	3.93	2.28
EGAN00001617678	EGAN00001617678	Genomic DNA extracted from peripheral white blood cells using two kits, the Qiamp DNA Investigator Kit for coagulated samples and Qiamp DNA Mini & Blood Mini Kit for non-coagulated samples (QIAgen Systems, Inc., Valencia, CA, USA).	Human	blood	NA	male	Iberian Population in Spain	overweight	Overweight and obesity	76	1.16	1.47
EGAN00001617679	EGAN00001617679	Genomic DNA extracted from peripheral white blood cells using two kits, the Qiamp DNA Investigator Kit for coagulated samples and Qiamp DNA Mini & Blood Mini Kit for non-coagulated samples (QIAgen Systems, Inc., Valencia, CA, USA).	Human	blood	NA	male	Iberian Population in Spain	obese	Overweight and obesity	79	2.89	2.46
EGAN00001617680	EGAN00001617680	Genomic DNA extracted from peripheral white blood cells using two kits, the Qiamp DNA Investigator Kit for coagulated samples and Qiamp DNA Mini & Blood Mini Kit for non-coagulated samples (QIAgen Systems, Inc., Valencia, CA, USA).	Human	blood	NA	male	Iberian Population in Spain	overweight	Overweight and obesity	79	0.86	1.99
EGAN00001617681	EGAN00001617681	Genomic DNA extracted from peripheral white blood cells using two kits, the Qiamp DNA Investigator Kit for coagulated samples and Qiamp DNA Mini & Blood Mini Kit for non-coagulated samples (QIAgen Systems, Inc., Valencia, CA, USA).	Human	blood	NA	male	Iberian Population in Spain	obese	Overweight and obesity	71	0.33	4.74
EGAN00001617682	EGAN00001617682	Genomic DNA extracted from peripheral white blood cells using two kits, the Qiamp DNA Investigator Kit for coagulated samples and Qiamp DNA Mini & Blood Mini Kit for non-coagulated samples (QIAgen Systems, Inc., Valencia, CA, USA).	Human	blood	NA	male	Iberian Population in Spain	obese	Overweight and obesity	81	2.18	3.27
EGAN00001617683	EGAN00001617683	Genomic DNA extracted from peripheral white blood cells using two kits, the Qiamp DNA Investigator Kit for coagulated samples and Qiamp DNA Mini & Blood Mini Kit for non-coagulated samples (QIAgen Systems, Inc., Valencia, CA, USA).	Human	blood	NA	male	Iberian Population in Spain	overweight	Overweight and obesity	84	NA	0.96
EGAN00001617684	EGAN00001617684	Genomic DNA extracted from peripheral white blood cells using two kits, the Qiamp DNA Investigator Kit for coagulated samples and Qiamp DNA Mini & Blood Mini Kit for non-coagulated samples (QIAgen Systems, Inc., Valencia, CA, USA).	Human	blood	NA	male	Iberian Population in Spain	obese	Overweight and obesity	78	0.39	4.14
EGAN00001617685	EGAN00001617685	Genomic DNA extracted from peripheral white blood cells using two kits, the Qiamp DNA Investigator Kit for coagulated samples and Qiamp DNA Mini & Blood Mini Kit for non-coagulated samples (QIAgen Systems, Inc., Valencia, CA, USA).	Human	blood	NA	male	Iberian Population in Spain	obese	Overweight and obesity	75	0.35	2.16
EGAN00001617686	EGAN00001617686	Genomic DNA extracted from peripheral white blood cells using two kits, the Qiamp DNA Investigator Kit for coagulated samples and Qiamp DNA Mini & Blood Mini Kit for non-coagulated samples (QIAgen Systems, Inc., Valencia, CA, USA).	Human	blood	NA	male	Iberian Population in Spain	obese	Overweight and obesity	93	5.12	2.98
EGAN00001617687	EGAN00001617687	Genomic DNA extracted from peripheral white blood cells using two kits, the Qiamp DNA Investigator Kit for coagulated samples and Qiamp DNA Mini & Blood Mini Kit for non-coagulated samples (QIAgen Systems, Inc., Valencia, CA, USA).	Human	blood	NA	male	Iberian Population in Spain	overweight	Overweight and obesity	93	4.64	1.59
EGAN00001617688	EGAN00001617688	Genomic DNA extracted from peripheral white blood cells using two kits, the Qiamp DNA Investigator Kit for coagulated samples and Qiamp DNA Mini & Blood Mini Kit for non-coagulated samples (QIAgen Systems, Inc., Valencia, CA, USA).	Human	blood	NA	male	Iberian Population in Spain	obese	Overweight and obesity	88	NA	3.45
EGAN00001617689	EGAN00001617689	Genomic DNA extracted from peripheral white blood cells using two kits, the Qiamp DNA Investigator Kit for coagulated samples and Qiamp DNA Mini & Blood Mini Kit for non-coagulated samples (QIAgen Systems, Inc., Valencia, CA, USA).	Human	blood	NA	male	Iberian Population in Spain	obese	Overweight and obesity	85	1.62	9.81
EGAN00001617690	EGAN00001617690	Genomic DNA extracted from peripheral white blood cells using two kits, the Qiamp DNA Investigator Kit for coagulated samples and Qiamp DNA Mini & Blood Mini Kit for non-coagulated samples (QIAgen Systems, Inc., Valencia, CA, USA).	Human	blood	NA	male	Iberian Population in Spain	obese	Overweight and obesity	83	0.59	4.2
EGAN00001617691	EGAN00001617691	Genomic DNA extracted from peripheral white blood cells using two kits, the Qiamp DNA Investigator Kit for coagulated samples and Qiamp DNA Mini & Blood Mini Kit for non-coagulated samples (QIAgen Systems, Inc., Valencia, CA, USA).	Human	blood	NA	male	Iberian Population in Spain	obese	Overweight and obesity	75	2.63	4.06
EGAN00001617692	EGAN00001617692	Genomic DNA extracted from peripheral white blood cells using two kits, the Qiamp DNA Investigator Kit for coagulated samples and Qiamp DNA Mini & Blood Mini Kit for non-coagulated samples (QIAgen Systems, Inc., Valencia, CA, USA).	Human	blood	NA	male	Iberian Population in Spain	obese	Overweight and obesity	79	3.86	2.8
EGAN00001617693	EGAN00001617693	Genomic DNA extracted from peripheral white blood cells using two kits, the Qiamp DNA Investigator Kit for coagulated samples and Qiamp DNA Mini & Blood Mini Kit for non-coagulated samples (QIAgen Systems, Inc., Valencia, CA, USA).	Human	blood	NA	male	Iberian Population in Spain	overweight	Overweight and obesity	83	NA	1.93
EGAN00001617694	EGAN00001617694	Genomic DNA extracted from peripheral white blood cells using two kits, the Qiamp DNA Investigator Kit for coagulated samples and Qiamp DNA Mini & Blood Mini Kit for non-coagulated samples (QIAgen Systems, Inc., Valencia, CA, USA).	Human	blood	NA	male	Iberian Population in Spain	obese	Overweight and obesity	77	1.18	4.7
EGAN00001617695	EGAN00001617695	Genomic DNA extracted from peripheral white blood cells using two kits, the Qiamp DNA Investigator Kit for coagulated samples and Qiamp DNA Mini & Blood Mini Kit for non-coagulated samples (QIAgen Systems, Inc., Valencia, CA, USA).	Human	blood	NA	male	Iberian Population in Spain	obese	Overweight and obesity	73	NA	2.15
EGAN00001617696	EGAN00001617696	Genomic DNA extracted from peripheral white blood cells using two kits, the Qiamp DNA Investigator Kit for coagulated samples and Qiamp DNA Mini & Blood Mini Kit for non-coagulated samples (QIAgen Systems, Inc., Valencia, CA, USA).	Human	blood	NA	male	Iberian Population in Spain	obese	Overweight and obesity	82	NA	11.47
EGAN00001617697	EGAN00001617697	Genomic DNA extracted from peripheral white blood cells using two kits, the Qiamp DNA Investigator Kit for coagulated samples and Qiamp DNA Mini & Blood Mini Kit for non-coagulated samples (QIAgen Systems, Inc., Valencia, CA, USA).	Human	blood	NA	male	Iberian Population in Spain	obese	Overweight and obesity	73	1.3	2.23
EGAN00001617698	EGAN00001617698	Genomic DNA extracted from peripheral white blood cells using two kits, the Qiamp DNA Investigator Kit for coagulated samples and Qiamp DNA Mini & Blood Mini Kit for non-coagulated samples (QIAgen Systems, Inc., Valencia, CA, USA).	Human	blood	NA	male	Iberian Population in Spain	obese	Overweight and obesity	94	8.33	3.76
EGAN00001617699	EGAN00001617699	Genomic DNA extracted from peripheral white blood cells using two kits, the Qiamp DNA Investigator Kit for coagulated samples and Qiamp DNA Mini & Blood Mini Kit for non-coagulated samples (QIAgen Systems, Inc., Valencia, CA, USA).	Human	blood	NA	male	Iberian Population in Spain	obese	Overweight and obesity	73	0.34	2.5
EGAN00001617700	EGAN00001617700	Genomic DNA extracted from peripheral white blood cells using two kits, the Qiamp DNA Investigator Kit for coagulated samples and Qiamp DNA Mini & Blood Mini Kit for non-coagulated samples (QIAgen Systems, Inc., Valencia, CA, USA).	Human	blood	NA	male	Iberian Population in Spain	obese	Overweight and obesity	72	0.39	5.06
EGAN00001617701	EGAN00001617701	Genomic DNA extracted from peripheral white blood cells using two kits, the Qiamp DNA Investigator Kit for coagulated samples and Qiamp DNA Mini & Blood Mini Kit for non-coagulated samples (QIAgen Systems, Inc., Valencia, CA, USA).	Human	blood	NA	male	Iberian Population in Spain	obese	Overweight and obesity	73	1.23	2.96
EGAN00001617702	EGAN00001617702	Genomic DNA extracted from peripheral white blood cells using two kits, the Qiamp DNA Investigator Kit for coagulated samples and Qiamp DNA Mini & Blood Mini Kit for non-coagulated samples (QIAgen Systems, Inc., Valencia, CA, USA).	Human	blood	NA	male	Iberian Population in Spain	obese	Overweight and obesity	69	0.89	2.68
EGAN00001617703	EGAN00001617703	Genomic DNA extracted from peripheral white blood cells using two kits, the Qiamp DNA Investigator Kit for coagulated samples and Qiamp DNA Mini & Blood Mini Kit for non-coagulated samples (QIAgen Systems, Inc., Valencia, CA, USA).	Human	blood	NA	male	Iberian Population in Spain	overweight	Overweight and obesity	91	1.82	1.97
EGAN00001617704	EGAN00001617704	Genomic DNA extracted from peripheral white blood cells using two kits, the Qiamp DNA Investigator Kit for coagulated samples and Qiamp DNA Mini & Blood Mini Kit for non-coagulated samples (QIAgen Systems, Inc., Valencia, CA, USA).	Human	blood	NA	male	Iberian Population in Spain	obese	Overweight and obesity	74	0.79	3.21
EGAN00001617705	EGAN00001617705	Genomic DNA extracted from peripheral white blood cells using two kits, the Qiamp DNA Investigator Kit for coagulated samples and Qiamp DNA Mini & Blood Mini Kit for non-coagulated samples (QIAgen Systems, Inc., Valencia, CA, USA).	Human	blood	NA	male	Iberian Population in Spain	obese	Overweight and obesity	68	0.47	2.36
EGAN00001617706	EGAN00001617706	Genomic DNA extracted from peripheral white blood cells using two kits, the Qiamp DNA Investigator Kit for coagulated samples and Qiamp DNA Mini & Blood Mini Kit for non-coagulated samples (QIAgen Systems, Inc., Valencia, CA, USA).	Human	blood	NA	male	Iberian Population in Spain	obese	Overweight and obesity	99	2.66	3.38
EGAN00001617707	EGAN00001617707	Genomic DNA extracted from peripheral white blood cells using two kits, the Qiamp DNA Investigator Kit for coagulated samples and Qiamp DNA Mini & Blood Mini Kit for non-coagulated samples (QIAgen Systems, Inc., Valencia, CA, USA).	Human	blood	NA	male	Iberian Population in Spain	obese	Overweight and obesity	78	4.74	3.42
EGAN00001617708	EGAN00001617708	Genomic DNA extracted from peripheral white blood cells using two kits, the Qiamp DNA Investigator Kit for coagulated samples and Qiamp DNA Mini & Blood Mini Kit for non-coagulated samples (QIAgen Systems, Inc., Valencia, CA, USA).	Human	blood	NA	male	Iberian Population in Spain	obese	Overweight and obesity	83	2.77	3.59
EGAN00001617709	EGAN00001617709	Genomic DNA extracted from peripheral white blood cells using two kits, the Qiamp DNA Investigator Kit for coagulated samples and Qiamp DNA Mini & Blood Mini Kit for non-coagulated samples (QIAgen Systems, Inc., Valencia, CA, USA).	Human	blood	NA	male	Iberian Population in Spain	obese	Overweight and obesity	85	1.43	2.12
EGAN00001617710	EGAN00001617710	Genomic DNA extracted from peripheral white blood cells using two kits, the Qiamp DNA Investigator Kit for coagulated samples and Qiamp DNA Mini & Blood Mini Kit for non-coagulated samples (QIAgen Systems, Inc., Valencia, CA, USA).	Human	blood	NA	male	Iberian Population in Spain	obese	Overweight and obesity	83	0.39	2.14
EGAN00001617711	EGAN00001617711	Genomic DNA extracted from peripheral white blood cells using two kits, the Qiamp DNA Investigator Kit for coagulated samples and Qiamp DNA Mini & Blood Mini Kit for non-coagulated samples (QIAgen Systems, Inc., Valencia, CA, USA).	Human	blood	NA	male	Iberian Population in Spain	obese	Overweight and obesity	82	1.72	2.99
EGAN00001617712	EGAN00001617712	Genomic DNA extracted from peripheral white blood cells using two kits, the Qiamp DNA Investigator Kit for coagulated samples and Qiamp DNA Mini & Blood Mini Kit for non-coagulated samples (QIAgen Systems, Inc., Valencia, CA, USA).	Human	blood	NA	male	Iberian Population in Spain	obese	Overweight and obesity	80	1.22	4.23
EGAN00001617713	EGAN00001617713	Genomic DNA extracted from peripheral white blood cells using two kits, the Qiamp DNA Investigator Kit for coagulated samples and Qiamp DNA Mini & Blood Mini Kit for non-coagulated samples (QIAgen Systems, Inc., Valencia, CA, USA).	Human	blood	NA	male	Iberian Population in Spain	overweight	Overweight and obesity	87	0.93	1.36
EGAN00001617714	EGAN00001617714	Genomic DNA extracted from peripheral white blood cells using two kits, the Qiamp DNA Investigator Kit for coagulated samples and Qiamp DNA Mini & Blood Mini Kit for non-coagulated samples (QIAgen Systems, Inc., Valencia, CA, USA).	Human	blood	NA	male	Iberian Population in Spain	obese	Overweight and obesity	87	3.5	9.87
EGAN00001617715	EGAN00001617715	Genomic DNA extracted from peripheral white blood cells using two kits, the Qiamp DNA Investigator Kit for coagulated samples and Qiamp DNA Mini & Blood Mini Kit for non-coagulated samples (QIAgen Systems, Inc., Valencia, CA, USA).	Human	blood	NA	male	Iberian Population in Spain	obese	Overweight and obesity	83	3.81	3.09
EGAN00001617716	EGAN00001617716	Genomic DNA extracted from peripheral white blood cells using two kits, the Qiamp DNA Investigator Kit for coagulated samples and Qiamp DNA Mini & Blood Mini Kit for non-coagulated samples (QIAgen Systems, Inc., Valencia, CA, USA).	Human	blood	NA	male	Iberian Population in Spain	obese	Overweight and obesity	98	2.18	3.36
EGAN00001617717	EGAN00001617717	Genomic DNA extracted from peripheral white blood cells using two kits, the Qiamp DNA Investigator Kit for coagulated samples and Qiamp DNA Mini & Blood Mini Kit for non-coagulated samples (QIAgen Systems, Inc., Valencia, CA, USA).	Human	blood	NA	male	Iberian Population in Spain	obese	Overweight and obesity	82	1.15	2.94
EGAN00001617718	EGAN00001617718	Genomic DNA extracted from peripheral white blood cells using two kits, the Qiamp DNA Investigator Kit for coagulated samples and Qiamp DNA Mini & Blood Mini Kit for non-coagulated samples (QIAgen Systems, Inc., Valencia, CA, USA).	Human	blood	NA	male	Iberian Population in Spain	obese	Overweight and obesity	88	2.24	5.77
EGAN00001617719	EGAN00001617719	Genomic DNA extracted from peripheral white blood cells using two kits, the Qiamp DNA Investigator Kit for coagulated samples and Qiamp DNA Mini & Blood Mini Kit for non-coagulated samples (QIAgen Systems, Inc., Valencia, CA, USA).	Human	blood	NA	male	Iberian Population in Spain	overweight	Overweight and obesity	90	3.76	1.04
EGAN00001617720	EGAN00001617720	Genomic DNA extracted from peripheral white blood cells using two kits, the Qiamp DNA Investigator Kit for coagulated samples and Qiamp DNA Mini & Blood Mini Kit for non-coagulated samples (QIAgen Systems, Inc., Valencia, CA, USA).	Human	blood	NA	male	Iberian Population in Spain	obese	Overweight and obesity	84	3.04	2.62
EGAN00001617721	EGAN00001617721	Genomic DNA extracted from peripheral white blood cells using two kits, the Qiamp DNA Investigator Kit for coagulated samples and Qiamp DNA Mini & Blood Mini Kit for non-coagulated samples (QIAgen Systems, Inc., Valencia, CA, USA).	Human	blood	NA	male	Iberian Population in Spain	obese	Overweight and obesity	85	3.71	3.59
EGAN00001617722	EGAN00001617722	Genomic DNA extracted from peripheral white blood cells using two kits, the Qiamp DNA Investigator Kit for coagulated samples and Qiamp DNA Mini & Blood Mini Kit for non-coagulated samples (QIAgen Systems, Inc., Valencia, CA, USA).	Human	blood	NA	male	Iberian Population in Spain	overweight	Overweight and obesity	88	2.33	1.66
EGAN00001617723	EGAN00001617723	Genomic DNA extracted from peripheral white blood cells using two kits, the Qiamp DNA Investigator Kit for coagulated samples and Qiamp DNA Mini & Blood Mini Kit for non-coagulated samples (QIAgen Systems, Inc., Valencia, CA, USA).	Human	blood	NA	male	Iberian Population in Spain	overweight	Overweight and obesity	85	2.29	0.56
EGAN00001617724	EGAN00001617724	Genomic DNA extracted from peripheral white blood cells using two kits, the Qiamp DNA Investigator Kit for coagulated samples and Qiamp DNA Mini & Blood Mini Kit for non-coagulated samples (QIAgen Systems, Inc., Valencia, CA, USA).	Human	blood	NA	male	Iberian Population in Spain	overweight	Overweight and obesity	85	0.97	1.89
EGAN00001617725	EGAN00001617725	Genomic DNA extracted from peripheral white blood cells using two kits, the Qiamp DNA Investigator Kit for coagulated samples and Qiamp DNA Mini & Blood Mini Kit for non-coagulated samples (QIAgen Systems, Inc., Valencia, CA, USA).	Human	blood	NA	male	Iberian Population in Spain	obese	Overweight and obesity	78	3.14	3.49
EGAN00001617726	EGAN00001617726	Genomic DNA extracted from peripheral white blood cells using two kits, the Qiamp DNA Investigator Kit for coagulated samples and Qiamp DNA Mini & Blood Mini Kit for non-coagulated samples (QIAgen Systems, Inc., Valencia, CA, USA).	Human	blood	NA	male	Iberian Population in Spain	obese	Overweight and obesity	86	2.93	2.33
EGAN00001617727	EGAN00001617727	Genomic DNA extracted from peripheral white blood cells using two kits, the Qiamp DNA Investigator Kit for coagulated samples and Qiamp DNA Mini & Blood Mini Kit for non-coagulated samples (QIAgen Systems, Inc., Valencia, CA, USA).	Human	blood	NA	male	Iberian Population in Spain	overweight	Overweight and obesity	88	2.59	1.35
EGAN00001617728	EGAN00001617728	Genomic DNA extracted from peripheral white blood cells using two kits, the Qiamp DNA Investigator Kit for coagulated samples and Qiamp DNA Mini & Blood Mini Kit for non-coagulated samples (QIAgen Systems, Inc., Valencia, CA, USA).	Human	blood	NA	male	Iberian Population in Spain	obese	Overweight and obesity	80	NA	10.44
EGAN00001617729	EGAN00001617729	Genomic DNA extracted from peripheral white blood cells using two kits, the Qiamp DNA Investigator Kit for coagulated samples and Qiamp DNA Mini & Blood Mini Kit for non-coagulated samples (QIAgen Systems, Inc., Valencia, CA, USA).	Human	blood	NA	male	Iberian Population in Spain	obese	Overweight and obesity	105	3.77	2.23
EGAN00001617730	EGAN00001617730	Genomic DNA extracted from peripheral white blood cells using two kits, the Qiamp DNA Investigator Kit for coagulated samples and Qiamp DNA Mini & Blood Mini Kit for non-coagulated samples (QIAgen Systems, Inc., Valencia, CA, USA).	Human	blood	NA	male	Iberian Population in Spain	obese	Overweight and obesity	85	3.33	2.54
EGAN00001617731	EGAN00001617731	Genomic DNA extracted from peripheral white blood cells using two kits, the Qiamp DNA Investigator Kit for coagulated samples and Qiamp DNA Mini & Blood Mini Kit for non-coagulated samples (QIAgen Systems, Inc., Valencia, CA, USA).	Human	blood	NA	male	Iberian Population in Spain	overweight	Overweight and obesity	79	1.73	1.8
EGAN00001617732	EGAN00001617732	Genomic DNA extracted from peripheral white blood cells using two kits, the Qiamp DNA Investigator Kit for coagulated samples and Qiamp DNA Mini & Blood Mini Kit for non-coagulated samples (QIAgen Systems, Inc., Valencia, CA, USA).	Human	blood	NA	male	Iberian Population in Spain	overweight	Overweight and obesity	86	1.18	1.49
EGAN00001617733	EGAN00001617733	Genomic DNA extracted from peripheral white blood cells using two kits, the Qiamp DNA Investigator Kit for coagulated samples and Qiamp DNA Mini & Blood Mini Kit for non-coagulated samples (QIAgen Systems, Inc., Valencia, CA, USA).	Human	blood	NA	male	Iberian Population in Spain	obese	Overweight and obesity	93	2.83	4.83
EGAN00001617734	EGAN00001617734	Genomic DNA extracted from peripheral white blood cells using two kits, the Qiamp DNA Investigator Kit for coagulated samples and Qiamp DNA Mini & Blood Mini Kit for non-coagulated samples (QIAgen Systems, Inc., Valencia, CA, USA).	Human	blood	NA	male	Iberian Population in Spain	obese	Overweight and obesity	87	2.57	4.11
EGAN00001617735	EGAN00001617735	Genomic DNA extracted from peripheral white blood cells using two kits, the Qiamp DNA Investigator Kit for coagulated samples and Qiamp DNA Mini & Blood Mini Kit for non-coagulated samples (QIAgen Systems, Inc., Valencia, CA, USA).	Human	blood	NA	male	Iberian Population in Spain	obese	Overweight and obesity	93	3.04	2.19
EGAN00001617736	EGAN00001617736	Genomic DNA extracted from peripheral white blood cells using two kits, the Qiamp DNA Investigator Kit for coagulated samples and Qiamp DNA Mini & Blood Mini Kit for non-coagulated samples (QIAgen Systems, Inc., Valencia, CA, USA).	Human	blood	NA	male	Iberian Population in Spain	obese	Overweight and obesity	85	2.56	2.12
EGAN00001617737	EGAN00001617737	Genomic DNA extracted from peripheral white blood cells using two kits, the Qiamp DNA Investigator Kit for coagulated samples and Qiamp DNA Mini & Blood Mini Kit for non-coagulated samples (QIAgen Systems, Inc., Valencia, CA, USA).	Human	blood	NA	male	Iberian Population in Spain	overweight	Overweight and obesity	90	5.17	0.61
EGAN00001617738	EGAN00001617738	Genomic DNA extracted from peripheral white blood cells using two kits, the Qiamp DNA Investigator Kit for coagulated samples and Qiamp DNA Mini & Blood Mini Kit for non-coagulated samples (QIAgen Systems, Inc., Valencia, CA, USA).	Human	blood	NA	male	Iberian Population in Spain	overweight	Overweight and obesity	89	2.43	1.12
EGAN00001617739	EGAN00001617739	Genomic DNA extracted from peripheral white blood cells using two kits, the Qiamp DNA Investigator Kit for coagulated samples and Qiamp DNA Mini & Blood Mini Kit for non-coagulated samples (QIAgen Systems, Inc., Valencia, CA, USA).	Human	blood	NA	male	Iberian Population in Spain	obese	Overweight and obesity	109	4.02	4.07
EGAN00001617740	EGAN00001617740	Genomic DNA extracted from peripheral white blood cells using two kits, the Qiamp DNA Investigator Kit for coagulated samples and Qiamp DNA Mini & Blood Mini Kit for non-coagulated samples (QIAgen Systems, Inc., Valencia, CA, USA).	Human	blood	NA	male	Iberian Population in Spain	overweight	Overweight and obesity	92	2.25	1.03
EGAN00001617741	EGAN00001617741	Genomic DNA extracted from peripheral white blood cells using two kits, the Qiamp DNA Investigator Kit for coagulated samples and Qiamp DNA Mini & Blood Mini Kit for non-coagulated samples (QIAgen Systems, Inc., Valencia, CA, USA).	Human	blood	NA	male	Iberian Population in Spain	obese	Overweight and obesity	99	5.16	2.4
EGAN00001617742	EGAN00001617742	Genomic DNA extracted from peripheral white blood cells using two kits, the Qiamp DNA Investigator Kit for coagulated samples and Qiamp DNA Mini & Blood Mini Kit for non-coagulated samples (QIAgen Systems, Inc., Valencia, CA, USA).	Human	blood	NA	male	Iberian Population in Spain	obese	Overweight and obesity	101	3.92	2.46
EGAN00001617743	EGAN00001617743	Genomic DNA extracted from peripheral white blood cells using two kits, the Qiamp DNA Investigator Kit for coagulated samples and Qiamp DNA Mini & Blood Mini Kit for non-coagulated samples (QIAgen Systems, Inc., Valencia, CA, USA).	Human	blood	NA	male	Iberian Population in Spain	obese	Overweight and obesity	95	7.01	3.18
EGAN00001617744	EGAN00001617744	Genomic DNA extracted from peripheral white blood cells using two kits, the Qiamp DNA Investigator Kit for coagulated samples and Qiamp DNA Mini & Blood Mini Kit for non-coagulated samples (QIAgen Systems, Inc., Valencia, CA, USA).	Human	blood	NA	male	Iberian Population in Spain	obese	Overweight and obesity	79	5.06	4.69
EGAN00001617745	EGAN00001617745	Genomic DNA extracted from peripheral white blood cells using two kits, the Qiamp DNA Investigator Kit for coagulated samples and Qiamp DNA Mini & Blood Mini Kit for non-coagulated samples (QIAgen Systems, Inc., Valencia, CA, USA).	Human	blood	NA	male	Iberian Population in Spain	obese	Overweight and obesity	94	6.06	2.57
EGAN00001617746	EGAN00001617746	Genomic DNA extracted from peripheral white blood cells using two kits, the Qiamp DNA Investigator Kit for coagulated samples and Qiamp DNA Mini & Blood Mini Kit for non-coagulated samples (QIAgen Systems, Inc., Valencia, CA, USA).	Human	blood	NA	male	Iberian Population in Spain	overweight	Overweight and obesity	95	2.91	1.64
EGAN00001617747	EGAN00001617747	Genomic DNA extracted from peripheral white blood cells using two kits, the Qiamp DNA Investigator Kit for coagulated samples and Qiamp DNA Mini & Blood Mini Kit for non-coagulated samples (QIAgen Systems, Inc., Valencia, CA, USA).	Human	blood	NA	male	Iberian Population in Spain	overweight	Overweight and obesity	85	1.21	0.72
EGAN00001617748	EGAN00001617748	Genomic DNA extracted from peripheral white blood cells using two kits, the Qiamp DNA Investigator Kit for coagulated samples and Qiamp DNA Mini & Blood Mini Kit for non-coagulated samples (QIAgen Systems, Inc., Valencia, CA, USA).	Human	blood	NA	male	Iberian Population in Spain	overweight	Overweight and obesity	84	2.28	1.47
EGAN00001617749	EGAN00001617749	Genomic DNA extracted from peripheral white blood cells using two kits, the Qiamp DNA Investigator Kit for coagulated samples and Qiamp DNA Mini & Blood Mini Kit for non-coagulated samples (QIAgen Systems, Inc., Valencia, CA, USA).	Human	blood	NA	male	Iberian Population in Spain	overweight	Overweight and obesity	95	7.66	1.49
EGAN00001617750	EGAN00001617750	Genomic DNA extracted from peripheral white blood cells using two kits, the Qiamp DNA Investigator Kit for coagulated samples and Qiamp DNA Mini & Blood Mini Kit for non-coagulated samples (QIAgen Systems, Inc., Valencia, CA, USA).	Human	blood	NA	male	Iberian Population in Spain	overweight	Overweight and obesity	66	1,483	2.16
EGAN00001617751	EGAN00001617751	Genomic DNA extracted from peripheral white blood cells using two kits, the Qiamp DNA Investigator Kit for coagulated samples and Qiamp DNA Mini & Blood Mini Kit for non-coagulated samples (QIAgen Systems, Inc., Valencia, CA, USA).	Human	blood	NA	male	Iberian Population in Spain	obese	Overweight and obesity	106	4,633	3.16
EGAN00001617752	EGAN00001617752	Genomic DNA extracted from peripheral white blood cells using two kits, the Qiamp DNA Investigator Kit for coagulated samples and Qiamp DNA Mini & Blood Mini Kit for non-coagulated samples (QIAgen Systems, Inc., Valencia, CA, USA).	Human	blood	NA	male	Iberian Population in Spain	obese	Overweight and obesity	94	1,485	3.69
EGAN00001617753	EGAN00001617753	Genomic DNA extracted from peripheral white blood cells using two kits, the Qiamp DNA Investigator Kit for coagulated samples and Qiamp DNA Mini & Blood Mini Kit for non-coagulated samples (QIAgen Systems, Inc., Valencia, CA, USA).	Human	blood	NA	male	Iberian Population in Spain	obese	Overweight and obesity	98	8,905	2.81
EGAN00001617754	EGAN00001617754	Genomic DNA extracted from peripheral white blood cells using two kits, the Qiamp DNA Investigator Kit for coagulated samples and Qiamp DNA Mini & Blood Mini Kit for non-coagulated samples (QIAgen Systems, Inc., Valencia, CA, USA).	Human	blood	NA	male	Iberian Population in Spain	obese	Overweight and obesity	97	NA	2.39
EGAN00001617755	EGAN00001617755	Genomic DNA extracted from peripheral white blood cells using two kits, the Qiamp DNA Investigator Kit for coagulated samples and Qiamp DNA Mini & Blood Mini Kit for non-coagulated samples (QIAgen Systems, Inc., Valencia, CA, USA).	Human	blood	NA	male	Iberian Population in Spain	overweight	Overweight and obesity	86	3,037	1.2
EGAN00001617756	EGAN00001617756	Genomic DNA extracted from peripheral white blood cells using two kits, the Qiamp DNA Investigator Kit for coagulated samples and Qiamp DNA Mini & Blood Mini Kit for non-coagulated samples (QIAgen Systems, Inc., Valencia, CA, USA).	Human	blood	NA	male	Iberian Population in Spain	obese	Overweight and obesity	80	1,837	4.57
EGAN00001617757	EGAN00001617757	Genomic DNA extracted from peripheral white blood cells using two kits, the Qiamp DNA Investigator Kit for coagulated samples and Qiamp DNA Mini & Blood Mini Kit for non-coagulated samples (QIAgen Systems, Inc., Valencia, CA, USA).	Human	blood	NA	male	Iberian Population in Spain	obese	Overweight and obesity	87	5,456	3.05
EGAN00001617758	EGAN00001617758	Genomic DNA extracted from peripheral white blood cells using two kits, the Qiamp DNA Investigator Kit for coagulated samples and Qiamp DNA Mini & Blood Mini Kit for non-coagulated samples (QIAgen Systems, Inc., Valencia, CA, USA).	Human	blood	NA	male	Iberian Population in Spain	obese	Overweight and obesity	103	2,569	2.37
EGAN00001617759	EGAN00001617759	Genomic DNA extracted from peripheral white blood cells using two kits, the Qiamp DNA Investigator Kit for coagulated samples and Qiamp DNA Mini & Blood Mini Kit for non-coagulated samples (QIAgen Systems, Inc., Valencia, CA, USA).	Human	blood	NA	male	Iberian Population in Spain	obese	Overweight and obesity	116	4.44	4.67
EGAN00001617760	EGAN00001617760	Genomic DNA extracted from peripheral white blood cells using two kits, the Qiamp DNA Investigator Kit for coagulated samples and Qiamp DNA Mini & Blood Mini Kit for non-coagulated samples (QIAgen Systems, Inc., Valencia, CA, USA).	Human	blood	NA	male	Iberian Population in Spain	obese	Overweight and obesity	89	4.46	5.38
EGAN00001617761	EGAN00001617761	Genomic DNA extracted from peripheral white blood cells using two kits, the Qiamp DNA Investigator Kit for coagulated samples and Qiamp DNA Mini & Blood Mini Kit for non-coagulated samples (QIAgen Systems, Inc., Valencia, CA, USA).	Human	blood	NA	male	Iberian Population in Spain	obese	Overweight and obesity	100	3.23	5.54
EGAN00001617762	EGAN00001617762	Genomic DNA extracted from peripheral white blood cells using two kits, the Qiamp DNA Investigator Kit for coagulated samples and Qiamp DNA Mini & Blood Mini Kit for non-coagulated samples (QIAgen Systems, Inc., Valencia, CA, USA).	Human	blood	NA	male	Iberian Population in Spain	obese	Overweight and obesity	94	7.05	4.67
EGAN00001617763	EGAN00001617763	Genomic DNA extracted from peripheral white blood cells using two kits, the Qiamp DNA Investigator Kit for coagulated samples and Qiamp DNA Mini & Blood Mini Kit for non-coagulated samples (QIAgen Systems, Inc., Valencia, CA, USA).	Human	blood	NA	male	Iberian Population in Spain	obese	Overweight and obesity	85	2.43	5.24
EGAN00001617764	EGAN00001617764	Genomic DNA extracted from peripheral white blood cells using two kits, the Qiamp DNA Investigator Kit for coagulated samples and Qiamp DNA Mini & Blood Mini Kit for non-coagulated samples (QIAgen Systems, Inc., Valencia, CA, USA).	Human	blood	NA	male	Iberian Population in Spain	obese	Overweight and obesity	77	2.51	4.9
EGAN00001617765	EGAN00001617765	Genomic DNA extracted from peripheral white blood cells using two kits, the Qiamp DNA Investigator Kit for coagulated samples and Qiamp DNA Mini & Blood Mini Kit for non-coagulated samples (QIAgen Systems, Inc., Valencia, CA, USA).	Human	blood	NA	male	Iberian Population in Spain	obese	Overweight and obesity	85	2.2	2.38
EGAN00001617766	EGAN00001617766	Genomic DNA extracted from peripheral white blood cells using two kits, the Qiamp DNA Investigator Kit for coagulated samples and Qiamp DNA Mini & Blood Mini Kit for non-coagulated samples (QIAgen Systems, Inc., Valencia, CA, USA).	Human	blood	NA	male	Iberian Population in Spain	obese	Overweight and obesity	98	2.08	5.04
EGAN00001617767	EGAN00001617767	Genomic DNA extracted from peripheral white blood cells using two kits, the Qiamp DNA Investigator Kit for coagulated samples and Qiamp DNA Mini & Blood Mini Kit for non-coagulated samples (QIAgen Systems, Inc., Valencia, CA, USA).	Human	blood	NA	male	Iberian Population in Spain	obese	Overweight and obesity	84	1.82	2.94
EGAN00001617768	EGAN00001617768	Genomic DNA extracted from peripheral white blood cells using two kits, the Qiamp DNA Investigator Kit for coagulated samples and Qiamp DNA Mini & Blood Mini Kit for non-coagulated samples (QIAgen Systems, Inc., Valencia, CA, USA).	Human	blood	NA	male	Iberian Population in Spain	obese	Overweight and obesity	100	2.27	4.15
EGAN00001617769	EGAN00001617769	Genomic DNA extracted from peripheral white blood cells using two kits, the Qiamp DNA Investigator Kit for coagulated samples and Qiamp DNA Mini & Blood Mini Kit for non-coagulated samples (QIAgen Systems, Inc., Valencia, CA, USA).	Human	blood	NA	male	Iberian Population in Spain	obese	Overweight and obesity	74	1.17	5.9
EGAN00001617770	EGAN00001617770	Genomic DNA extracted from peripheral white blood cells using two kits, the Qiamp DNA Investigator Kit for coagulated samples and Qiamp DNA Mini & Blood Mini Kit for non-coagulated samples (QIAgen Systems, Inc., Valencia, CA, USA).	Human	blood	NA	male	Iberian Population in Spain	obese	Overweight and obesity	75	1.65	4.74
EGAN00001617771	EGAN00001617771	Genomic DNA extracted from peripheral white blood cells using two kits, the Qiamp DNA Investigator Kit for coagulated samples and Qiamp DNA Mini & Blood Mini Kit for non-coagulated samples (QIAgen Systems, Inc., Valencia, CA, USA).	Human	blood	NA	male	Iberian Population in Spain	obese	Overweight and obesity	88	1.61	2.58
EGAN00001617772	EGAN00001617772	Genomic DNA extracted from peripheral white blood cells using two kits, the Qiamp DNA Investigator Kit for coagulated samples and Qiamp DNA Mini & Blood Mini Kit for non-coagulated samples (QIAgen Systems, Inc., Valencia, CA, USA).	Human	blood	NA	male	Iberian Population in Spain	obese	Overweight and obesity	86	1.85	4.57
EGAN00001617773	EGAN00001617773	Genomic DNA extracted from peripheral white blood cells using two kits, the Qiamp DNA Investigator Kit for coagulated samples and Qiamp DNA Mini & Blood Mini Kit for non-coagulated samples (QIAgen Systems, Inc., Valencia, CA, USA).	Human	blood	NA	male	Iberian Population in Spain	obese	Overweight and obesity	73	2.12	6.59
EGAN00001617774	EGAN00001617774	Genomic DNA extracted from peripheral white blood cells using two kits, the Qiamp DNA Investigator Kit for coagulated samples and Qiamp DNA Mini & Blood Mini Kit for non-coagulated samples (QIAgen Systems, Inc., Valencia, CA, USA).	Human	blood	NA	male	Iberian Population in Spain	obese	Overweight and obesity	84	1.51	4.31
EGAN00001617775	EGAN00001617775	Genomic DNA extracted from peripheral white blood cells using two kits, the Qiamp DNA Investigator Kit for coagulated samples and Qiamp DNA Mini & Blood Mini Kit for non-coagulated samples (QIAgen Systems, Inc., Valencia, CA, USA).	Human	blood	NA	male	Iberian Population in Spain	obese	Overweight and obesity	87	1.35	2.09
EGAN00001617776	EGAN00001617776	Genomic DNA extracted from peripheral white blood cells using two kits, the Qiamp DNA Investigator Kit for coagulated samples and Qiamp DNA Mini & Blood Mini Kit for non-coagulated samples (QIAgen Systems, Inc., Valencia, CA, USA).	Human	blood	NA	male	Iberian Population in Spain	obese	Overweight and obesity	78	NA	5.32
EGAN00001617777	EGAN00001617777	Genomic DNA extracted from peripheral white blood cells using two kits, the Qiamp DNA Investigator Kit for coagulated samples and Qiamp DNA Mini & Blood Mini Kit for non-coagulated samples (QIAgen Systems, Inc., Valencia, CA, USA).	Human	blood	NA	male	Iberian Population in Spain	obese	Overweight and obesity	84	2.53	5.54
EGAN00001617778	EGAN00001617778	Genomic DNA extracted from peripheral white blood cells using two kits, the Qiamp DNA Investigator Kit for coagulated samples and Qiamp DNA Mini & Blood Mini Kit for non-coagulated samples (QIAgen Systems, Inc., Valencia, CA, USA).	Human	blood	NA	male	Iberian Population in Spain	obese	Overweight and obesity	89	2.81	2.71
EGAN00001617779	EGAN00001617779	Genomic DNA extracted from peripheral white blood cells using two kits, the Qiamp DNA Investigator Kit for coagulated samples and Qiamp DNA Mini & Blood Mini Kit for non-coagulated samples (QIAgen Systems, Inc., Valencia, CA, USA).	Human	blood	NA	male	Iberian Population in Spain	obese	Overweight and obesity	101	3.99	3.9
EGAN00001617780	EGAN00001617780	Genomic DNA extracted from peripheral white blood cells using two kits, the Qiamp DNA Investigator Kit for coagulated samples and Qiamp DNA Mini & Blood Mini Kit for non-coagulated samples (QIAgen Systems, Inc., Valencia, CA, USA).	Human	blood	NA	male	Iberian Population in Spain	obese	Overweight and obesity	89	1.25	2.28
EGAN00001617781	EGAN00001617781	Genomic DNA extracted from peripheral white blood cells using two kits, the Qiamp DNA Investigator Kit for coagulated samples and Qiamp DNA Mini & Blood Mini Kit for non-coagulated samples (QIAgen Systems, Inc., Valencia, CA, USA).	Human	blood	NA	male	Iberian Population in Spain	obese	Overweight and obesity	NA	NA	3.11
EGAN00001617782	EGAN00001617782	Genomic DNA extracted from peripheral white blood cells using two kits, the Qiamp DNA Investigator Kit for coagulated samples and Qiamp DNA Mini & Blood Mini Kit for non-coagulated samples (QIAgen Systems, Inc., Valencia, CA, USA).	Human	blood	NA	male	Iberian Population in Spain	obese	Overweight and obesity	100	0.91	4.17
EGAN00001617783	EGAN00001617783	Genomic DNA extracted from peripheral white blood cells using two kits, the Qiamp DNA Investigator Kit for coagulated samples and Qiamp DNA Mini & Blood Mini Kit for non-coagulated samples (QIAgen Systems, Inc., Valencia, CA, USA).	Human	blood	NA	male	Iberian Population in Spain	obese	Overweight and obesity	88	0.46	NA
EGAN00001617784	EGAN00001617784	Genomic DNA extracted from peripheral white blood cells using two kits, the Qiamp DNA Investigator Kit for coagulated samples and Qiamp DNA Mini & Blood Mini Kit for non-coagulated samples (QIAgen Systems, Inc., Valencia, CA, USA).	Human	blood	NA	male	Iberian Population in Spain	obese	Overweight and obesity	91	2.15	NA
EGAN00001617785	EGAN00001617785	Genomic DNA extracted from peripheral white blood cells using two kits, the Qiamp DNA Investigator Kit for coagulated samples and Qiamp DNA Mini & Blood Mini Kit for non-coagulated samples (QIAgen Systems, Inc., Valencia, CA, USA).	Human	blood	NA	male	Iberian Population in Spain	obese	Overweight and obesity	88	2.37	2.5
EGAN00001617786	EGAN00001617786	Genomic DNA extracted from peripheral white blood cells using two kits, the Qiamp DNA Investigator Kit for coagulated samples and Qiamp DNA Mini & Blood Mini Kit for non-coagulated samples (QIAgen Systems, Inc., Valencia, CA, USA).	Human	blood	NA	male	Iberian Population in Spain	obese	Overweight and obesity	89	6.63	5.46
EGAN00001617787	EGAN00001617787	Genomic DNA extracted from peripheral white blood cells using two kits, the Qiamp DNA Investigator Kit for coagulated samples and Qiamp DNA Mini & Blood Mini Kit for non-coagulated samples (QIAgen Systems, Inc., Valencia, CA, USA).	Human	blood	NA	male	Iberian Population in Spain	obese	Overweight and obesity	90	1.69	3.33
EGAN00001617788	EGAN00001617788	Genomic DNA extracted from peripheral white blood cells using two kits, the Qiamp DNA Investigator Kit for coagulated samples and Qiamp DNA Mini & Blood Mini Kit for non-coagulated samples (QIAgen Systems, Inc., Valencia, CA, USA).	Human	blood	NA	male	Iberian Population in Spain	obese	Overweight and obesity	89	5.27	3.79
EGAN00001617789	EGAN00001617789	Genomic DNA extracted from peripheral white blood cells using two kits, the Qiamp DNA Investigator Kit for coagulated samples and Qiamp DNA Mini & Blood Mini Kit for non-coagulated samples (QIAgen Systems, Inc., Valencia, CA, USA).	Human	blood	NA	male	Iberian Population in Spain	obese	Overweight and obesity	91	6.91	5.84
EGAN00001617790	EGAN00001617790	Genomic DNA extracted from peripheral white blood cells using two kits, the Qiamp DNA Investigator Kit for coagulated samples and Qiamp DNA Mini & Blood Mini Kit for non-coagulated samples (QIAgen Systems, Inc., Valencia, CA, USA).	Human	blood	NA	male	Iberian Population in Spain	obese	Overweight and obesity	89	6.23	4.03
EGAN00001617791	EGAN00001617791	Genomic DNA extracted from peripheral white blood cells using two kits, the Qiamp DNA Investigator Kit for coagulated samples and Qiamp DNA Mini & Blood Mini Kit for non-coagulated samples (QIAgen Systems, Inc., Valencia, CA, USA).	Human	blood	NA	male	Iberian Population in Spain	obese	Overweight and obesity	91	2.54	6.46
EGAN00001617792	EGAN00001617792	Genomic DNA extracted from peripheral white blood cells using two kits, the Qiamp DNA Investigator Kit for coagulated samples and Qiamp DNA Mini & Blood Mini Kit for non-coagulated samples (QIAgen Systems, Inc., Valencia, CA, USA).	Human	blood	NA	male	Iberian Population in Spain	obese	Overweight and obesity	78	2.56	3.11
EGAN00001617793	EGAN00001617793	Genomic DNA extracted from peripheral white blood cells using two kits, the Qiamp DNA Investigator Kit for coagulated samples and Qiamp DNA Mini & Blood Mini Kit for non-coagulated samples (QIAgen Systems, Inc., Valencia, CA, USA).	Human	blood	NA	male	Iberian Population in Spain	obese	Overweight and obesity	91	0.22	2.2
EGAN00001617794	EGAN00001617794	Genomic DNA extracted from peripheral white blood cells using two kits, the Qiamp DNA Investigator Kit for coagulated samples and Qiamp DNA Mini & Blood Mini Kit for non-coagulated samples (QIAgen Systems, Inc., Valencia, CA, USA).	Human	blood	NA	male	Iberian Population in Spain	obese	Overweight and obesity	97	6.32	2.77
EGAN00001617795	EGAN00001617795	Genomic DNA extracted from peripheral white blood cells using two kits, the Qiamp DNA Investigator Kit for coagulated samples and Qiamp DNA Mini & Blood Mini Kit for non-coagulated samples (QIAgen Systems, Inc., Valencia, CA, USA).	Human	blood	NA	male	Iberian Population in Spain	obese	Overweight and obesity	102	1.23	3.37
EGAN00001617796	EGAN00001617796	Genomic DNA extracted from peripheral white blood cells using two kits, the Qiamp DNA Investigator Kit for coagulated samples and Qiamp DNA Mini & Blood Mini Kit for non-coagulated samples (QIAgen Systems, Inc., Valencia, CA, USA).	Human	blood	NA	male	Iberian Population in Spain	obese	Overweight and obesity	82	NA	2.29
EGAN00001617797	EGAN00001617797	Genomic DNA extracted from peripheral white blood cells using two kits, the Qiamp DNA Investigator Kit for coagulated samples and Qiamp DNA Mini & Blood Mini Kit for non-coagulated samples (QIAgen Systems, Inc., Valencia, CA, USA).	Human	blood	NA	male	Iberian Population in Spain	obese	Overweight and obesity	79	1.13	2.82
EGAN00001617798	EGAN00001617798	Genomic DNA extracted from peripheral white blood cells using two kits, the Qiamp DNA Investigator Kit for coagulated samples and Qiamp DNA Mini & Blood Mini Kit for non-coagulated samples (QIAgen Systems, Inc., Valencia, CA, USA).	Human	blood	NA	male	Iberian Population in Spain	obese	Overweight and obesity	89	2.04	2.11
EGAN00001617799	EGAN00001617799	Genomic DNA extracted from peripheral white blood cells using two kits, the Qiamp DNA Investigator Kit for coagulated samples and Qiamp DNA Mini & Blood Mini Kit for non-coagulated samples (QIAgen Systems, Inc., Valencia, CA, USA).	Human	blood	NA	male	Iberian Population in Spain	obese	Overweight and obesity	83	2.83	4.04
EGAN00001617800	EGAN00001617800	Genomic DNA extracted from peripheral white blood cells using two kits, the Qiamp DNA Investigator Kit for coagulated samples and Qiamp DNA Mini & Blood Mini Kit for non-coagulated samples (QIAgen Systems, Inc., Valencia, CA, USA).	Human	blood	NA	male	Iberian Population in Spain	obese	Overweight and obesity	81	1.96	4.46
EGAN00001617801	EGAN00001617801	Genomic DNA extracted from peripheral white blood cells using two kits, the Qiamp DNA Investigator Kit for coagulated samples and Qiamp DNA Mini & Blood Mini Kit for non-coagulated samples (QIAgen Systems, Inc., Valencia, CA, USA).	Human	blood	NA	male	Iberian Population in Spain	obese	Overweight and obesity	83	0.53	2.24
EGAN00001617802	EGAN00001617802	Genomic DNA extracted from peripheral white blood cells using two kits, the Qiamp DNA Investigator Kit for coagulated samples and Qiamp DNA Mini & Blood Mini Kit for non-coagulated samples (QIAgen Systems, Inc., Valencia, CA, USA).	Human	blood	NA	male	Iberian Population in Spain	obese	Overweight and obesity	83	2.91	2.46
EGAN00001617803	EGAN00001617803	Genomic DNA extracted from peripheral white blood cells using two kits, the Qiamp DNA Investigator Kit for coagulated samples and Qiamp DNA Mini & Blood Mini Kit for non-coagulated samples (QIAgen Systems, Inc., Valencia, CA, USA).	Human	blood	NA	male	Iberian Population in Spain	obese	Overweight and obesity	75	1.44	4.15
EGAN00001617804	EGAN00001617804	Genomic DNA extracted from peripheral white blood cells using two kits, the Qiamp DNA Investigator Kit for coagulated samples and Qiamp DNA Mini & Blood Mini Kit for non-coagulated samples (QIAgen Systems, Inc., Valencia, CA, USA).	Human	blood	NA	male	Iberian Population in Spain	obese	Overweight and obesity	83	2.68	3.97
EGAN00001617805	EGAN00001617805	Genomic DNA extracted from peripheral white blood cells using two kits, the Qiamp DNA Investigator Kit for coagulated samples and Qiamp DNA Mini & Blood Mini Kit for non-coagulated samples (QIAgen Systems, Inc., Valencia, CA, USA).	Human	blood	NA	male	Iberian Population in Spain	obese	Overweight and obesity	82	3.09	2.7
EGAN00001617806	EGAN00001617806	Genomic DNA extracted from peripheral white blood cells using two kits, the Qiamp DNA Investigator Kit for coagulated samples and Qiamp DNA Mini & Blood Mini Kit for non-coagulated samples (QIAgen Systems, Inc., Valencia, CA, USA).	Human	blood	NA	male	Iberian Population in Spain	obese	Overweight and obesity	81	6.19	4.01
EGAN00001617807	EGAN00001617807	Genomic DNA extracted from peripheral white blood cells using two kits, the Qiamp DNA Investigator Kit for coagulated samples and Qiamp DNA Mini & Blood Mini Kit for non-coagulated samples (QIAgen Systems, Inc., Valencia, CA, USA).	Human	blood	NA	male	Iberian Population in Spain	obese	Overweight and obesity	87	4.31	3.79
EGAN00001617808	EGAN00001617808	Genomic DNA extracted from peripheral white blood cells using two kits, the Qiamp DNA Investigator Kit for coagulated samples and Qiamp DNA Mini & Blood Mini Kit for non-coagulated samples (QIAgen Systems, Inc., Valencia, CA, USA).	Human	blood	NA	male	Iberian Population in Spain	obese	Overweight and obesity	95	7.11	5.94
EGAN00001617809	EGAN00001617809	Genomic DNA extracted from peripheral white blood cells using two kits, the Qiamp DNA Investigator Kit for coagulated samples and Qiamp DNA Mini & Blood Mini Kit for non-coagulated samples (QIAgen Systems, Inc., Valencia, CA, USA).	Human	blood	NA	male	Iberian Population in Spain	obese	Overweight and obesity	84	4.99	4.52
EGAN00001617810	EGAN00001617810	Genomic DNA extracted from peripheral white blood cells using two kits, the Qiamp DNA Investigator Kit for coagulated samples and Qiamp DNA Mini & Blood Mini Kit for non-coagulated samples (QIAgen Systems, Inc., Valencia, CA, USA).	Human	blood	NA	male	Iberian Population in Spain	obese	Overweight and obesity	94	3.17	3.44
EGAN00001617811	EGAN00001617811	Genomic DNA extracted from peripheral white blood cells using two kits, the Qiamp DNA Investigator Kit for coagulated samples and Qiamp DNA Mini & Blood Mini Kit for non-coagulated samples (QIAgen Systems, Inc., Valencia, CA, USA).	Human	blood	NA	male	Iberian Population in Spain	obese	Overweight and obesity	91	5.79	2.93
EGAN00001617812	EGAN00001617812	Genomic DNA extracted from peripheral white blood cells using two kits, the Qiamp DNA Investigator Kit for coagulated samples and Qiamp DNA Mini & Blood Mini Kit for non-coagulated samples (QIAgen Systems, Inc., Valencia, CA, USA).	Human	blood	NA	male	Iberian Population in Spain	obese	Overweight and obesity	88	2.04	2.88
EGAN00001617813	EGAN00001617813	Genomic DNA extracted from peripheral white blood cells using two kits, the Qiamp DNA Investigator Kit for coagulated samples and Qiamp DNA Mini & Blood Mini Kit for non-coagulated samples (QIAgen Systems, Inc., Valencia, CA, USA).	Human	blood	NA	male	Iberian Population in Spain	obese	Overweight and obesity	92	5.31	2.4
EGAN00001617814	EGAN00001617814	Genomic DNA extracted from peripheral white blood cells using two kits, the Qiamp DNA Investigator Kit for coagulated samples and Qiamp DNA Mini & Blood Mini Kit for non-coagulated samples (QIAgen Systems, Inc., Valencia, CA, USA).	Human	blood	NA	male	Iberian Population in Spain	obese	Overweight and obesity	89	2.68	2.44
EGAN00001617815	EGAN00001617815	Genomic DNA extracted from peripheral white blood cells using two kits, the Qiamp DNA Investigator Kit for coagulated samples and Qiamp DNA Mini & Blood Mini Kit for non-coagulated samples (QIAgen Systems, Inc., Valencia, CA, USA).	Human	blood	NA	male	Iberian Population in Spain	obese	Overweight and obesity	85	2.63	2.7
EGAN00001617816	EGAN00001617816	Genomic DNA extracted from peripheral white blood cells using two kits, the Qiamp DNA Investigator Kit for coagulated samples and Qiamp DNA Mini & Blood Mini Kit for non-coagulated samples (QIAgen Systems, Inc., Valencia, CA, USA).	Human	blood	NA	male	Iberian Population in Spain	obese	Overweight and obesity	93	2.3	2.34
EGAN00001617817	EGAN00001617817	Genomic DNA extracted from peripheral white blood cells using two kits, the Qiamp DNA Investigator Kit for coagulated samples and Qiamp DNA Mini & Blood Mini Kit for non-coagulated samples (QIAgen Systems, Inc., Valencia, CA, USA).	Human	blood	NA	male	Iberian Population in Spain	obese	Overweight and obesity	81	1.92	3.06
EGAN00001617818	EGAN00001617818	Genomic DNA extracted from peripheral white blood cells using two kits, the Qiamp DNA Investigator Kit for coagulated samples and Qiamp DNA Mini & Blood Mini Kit for non-coagulated samples (QIAgen Systems, Inc., Valencia, CA, USA).	Human	blood	NA	male	Iberian Population in Spain	obese	Overweight and obesity	83	4.24	2.39
EGAN00001617819	EGAN00001617819	Genomic DNA extracted from peripheral white blood cells using two kits, the Qiamp DNA Investigator Kit for coagulated samples and Qiamp DNA Mini & Blood Mini Kit for non-coagulated samples (QIAgen Systems, Inc., Valencia, CA, USA).	Human	blood	NA	male	Iberian Population in Spain	obese	Overweight and obesity	97	10.69	2.55
EGAN00001617820	EGAN00001617820	Genomic DNA extracted from peripheral white blood cells using two kits, the Qiamp DNA Investigator Kit for coagulated samples and Qiamp DNA Mini & Blood Mini Kit for non-coagulated samples (QIAgen Systems, Inc., Valencia, CA, USA).	Human	blood	NA	male	Iberian Population in Spain	obese	Overweight and obesity	92	6.89	4.1
EGAN00001617821	EGAN00001617821	Genomic DNA extracted from peripheral white blood cells using two kits, the Qiamp DNA Investigator Kit for coagulated samples and Qiamp DNA Mini & Blood Mini Kit for non-coagulated samples (QIAgen Systems, Inc., Valencia, CA, USA).	Human	blood	NA	female	Iberian Population in Spain	normal weight	Overweight and obesity	96	5.93	0.87
EGAN00001617822	EGAN00001617822	Genomic DNA extracted from peripheral white blood cells using two kits, the Qiamp DNA Investigator Kit for coagulated samples and Qiamp DNA Mini & Blood Mini Kit for non-coagulated samples (QIAgen Systems, Inc., Valencia, CA, USA).	Human	blood	NA	female	Iberian Population in Spain	normal weight	Overweight and obesity	105	4.46	−0.06
EGAN00001617823	EGAN00001617823	Genomic DNA extracted from peripheral white blood cells using two kits, the Qiamp DNA Investigator Kit for coagulated samples and Qiamp DNA Mini & Blood Mini Kit for non-coagulated samples (QIAgen Systems, Inc., Valencia, CA, USA).	Human	blood	NA	female	Iberian Population in Spain	normal weight	Overweight and obesity	89	1.63	−0.26
EGAN00001617824	EGAN00001617824	Genomic DNA extracted from peripheral white blood cells using two kits, the Qiamp DNA Investigator Kit for coagulated samples and Qiamp DNA Mini & Blood Mini Kit for non-coagulated samples (QIAgen Systems, Inc., Valencia, CA, USA).	Human	blood	NA	female	Iberian Population in Spain	normal weight	Overweight and obesity	96	0.47	0.49
EGAN00001617825	EGAN00001617825	Genomic DNA extracted from peripheral white blood cells using two kits, the Qiamp DNA Investigator Kit for coagulated samples and Qiamp DNA Mini & Blood Mini Kit for non-coagulated samples (QIAgen Systems, Inc., Valencia, CA, USA).	Human	blood	NA	female	Iberian Population in Spain	normal weight	Overweight and obesity	86	0.62	−0.96
EGAN00001617826	EGAN00001617826	Genomic DNA extracted from peripheral white blood cells using two kits, the Qiamp DNA Investigator Kit for coagulated samples and Qiamp DNA Mini & Blood Mini Kit for non-coagulated samples (QIAgen Systems, Inc., Valencia, CA, USA).	Human	blood	NA	female	Iberian Population in Spain	normal weight	Overweight and obesity	81	0.4	0.2
EGAN00001617827	EGAN00001617827	Genomic DNA extracted from peripheral white blood cells using two kits, the Qiamp DNA Investigator Kit for coagulated samples and Qiamp DNA Mini & Blood Mini Kit for non-coagulated samples (QIAgen Systems, Inc., Valencia, CA, USA).	Human	blood	NA	female	Iberian Population in Spain	normal weight	Overweight and obesity	87	1.5	−0.77
EGAN00001617828	EGAN00001617828	Genomic DNA extracted from peripheral white blood cells using two kits, the Qiamp DNA Investigator Kit for coagulated samples and Qiamp DNA Mini & Blood Mini Kit for non-coagulated samples (QIAgen Systems, Inc., Valencia, CA, USA).	Human	blood	NA	female	Iberian Population in Spain	normal weight	Overweight and obesity	80	1.23	−0.95
EGAN00001617829	EGAN00001617829	Genomic DNA extracted from peripheral white blood cells using two kits, the Qiamp DNA Investigator Kit for coagulated samples and Qiamp DNA Mini & Blood Mini Kit for non-coagulated samples (QIAgen Systems, Inc., Valencia, CA, USA).	Human	blood	NA	female	Iberian Population in Spain	normal weight	Overweight and obesity	81	NA	−1.08
EGAN00001617830	EGAN00001617830	Genomic DNA extracted from peripheral white blood cells using two kits, the Qiamp DNA Investigator Kit for coagulated samples and Qiamp DNA Mini & Blood Mini Kit for non-coagulated samples (QIAgen Systems, Inc., Valencia, CA, USA).	Human	blood	NA	female	Iberian Population in Spain	normal weight	Overweight and obesity	65	0.39	−0.5
EGAN00001617831	EGAN00001617831	Genomic DNA extracted from peripheral white blood cells using two kits, the Qiamp DNA Investigator Kit for coagulated samples and Qiamp DNA Mini & Blood Mini Kit for non-coagulated samples (QIAgen Systems, Inc., Valencia, CA, USA).	Human	blood	NA	female	Iberian Population in Spain	normal weight	Overweight and obesity	77	1.03	−0.14
EGAN00001617832	EGAN00001617832	Genomic DNA extracted from peripheral white blood cells using two kits, the Qiamp DNA Investigator Kit for coagulated samples and Qiamp DNA Mini & Blood Mini Kit for non-coagulated samples (QIAgen Systems, Inc., Valencia, CA, USA).	Human	blood	NA	female	Iberian Population in Spain	normal weight	Overweight and obesity	89	1.89	−0.42
EGAN00001617833	EGAN00001617833	Genomic DNA extracted from peripheral white blood cells using two kits, the Qiamp DNA Investigator Kit for coagulated samples and Qiamp DNA Mini & Blood Mini Kit for non-coagulated samples (QIAgen Systems, Inc., Valencia, CA, USA).	Human	blood	NA	female	Iberian Population in Spain	normal weight	Overweight and obesity	91	1.03	−0.92
EGAN00001617834	EGAN00001617834	Genomic DNA extracted from peripheral white blood cells using two kits, the Qiamp DNA Investigator Kit for coagulated samples and Qiamp DNA Mini & Blood Mini Kit for non-coagulated samples (QIAgen Systems, Inc., Valencia, CA, USA).	Human	blood	NA	female	Iberian Population in Spain	normal weight	Overweight and obesity	90	1.38	−0.49
EGAN00001617835	EGAN00001617835	Genomic DNA extracted from peripheral white blood cells using two kits, the Qiamp DNA Investigator Kit for coagulated samples and Qiamp DNA Mini & Blood Mini Kit for non-coagulated samples (QIAgen Systems, Inc., Valencia, CA, USA).	Human	blood	NA	female	Iberian Population in Spain	normal weight	Overweight and obesity	80	NA	0.42
EGAN00001617836	EGAN00001617836	Genomic DNA extracted from peripheral white blood cells using two kits, the Qiamp DNA Investigator Kit for coagulated samples and Qiamp DNA Mini & Blood Mini Kit for non-coagulated samples (QIAgen Systems, Inc., Valencia, CA, USA).	Human	blood	NA	female	Iberian Population in Spain	normal weight	Overweight and obesity	84	0.79	−0.1
EGAN00001617837	EGAN00001617837	Genomic DNA extracted from peripheral white blood cells using two kits, the Qiamp DNA Investigator Kit for coagulated samples and Qiamp DNA Mini & Blood Mini Kit for non-coagulated samples (QIAgen Systems, Inc., Valencia, CA, USA).	Human	blood	NA	female	Iberian Population in Spain	normal weight	Overweight and obesity	76	0.49	−0.22
EGAN00001617838	EGAN00001617838	Genomic DNA extracted from peripheral white blood cells using two kits, the Qiamp DNA Investigator Kit for coagulated samples and Qiamp DNA Mini & Blood Mini Kit for non-coagulated samples (QIAgen Systems, Inc., Valencia, CA, USA).	Human	blood	NA	female	Iberian Population in Spain	normal weight	Overweight and obesity	73	0.43	−0.49
EGAN00001617839	EGAN00001617839	Genomic DNA extracted from peripheral white blood cells using two kits, the Qiamp DNA Investigator Kit for coagulated samples and Qiamp DNA Mini & Blood Mini Kit for non-coagulated samples (QIAgen Systems, Inc., Valencia, CA, USA).	Human	blood	NA	female	Iberian Population in Spain	normal weight	Overweight and obesity	91	2.02	0.39
EGAN00001617840	EGAN00001617840	Genomic DNA extracted from peripheral white blood cells using two kits, the Qiamp DNA Investigator Kit for coagulated samples and Qiamp DNA Mini & Blood Mini Kit for non-coagulated samples (QIAgen Systems, Inc., Valencia, CA, USA).	Human	blood	NA	female	Iberian Population in Spain	normal weight	Overweight and obesity	78	0.56	−0.78
EGAN00001617841	EGAN00001617841	Genomic DNA extracted from peripheral white blood cells using two kits, the Qiamp DNA Investigator Kit for coagulated samples and Qiamp DNA Mini & Blood Mini Kit for non-coagulated samples (QIAgen Systems, Inc., Valencia, CA, USA).	Human	blood	NA	female	Iberian Population in Spain	normal weight	Overweight and obesity	97	5.03	0.33
EGAN00001617842	EGAN00001617842	Genomic DNA extracted from peripheral white blood cells using two kits, the Qiamp DNA Investigator Kit for coagulated samples and Qiamp DNA Mini & Blood Mini Kit for non-coagulated samples (QIAgen Systems, Inc., Valencia, CA, USA).	Human	blood	NA	female	Iberian Population in Spain	normal weight	Overweight and obesity	77	1.05	−0.17
EGAN00001617843	EGAN00001617843	Genomic DNA extracted from peripheral white blood cells using two kits, the Qiamp DNA Investigator Kit for coagulated samples and Qiamp DNA Mini & Blood Mini Kit for non-coagulated samples (QIAgen Systems, Inc., Valencia, CA, USA).	Human	blood	NA	female	Iberian Population in Spain	normal weight	Overweight and obesity	89	1.89	−0.36
EGAN00001617844	EGAN00001617844	Genomic DNA extracted from peripheral white blood cells using two kits, the Qiamp DNA Investigator Kit for coagulated samples and Qiamp DNA Mini & Blood Mini Kit for non-coagulated samples (QIAgen Systems, Inc., Valencia, CA, USA).	Human	blood	NA	female	Iberian Population in Spain	normal weight	Overweight and obesity	83	1.64	0.26
EGAN00001617845	EGAN00001617845	Genomic DNA extracted from peripheral white blood cells using two kits, the Qiamp DNA Investigator Kit for coagulated samples and Qiamp DNA Mini & Blood Mini Kit for non-coagulated samples (QIAgen Systems, Inc., Valencia, CA, USA).	Human	blood	NA	female	Iberian Population in Spain	normal weight	Overweight and obesity	82	1.05	−0.84
EGAN00001617846	EGAN00001617846	Genomic DNA extracted from peripheral white blood cells using two kits, the Qiamp DNA Investigator Kit for coagulated samples and Qiamp DNA Mini & Blood Mini Kit for non-coagulated samples (QIAgen Systems, Inc., Valencia, CA, USA).	Human	blood	NA	female	Iberian Population in Spain	normal weight	Overweight and obesity	82	1.98	−0.25
EGAN00001617847	EGAN00001617847	Genomic DNA extracted from peripheral white blood cells using two kits, the Qiamp DNA Investigator Kit for coagulated samples and Qiamp DNA Mini & Blood Mini Kit for non-coagulated samples (QIAgen Systems, Inc., Valencia, CA, USA).	Human	blood	NA	female	Iberian Population in Spain	normal weight	Overweight and obesity	84	1.62	−1.26
EGAN00001617848	EGAN00001617848	Genomic DNA extracted from peripheral white blood cells using two kits, the Qiamp DNA Investigator Kit for coagulated samples and Qiamp DNA Mini & Blood Mini Kit for non-coagulated samples (QIAgen Systems, Inc., Valencia, CA, USA).	Human	blood	NA	female	Iberian Population in Spain	normal weight	Overweight and obesity	92	1.76	0.38
EGAN00001617849	EGAN00001617849	Genomic DNA extracted from peripheral white blood cells using two kits, the Qiamp DNA Investigator Kit for coagulated samples and Qiamp DNA Mini & Blood Mini Kit for non-coagulated samples (QIAgen Systems, Inc., Valencia, CA, USA).	Human	blood	NA	female	Iberian Population in Spain	normal weight	Overweight and obesity	90	1.31	−0.33
EGAN00001617850	EGAN00001617850	Genomic DNA extracted from peripheral white blood cells using two kits, the Qiamp DNA Investigator Kit for coagulated samples and Qiamp DNA Mini & Blood Mini Kit for non-coagulated samples (QIAgen Systems, Inc., Valencia, CA, USA).	Human	blood	NA	female	Iberian Population in Spain	normal weight	Overweight and obesity	82	0.73	0.33
EGAN00001617851	EGAN00001617851	Genomic DNA extracted from peripheral white blood cells using two kits, the Qiamp DNA Investigator Kit for coagulated samples and Qiamp DNA Mini & Blood Mini Kit for non-coagulated samples (QIAgen Systems, Inc., Valencia, CA, USA).	Human	blood	NA	female	Iberian Population in Spain	normal weight	Overweight and obesity	81	1.11	−0.49
EGAN00001617852	EGAN00001617852	Genomic DNA extracted from peripheral white blood cells using two kits, the Qiamp DNA Investigator Kit for coagulated samples and Qiamp DNA Mini & Blood Mini Kit for non-coagulated samples (QIAgen Systems, Inc., Valencia, CA, USA).	Human	blood	NA	female	Iberian Population in Spain	normal weight	Overweight and obesity	85	1.15	0.41
EGAN00001617853	EGAN00001617853	Genomic DNA extracted from peripheral white blood cells using two kits, the Qiamp DNA Investigator Kit for coagulated samples and Qiamp DNA Mini & Blood Mini Kit for non-coagulated samples (QIAgen Systems, Inc., Valencia, CA, USA).	Human	blood	NA	female	Iberian Population in Spain	normal weight	Overweight and obesity	93	1	−0.58
EGAN00001617854	EGAN00001617854	Genomic DNA extracted from peripheral white blood cells using two kits, the Qiamp DNA Investigator Kit for coagulated samples and Qiamp DNA Mini & Blood Mini Kit for non-coagulated samples (QIAgen Systems, Inc., Valencia, CA, USA).	Human	blood	NA	female	Iberian Population in Spain	normal weight	Overweight and obesity	77	0.59	−0.17
EGAN00001617855	EGAN00001617855	Genomic DNA extracted from peripheral white blood cells using two kits, the Qiamp DNA Investigator Kit for coagulated samples and Qiamp DNA Mini & Blood Mini Kit for non-coagulated samples (QIAgen Systems, Inc., Valencia, CA, USA).	Human	blood	NA	female	Iberian Population in Spain	normal weight	Overweight and obesity	83	0.62	0.23
EGAN00001617856	EGAN00001617856	Genomic DNA extracted from peripheral white blood cells using two kits, the Qiamp DNA Investigator Kit for coagulated samples and Qiamp DNA Mini & Blood Mini Kit for non-coagulated samples (QIAgen Systems, Inc., Valencia, CA, USA).	Human	blood	NA	female	Iberian Population in Spain	normal weight	Overweight and obesity	77	1.05	0.05
EGAN00001617857	EGAN00001617857	Genomic DNA extracted from peripheral white blood cells using two kits, the Qiamp DNA Investigator Kit for coagulated samples and Qiamp DNA Mini & Blood Mini Kit for non-coagulated samples (QIAgen Systems, Inc., Valencia, CA, USA).	Human	blood	NA	female	Iberian Population in Spain	normal weight	Overweight and obesity	87	1.49	−0.13
EGAN00001617858	EGAN00001617858	Genomic DNA extracted from peripheral white blood cells using two kits, the Qiamp DNA Investigator Kit for coagulated samples and Qiamp DNA Mini & Blood Mini Kit for non-coagulated samples (QIAgen Systems, Inc., Valencia, CA, USA).	Human	blood	NA	female	Iberian Population in Spain	normal weight	Overweight and obesity	71	0.35	0.02
EGAN00001617859	EGAN00001617859	Genomic DNA extracted from peripheral white blood cells using two kits, the Qiamp DNA Investigator Kit for coagulated samples and Qiamp DNA Mini & Blood Mini Kit for non-coagulated samples (QIAgen Systems, Inc., Valencia, CA, USA).	Human	blood	NA	female	Iberian Population in Spain	normal weight	Overweight and obesity	76	0.51	0.71
EGAN00001617860	EGAN00001617860	Genomic DNA extracted from peripheral white blood cells using two kits, the Qiamp DNA Investigator Kit for coagulated samples and Qiamp DNA Mini & Blood Mini Kit for non-coagulated samples (QIAgen Systems, Inc., Valencia, CA, USA).	Human	blood	NA	female	Iberian Population in Spain	normal weight	Overweight and obesity	78	2.12	−0.02
EGAN00001617861	EGAN00001617861	Genomic DNA extracted from peripheral white blood cells using two kits, the Qiamp DNA Investigator Kit for coagulated samples and Qiamp DNA Mini & Blood Mini Kit for non-coagulated samples (QIAgen Systems, Inc., Valencia, CA, USA).	Human	blood	NA	female	Iberian Population in Spain	normal weight	Overweight and obesity	85	3.19	0.32
EGAN00001617862	EGAN00001617862	Genomic DNA extracted from peripheral white blood cells using two kits, the Qiamp DNA Investigator Kit for coagulated samples and Qiamp DNA Mini & Blood Mini Kit for non-coagulated samples (QIAgen Systems, Inc., Valencia, CA, USA).	Human	blood	NA	female	Iberian Population in Spain	normal weight	Overweight and obesity	82	1.9	0.37
EGAN00001617863	EGAN00001617863	Genomic DNA extracted from peripheral white blood cells using two kits, the Qiamp DNA Investigator Kit for coagulated samples and Qiamp DNA Mini & Blood Mini Kit for non-coagulated samples (QIAgen Systems, Inc., Valencia, CA, USA).	Human	blood	NA	female	Iberian Population in Spain	normal weight	Overweight and obesity	90	1.08	−0.8
EGAN00001617864	EGAN00001617864	Genomic DNA extracted from peripheral white blood cells using two kits, the Qiamp DNA Investigator Kit for coagulated samples and Qiamp DNA Mini & Blood Mini Kit for non-coagulated samples (QIAgen Systems, Inc., Valencia, CA, USA).	Human	blood	NA	female	Iberian Population in Spain	normal weight	Overweight and obesity	87	0.9	0.05
EGAN00001617865	EGAN00001617865	Genomic DNA extracted from peripheral white blood cells using two kits, the Qiamp DNA Investigator Kit for coagulated samples and Qiamp DNA Mini & Blood Mini Kit for non-coagulated samples (QIAgen Systems, Inc., Valencia, CA, USA).	Human	blood	NA	female	Iberian Population in Spain	normal weight	Overweight and obesity	101	1	0.04
EGAN00001617866	EGAN00001617866	Genomic DNA extracted from peripheral white blood cells using two kits, the Qiamp DNA Investigator Kit for coagulated samples and Qiamp DNA Mini & Blood Mini Kit for non-coagulated samples (QIAgen Systems, Inc., Valencia, CA, USA).	Human	blood	NA	female	Iberian Population in Spain	normal weight	Overweight and obesity	84	0.77	−0.14
EGAN00001617867	EGAN00001617867	Genomic DNA extracted from peripheral white blood cells using two kits, the Qiamp DNA Investigator Kit for coagulated samples and Qiamp DNA Mini & Blood Mini Kit for non-coagulated samples (QIAgen Systems, Inc., Valencia, CA, USA).	Human	blood	NA	female	Iberian Population in Spain	normal weight	Overweight and obesity	83	0.79	−0.38
EGAN00001617868	EGAN00001617868	Genomic DNA extracted from peripheral white blood cells using two kits, the Qiamp DNA Investigator Kit for coagulated samples and Qiamp DNA Mini & Blood Mini Kit for non-coagulated samples (QIAgen Systems, Inc., Valencia, CA, USA).	Human	blood	NA	female	Iberian Population in Spain	normal weight	Overweight and obesity	77	0.88	−0.4
EGAN00001617869	EGAN00001617869	Genomic DNA extracted from peripheral white blood cells using two kits, the Qiamp DNA Investigator Kit for coagulated samples and Qiamp DNA Mini & Blood Mini Kit for non-coagulated samples (QIAgen Systems, Inc., Valencia, CA, USA).	Human	blood	NA	female	Iberian Population in Spain	normal weight	Overweight and obesity	77	0.65	−0.61
EGAN00001617870	EGAN00001617870	Genomic DNA extracted from peripheral white blood cells using two kits, the Qiamp DNA Investigator Kit for coagulated samples and Qiamp DNA Mini & Blood Mini Kit for non-coagulated samples (QIAgen Systems, Inc., Valencia, CA, USA).	Human	blood	NA	female	Iberian Population in Spain	normal weight	Overweight and obesity	79	NA	−0.87
EGAN00001617871	EGAN00001617871	Genomic DNA extracted from peripheral white blood cells using two kits, the Qiamp DNA Investigator Kit for coagulated samples and Qiamp DNA Mini & Blood Mini Kit for non-coagulated samples (QIAgen Systems, Inc., Valencia, CA, USA).	Human	blood	NA	female	Iberian Population in Spain	normal weight	Overweight and obesity	77	0.7	−0.97
EGAN00001617872	EGAN00001617872	Genomic DNA extracted from peripheral white blood cells using two kits, the Qiamp DNA Investigator Kit for coagulated samples and Qiamp DNA Mini & Blood Mini Kit for non-coagulated samples (QIAgen Systems, Inc., Valencia, CA, USA).	Human	blood	NA	female	Iberian Population in Spain	normal weight	Overweight and obesity	80	0.97	0.28
EGAN00001617873	EGAN00001617873	Genomic DNA extracted from peripheral white blood cells using two kits, the Qiamp DNA Investigator Kit for coagulated samples and Qiamp DNA Mini & Blood Mini Kit for non-coagulated samples (QIAgen Systems, Inc., Valencia, CA, USA).	Human	blood	NA	female	Iberian Population in Spain	normal weight	Overweight and obesity	76	1.06	−0.35
EGAN00001617874	EGAN00001617874	Genomic DNA extracted from peripheral white blood cells using two kits, the Qiamp DNA Investigator Kit for coagulated samples and Qiamp DNA Mini & Blood Mini Kit for non-coagulated samples (QIAgen Systems, Inc., Valencia, CA, USA).	Human	blood	NA	female	Iberian Population in Spain	normal weight	Overweight and obesity	104	2.19	−0.42
EGAN00001617875	EGAN00001617875	Genomic DNA extracted from peripheral white blood cells using two kits, the Qiamp DNA Investigator Kit for coagulated samples and Qiamp DNA Mini & Blood Mini Kit for non-coagulated samples (QIAgen Systems, Inc., Valencia, CA, USA).	Human	blood	NA	female	Iberian Population in Spain	normal weight	Overweight and obesity	95	1.09	0.32
EGAN00001617876	EGAN00001617876	Genomic DNA extracted from peripheral white blood cells using two kits, the Qiamp DNA Investigator Kit for coagulated samples and Qiamp DNA Mini & Blood Mini Kit for non-coagulated samples (QIAgen Systems, Inc., Valencia, CA, USA).	Human	blood	NA	female	Iberian Population in Spain	normal weight	Overweight and obesity	83	1.44	−0.23
EGAN00001617877	EGAN00001617877	Genomic DNA extracted from peripheral white blood cells using two kits, the Qiamp DNA Investigator Kit for coagulated samples and Qiamp DNA Mini & Blood Mini Kit for non-coagulated samples (QIAgen Systems, Inc., Valencia, CA, USA).	Human	blood	NA	female	Iberian Population in Spain	normal weight	Overweight and obesity	78	1.54	0.4
EGAN00001617878	EGAN00001617878	Genomic DNA extracted from peripheral white blood cells using two kits, the Qiamp DNA Investigator Kit for coagulated samples and Qiamp DNA Mini & Blood Mini Kit for non-coagulated samples (QIAgen Systems, Inc., Valencia, CA, USA).	Human	blood	NA	female	Iberian Population in Spain	normal weight	Overweight and obesity	79	0.37	−1.21
EGAN00001617879	EGAN00001617879	Genomic DNA extracted from peripheral white blood cells using two kits, the Qiamp DNA Investigator Kit for coagulated samples and Qiamp DNA Mini & Blood Mini Kit for non-coagulated samples (QIAgen Systems, Inc., Valencia, CA, USA).	Human	blood	NA	female	Iberian Population in Spain	normal weight	Overweight and obesity	75	1.43	−0.48
EGAN00001617880	EGAN00001617880	Genomic DNA extracted from peripheral white blood cells using two kits, the Qiamp DNA Investigator Kit for coagulated samples and Qiamp DNA Mini & Blood Mini Kit for non-coagulated samples (QIAgen Systems, Inc., Valencia, CA, USA).	Human	blood	NA	female	Iberian Population in Spain	normal weight	Overweight and obesity	79	0.76	0.02
EGAN00001617881	EGAN00001617881	Genomic DNA extracted from peripheral white blood cells using two kits, the Qiamp DNA Investigator Kit for coagulated samples and Qiamp DNA Mini & Blood Mini Kit for non-coagulated samples (QIAgen Systems, Inc., Valencia, CA, USA).	Human	blood	NA	female	Iberian Population in Spain	normal weight	Overweight and obesity	82	2.77	−0.58
EGAN00001617882	EGAN00001617882	Genomic DNA extracted from peripheral white blood cells using two kits, the Qiamp DNA Investigator Kit for coagulated samples and Qiamp DNA Mini & Blood Mini Kit for non-coagulated samples (QIAgen Systems, Inc., Valencia, CA, USA).	Human	blood	NA	female	Iberian Population in Spain	normal weight	Overweight and obesity	83	3.03	−0.43
EGAN00001617883	EGAN00001617883	Genomic DNA extracted from peripheral white blood cells using two kits, the Qiamp DNA Investigator Kit for coagulated samples and Qiamp DNA Mini & Blood Mini Kit for non-coagulated samples (QIAgen Systems, Inc., Valencia, CA, USA).	Human	blood	NA	female	Iberian Population in Spain	normal weight	Overweight and obesity	77	1.52	−0.71
EGAN00001617884	EGAN00001617884	Genomic DNA extracted from peripheral white blood cells using two kits, the Qiamp DNA Investigator Kit for coagulated samples and Qiamp DNA Mini & Blood Mini Kit for non-coagulated samples (QIAgen Systems, Inc., Valencia, CA, USA).	Human	blood	NA	female	Iberian Population in Spain	normal weight	Overweight and obesity	86	1.8	−0.23
EGAN00001617885	EGAN00001617885	Genomic DNA extracted from peripheral white blood cells using two kits, the Qiamp DNA Investigator Kit for coagulated samples and Qiamp DNA Mini & Blood Mini Kit for non-coagulated samples (QIAgen Systems, Inc., Valencia, CA, USA).	Human	blood	NA	female	Iberian Population in Spain	normal weight	Overweight and obesity	63	1.57	−0.34
EGAN00001617886	EGAN00001617886	Genomic DNA extracted from peripheral white blood cells using two kits, the Qiamp DNA Investigator Kit for coagulated samples and Qiamp DNA Mini & Blood Mini Kit for non-coagulated samples (QIAgen Systems, Inc., Valencia, CA, USA).	Human	blood	NA	female	Iberian Population in Spain	normal weight	Overweight and obesity	71	2.31	−0.12
EGAN00001617887	EGAN00001617887	Genomic DNA extracted from peripheral white blood cells using two kits, the Qiamp DNA Investigator Kit for coagulated samples and Qiamp DNA Mini & Blood Mini Kit for non-coagulated samples (QIAgen Systems, Inc., Valencia, CA, USA).	Human	blood	NA	female	Iberian Population in Spain	normal weight	Overweight and obesity	82	1.54	−0.61
EGAN00001617888	EGAN00001617888	Genomic DNA extracted from peripheral white blood cells using two kits, the Qiamp DNA Investigator Kit for coagulated samples and Qiamp DNA Mini & Blood Mini Kit for non-coagulated samples (QIAgen Systems, Inc., Valencia, CA, USA).	Human	blood	NA	female	Iberian Population in Spain	normal weight	Overweight and obesity	77	2.17	−0.47
EGAN00001617889	EGAN00001617889	Genomic DNA extracted from peripheral white blood cells using two kits, the Qiamp DNA Investigator Kit for coagulated samples and Qiamp DNA Mini & Blood Mini Kit for non-coagulated samples (QIAgen Systems, Inc., Valencia, CA, USA).	Human	blood	NA	female	Iberian Population in Spain	normal weight	Overweight and obesity	96	0.69	0.29
EGAN00001617890	EGAN00001617890	Genomic DNA extracted from peripheral white blood cells using two kits, the Qiamp DNA Investigator Kit for coagulated samples and Qiamp DNA Mini & Blood Mini Kit for non-coagulated samples (QIAgen Systems, Inc., Valencia, CA, USA).	Human	blood	NA	female	Iberian Population in Spain	normal weight	Overweight and obesity	90	1.6	0
EGAN00001617891	EGAN00001617891	Genomic DNA extracted from peripheral white blood cells using two kits, the Qiamp DNA Investigator Kit for coagulated samples and Qiamp DNA Mini & Blood Mini Kit for non-coagulated samples (QIAgen Systems, Inc., Valencia, CA, USA).	Human	blood	NA	female	Iberian Population in Spain	normal weight	Overweight and obesity	86	0.42	−0.34
EGAN00001617892	EGAN00001617892	Genomic DNA extracted from peripheral white blood cells using two kits, the Qiamp DNA Investigator Kit for coagulated samples and Qiamp DNA Mini & Blood Mini Kit for non-coagulated samples (QIAgen Systems, Inc., Valencia, CA, USA).	Human	blood	NA	female	Iberian Population in Spain	normal weight	Overweight and obesity	87	0.6	−0.69
EGAN00001617893	EGAN00001617893	Genomic DNA extracted from peripheral white blood cells using two kits, the Qiamp DNA Investigator Kit for coagulated samples and Qiamp DNA Mini & Blood Mini Kit for non-coagulated samples (QIAgen Systems, Inc., Valencia, CA, USA).	Human	blood	NA	female	Iberian Population in Spain	normal weight	Overweight and obesity	80	0.82	−0.95
EGAN00001617894	EGAN00001617894	Genomic DNA extracted from peripheral white blood cells using two kits, the Qiamp DNA Investigator Kit for coagulated samples and Qiamp DNA Mini & Blood Mini Kit for non-coagulated samples (QIAgen Systems, Inc., Valencia, CA, USA).	Human	blood	NA	female	Iberian Population in Spain	normal weight	Overweight and obesity	80	1.52	0.35
EGAN00001617895	EGAN00001617895	Genomic DNA extracted from peripheral white blood cells using two kits, the Qiamp DNA Investigator Kit for coagulated samples and Qiamp DNA Mini & Blood Mini Kit for non-coagulated samples (QIAgen Systems, Inc., Valencia, CA, USA).	Human	blood	NA	female	Iberian Population in Spain	normal weight	Overweight and obesity	81	0.68	−0.22
EGAN00001617896	EGAN00001617896	Genomic DNA extracted from peripheral white blood cells using two kits, the Qiamp DNA Investigator Kit for coagulated samples and Qiamp DNA Mini & Blood Mini Kit for non-coagulated samples (QIAgen Systems, Inc., Valencia, CA, USA).	Human	blood	NA	female	Iberian Population in Spain	normal weight	Overweight and obesity	89	2.48	−0.38
EGAN00001617897	EGAN00001617897	Genomic DNA extracted from peripheral white blood cells using two kits, the Qiamp DNA Investigator Kit for coagulated samples and Qiamp DNA Mini & Blood Mini Kit for non-coagulated samples (QIAgen Systems, Inc., Valencia, CA, USA).	Human	blood	NA	female	Iberian Population in Spain	normal weight	Overweight and obesity	85	0.42	−0.97
EGAN00001617898	EGAN00001617898	Genomic DNA extracted from peripheral white blood cells using two kits, the Qiamp DNA Investigator Kit for coagulated samples and Qiamp DNA Mini & Blood Mini Kit for non-coagulated samples (QIAgen Systems, Inc., Valencia, CA, USA).	Human	blood	NA	female	Iberian Population in Spain	normal weight	Overweight and obesity	NA	NA	−0.2
EGAN00001617899	EGAN00001617899	Genomic DNA extracted from peripheral white blood cells using two kits, the Qiamp DNA Investigator Kit for coagulated samples and Qiamp DNA Mini & Blood Mini Kit for non-coagulated samples (QIAgen Systems, Inc., Valencia, CA, USA).	Human	blood	NA	female	Iberian Population in Spain	normal weight	Overweight and obesity	79	0.39	−0.25
EGAN00001617900	EGAN00001617900	Genomic DNA extracted from peripheral white blood cells using two kits, the Qiamp DNA Investigator Kit for coagulated samples and Qiamp DNA Mini & Blood Mini Kit for non-coagulated samples (QIAgen Systems, Inc., Valencia, CA, USA).	Human	blood	NA	female	Iberian Population in Spain	normal weight	Overweight and obesity	83	0.41	−0.47
EGAN00001617901	EGAN00001617901	Genomic DNA extracted from peripheral white blood cells using two kits, the Qiamp DNA Investigator Kit for coagulated samples and Qiamp DNA Mini & Blood Mini Kit for non-coagulated samples (QIAgen Systems, Inc., Valencia, CA, USA).	Human	blood	NA	female	Iberian Population in Spain	normal weight	Overweight and obesity	90	1.01	0.31
EGAN00001617902	EGAN00001617902	Genomic DNA extracted from peripheral white blood cells using two kits, the Qiamp DNA Investigator Kit for coagulated samples and Qiamp DNA Mini & Blood Mini Kit for non-coagulated samples (QIAgen Systems, Inc., Valencia, CA, USA).	Human	blood	NA	female	Iberian Population in Spain	normal weight	Overweight and obesity	83	0.41	−0.54
EGAN00001617903	EGAN00001617903	Genomic DNA extracted from peripheral white blood cells using two kits, the Qiamp DNA Investigator Kit for coagulated samples and Qiamp DNA Mini & Blood Mini Kit for non-coagulated samples (QIAgen Systems, Inc., Valencia, CA, USA).	Human	blood	NA	female	Iberian Population in Spain	normal weight	Overweight and obesity	88	0.43	−0.95
EGAN00001617904	EGAN00001617904	Genomic DNA extracted from peripheral white blood cells using two kits, the Qiamp DNA Investigator Kit for coagulated samples and Qiamp DNA Mini & Blood Mini Kit for non-coagulated samples (QIAgen Systems, Inc., Valencia, CA, USA).	Human	blood	NA	female	Iberian Population in Spain	normal weight	Overweight and obesity	92	4.19	0.46
EGAN00001617905	EGAN00001617905	Genomic DNA extracted from peripheral white blood cells using two kits, the Qiamp DNA Investigator Kit for coagulated samples and Qiamp DNA Mini & Blood Mini Kit for non-coagulated samples (QIAgen Systems, Inc., Valencia, CA, USA).	Human	blood	NA	female	Iberian Population in Spain	normal weight	Overweight and obesity	87	2.98	0.37
EGAN00001617906	EGAN00001617906	Genomic DNA extracted from peripheral white blood cells using two kits, the Qiamp DNA Investigator Kit for coagulated samples and Qiamp DNA Mini & Blood Mini Kit for non-coagulated samples (QIAgen Systems, Inc., Valencia, CA, USA).	Human	blood	NA	female	Iberian Population in Spain	normal weight	Overweight and obesity	88	1.78	−0.53
EGAN00001617907	EGAN00001617907	Genomic DNA extracted from peripheral white blood cells using two kits, the Qiamp DNA Investigator Kit for coagulated samples and Qiamp DNA Mini & Blood Mini Kit for non-coagulated samples (QIAgen Systems, Inc., Valencia, CA, USA).	Human	blood	NA	female	Iberian Population in Spain	normal weight	Overweight and obesity	92	2.68	−0.89
EGAN00001617908	EGAN00001617908	Genomic DNA extracted from peripheral white blood cells using two kits, the Qiamp DNA Investigator Kit for coagulated samples and Qiamp DNA Mini & Blood Mini Kit for non-coagulated samples (QIAgen Systems, Inc., Valencia, CA, USA).	Human	blood	NA	female	Iberian Population in Spain	normal weight	Overweight and obesity	90	3.26	−0.53
EGAN00001617909	EGAN00001617909	Genomic DNA extracted from peripheral white blood cells using two kits, the Qiamp DNA Investigator Kit for coagulated samples and Qiamp DNA Mini & Blood Mini Kit for non-coagulated samples (QIAgen Systems, Inc., Valencia, CA, USA).	Human	blood	NA	female	Iberian Population in Spain	normal weight	Overweight and obesity	84	1.1	−1.08
EGAN00001617910	EGAN00001617910	Genomic DNA extracted from peripheral white blood cells using two kits, the Qiamp DNA Investigator Kit for coagulated samples and Qiamp DNA Mini & Blood Mini Kit for non-coagulated samples (QIAgen Systems, Inc., Valencia, CA, USA).	Human	blood	NA	female	Iberian Population in Spain	normal weight	Overweight and obesity	94	4.65	−1.48
EGAN00001617911	EGAN00001617911	Genomic DNA extracted from peripheral white blood cells using two kits, the Qiamp DNA Investigator Kit for coagulated samples and Qiamp DNA Mini & Blood Mini Kit for non-coagulated samples (QIAgen Systems, Inc., Valencia, CA, USA).	Human	blood	NA	female	Iberian Population in Spain	normal weight	Overweight and obesity	91	2.62	−0.16
EGAN00001617912	EGAN00001617912	Genomic DNA extracted from peripheral white blood cells using two kits, the Qiamp DNA Investigator Kit for coagulated samples and Qiamp DNA Mini & Blood Mini Kit for non-coagulated samples (QIAgen Systems, Inc., Valencia, CA, USA).	Human	blood	NA	female	Iberian Population in Spain	normal weight	Overweight and obesity	97	2.62	0.34
EGAN00001617913	EGAN00001617913	Genomic DNA extracted from peripheral white blood cells using two kits, the Qiamp DNA Investigator Kit for coagulated samples and Qiamp DNA Mini & Blood Mini Kit for non-coagulated samples (QIAgen Systems, Inc., Valencia, CA, USA).	Human	blood	NA	female	Iberian Population in Spain	normal weight	Overweight and obesity	86	3.36	0.66
EGAN00001617914	EGAN00001617914	Genomic DNA extracted from peripheral white blood cells using two kits, the Qiamp DNA Investigator Kit for coagulated samples and Qiamp DNA Mini & Blood Mini Kit for non-coagulated samples (QIAgen Systems, Inc., Valencia, CA, USA).	Human	blood	NA	female	Iberian Population in Spain	normal weight	Overweight and obesity	94	3.26	0.39
EGAN00001617915	EGAN00001617915	Genomic DNA extracted from peripheral white blood cells using two kits, the Qiamp DNA Investigator Kit for coagulated samples and Qiamp DNA Mini & Blood Mini Kit for non-coagulated samples (QIAgen Systems, Inc., Valencia, CA, USA).	Human	blood	NA	female	Iberian Population in Spain	normal weight	Overweight and obesity	94	3.53	−0.46
EGAN00001617916	EGAN00001617916	Genomic DNA extracted from peripheral white blood cells using two kits, the Qiamp DNA Investigator Kit for coagulated samples and Qiamp DNA Mini & Blood Mini Kit for non-coagulated samples (QIAgen Systems, Inc., Valencia, CA, USA).	Human	blood	NA	female	Iberian Population in Spain	normal weight	Overweight and obesity	78	3.06	−0.81
EGAN00001617917	EGAN00001617917	Genomic DNA extracted from peripheral white blood cells using two kits, the Qiamp DNA Investigator Kit for coagulated samples and Qiamp DNA Mini & Blood Mini Kit for non-coagulated samples (QIAgen Systems, Inc., Valencia, CA, USA).	Human	blood	NA	female	Iberian Population in Spain	normal weight	Overweight and obesity	85	3.9	−0.35
EGAN00001617918	EGAN00001617918	Genomic DNA extracted from peripheral white blood cells using two kits, the Qiamp DNA Investigator Kit for coagulated samples and Qiamp DNA Mini & Blood Mini Kit for non-coagulated samples (QIAgen Systems, Inc., Valencia, CA, USA).	Human	blood	NA	female	Iberian Population in Spain	normal weight	Overweight and obesity	82	1.65	−0.28
EGAN00001617919	EGAN00001617919	Genomic DNA extracted from peripheral white blood cells using two kits, the Qiamp DNA Investigator Kit for coagulated samples and Qiamp DNA Mini & Blood Mini Kit for non-coagulated samples (QIAgen Systems, Inc., Valencia, CA, USA).	Human	blood	NA	female	Iberian Population in Spain	normal weight	Overweight and obesity	81	0.9	−0.79
EGAN00001617920	EGAN00001617920	Genomic DNA extracted from peripheral white blood cells using two kits, the Qiamp DNA Investigator Kit for coagulated samples and Qiamp DNA Mini & Blood Mini Kit for non-coagulated samples (QIAgen Systems, Inc., Valencia, CA, USA).	Human	blood	NA	female	Iberian Population in Spain	normal weight	Overweight and obesity	83	1.22	0.29
EGAN00001617921	EGAN00001617921	Genomic DNA extracted from peripheral white blood cells using two kits, the Qiamp DNA Investigator Kit for coagulated samples and Qiamp DNA Mini & Blood Mini Kit for non-coagulated samples (QIAgen Systems, Inc., Valencia, CA, USA).	Human	blood	NA	female	Iberian Population in Spain	normal weight	Overweight and obesity	82	1.93	−0.34
EGAN00001617922	EGAN00001617922	Genomic DNA extracted from peripheral white blood cells using two kits, the Qiamp DNA Investigator Kit for coagulated samples and Qiamp DNA Mini & Blood Mini Kit for non-coagulated samples (QIAgen Systems, Inc., Valencia, CA, USA).	Human	blood	NA	female	Iberian Population in Spain	normal weight	Overweight and obesity	88	2.34	−1.32
EGAN00001617923	EGAN00001617923	Genomic DNA extracted from peripheral white blood cells using two kits, the Qiamp DNA Investigator Kit for coagulated samples and Qiamp DNA Mini & Blood Mini Kit for non-coagulated samples (QIAgen Systems, Inc., Valencia, CA, USA).	Human	blood	NA	female	Iberian Population in Spain	normal weight	Overweight and obesity	84	2.23	−0.98
EGAN00001617924	EGAN00001617924	Genomic DNA extracted from peripheral white blood cells using two kits, the Qiamp DNA Investigator Kit for coagulated samples and Qiamp DNA Mini & Blood Mini Kit for non-coagulated samples (QIAgen Systems, Inc., Valencia, CA, USA).	Human	blood	NA	female	Iberian Population in Spain	normal weight	Overweight and obesity	78	1.25	−2.02
EGAN00001617925	EGAN00001617925	Genomic DNA extracted from peripheral white blood cells using two kits, the Qiamp DNA Investigator Kit for coagulated samples and Qiamp DNA Mini & Blood Mini Kit for non-coagulated samples (QIAgen Systems, Inc., Valencia, CA, USA).	Human	blood	NA	female	Iberian Population in Spain	normal weight	Overweight and obesity	79	3.32	−0.63
EGAN00001617926	EGAN00001617926	Genomic DNA extracted from peripheral white blood cells using two kits, the Qiamp DNA Investigator Kit for coagulated samples and Qiamp DNA Mini & Blood Mini Kit for non-coagulated samples (QIAgen Systems, Inc., Valencia, CA, USA).	Human	blood	NA	female	Iberian Population in Spain	normal weight	Overweight and obesity	79	1.8	−0.45
EGAN00001617927	EGAN00001617927	Genomic DNA extracted from peripheral white blood cells using two kits, the Qiamp DNA Investigator Kit for coagulated samples and Qiamp DNA Mini & Blood Mini Kit for non-coagulated samples (QIAgen Systems, Inc., Valencia, CA, USA).	Human	blood	NA	female	Iberian Population in Spain	normal weight	Overweight and obesity	89	1.58	−0.26
EGAN00001617928	EGAN00001617928	Genomic DNA extracted from peripheral white blood cells using two kits, the Qiamp DNA Investigator Kit for coagulated samples and Qiamp DNA Mini & Blood Mini Kit for non-coagulated samples (QIAgen Systems, Inc., Valencia, CA, USA).	Human	blood	NA	female	Iberian Population in Spain	normal weight	Overweight and obesity	90	0.84	0.01
EGAN00001617929	EGAN00001617929	Genomic DNA extracted from peripheral white blood cells using two kits, the Qiamp DNA Investigator Kit for coagulated samples and Qiamp DNA Mini & Blood Mini Kit for non-coagulated samples (QIAgen Systems, Inc., Valencia, CA, USA).	Human	blood	NA	female	Iberian Population in Spain	normal weight	Overweight and obesity	80	2.05	−0.32
EGAN00001617930	EGAN00001617930	Genomic DNA extracted from peripheral white blood cells using two kits, the Qiamp DNA Investigator Kit for coagulated samples and Qiamp DNA Mini & Blood Mini Kit for non-coagulated samples (QIAgen Systems, Inc., Valencia, CA, USA).	Human	blood	NA	female	Iberian Population in Spain	normal weight	Overweight and obesity	89	3.86	−0.43
EGAN00001617931	EGAN00001617931	Genomic DNA extracted from peripheral white blood cells using two kits, the Qiamp DNA Investigator Kit for coagulated samples and Qiamp DNA Mini & Blood Mini Kit for non-coagulated samples (QIAgen Systems, Inc., Valencia, CA, USA).	Human	blood	NA	female	Iberian Population in Spain	normal weight	Overweight and obesity	87	3.16	−0.1
EGAN00001617932	EGAN00001617932	Genomic DNA extracted from peripheral white blood cells using two kits, the Qiamp DNA Investigator Kit for coagulated samples and Qiamp DNA Mini & Blood Mini Kit for non-coagulated samples (QIAgen Systems, Inc., Valencia, CA, USA).	Human	blood	NA	female	Iberian Population in Spain	normal weight	Overweight and obesity	78	1.94	−0.69
EGAN00001617933	EGAN00001617933	Genomic DNA extracted from peripheral white blood cells using two kits, the Qiamp DNA Investigator Kit for coagulated samples and Qiamp DNA Mini & Blood Mini Kit for non-coagulated samples (QIAgen Systems, Inc., Valencia, CA, USA).	Human	blood	NA	female	Iberian Population in Spain	normal weight	Overweight and obesity	87	0.87	−1.39
EGAN00001617934	EGAN00001617934	Genomic DNA extracted from peripheral white blood cells using two kits, the Qiamp DNA Investigator Kit for coagulated samples and Qiamp DNA Mini & Blood Mini Kit for non-coagulated samples (QIAgen Systems, Inc., Valencia, CA, USA).	Human	blood	NA	female	Iberian Population in Spain	normal weight	Overweight and obesity	84	3.29	0.07
EGAN00001617935	EGAN00001617935	Genomic DNA extracted from peripheral white blood cells using two kits, the Qiamp DNA Investigator Kit for coagulated samples and Qiamp DNA Mini & Blood Mini Kit for non-coagulated samples (QIAgen Systems, Inc., Valencia, CA, USA).	Human	blood	NA	female	Iberian Population in Spain	normal weight	Overweight and obesity	77	1.62	−0.23
EGAN00001617936	EGAN00001617936	Genomic DNA extracted from peripheral white blood cells using two kits, the Qiamp DNA Investigator Kit for coagulated samples and Qiamp DNA Mini & Blood Mini Kit for non-coagulated samples (QIAgen Systems, Inc., Valencia, CA, USA).	Human	blood	NA	female	Iberian Population in Spain	normal weight	Overweight and obesity	78	1.59	−0.09
EGAN00001617937	EGAN00001617937	Genomic DNA extracted from peripheral white blood cells using two kits, the Qiamp DNA Investigator Kit for coagulated samples and Qiamp DNA Mini & Blood Mini Kit for non-coagulated samples (QIAgen Systems, Inc., Valencia, CA, USA).	Human	blood	NA	female	Iberian Population in Spain	normal weight	Overweight and obesity	93	3.5	0.25
EGAN00001617938	EGAN00001617938	Genomic DNA extracted from peripheral white blood cells using two kits, the Qiamp DNA Investigator Kit for coagulated samples and Qiamp DNA Mini & Blood Mini Kit for non-coagulated samples (QIAgen Systems, Inc., Valencia, CA, USA).	Human	blood	NA	female	Iberian Population in Spain	normal weight	Overweight and obesity	94	3.68	0.52
EGAN00001617939	EGAN00001617939	Genomic DNA extracted from peripheral white blood cells using two kits, the Qiamp DNA Investigator Kit for coagulated samples and Qiamp DNA Mini & Blood Mini Kit for non-coagulated samples (QIAgen Systems, Inc., Valencia, CA, USA).	Human	blood	NA	female	Iberian Population in Spain	normal weight	Overweight and obesity	88	3.17	0.13
EGAN00001617940	EGAN00001617940	Genomic DNA extracted from peripheral white blood cells using two kits, the Qiamp DNA Investigator Kit for coagulated samples and Qiamp DNA Mini & Blood Mini Kit for non-coagulated samples (QIAgen Systems, Inc., Valencia, CA, USA).	Human	blood	NA	female	Iberian Population in Spain	normal weight	Overweight and obesity	90	1.59	−0.41
EGAN00001617941	EGAN00001617941	Genomic DNA extracted from peripheral white blood cells using two kits, the Qiamp DNA Investigator Kit for coagulated samples and Qiamp DNA Mini & Blood Mini Kit for non-coagulated samples (QIAgen Systems, Inc., Valencia, CA, USA).	Human	blood	NA	female	Iberian Population in Spain	normal weight	Overweight and obesity	88	1.46	−0.44
EGAN00001617942	EGAN00001617942	Genomic DNA extracted from peripheral white blood cells using two kits, the Qiamp DNA Investigator Kit for coagulated samples and Qiamp DNA Mini & Blood Mini Kit for non-coagulated samples (QIAgen Systems, Inc., Valencia, CA, USA).	Human	blood	NA	female	Iberian Population in Spain	normal weight	Overweight and obesity	83	2,746	−0.63
EGAN00001617943	EGAN00001617943	Genomic DNA extracted from peripheral white blood cells using two kits, the Qiamp DNA Investigator Kit for coagulated samples and Qiamp DNA Mini & Blood Mini Kit for non-coagulated samples (QIAgen Systems, Inc., Valencia, CA, USA).	Human	blood	NA	male	Iberian Population in Spain	normal weight	Overweight and obesity	85	0.92	−0.09
EGAN00001617944	EGAN00001617944	Genomic DNA extracted from peripheral white blood cells using two kits, the Qiamp DNA Investigator Kit for coagulated samples and Qiamp DNA Mini & Blood Mini Kit for non-coagulated samples (QIAgen Systems, Inc., Valencia, CA, USA).	Human	blood	NA	male	Iberian Population in Spain	normal weight	Overweight and obesity	79	0.45	−0.52
EGAN00001617945	EGAN00001617945	Genomic DNA extracted from peripheral white blood cells using two kits, the Qiamp DNA Investigator Kit for coagulated samples and Qiamp DNA Mini & Blood Mini Kit for non-coagulated samples (QIAgen Systems, Inc., Valencia, CA, USA).	Human	blood	NA	male	Iberian Population in Spain	normal weight	Overweight and obesity	76	0.37	−1.49
EGAN00001617946	EGAN00001617946	Genomic DNA extracted from peripheral white blood cells using two kits, the Qiamp DNA Investigator Kit for coagulated samples and Qiamp DNA Mini & Blood Mini Kit for non-coagulated samples (QIAgen Systems, Inc., Valencia, CA, USA).	Human	blood	NA	male	Iberian Population in Spain	normal weight	Overweight and obesity	88	1.74	0.08
EGAN00001617947	EGAN00001617947	Genomic DNA extracted from peripheral white blood cells using two kits, the Qiamp DNA Investigator Kit for coagulated samples and Qiamp DNA Mini & Blood Mini Kit for non-coagulated samples (QIAgen Systems, Inc., Valencia, CA, USA).	Human	blood	NA	male	Iberian Population in Spain	normal weight	Overweight and obesity	101	1.02	−0.43
EGAN00001617948	EGAN00001617948	Genomic DNA extracted from peripheral white blood cells using two kits, the Qiamp DNA Investigator Kit for coagulated samples and Qiamp DNA Mini & Blood Mini Kit for non-coagulated samples (QIAgen Systems, Inc., Valencia, CA, USA).	Human	blood	NA	male	Iberian Population in Spain	normal weight	Overweight and obesity	77	0.88	−0.97
EGAN00001617949	EGAN00001617949	Genomic DNA extracted from peripheral white blood cells using two kits, the Qiamp DNA Investigator Kit for coagulated samples and Qiamp DNA Mini & Blood Mini Kit for non-coagulated samples (QIAgen Systems, Inc., Valencia, CA, USA).	Human	blood	NA	male	Iberian Population in Spain	normal weight	Overweight and obesity	83	0.7	−0.81
EGAN00001617950	EGAN00001617950	Genomic DNA extracted from peripheral white blood cells using two kits, the Qiamp DNA Investigator Kit for coagulated samples and Qiamp DNA Mini & Blood Mini Kit for non-coagulated samples (QIAgen Systems, Inc., Valencia, CA, USA).	Human	blood	NA	male	Iberian Population in Spain	normal weight	Overweight and obesity	72	0.34	0.6
EGAN00001617951	EGAN00001617951	Genomic DNA extracted from peripheral white blood cells using two kits, the Qiamp DNA Investigator Kit for coagulated samples and Qiamp DNA Mini & Blood Mini Kit for non-coagulated samples (QIAgen Systems, Inc., Valencia, CA, USA).	Human	blood	NA	male	Iberian Population in Spain	normal weight	Overweight and obesity	85	0.9	0.62
EGAN00001617952	EGAN00001617952	Genomic DNA extracted from peripheral white blood cells using two kits, the Qiamp DNA Investigator Kit for coagulated samples and Qiamp DNA Mini & Blood Mini Kit for non-coagulated samples (QIAgen Systems, Inc., Valencia, CA, USA).	Human	blood	NA	male	Iberian Population in Spain	normal weight	Overweight and obesity	87	1.5	−0.77
EGAN00001617953	EGAN00001617953	Genomic DNA extracted from peripheral white blood cells using two kits, the Qiamp DNA Investigator Kit for coagulated samples and Qiamp DNA Mini & Blood Mini Kit for non-coagulated samples (QIAgen Systems, Inc., Valencia, CA, USA).	Human	blood	NA	male	Iberian Population in Spain	normal weight	Overweight and obesity	87	1.2	−0.79
EGAN00001617954	EGAN00001617954	Genomic DNA extracted from peripheral white blood cells using two kits, the Qiamp DNA Investigator Kit for coagulated samples and Qiamp DNA Mini & Blood Mini Kit for non-coagulated samples (QIAgen Systems, Inc., Valencia, CA, USA).	Human	blood	NA	male	Iberian Population in Spain	normal weight	Overweight and obesity	87	1.35	−0.56
EGAN00001617955	EGAN00001617955	Genomic DNA extracted from peripheral white blood cells using two kits, the Qiamp DNA Investigator Kit for coagulated samples and Qiamp DNA Mini & Blood Mini Kit for non-coagulated samples (QIAgen Systems, Inc., Valencia, CA, USA).	Human	blood	NA	male	Iberian Population in Spain	normal weight	Overweight and obesity	81	1.2	0.15
EGAN00001617956	EGAN00001617956	Genomic DNA extracted from peripheral white blood cells using two kits, the Qiamp DNA Investigator Kit for coagulated samples and Qiamp DNA Mini & Blood Mini Kit for non-coagulated samples (QIAgen Systems, Inc., Valencia, CA, USA).	Human	blood	NA	male	Iberian Population in Spain	normal weight	Overweight and obesity	82	1.15	0.06
EGAN00001617957	EGAN00001617957	Genomic DNA extracted from peripheral white blood cells using two kits, the Qiamp DNA Investigator Kit for coagulated samples and Qiamp DNA Mini & Blood Mini Kit for non-coagulated samples (QIAgen Systems, Inc., Valencia, CA, USA).	Human	blood	NA	male	Iberian Population in Spain	normal weight	Overweight and obesity	86	0.79	−0.29
EGAN00001617958	EGAN00001617958	Genomic DNA extracted from peripheral white blood cells using two kits, the Qiamp DNA Investigator Kit for coagulated samples and Qiamp DNA Mini & Blood Mini Kit for non-coagulated samples (QIAgen Systems, Inc., Valencia, CA, USA).	Human	blood	NA	male	Iberian Population in Spain	normal weight	Overweight and obesity	91	0.97	0.84
EGAN00001617959	EGAN00001617959	Genomic DNA extracted from peripheral white blood cells using two kits, the Qiamp DNA Investigator Kit for coagulated samples and Qiamp DNA Mini & Blood Mini Kit for non-coagulated samples (QIAgen Systems, Inc., Valencia, CA, USA).	Human	blood	NA	male	Iberian Population in Spain	normal weight	Overweight and obesity	99	2.4	0.41
EGAN00001617960	EGAN00001617960	Genomic DNA extracted from peripheral white blood cells using two kits, the Qiamp DNA Investigator Kit for coagulated samples and Qiamp DNA Mini & Blood Mini Kit for non-coagulated samples (QIAgen Systems, Inc., Valencia, CA, USA).	Human	blood	NA	male	Iberian Population in Spain	normal weight	Overweight and obesity	80	0.67	−0.33
EGAN00001617961	EGAN00001617961	Genomic DNA extracted from peripheral white blood cells using two kits, the Qiamp DNA Investigator Kit for coagulated samples and Qiamp DNA Mini & Blood Mini Kit for non-coagulated samples (QIAgen Systems, Inc., Valencia, CA, USA).	Human	blood	NA	male	Iberian Population in Spain	normal weight	Overweight and obesity	82	1.15	−0.63
EGAN00001617962	EGAN00001617962	Genomic DNA extracted from peripheral white blood cells using two kits, the Qiamp DNA Investigator Kit for coagulated samples and Qiamp DNA Mini & Blood Mini Kit for non-coagulated samples (QIAgen Systems, Inc., Valencia, CA, USA).	Human	blood	NA	male	Iberian Population in Spain	normal weight	Overweight and obesity	76	1.67	−0.34
EGAN00001617963	EGAN00001617963	Genomic DNA extracted from peripheral white blood cells using two kits, the Qiamp DNA Investigator Kit for coagulated samples and Qiamp DNA Mini & Blood Mini Kit for non-coagulated samples (QIAgen Systems, Inc., Valencia, CA, USA).	Human	blood	NA	male	Iberian Population in Spain	normal weight	Overweight and obesity	69	0.26	−0.69
EGAN00001617964	EGAN00001617964	Genomic DNA extracted from peripheral white blood cells using two kits, the Qiamp DNA Investigator Kit for coagulated samples and Qiamp DNA Mini & Blood Mini Kit for non-coagulated samples (QIAgen Systems, Inc., Valencia, CA, USA).	Human	blood	NA	male	Iberian Population in Spain	normal weight	Overweight and obesity	88	1.41	0.5
EGAN00001617965	EGAN00001617965	Genomic DNA extracted from peripheral white blood cells using two kits, the Qiamp DNA Investigator Kit for coagulated samples and Qiamp DNA Mini & Blood Mini Kit for non-coagulated samples (QIAgen Systems, Inc., Valencia, CA, USA).	Human	blood	NA	male	Iberian Population in Spain	normal weight	Overweight and obesity	74	0.57	−0.1
EGAN00001617966	EGAN00001617966	Genomic DNA extracted from peripheral white blood cells using two kits, the Qiamp DNA Investigator Kit for coagulated samples and Qiamp DNA Mini & Blood Mini Kit for non-coagulated samples (QIAgen Systems, Inc., Valencia, CA, USA).	Human	blood	NA	male	Iberian Population in Spain	normal weight	Overweight and obesity	87	1.18	−0.22
EGAN00001617967	EGAN00001617967	Genomic DNA extracted from peripheral white blood cells using two kits, the Qiamp DNA Investigator Kit for coagulated samples and Qiamp DNA Mini & Blood Mini Kit for non-coagulated samples (QIAgen Systems, Inc., Valencia, CA, USA).	Human	blood	NA	male	Iberian Population in Spain	normal weight	Overweight and obesity	92	2.29	0.49
EGAN00001617968	EGAN00001617968	Genomic DNA extracted from peripheral white blood cells using two kits, the Qiamp DNA Investigator Kit for coagulated samples and Qiamp DNA Mini & Blood Mini Kit for non-coagulated samples (QIAgen Systems, Inc., Valencia, CA, USA).	Human	blood	NA	male	Iberian Population in Spain	normal weight	Overweight and obesity	83	0.7	0.06
EGAN00001617969	EGAN00001617969	Genomic DNA extracted from peripheral white blood cells using two kits, the Qiamp DNA Investigator Kit for coagulated samples and Qiamp DNA Mini & Blood Mini Kit for non-coagulated samples (QIAgen Systems, Inc., Valencia, CA, USA).	Human	blood	NA	male	Iberian Population in Spain	normal weight	Overweight and obesity	80	0.87	−0.77
EGAN00001617970	EGAN00001617970	Genomic DNA extracted from peripheral white blood cells using two kits, the Qiamp DNA Investigator Kit for coagulated samples and Qiamp DNA Mini & Blood Mini Kit for non-coagulated samples (QIAgen Systems, Inc., Valencia, CA, USA).	Human	blood	NA	male	Iberian Population in Spain	normal weight	Overweight and obesity	88	1.74	−0.37
EGAN00001617971	EGAN00001617971	Genomic DNA extracted from peripheral white blood cells using two kits, the Qiamp DNA Investigator Kit for coagulated samples and Qiamp DNA Mini & Blood Mini Kit for non-coagulated samples (QIAgen Systems, Inc., Valencia, CA, USA).	Human	blood	NA	male	Iberian Population in Spain	normal weight	Overweight and obesity	89	0.85	0.31
EGAN00001617972	EGAN00001617972	Genomic DNA extracted from peripheral white blood cells using two kits, the Qiamp DNA Investigator Kit for coagulated samples and Qiamp DNA Mini & Blood Mini Kit for non-coagulated samples (QIAgen Systems, Inc., Valencia, CA, USA).	Human	blood	NA	male	Iberian Population in Spain	normal weight	Overweight and obesity	79	1.11	−0.81
EGAN00001617973	EGAN00001617973	Genomic DNA extracted from peripheral white blood cells using two kits, the Qiamp DNA Investigator Kit for coagulated samples and Qiamp DNA Mini & Blood Mini Kit for non-coagulated samples (QIAgen Systems, Inc., Valencia, CA, USA).	Human	blood	NA	male	Iberian Population in Spain	normal weight	Overweight and obesity	84	1.29	−0.88
EGAN00001617974	EGAN00001617974	Genomic DNA extracted from peripheral white blood cells using two kits, the Qiamp DNA Investigator Kit for coagulated samples and Qiamp DNA Mini & Blood Mini Kit for non-coagulated samples (QIAgen Systems, Inc., Valencia, CA, USA).	Human	blood	NA	male	Iberian Population in Spain	normal weight	Overweight and obesity	74	0.71	−0.58
EGAN00001617975	EGAN00001617975	Genomic DNA extracted from peripheral white blood cells using two kits, the Qiamp DNA Investigator Kit for coagulated samples and Qiamp DNA Mini & Blood Mini Kit for non-coagulated samples (QIAgen Systems, Inc., Valencia, CA, USA).	Human	blood	NA	male	Iberian Population in Spain	normal weight	Overweight and obesity	92	2.25	0.46
EGAN00001617976	EGAN00001617976	Genomic DNA extracted from peripheral white blood cells using two kits, the Qiamp DNA Investigator Kit for coagulated samples and Qiamp DNA Mini & Blood Mini Kit for non-coagulated samples (QIAgen Systems, Inc., Valencia, CA, USA).	Human	blood	NA	male	Iberian Population in Spain	normal weight	Overweight and obesity	97	2.11	−1.05
EGAN00001617977	EGAN00001617977	Genomic DNA extracted from peripheral white blood cells using two kits, the Qiamp DNA Investigator Kit for coagulated samples and Qiamp DNA Mini & Blood Mini Kit for non-coagulated samples (QIAgen Systems, Inc., Valencia, CA, USA).	Human	blood	NA	male	Iberian Population in Spain	normal weight	Overweight and obesity	81	0.78	0.48
EGAN00001617978	EGAN00001617978	Genomic DNA extracted from peripheral white blood cells using two kits, the Qiamp DNA Investigator Kit for coagulated samples and Qiamp DNA Mini & Blood Mini Kit for non-coagulated samples (QIAgen Systems, Inc., Valencia, CA, USA).	Human	blood	NA	male	Iberian Population in Spain	normal weight	Overweight and obesity	86	1.32	0.64
EGAN00001617979	EGAN00001617979	Genomic DNA extracted from peripheral white blood cells using two kits, the Qiamp DNA Investigator Kit for coagulated samples and Qiamp DNA Mini & Blood Mini Kit for non-coagulated samples (QIAgen Systems, Inc., Valencia, CA, USA).	Human	blood	NA	male	Iberian Population in Spain	normal weight	Overweight and obesity	84	1.41	0.54
EGAN00001617980	EGAN00001617980	Genomic DNA extracted from peripheral white blood cells using two kits, the Qiamp DNA Investigator Kit for coagulated samples and Qiamp DNA Mini & Blood Mini Kit for non-coagulated samples (QIAgen Systems, Inc., Valencia, CA, USA).	Human	blood	NA	male	Iberian Population in Spain	normal weight	Overweight and obesity	96	NA	−0.06
EGAN00001617981	EGAN00001617981	Genomic DNA extracted from peripheral white blood cells using two kits, the Qiamp DNA Investigator Kit for coagulated samples and Qiamp DNA Mini & Blood Mini Kit for non-coagulated samples (QIAgen Systems, Inc., Valencia, CA, USA).	Human	blood	NA	male	Iberian Population in Spain	normal weight	Overweight and obesity	98	1.21	−0.12
EGAN00001617982	EGAN00001617982	Genomic DNA extracted from peripheral white blood cells using two kits, the Qiamp DNA Investigator Kit for coagulated samples and Qiamp DNA Mini & Blood Mini Kit for non-coagulated samples (QIAgen Systems, Inc., Valencia, CA, USA).	Human	blood	NA	male	Iberian Population in Spain	normal weight	Overweight and obesity	76	0.37	0.63
EGAN00001617983	EGAN00001617983	Genomic DNA extracted from peripheral white blood cells using two kits, the Qiamp DNA Investigator Kit for coagulated samples and Qiamp DNA Mini & Blood Mini Kit for non-coagulated samples (QIAgen Systems, Inc., Valencia, CA, USA).	Human	blood	NA	male	Iberian Population in Spain	normal weight	Overweight and obesity	76	1.28	−0.07
EGAN00001617984	EGAN00001617984	Genomic DNA extracted from peripheral white blood cells using two kits, the Qiamp DNA Investigator Kit for coagulated samples and Qiamp DNA Mini & Blood Mini Kit for non-coagulated samples (QIAgen Systems, Inc., Valencia, CA, USA).	Human	blood	NA	male	Iberian Population in Spain	normal weight	Overweight and obesity	80	0.77	0.39
EGAN00001617985	EGAN00001617985	Genomic DNA extracted from peripheral white blood cells using two kits, the Qiamp DNA Investigator Kit for coagulated samples and Qiamp DNA Mini & Blood Mini Kit for non-coagulated samples (QIAgen Systems, Inc., Valencia, CA, USA).	Human	blood	NA	male	Iberian Population in Spain	normal weight	Overweight and obesity	85	2.85	0.48
EGAN00001617986	EGAN00001617986	Genomic DNA extracted from peripheral white blood cells using two kits, the Qiamp DNA Investigator Kit for coagulated samples and Qiamp DNA Mini & Blood Mini Kit for non-coagulated samples (QIAgen Systems, Inc., Valencia, CA, USA).	Human	blood	NA	male	Iberian Population in Spain	normal weight	Overweight and obesity	89	0.44	−0.26
EGAN00001617987	EGAN00001617987	Genomic DNA extracted from peripheral white blood cells using two kits, the Qiamp DNA Investigator Kit for coagulated samples and Qiamp DNA Mini & Blood Mini Kit for non-coagulated samples (QIAgen Systems, Inc., Valencia, CA, USA).	Human	blood	NA	male	Iberian Population in Spain	normal weight	Overweight and obesity	82	1.05	0.69
EGAN00001617988	EGAN00001617988	Genomic DNA extracted from peripheral white blood cells using two kits, the Qiamp DNA Investigator Kit for coagulated samples and Qiamp DNA Mini & Blood Mini Kit for non-coagulated samples (QIAgen Systems, Inc., Valencia, CA, USA).	Human	blood	NA	male	Iberian Population in Spain	normal weight	Overweight and obesity	79	0.66	−0.51
EGAN00001617989	EGAN00001617989	Genomic DNA extracted from peripheral white blood cells using two kits, the Qiamp DNA Investigator Kit for coagulated samples and Qiamp DNA Mini & Blood Mini Kit for non-coagulated samples (QIAgen Systems, Inc., Valencia, CA, USA).	Human	blood	NA	male	Iberian Population in Spain	normal weight	Overweight and obesity	74	0.42	−0.48
EGAN00001617990	EGAN00001617990	Genomic DNA extracted from peripheral white blood cells using two kits, the Qiamp DNA Investigator Kit for coagulated samples and Qiamp DNA Mini & Blood Mini Kit for non-coagulated samples (QIAgen Systems, Inc., Valencia, CA, USA).	Human	blood	NA	male	Iberian Population in Spain	normal weight	Overweight and obesity	87	1.29	−0.97
EGAN00001617991	EGAN00001617991	Genomic DNA extracted from peripheral white blood cells using two kits, the Qiamp DNA Investigator Kit for coagulated samples and Qiamp DNA Mini & Blood Mini Kit for non-coagulated samples (QIAgen Systems, Inc., Valencia, CA, USA).	Human	blood	NA	male	Iberian Population in Spain	normal weight	Overweight and obesity	81	1	−0.08
EGAN00001617992	EGAN00001617992	Genomic DNA extracted from peripheral white blood cells using two kits, the Qiamp DNA Investigator Kit for coagulated samples and Qiamp DNA Mini & Blood Mini Kit for non-coagulated samples (QIAgen Systems, Inc., Valencia, CA, USA).	Human	blood	NA	male	Iberian Population in Spain	normal weight	Overweight and obesity	82	0.45	−0.8
EGAN00001617993	EGAN00001617993	Genomic DNA extracted from peripheral white blood cells using two kits, the Qiamp DNA Investigator Kit for coagulated samples and Qiamp DNA Mini & Blood Mini Kit for non-coagulated samples (QIAgen Systems, Inc., Valencia, CA, USA).	Human	blood	NA	male	Iberian Population in Spain	normal weight	Overweight and obesity	70	0.33	−0.78
EGAN00001617994	EGAN00001617994	Genomic DNA extracted from peripheral white blood cells using two kits, the Qiamp DNA Investigator Kit for coagulated samples and Qiamp DNA Mini & Blood Mini Kit for non-coagulated samples (QIAgen Systems, Inc., Valencia, CA, USA).	Human	blood	NA	male	Iberian Population in Spain	normal weight	Overweight and obesity	90	3.39	0.5
EGAN00001617995	EGAN00001617995	Genomic DNA extracted from peripheral white blood cells using two kits, the Qiamp DNA Investigator Kit for coagulated samples and Qiamp DNA Mini & Blood Mini Kit for non-coagulated samples (QIAgen Systems, Inc., Valencia, CA, USA).	Human	blood	NA	male	Iberian Population in Spain	normal weight	Overweight and obesity	80	1.17	0.42
EGAN00001617996	EGAN00001617996	Genomic DNA extracted from peripheral white blood cells using two kits, the Qiamp DNA Investigator Kit for coagulated samples and Qiamp DNA Mini & Blood Mini Kit for non-coagulated samples (QIAgen Systems, Inc., Valencia, CA, USA).	Human	blood	NA	male	Iberian Population in Spain	normal weight	Overweight and obesity	80	0.8	0.36
EGAN00001617997	EGAN00001617997	Genomic DNA extracted from peripheral white blood cells using two kits, the Qiamp DNA Investigator Kit for coagulated samples and Qiamp DNA Mini & Blood Mini Kit for non-coagulated samples (QIAgen Systems, Inc., Valencia, CA, USA).	Human	blood	NA	male	Iberian Population in Spain	normal weight	Overweight and obesity	95	0.89	0.18
EGAN00001617998	EGAN00001617998	Genomic DNA extracted from peripheral white blood cells using two kits, the Qiamp DNA Investigator Kit for coagulated samples and Qiamp DNA Mini & Blood Mini Kit for non-coagulated samples (QIAgen Systems, Inc., Valencia, CA, USA).	Human	blood	NA	male	Iberian Population in Spain	normal weight	Overweight and obesity	89	0.94	−0.33
EGAN00001617999	EGAN00001617999	Genomic DNA extracted from peripheral white blood cells using two kits, the Qiamp DNA Investigator Kit for coagulated samples and Qiamp DNA Mini & Blood Mini Kit for non-coagulated samples (QIAgen Systems, Inc., Valencia, CA, USA).	Human	blood	NA	male	Iberian Population in Spain	normal weight	Overweight and obesity	86	1.04	−1.01
EGAN00001618000	EGAN00001618000	Genomic DNA extracted from peripheral white blood cells using two kits, the Qiamp DNA Investigator Kit for coagulated samples and Qiamp DNA Mini & Blood Mini Kit for non-coagulated samples (QIAgen Systems, Inc., Valencia, CA, USA).	Human	blood	NA	male	Iberian Population in Spain	normal weight	Overweight and obesity	85	1.11	−0.75
EGAN00001618001	EGAN00001618001	Genomic DNA extracted from peripheral white blood cells using two kits, the Qiamp DNA Investigator Kit for coagulated samples and Qiamp DNA Mini & Blood Mini Kit for non-coagulated samples (QIAgen Systems, Inc., Valencia, CA, USA).	Human	blood	NA	male	Iberian Population in Spain	normal weight	Overweight and obesity	87	1.57	0.28
EGAN00001618002	EGAN00001618002	Genomic DNA extracted from peripheral white blood cells using two kits, the Qiamp DNA Investigator Kit for coagulated samples and Qiamp DNA Mini & Blood Mini Kit for non-coagulated samples (QIAgen Systems, Inc., Valencia, CA, USA).	Human	blood	NA	male	Iberian Population in Spain	normal weight	Overweight and obesity	86	0.63	−0.47
EGAN00001618003	EGAN00001618003	Genomic DNA extracted from peripheral white blood cells using two kits, the Qiamp DNA Investigator Kit for coagulated samples and Qiamp DNA Mini & Blood Mini Kit for non-coagulated samples (QIAgen Systems, Inc., Valencia, CA, USA).	Human	blood	NA	male	Iberian Population in Spain	normal weight	Overweight and obesity	79	0.69	0.12
EGAN00001618004	EGAN00001618004	Genomic DNA extracted from peripheral white blood cells using two kits, the Qiamp DNA Investigator Kit for coagulated samples and Qiamp DNA Mini & Blood Mini Kit for non-coagulated samples (QIAgen Systems, Inc., Valencia, CA, USA).	Human	blood	NA	male	Iberian Population in Spain	normal weight	Overweight and obesity	85	2.2	−0.14
EGAN00001618005	EGAN00001618005	Genomic DNA extracted from peripheral white blood cells using two kits, the Qiamp DNA Investigator Kit for coagulated samples and Qiamp DNA Mini & Blood Mini Kit for non-coagulated samples (QIAgen Systems, Inc., Valencia, CA, USA).	Human	blood	NA	male	Iberian Population in Spain	normal weight	Overweight and obesity	82	0.65	0.28
EGAN00001618006	EGAN00001618006	Genomic DNA extracted from peripheral white blood cells using two kits, the Qiamp DNA Investigator Kit for coagulated samples and Qiamp DNA Mini & Blood Mini Kit for non-coagulated samples (QIAgen Systems, Inc., Valencia, CA, USA).	Human	blood	NA	male	Iberian Population in Spain	normal weight	Overweight and obesity	82	0.57	0.11
EGAN00001618007	EGAN00001618007	Genomic DNA extracted from peripheral white blood cells using two kits, the Qiamp DNA Investigator Kit for coagulated samples and Qiamp DNA Mini & Blood Mini Kit for non-coagulated samples (QIAgen Systems, Inc., Valencia, CA, USA).	Human	blood	NA	male	Iberian Population in Spain	normal weight	Overweight and obesity	73	0.51	−0.41
EGAN00001618008	EGAN00001618008	Genomic DNA extracted from peripheral white blood cells using two kits, the Qiamp DNA Investigator Kit for coagulated samples and Qiamp DNA Mini & Blood Mini Kit for non-coagulated samples (QIAgen Systems, Inc., Valencia, CA, USA).	Human	blood	NA	male	Iberian Population in Spain	normal weight	Overweight and obesity	71	0.65	−0.71
EGAN00001618009	EGAN00001618009	Genomic DNA extracted from peripheral white blood cells using two kits, the Qiamp DNA Investigator Kit for coagulated samples and Qiamp DNA Mini & Blood Mini Kit for non-coagulated samples (QIAgen Systems, Inc., Valencia, CA, USA).	Human	blood	NA	male	Iberian Population in Spain	normal weight	Overweight and obesity	78	0.6	−0.41
EGAN00001618010	EGAN00001618010	Genomic DNA extracted from peripheral white blood cells using two kits, the Qiamp DNA Investigator Kit for coagulated samples and Qiamp DNA Mini & Blood Mini Kit for non-coagulated samples (QIAgen Systems, Inc., Valencia, CA, USA).	Human	blood	NA	male	Iberian Population in Spain	normal weight	Overweight and obesity	78	1.33	−0.57
EGAN00001618011	EGAN00001618011	Genomic DNA extracted from peripheral white blood cells using two kits, the Qiamp DNA Investigator Kit for coagulated samples and Qiamp DNA Mini & Blood Mini Kit for non-coagulated samples (QIAgen Systems, Inc., Valencia, CA, USA).	Human	blood	NA	male	Iberian Population in Spain	normal weight	Overweight and obesity	83	1.14	−0.4
EGAN00001618012	EGAN00001618012	Genomic DNA extracted from peripheral white blood cells using two kits, the Qiamp DNA Investigator Kit for coagulated samples and Qiamp DNA Mini & Blood Mini Kit for non-coagulated samples (QIAgen Systems, Inc., Valencia, CA, USA).	Human	blood	NA	male	Iberian Population in Spain	normal weight	Overweight and obesity	85	1.17	−0.43
EGAN00001618013	EGAN00001618013	Genomic DNA extracted from peripheral white blood cells using two kits, the Qiamp DNA Investigator Kit for coagulated samples and Qiamp DNA Mini & Blood Mini Kit for non-coagulated samples (QIAgen Systems, Inc., Valencia, CA, USA).	Human	blood	NA	male	Iberian Population in Spain	normal weight	Overweight and obesity	84	1.07	−0.47
EGAN00001618014	EGAN00001618014	Genomic DNA extracted from peripheral white blood cells using two kits, the Qiamp DNA Investigator Kit for coagulated samples and Qiamp DNA Mini & Blood Mini Kit for non-coagulated samples (QIAgen Systems, Inc., Valencia, CA, USA).	Human	blood	NA	male	Iberian Population in Spain	normal weight	Overweight and obesity	82	1.29	0.39
EGAN00001618015	EGAN00001618015	Genomic DNA extracted from peripheral white blood cells using two kits, the Qiamp DNA Investigator Kit for coagulated samples and Qiamp DNA Mini & Blood Mini Kit for non-coagulated samples (QIAgen Systems, Inc., Valencia, CA, USA).	Human	blood	NA	male	Iberian Population in Spain	normal weight	Overweight and obesity	81	0.56	−0.75
EGAN00001618016	EGAN00001618016	Genomic DNA extracted from peripheral white blood cells using two kits, the Qiamp DNA Investigator Kit for coagulated samples and Qiamp DNA Mini & Blood Mini Kit for non-coagulated samples (QIAgen Systems, Inc., Valencia, CA, USA).	Human	blood	NA	male	Iberian Population in Spain	normal weight	Overweight and obesity	83	0.85	−0.42
EGAN00001618017	EGAN00001618017	Genomic DNA extracted from peripheral white blood cells using two kits, the Qiamp DNA Investigator Kit for coagulated samples and Qiamp DNA Mini & Blood Mini Kit for non-coagulated samples (QIAgen Systems, Inc., Valencia, CA, USA).	Human	blood	NA	male	Iberian Population in Spain	normal weight	Overweight and obesity	77	0.77	−0.59
EGAN00001618018	EGAN00001618018	Genomic DNA extracted from peripheral white blood cells using two kits, the Qiamp DNA Investigator Kit for coagulated samples and Qiamp DNA Mini & Blood Mini Kit for non-coagulated samples (QIAgen Systems, Inc., Valencia, CA, USA).	Human	blood	NA	male	Iberian Population in Spain	normal weight	Overweight and obesity	89	0.92	−0.44
EGAN00001618019	EGAN00001618019	Genomic DNA extracted from peripheral white blood cells using two kits, the Qiamp DNA Investigator Kit for coagulated samples and Qiamp DNA Mini & Blood Mini Kit for non-coagulated samples (QIAgen Systems, Inc., Valencia, CA, USA).	Human	blood	NA	male	Iberian Population in Spain	normal weight	Overweight and obesity	77	1.05	0.57
EGAN00001618020	EGAN00001618020	Genomic DNA extracted from peripheral white blood cells using two kits, the Qiamp DNA Investigator Kit for coagulated samples and Qiamp DNA Mini & Blood Mini Kit for non-coagulated samples (QIAgen Systems, Inc., Valencia, CA, USA).	Human	blood	NA	male	Iberian Population in Spain	normal weight	Overweight and obesity	81	2.64	−0.26
EGAN00001618021	EGAN00001618021	Genomic DNA extracted from peripheral white blood cells using two kits, the Qiamp DNA Investigator Kit for coagulated samples and Qiamp DNA Mini & Blood Mini Kit for non-coagulated samples (QIAgen Systems, Inc., Valencia, CA, USA).	Human	blood	NA	male	Iberian Population in Spain	normal weight	Overweight and obesity	80	0.61	−1.16
EGAN00001618022	EGAN00001618022	Genomic DNA extracted from peripheral white blood cells using two kits, the Qiamp DNA Investigator Kit for coagulated samples and Qiamp DNA Mini & Blood Mini Kit for non-coagulated samples (QIAgen Systems, Inc., Valencia, CA, USA).	Human	blood	NA	male	Iberian Population in Spain	normal weight	Overweight and obesity	78	0.73	−1.16
EGAN00001618023	EGAN00001618023	Genomic DNA extracted from peripheral white blood cells using two kits, the Qiamp DNA Investigator Kit for coagulated samples and Qiamp DNA Mini & Blood Mini Kit for non-coagulated samples (QIAgen Systems, Inc., Valencia, CA, USA).	Human	blood	NA	male	Iberian Population in Spain	normal weight	Overweight and obesity	74	0.71	−0.07
EGAN00001618024	EGAN00001618024	Genomic DNA extracted from peripheral white blood cells using two kits, the Qiamp DNA Investigator Kit for coagulated samples and Qiamp DNA Mini & Blood Mini Kit for non-coagulated samples (QIAgen Systems, Inc., Valencia, CA, USA).	Human	blood	NA	male	Iberian Population in Spain	normal weight	Overweight and obesity	82	1.4	−1.71
EGAN00001618025	EGAN00001618025	Genomic DNA extracted from peripheral white blood cells using two kits, the Qiamp DNA Investigator Kit for coagulated samples and Qiamp DNA Mini & Blood Mini Kit for non-coagulated samples (QIAgen Systems, Inc., Valencia, CA, USA).	Human	blood	NA	male	Iberian Population in Spain	normal weight	Overweight and obesity	75	1.06	0.46
EGAN00001618026	EGAN00001618026	Genomic DNA extracted from peripheral white blood cells using two kits, the Qiamp DNA Investigator Kit for coagulated samples and Qiamp DNA Mini & Blood Mini Kit for non-coagulated samples (QIAgen Systems, Inc., Valencia, CA, USA).	Human	blood	NA	male	Iberian Population in Spain	normal weight	Overweight and obesity	75	2.04	0.62
EGAN00001618027	EGAN00001618027	Genomic DNA extracted from peripheral white blood cells using two kits, the Qiamp DNA Investigator Kit for coagulated samples and Qiamp DNA Mini & Blood Mini Kit for non-coagulated samples (QIAgen Systems, Inc., Valencia, CA, USA).	Human	blood	NA	male	Iberian Population in Spain	normal weight	Overweight and obesity	75	0.94	−0.77
EGAN00001618028	EGAN00001618028	Genomic DNA extracted from peripheral white blood cells using two kits, the Qiamp DNA Investigator Kit for coagulated samples and Qiamp DNA Mini & Blood Mini Kit for non-coagulated samples (QIAgen Systems, Inc., Valencia, CA, USA).	Human	blood	NA	male	Iberian Population in Spain	normal weight	Overweight and obesity	95	2.56	0.57
EGAN00001618029	EGAN00001618029	Genomic DNA extracted from peripheral white blood cells using two kits, the Qiamp DNA Investigator Kit for coagulated samples and Qiamp DNA Mini & Blood Mini Kit for non-coagulated samples (QIAgen Systems, Inc., Valencia, CA, USA).	Human	blood	NA	male	Iberian Population in Spain	normal weight	Overweight and obesity	85	0.42	−0.32
EGAN00001618030	EGAN00001618030	Genomic DNA extracted from peripheral white blood cells using two kits, the Qiamp DNA Investigator Kit for coagulated samples and Qiamp DNA Mini & Blood Mini Kit for non-coagulated samples (QIAgen Systems, Inc., Valencia, CA, USA).	Human	blood	NA	male	Iberian Population in Spain	normal weight	Overweight and obesity	84	0.41	0.81
EGAN00001618031	EGAN00001618031	Genomic DNA extracted from peripheral white blood cells using two kits, the Qiamp DNA Investigator Kit for coagulated samples and Qiamp DNA Mini & Blood Mini Kit for non-coagulated samples (QIAgen Systems, Inc., Valencia, CA, USA).	Human	blood	NA	male	Iberian Population in Spain	normal weight	Overweight and obesity	86	0.42	−0.23
EGAN00001618032	EGAN00001618032	Genomic DNA extracted from peripheral white blood cells using two kits, the Qiamp DNA Investigator Kit for coagulated samples and Qiamp DNA Mini & Blood Mini Kit for non-coagulated samples (QIAgen Systems, Inc., Valencia, CA, USA).	Human	blood	NA	male	Iberian Population in Spain	normal weight	Overweight and obesity	84	0.41	−1
EGAN00001618033	EGAN00001618033	Genomic DNA extracted from peripheral white blood cells using two kits, the Qiamp DNA Investigator Kit for coagulated samples and Qiamp DNA Mini & Blood Mini Kit for non-coagulated samples (QIAgen Systems, Inc., Valencia, CA, USA).	Human	blood	NA	male	Iberian Population in Spain	normal weight	Overweight and obesity	82	0.4	−0.32
EGAN00001618034	EGAN00001618034	Genomic DNA extracted from peripheral white blood cells using two kits, the Qiamp DNA Investigator Kit for coagulated samples and Qiamp DNA Mini & Blood Mini Kit for non-coagulated samples (QIAgen Systems, Inc., Valencia, CA, USA).	Human	blood	NA	male	Iberian Population in Spain	normal weight	Overweight and obesity	86	0.43	−0.78
EGAN00001618035	EGAN00001618035	Genomic DNA extracted from peripheral white blood cells using two kits, the Qiamp DNA Investigator Kit for coagulated samples and Qiamp DNA Mini & Blood Mini Kit for non-coagulated samples (QIAgen Systems, Inc., Valencia, CA, USA).	Human	blood	NA	male	Iberian Population in Spain	normal weight	Overweight and obesity	87	0.93	−0.21
EGAN00001618036	EGAN00001618036	Genomic DNA extracted from peripheral white blood cells using two kits, the Qiamp DNA Investigator Kit for coagulated samples and Qiamp DNA Mini & Blood Mini Kit for non-coagulated samples (QIAgen Systems, Inc., Valencia, CA, USA).	Human	blood	NA	male	Iberian Population in Spain	normal weight	Overweight and obesity	74	0.36	−0.8
EGAN00001618037	EGAN00001618037	Genomic DNA extracted from peripheral white blood cells using two kits, the Qiamp DNA Investigator Kit for coagulated samples and Qiamp DNA Mini & Blood Mini Kit for non-coagulated samples (QIAgen Systems, Inc., Valencia, CA, USA).	Human	blood	NA	male	Iberian Population in Spain	normal weight	Overweight and obesity	88	0.68	0.17
EGAN00001618038	EGAN00001618038	Genomic DNA extracted from peripheral white blood cells using two kits, the Qiamp DNA Investigator Kit for coagulated samples and Qiamp DNA Mini & Blood Mini Kit for non-coagulated samples (QIAgen Systems, Inc., Valencia, CA, USA).	Human	blood	NA	male	Iberian Population in Spain	normal weight	Overweight and obesity	87	2.01	0.2
EGAN00001618039	EGAN00001618039	Genomic DNA extracted from peripheral white blood cells using two kits, the Qiamp DNA Investigator Kit for coagulated samples and Qiamp DNA Mini & Blood Mini Kit for non-coagulated samples (QIAgen Systems, Inc., Valencia, CA, USA).	Human	blood	NA	male	Iberian Population in Spain	normal weight	Overweight and obesity	85	0.69	0
EGAN00001618040	EGAN00001618040	Genomic DNA extracted from peripheral white blood cells using two kits, the Qiamp DNA Investigator Kit for coagulated samples and Qiamp DNA Mini & Blood Mini Kit for non-coagulated samples (QIAgen Systems, Inc., Valencia, CA, USA).	Human	blood	NA	male	Iberian Population in Spain	normal weight	Overweight and obesity	95	0.78	−0.06
EGAN00001618041	EGAN00001618041	Genomic DNA extracted from peripheral white blood cells using two kits, the Qiamp DNA Investigator Kit for coagulated samples and Qiamp DNA Mini & Blood Mini Kit for non-coagulated samples (QIAgen Systems, Inc., Valencia, CA, USA).	Human	blood	NA	male	Iberian Population in Spain	normal weight	Overweight and obesity	90	1.39	0.43
EGAN00001618042	EGAN00001618042	Genomic DNA extracted from peripheral white blood cells using two kits, the Qiamp DNA Investigator Kit for coagulated samples and Qiamp DNA Mini & Blood Mini Kit for non-coagulated samples (QIAgen Systems, Inc., Valencia, CA, USA).	Human	blood	NA	male	Iberian Population in Spain	normal weight	Overweight and obesity	74	0.2	−0.38
EGAN00001618043	EGAN00001618043	Genomic DNA extracted from peripheral white blood cells using two kits, the Qiamp DNA Investigator Kit for coagulated samples and Qiamp DNA Mini & Blood Mini Kit for non-coagulated samples (QIAgen Systems, Inc., Valencia, CA, USA).	Human	blood	NA	male	Iberian Population in Spain	normal weight	Overweight and obesity	76	3.38	−0.29
EGAN00001618044	EGAN00001618044	Genomic DNA extracted from peripheral white blood cells using two kits, the Qiamp DNA Investigator Kit for coagulated samples and Qiamp DNA Mini & Blood Mini Kit for non-coagulated samples (QIAgen Systems, Inc., Valencia, CA, USA).	Human	blood	NA	male	Iberian Population in Spain	normal weight	Overweight and obesity	92	3.12	−0.1
EGAN00001618045	EGAN00001618045	Genomic DNA extracted from peripheral white blood cells using two kits, the Qiamp DNA Investigator Kit for coagulated samples and Qiamp DNA Mini & Blood Mini Kit for non-coagulated samples (QIAgen Systems, Inc., Valencia, CA, USA).	Human	blood	NA	male	Iberian Population in Spain	normal weight	Overweight and obesity	80	1.45	−1.19
EGAN00001618046	EGAN00001618046	Genomic DNA extracted from peripheral white blood cells using two kits, the Qiamp DNA Investigator Kit for coagulated samples and Qiamp DNA Mini & Blood Mini Kit for non-coagulated samples (QIAgen Systems, Inc., Valencia, CA, USA).	Human	blood	NA	male	Iberian Population in Spain	normal weight	Overweight and obesity	85	2.21	0.32
EGAN00001618047	EGAN00001618047	Genomic DNA extracted from peripheral white blood cells using two kits, the Qiamp DNA Investigator Kit for coagulated samples and Qiamp DNA Mini & Blood Mini Kit for non-coagulated samples (QIAgen Systems, Inc., Valencia, CA, USA).	Human	blood	NA	male	Iberian Population in Spain	normal weight	Overweight and obesity	75	0.43	−0.56
EGAN00001618048	EGAN00001618048	Genomic DNA extracted from peripheral white blood cells using two kits, the Qiamp DNA Investigator Kit for coagulated samples and Qiamp DNA Mini & Blood Mini Kit for non-coagulated samples (QIAgen Systems, Inc., Valencia, CA, USA).	Human	blood	NA	male	Iberian Population in Spain	normal weight	Overweight and obesity	75	0.91	−0.04
EGAN00001618049	EGAN00001618049	Genomic DNA extracted from peripheral white blood cells using two kits, the Qiamp DNA Investigator Kit for coagulated samples and Qiamp DNA Mini & Blood Mini Kit for non-coagulated samples (QIAgen Systems, Inc., Valencia, CA, USA).	Human	blood	NA	male	Iberian Population in Spain	normal weight	Overweight and obesity	87	0.84	−0.29
EGAN00001618050	EGAN00001618050	Genomic DNA extracted from peripheral white blood cells using two kits, the Qiamp DNA Investigator Kit for coagulated samples and Qiamp DNA Mini & Blood Mini Kit for non-coagulated samples (QIAgen Systems, Inc., Valencia, CA, USA).	Human	blood	NA	male	Iberian Population in Spain	normal weight	Overweight and obesity	85	1.04	−1.21
EGAN00001618051	EGAN00001618051	Genomic DNA extracted from peripheral white blood cells using two kits, the Qiamp DNA Investigator Kit for coagulated samples and Qiamp DNA Mini & Blood Mini Kit for non-coagulated samples (QIAgen Systems, Inc., Valencia, CA, USA).	Human	blood	NA	male	Iberian Population in Spain	normal weight	Overweight and obesity	84	1.41	−1.11
EGAN00001618052	EGAN00001618052	Genomic DNA extracted from peripheral white blood cells using two kits, the Qiamp DNA Investigator Kit for coagulated samples and Qiamp DNA Mini & Blood Mini Kit for non-coagulated samples (QIAgen Systems, Inc., Valencia, CA, USA).	Human	blood	NA	male	Iberian Population in Spain	normal weight	Overweight and obesity	82	1.75	0.36
EGAN00001618053	EGAN00001618053	Genomic DNA extracted from peripheral white blood cells using two kits, the Qiamp DNA Investigator Kit for coagulated samples and Qiamp DNA Mini & Blood Mini Kit for non-coagulated samples (QIAgen Systems, Inc., Valencia, CA, USA).	Human	blood	NA	male	Iberian Population in Spain	normal weight	Overweight and obesity	88	2.89	−0.34
EGAN00001618054	EGAN00001618054	Genomic DNA extracted from peripheral white blood cells using two kits, the Qiamp DNA Investigator Kit for coagulated samples and Qiamp DNA Mini & Blood Mini Kit for non-coagulated samples (QIAgen Systems, Inc., Valencia, CA, USA).	Human	blood	NA	male	Iberian Population in Spain	normal weight	Overweight and obesity	92	3.59	−0.03
EGAN00001618055	EGAN00001618055	Genomic DNA extracted from peripheral white blood cells using two kits, the Qiamp DNA Investigator Kit for coagulated samples and Qiamp DNA Mini & Blood Mini Kit for non-coagulated samples (QIAgen Systems, Inc., Valencia, CA, USA).	Human	blood	NA	male	Iberian Population in Spain	normal weight	Overweight and obesity	75	0.66	−0.87
EGAN00001618056	EGAN00001618056	Genomic DNA extracted from peripheral white blood cells using two kits, the Qiamp DNA Investigator Kit for coagulated samples and Qiamp DNA Mini & Blood Mini Kit for non-coagulated samples (QIAgen Systems, Inc., Valencia, CA, USA).	Human	blood	NA	male	Iberian Population in Spain	normal weight	Overweight and obesity	82	0.68	−1.55
EGAN00001618057	EGAN00001618057	Genomic DNA extracted from peripheral white blood cells using two kits, the Qiamp DNA Investigator Kit for coagulated samples and Qiamp DNA Mini & Blood Mini Kit for non-coagulated samples (QIAgen Systems, Inc., Valencia, CA, USA).	Human	blood	NA	male	Iberian Population in Spain	normal weight	Overweight and obesity	88	4.41	0.13
EGAN00001618058	EGAN00001618058	Genomic DNA extracted from peripheral white blood cells using two kits, the Qiamp DNA Investigator Kit for coagulated samples and Qiamp DNA Mini & Blood Mini Kit for non-coagulated samples (QIAgen Systems, Inc., Valencia, CA, USA).	Human	blood	NA	male	Iberian Population in Spain	normal weight	Overweight and obesity	86	1.34	−0.22
EGAN00001618059	EGAN00001618059	Genomic DNA extracted from peripheral white blood cells using two kits, the Qiamp DNA Investigator Kit for coagulated samples and Qiamp DNA Mini & Blood Mini Kit for non-coagulated samples (QIAgen Systems, Inc., Valencia, CA, USA).	Human	blood	NA	male	Iberian Population in Spain	normal weight	Overweight and obesity	94	1.83	−1.37
EGAN00001618060	EGAN00001618060	Genomic DNA extracted from peripheral white blood cells using two kits, the Qiamp DNA Investigator Kit for coagulated samples and Qiamp DNA Mini & Blood Mini Kit for non-coagulated samples (QIAgen Systems, Inc., Valencia, CA, USA).	Human	blood	NA	male	Iberian Population in Spain	normal weight	Overweight and obesity	91	2.67	−0.04
EGAN00001618061	EGAN00001618061	Genomic DNA extracted from peripheral white blood cells using two kits, the Qiamp DNA Investigator Kit for coagulated samples and Qiamp DNA Mini & Blood Mini Kit for non-coagulated samples (QIAgen Systems, Inc., Valencia, CA, USA).	Human	blood	NA	male	Iberian Population in Spain	normal weight	Overweight and obesity	94	1.2	−0.22
EGAN00001618062	EGAN00001618062	Genomic DNA extracted from peripheral white blood cells using two kits, the Qiamp DNA Investigator Kit for coagulated samples and Qiamp DNA Mini & Blood Mini Kit for non-coagulated samples (QIAgen Systems, Inc., Valencia, CA, USA).	Human	blood	NA	male	Iberian Population in Spain	normal weight	Overweight and obesity	89	1.34	−1.06
EGAN00001618063	EGAN00001618063	Genomic DNA extracted from peripheral white blood cells using two kits, the Qiamp DNA Investigator Kit for coagulated samples and Qiamp DNA Mini & Blood Mini Kit for non-coagulated samples (QIAgen Systems, Inc., Valencia, CA, USA).	Human	blood	NA	male	Iberian Population in Spain	normal weight	Overweight and obesity	85	3.09	0.07
EGAN00001618064	EGAN00001618064	Genomic DNA extracted from peripheral white blood cells using two kits, the Qiamp DNA Investigator Kit for coagulated samples and Qiamp DNA Mini & Blood Mini Kit for non-coagulated samples (QIAgen Systems, Inc., Valencia, CA, USA).	Human	blood	NA	male	Iberian Population in Spain	normal weight	Overweight and obesity	87	1.07	−1.38
EGAN00001618065	EGAN00001618065	Genomic DNA extracted from peripheral white blood cells using two kits, the Qiamp DNA Investigator Kit for coagulated samples and Qiamp DNA Mini & Blood Mini Kit for non-coagulated samples (QIAgen Systems, Inc., Valencia, CA, USA).	Human	blood	NA	male	Iberian Population in Spain	normal weight	Overweight and obesity	91	2.07	−0.91
EGAN00001618066	EGAN00001618066	Genomic DNA extracted from peripheral white blood cells using two kits, the Qiamp DNA Investigator Kit for coagulated samples and Qiamp DNA Mini & Blood Mini Kit for non-coagulated samples (QIAgen Systems, Inc., Valencia, CA, USA).	Human	blood	NA	male	Iberian Population in Spain	normal weight	Overweight and obesity	82	1.87	−0.32
EGAN00001618067	EGAN00001618067	Genomic DNA extracted from peripheral white blood cells using two kits, the Qiamp DNA Investigator Kit for coagulated samples and Qiamp DNA Mini & Blood Mini Kit for non-coagulated samples (QIAgen Systems, Inc., Valencia, CA, USA).	Human	blood	NA	male	Iberian Population in Spain	normal weight	Overweight and obesity	78	1.59	−0.16
EGAN00001618068	EGAN00001618068	Genomic DNA extracted from peripheral white blood cells using two kits, the Qiamp DNA Investigator Kit for coagulated samples and Qiamp DNA Mini & Blood Mini Kit for non-coagulated samples (QIAgen Systems, Inc., Valencia, CA, USA).	Human	blood	NA	male	Iberian Population in Spain	normal weight	Overweight and obesity	92	3.62	−1.05
EGAN00001618069	EGAN00001618069	Genomic DNA extracted from peripheral white blood cells using two kits, the Qiamp DNA Investigator Kit for coagulated samples and Qiamp DNA Mini & Blood Mini Kit for non-coagulated samples (QIAgen Systems, Inc., Valencia, CA, USA).	Human	blood	NA	male	Iberian Population in Spain	normal weight	Overweight and obesity	100	2.79	−0.51
EGAN00001618070	EGAN00001618070	Genomic DNA extracted from peripheral white blood cells using two kits, the Qiamp DNA Investigator Kit for coagulated samples and Qiamp DNA Mini & Blood Mini Kit for non-coagulated samples (QIAgen Systems, Inc., Valencia, CA, USA).	Human	blood	NA	male	Iberian Population in Spain	normal weight	Overweight and obesity	91	1.88	−0.36
EGAN00001618071	EGAN00001618071	Genomic DNA extracted from peripheral white blood cells using two kits, the Qiamp DNA Investigator Kit for coagulated samples and Qiamp DNA Mini & Blood Mini Kit for non-coagulated samples (QIAgen Systems, Inc., Valencia, CA, USA).	Human	blood	NA	male	Iberian Population in Spain	normal weight	Overweight and obesity	88	0.99	−0.68
EGAN00001618072	EGAN00001618072	Genomic DNA extracted from peripheral white blood cells using two kits, the Qiamp DNA Investigator Kit for coagulated samples and Qiamp DNA Mini & Blood Mini Kit for non-coagulated samples (QIAgen Systems, Inc., Valencia, CA, USA).	Human	blood	NA	male	Iberian Population in Spain	normal weight	Overweight and obesity	89	1.77	−0.15
EGAN00001618073	EGAN00001618073	Genomic DNA extracted from peripheral white blood cells using two kits, the Qiamp DNA Investigator Kit for coagulated samples and Qiamp DNA Mini & Blood Mini Kit for non-coagulated samples (QIAgen Systems, Inc., Valencia, CA, USA).	Human	blood	NA	male	Iberian Population in Spain	normal weight	Overweight and obesity	89	2.45	−0.27
EGAN00001618074	EGAN00001618074	Genomic DNA extracted from peripheral white blood cells using two kits, the Qiamp DNA Investigator Kit for coagulated samples and Qiamp DNA Mini & Blood Mini Kit for non-coagulated samples (QIAgen Systems, Inc., Valencia, CA, USA).	Human	blood	NA	male	Iberian Population in Spain	normal weight	Overweight and obesity	69	0.733	0.03
EGAN00001618075	EGAN00001618075	Genomic DNA extracted from peripheral white blood cells using two kits, the Qiamp DNA Investigator Kit for coagulated samples and Qiamp DNA Mini & Blood Mini Kit for non-coagulated samples (QIAgen Systems, Inc., Valencia, CA, USA).	Human	blood	NA	male	Iberian Population in Spain	normal weight	Overweight and obesity	89	1,538	−0.9
EGAN00001618076	EGAN00001618076	Genomic DNA extracted from peripheral white blood cells using two kits, the Qiamp DNA Investigator Kit for coagulated samples and Qiamp DNA Mini & Blood Mini Kit for non-coagulated samples (QIAgen Systems, Inc., Valencia, CA, USA).	Human	blood	NA	male	Iberian Population in Spain	normal weight	Overweight and obesity	90	NA	−0.49
EGAN00001618077	EGAN00001618077	Genomic DNA extracted from peripheral white blood cells using two kits, the Qiamp DNA Investigator Kit for coagulated samples and Qiamp DNA Mini & Blood Mini Kit for non-coagulated samples (QIAgen Systems, Inc., Valencia, CA, USA).	Human	blood	NA	male	Iberian Population in Spain	normal weight	Overweight and obesity	123	3,857	−0.26
EGAN00001618078	EGAN00001618078	Genomic DNA extracted from peripheral white blood cells using two kits, the Qiamp DNA Investigator Kit for coagulated samples and Qiamp DNA Mini & Blood Mini Kit for non-coagulated samples (QIAgen Systems, Inc., Valencia, CA, USA).	Human	blood	NA	male	Iberian Population in Spain	normal weight	Overweight and obesity	NA	NA	0.13

**Online-only Table 2:** Dataset Description. This table collects information about the shared datasets at the EGA repository. It provides a unique name for each dataset and its description.

Dataset Name	Description	Data type	Technology	Case or Control	Number of samples	Accession number	DAC accession number
stepwise_sobrepesosyobesos_FEMALESyMALES_het_set_tomissing_ONLY_CASES	CASE SAMPLES USING QuantStudio 12 K Flex Real-Time PCR System (Thermo Fisher Scientific, Waltham, MA, USA)	Genotype	OpenArray	Case	657	EGAD00010001482	EGAC00001000779
stepwise_sobrepesosyobesos_FEMALESyMALES_het_set_tomissing_ONLY_CONTROL	CONTROL SAMPLES USING QuantStudio 12 K Flex Real-Time PCR System (Thermo Fisher Scientific, Waltham, MA, USA)	Genotype	OpenArray	Control	258	EGAD00010001481	EGAC00001000779
